# New Dual P-Glycoprotein (P-gp) and Human Carbonic Anhydrase XII (hCA XII) Inhibitors as Multidrug Resistance (MDR) Reversers in Cancer Cells

**DOI:** 10.1021/acs.jmedchem.2c01175

**Published:** 2022-10-21

**Authors:** Laura Braconi, Elisabetta Teodori, Chiara Riganti, Marcella Coronnello, Alessio Nocentini, Gianluca Bartolucci, Marco Pallecchi, Marialessandra Contino, Dina Manetti, Maria Novella Romanelli, Claudiu T. Supuran, Silvia Dei

**Affiliations:** † Department of Neuroscience, Psychology, Drug Research and Child Health - Section of Pharmaceutical and Nutraceutical Sciences, University of Florence, via Ugo Schiff 6, 50019Sesto Fiorentino (FI), Italy; ‡ Department of Oncology, University of Turin, Via Santena 5/bis, 10126Torino, Italy; § Department of Health Sciences - Clinical Pharmacology and Oncology Section, University of Florence, Viale Pieraccini 6, 50139Firenze, Italy; ∥ Department of Pharmacy - Drug Sciences, University of Bari “A. Moro”, via Orabona 4, 70125Bari, Italy

## Abstract

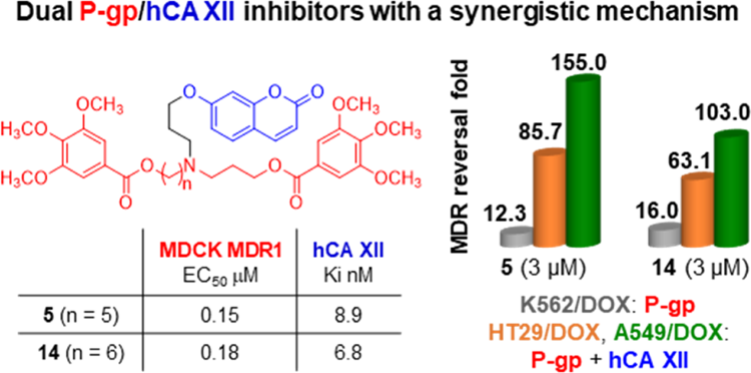

In a continuing search of dual P-gp and hCA XII inhibitors, we synthesized and studied new *N*,*N*-bis(alkanol)amine aryl diester derivatives characterized by the presence of a coumarin group. These hybrids contain both P-gp and hCA XII binding groups to synergistically overcome the P-gp-mediated multidrug resistance (MDR) in cancer cells expressing both P-gp and hCA XII. Indeed, hCA XII modulates the efflux activity of P-gp and the inhibition of hCA XII reduces the intracellular pH, thereby decreasing the ATPase activity of P-gp. All compounds showed inhibitory activities on P-gp and hCA XII proteins taken individually, and many of them displayed a synergistic effect in HT29/DOX and A549/DOX cells that overexpress both P-gp and hCA XII, being more potent than in K562/DOX cells overexpressing only P-gp. Compounds **5** and **14** were identified as promising chemosensitizer agents for selective inhibition in MDR cancer cells overexpressing both P-gp and hCA XII.

## Introduction

The expression of some ATP binding cassette (ABC) transporter proteins on the cell membrane is one of the main features of chemoresistant cancer cells.^1,2^ These energy-dependent transmembrane proteins act as extrusion pumps and reduce the intracellular concentration of anticancer drugs by actively transporting them out of tumor cells and, consequently, lowering their therapeutic efficacy.^3,4^ This phenomenon is one of the main causes of multidrug resistance (MDR), a condition in which cells acquire resistance, over the course of the treatment, to several anticancer drugs with different structures and mechanism of action.^5^ The main human ABC proteins associated with MDR are P-glycoprotein (P-gp), multidrug-resistance-associated protein-1 (MRP1), and breast cancer resistance protein (BCRP) whose expression in tumor cells has been correlated to poor patients’ prognosis in numerous studies.^6,7^

The most highly studied ABC transporter P-glycoprotein (P-gp) is widely overexpressed in human cancer tissues and plays an important role in removing chemotherapeutic agents from cells and decreasing the intracellular drug accumulation.^8^ Because of the importance of P-gp in the regulation of MDR and its clinical correlation, many efforts have been devoted to the development of novel P-gp inhibitors to reverse MDR.^9,10^ These compounds, also known as chemosensitizers, can restore the efficacy of anticancer agents, which are substrates of ABC transporters, when coadministered with them in resistant tumor cells.^11^ To date, many P-gp modulators have been identified that can be classified into three generations according to their chronology and characteristics;^12,13^ however, only a few of these compounds have entered clinical trials.^14^ The observed problems are mainly due to the presence of P-gp in several healthy tissues where it is responsible for various physiological and pharmacological effects.^15,16^ Furthermore, P-gp modulators could modify the pharmacokinetics of other coadministered substances such as chemotherapeutic agents.^17^

To reduce the alteration of the permeability of the normal tissue membranes, it is therefore desirable to develop structurally novel compounds capable of selectively inhibiting the P-gp efflux effect in resistant tumor cells.

A recent work^18^ reported that P-gp is colocalized and physically associated with the isoform XII of human carbonic anhydrase (hCA XII) on the membrane of several resistant cancer cells. The metalloenzymes carbonic anhydrases (CAs, EC 4.1.1.1) catalyze the conversion of carbon dioxide to bicarbonate and a proton. Human CAs (hCAs) include 15 isoforms of α-CA with different tissue distributions and cellular localization and play an important role in numerous physiological and pathological processes.^19^ Among these isoforms, hCA IX and XII are extracellular membrane-bound CAs overexpressed in many solid and hypoxic tumors and are associated with their progression and metastases formation.^20−22^ hCA IX and XII preserve an alkaline intracellular pH and extracellular acidosis, which promotes the growth of cancer cells, compromising that of normal cells.^23,24^ The intracellular alkalinization maintained by hCA XII is optimal for the efflux activity of P-gp. Therefore, the high expression of hCA XII in some chemoresistant P-gp-positive cancer cells^18^ contributes to MDR. Indeed, the pharmacological inhibition of hCA XII causes a decrease in the intracellular pH, which elicits a remarkable reduction in the ATPase activity of P-gp and consequently in the efflux activity of the transporter.^18,25^ Therefore, the development of compounds with a dual inhibition of P-gp and hCA XII could be a useful strategic approach to revert MDR in resistant tumor cells that overexpress both proteins. This synergistic mechanism may allow these compounds to act primarily in resistant tumors without interfering with the physiological function of P-gp.

**Figure 1:**
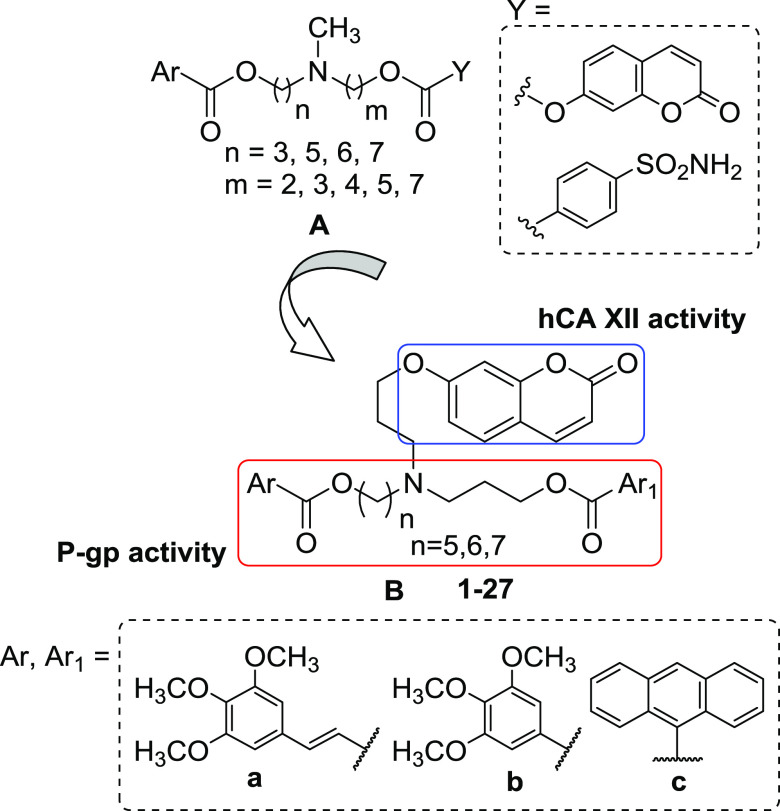
General structure of the leads (structure A) and the (*N*-alkylcoumarin)aminoaryl diester compounds **1–27** synthesized in this study (structure B).

In a previous study,^26^ we reported a series of compounds capable of inhibiting P-gp and hCA XII in tumor cells that overexpress both proteins. Indeed, these hybrids are characterized by an *N,N*-bis(alkanol)amine diester group functionalized with a different aryl nucleus (Ar), found in potent P-gp ligands,^27−30^ and a coumarin or benzene sulfonamide group (Y) to inhibit hCA XII^31−33^ (Figure 1, structure A). Many compounds displayed a multitarget activity against hCA XII and P-gp being active in the hCA XII inhibition test and in the rhodamine-123 (Rhd-123) uptake test in doxorubicin-resistant erythroleukemia K562 cells (K562/DOX) that overexpress only P-gp. Derivatives containing a coumarin residue were potent and selective hCA XII inhibitors and exhibited a modest inhibitory effect on P-gp in K562/DOX cells. Moreover, some coumarin compounds showed high MDR reversal effects on doxorubicin-resistant human colorectal carcinoma LoVo/DOX cells, which overexpress both P-gp and hCA XII proteins. These compounds were more potent as P-gp inhibitors in LoVo/DOX cells than in K562/DOX cells overexpressing only P-gp, showing a synergistic MDR reversal effect.^26^

To continue our project on dual P-gp/hCA XII inhibitors, we synthesized new derivatives containing the *N*,*N*-bis(alkanol)amine aryl diester scaffold to modulate the P-gp activity and a coumarin group to selectively target hCA XII, as observed in the first series.^26^ In this new series, the tertiary amino group is linked to two ester groups by a propyl and a 5-, 6-, or 7-methylene chain, while the coumarin moiety is connected through a propyl chain to the nitrogen atom by an ethereal bond. The aromatic ester groups inserted were a combination of (*E*)*-*3-(3,4,5-trimethoxyphenyl)vinyl, 3,4,5-trimethoxyphenyl, or anthracene residues (**a**–**c**) (Figure 1, structure B).

The new compounds **1–27**, as hydrochlorides, were first tested for their inhibitory effect on P-gp and hCA XII proteins taken individually. As regards the P-gp inhibition, all of these compounds were tested in the coadministration assay with doxorubicin in K562/DOX cells that overexpress only P-gp.^34^ To evaluate their hCA selectivity profiles, all of the synthesized compounds were studied on four different hCA isoforms (hCA I, II, IX, and XII).

Selected compounds were also tested in doxorubicin-resistant human adenocarcinoma colon cells (HT29/DOX) and doxorubicin-resistant non-small cell lung cancer cells (A549/DOX), which overexpress both P-gp and hCA XII:^18^ thus, the synergistic effect of these compounds was analyzed in a specific environment where these two proteins coexist. Moreover, to confirm the influence of the hCA XII catalytic effect on the P-gp efflux activity in MDR-resistant cells, doxorubicin cytotoxicity was evaluated in P-gp knockout (P-gp KO) and hCA XII knockout (hCA XII KO) HT29/DOX and A549/DOX cell lines. Then, compounds **5** and **14** were tested in the coadministration assay with doxorubicin in the same cell lines. In addition, the intracellular pH and doxorubicin accumulation were evaluated in all studied cell lines.

Finally, the chemical stability of these diester derivatives was investigated in phosphate-buffered solution (PBS) and human plasma samples.

## Results and Discussion

### Chemistry

**Scheme 1:**
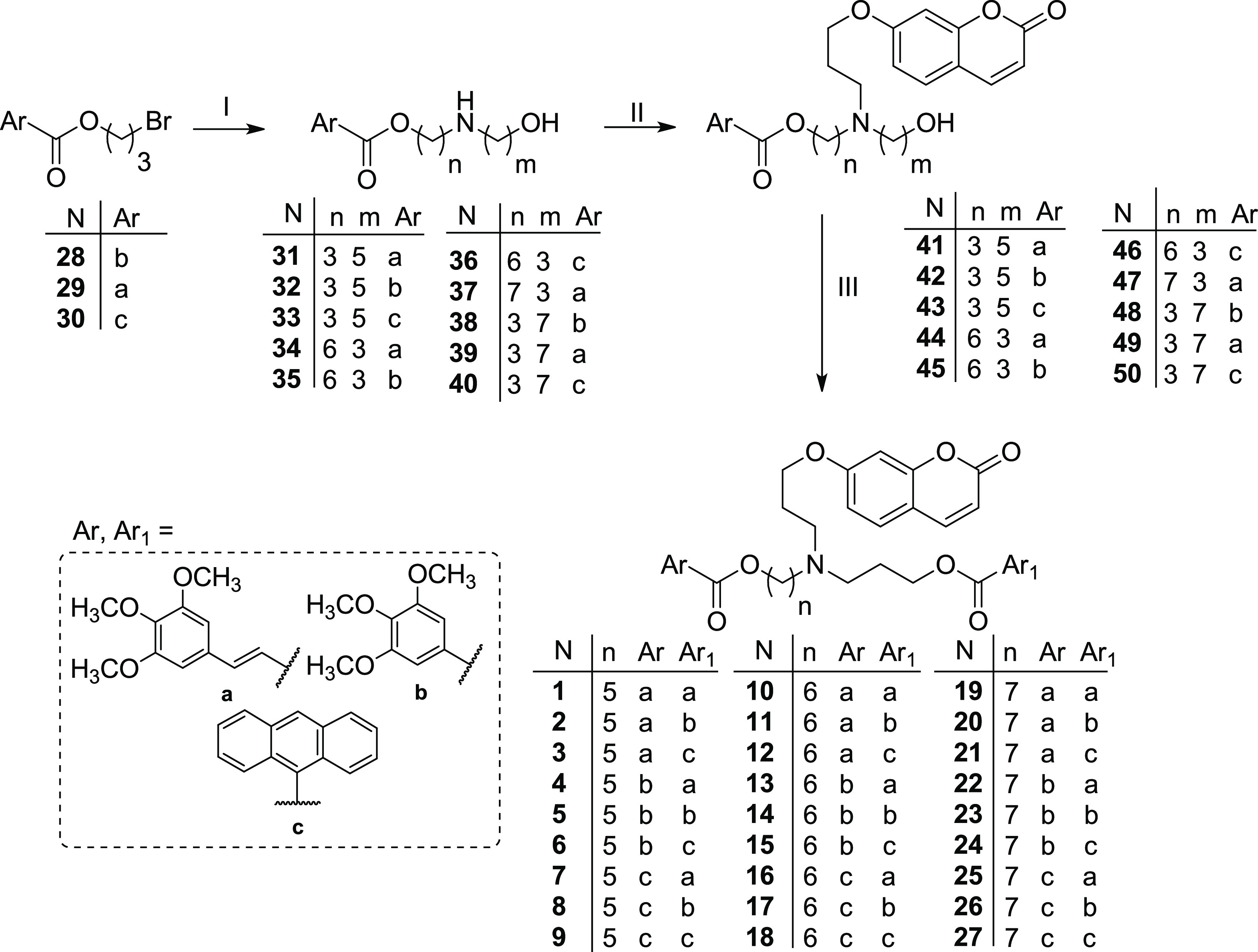
Reagents and conditions: (I) 7-aminoheptan-1-ol,^36^ K_2_CO_3_, dry CH_3_CN, 60 °C, overnight (yield 71–72%); (II) **51**, K_2_CO_3_, dry CH_3_CN, 60 °C, 20 h (yield 47–69%); (III) Ar_1_COOH, EDC hydrochloride, DMAP, dry CH_2_Cl_2_, rt, 48 h (method A) or Ar_1_COCl, CHCl_3_ (free of ethanol), rt, 18 h (method B) (yield 14–100%)

The reaction pathway used to synthesize the designed derivatives **1–27** is reported in Scheme 1. The (hydroxyalkyl)aminoesters **31–37** were previously synthesized by our group,^28,29,35^ while **38–40** were obtained by reaction of the proper bromoesters **28–30**(28,35) with 7-aminoheptan-1-ol^36^ in dry CH_3_CN, following standard procedures. Final compounds **1–27** were obtained starting from ((hydroxyalkyl)alkylcoumarin)aminoesters **41–50**, which were synthesized by the alkylation of the proper (hydroxyalkyl)aminoester with 7-(3-bromopropoxy)-2*H*-chromen-2-one (**51**) in dry CH_3_CN (Scheme 1). Finally, compounds **1–27** were obtained by esterification of **41–50** with the proper carboxylic acid ((*E*)-3-(3,4,5-trimethoxyphenyl)acrylic acid, 3,4,5-trimethoxybenzoic acid, or anthracene-9-carboxylic acid), using 1-ethyl-3-(3′-dimethylaminopropyl)carbodiimide (EDC) hydrochloride and 4-dimethylaminopyridine (DMAP) in dry CH_2_Cl_2_ or through the acyl chloride obtained by treatment of the suitable acid with SOCl_2_ in CHCl_3_ (free of ethanol),^37^ as reported in Scheme 1 (for details, see the Experimental Section). All free bases **1–27** were transformed into the corresponding hydrochlorides, which were used in the biological experiments and stability analysis (for details, see the Experimental Section).

**Scheme 2:**
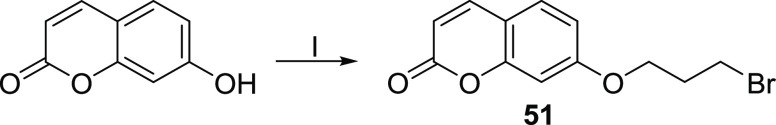
Reagents and conditions: (I) 1,3-dibromopropane, K_2_CO_3_, acetone, reflux, overnight (yield 92%)

Compound **51** was obtained by reaction of the commercially available 7-hydroxy-2*H*-chromen-2-one with 1,3-dibromopropane in acetone with very good yields, as reported in Scheme 2.

### Chemical Stability Tests

The chemical stability of all of these diester derivatives was evaluated in phosphate-buffered solution (PBS) and human plasma samples. The stability analyses were performed by liquid chromatography coupled with a triple quadrupole mass spectrometry system (LC-MS/MS), operating in multiple reaction monitoring (MRM) mode. The LC-MS/MS instrument and parameters used are reported in the Supporting Information.

In these assays, we monitored the variation of our diester molecules’ concentration at four different incubation times both in PBS and in human plasma samples to evaluate their susceptibility toward spontaneous or enzymatic hydrolysis, respectively. By plotting any variation of analyte concentration *vs* the incubation time, the corresponding degradation profiles in both the tested matrices were obtained. The analyte concentration (1 μM) used during the stability tests is generally smaller than its Michaelis–Menten constant (*K*_M_), and the enzymatic degradation rate is described by a first-order kinetic. Therefore, by plotting the natural logarithm of the quantitative data versus the incubation time, a linear function can be used, and its slope represents the degradation rate constant (*k*). Accordingly, with the linear function, the half-life (*t*_1/2_) of each tested compound can be calculated as follows
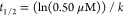


The plots of the natural logarithm of the quantitative data versus the incubation time of all of the studied compounds were analyzed. Results demonstrated that all of these compounds were stable both in PBS and in human plasma samples. The *k* values of all our compounds were close to 0, yielding extremely high *t*_1/2_ values. Since under the proposed experimental conditions a half-life over 240 min is not properly assessed, it is reasonable to consider that their *t*_1/2_ values could be equal to or greater than 240 min. The degradation profiles of all of these molecules in both the tested media are reported in the Supporting Information. The *t*_1/2_ value ≤2 h of ketoprofen ethyl ester (KEE), used as a reference compound, demonstrated that the employed human batch was enzymatically active.

### CA Inhibitory Activity

Compounds **1–27** were tested on four hCA isoforms, the cytosolic hCA I and II, and the tumor-associated transmembrane hCA IX and XII isoforms by a stopped-flow CO_2_ hydrase assay.^38^ The hCA inhibition data of the new compounds are reported in Table 1 together with those of acetazolamide (AAZ), used as a standard inhibitor. Results show that all of these derivatives were inactive against the off-target hCA I and II isoforms, while they inhibited both hCA IX and XII at nanomolar concentrations. As expected, the presence of the coumarin group addresses the activity only to hCA IX and XII.^39,40^ Interestingly, in this series of compounds, the interaction with hCA XII seems to be influenced by the length of the linkers: indeed, derivatives **1–18**, carrying a total spacer of 8 or 9 methylenes (*n* = 5 or 6), displayed preference toward hCA XII, except for **3**, **10**, and **12**; compounds characterized by *n* = 7 were generally more active on hCA IX, except for **22**, **26**, and **27**. Compounds **3**, **10**, and **20** were more potent on the hCA IX isoform than AAZ, showing *K*_i_ values < 10 nM.

**Table 1:**
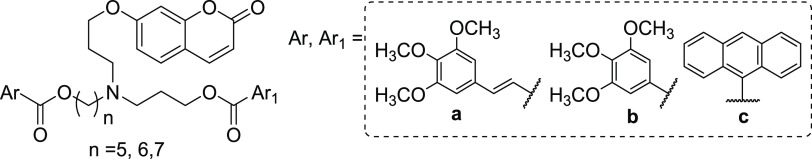
Inhibitory Activity on hCA I, II, IX, and XII Isoforms and Doxorubicin Cytotoxicity Enhancement Effect in K562/DOX Cells of Compounds **1–27** and of the Two Reference Compounds Acetazolamide (AAZ) and Verapamil (Ver)

compounds				*K*_i_ (nM)^a^				RF^b^	
N	*n*	Ar	Ar_1_	hCA I	hCA II	hCA IX	hCA XII	1 μM	3 μM
**1**	5	a	a	>10 000	>10 000	39.5	34.6	9.5	25.7
**2**	5	a	b	>10 000	>10 000	24.2	16.2	3.3	7.7
**3**	5	a	c	>10 000	>10 000	7.9	44.9	3.1	8.4
**4**	5	b	a	>10 000	>10 000	58.8	22.3	6.9	25.7
**5**	5	b	b	>10 000	>10 000	40.7	8.9	5.2	12.3
**6**	5	b	c	>10 000	>10 000	36.2	30.8	3.1	7.7
**7**	5	c	a	>10 000	>10 000	104.5	56.4	3.6	8.9
**8**	5	c	b	>10 000	>10 000	93.7	55.2	2.4	7.1
**9**	5	c	c	>10 000	>10 000	136.8	73.4	1.0	1.0
**10**	6	a	a	>10 000	>10 000	8.1	32.4	22.5	30.0
**11**	6	a	b	>10 000	>10 000	50.2	21.6	8.2	45.0
**12**	6	a	c	>10 000	>10 000	26.8	66.3	2.2	9.0
**13**	6	b	a	>10 000	>10 000	71.7	10.1	1.1	11.1
**14**	6	b	b	>10 000	>10 000	82.7	6.8	6.4	16.0
**15**	6	b	c	>10 000	>10 000	54.1	43.3	3.9	8.9
**16**	6	c	a	>10 000	>10 000	148.3	74.0	4.3	13.6
**17**	6	c	b	>10 000	>10 000	123.2	23.9	4.4	9.3
**18**	6	c	c	>10 000	>10 000	166.5	90.2	1.2	1.3
**19**	7	a	a	>10 000	>10 000	27.8	50.9	1.8	26.7
**20**	7	a	b	>10 000	>10 000	5.2	17.2	8.0	26.7
**21**	7	a	c	>10 000	>10 000	14.2	37.6	2.0	3.0
**22**	7	b	a	>10 000	>10 000	43.8	4.6	16.0	22.8
**23**	7	b	b	>10 000	>10 000	18.3	31.7	8.0	20.0
**24**	7	b	c	>10 000	>10 000	38.5	62.5	2.3	6.1
**25**	7	c	a	>10 000	>10 000	71.1	113.1	3.0	6.4
**26**	7	c	b	>10 000	>10 000	41.3	10.1	2.0	6.1
**27**	7	c	c	>10 000	>10 000	102.2	83.8	1.0	2.4
**AAZ**				250.0	12.0	25.0	5.7		
**Ver**								1.2	3.0

a Mean from three different assays by a stopped-flow technique (errors were in the range of ±5–10% of the reported values).

b Inhibition of the P-gp transport activity in K562/DOX cells expressed as RF that is the ratio between the IC_50_ of doxorubicin alone and in the presence of modulators (RF = IC_50_ of doxorubicin – modulator/IC_50_ of doxorubicin + modulator).

Notably, compounds **5**, **14**, and **22** showed the highest potency toward hCA XII, with *K*_i_ values < 10 nM (*K*_i_ = 8.9, 6.8, and 4.6 nM, respectively), as compared with the reference compound AAZ. Compounds **5** and **14** have a 5- or 6-methylene chain length, respectively, and the 3,4,5-trimethoxyphenyl ester residue (**b**) for both the aryl moieties; compound **22**, with a 7-methylene chain length, shows instead a combination of the (*E*)*-*3-(3,4,5-trimethoxyphenyl)vinyl (**a**) and the 3,4,5-trimethoxyphenyl (**b**) groups.

### Doxorubicin Cytotoxicity Enhancement Assay on K562/DOX Cells

The P-gp transport activity inhibition of compounds **1–27** was evaluated on the doxorubicin coadministration assay to assess their effects on the enhancement of the cytotoxicity of the antitumoral drug in the resistant K562/DOX cells, which overexpress only P-gp.^34^ K562 is a highly undifferentiated erythroleukemia cell line.^41^ The P-gp substrate, doxorubicin, is generally inactive in tumor cells, which express the protein, as it is expelled from the membrane by the pump.

Compounds were first studied, at 1, 3, and 10 μM concentrations, to evaluate their intrinsic cytotoxicity in both the parental K562 and the resistant K562/DOX cell lines, using the 3-(4,5-dimethylthiazolyl-2)-2,5-diphenyl tetrazolium bromide (MTT) assay.^42^ All compounds had no intrinsic cytotoxicity in the parental line and showed a toxicity not exceeding 20% in the resistant cells at the three concentrations tested (Supporting information, Figure S45).

The ability of compounds **1–27** to enhance the doxorubicin cytotoxicity in K562/DOX cells was assessed by evaluating the decrease of doxorubicin IC_50_ in the presence of 1 and 3 μM concentrations of the tested molecules. The results were expressed as RF (reversal fold) values that are the ratio between the IC_50_ value of doxorubicin alone and in the presence of the studied compounds: the higher the RF value, the higher the MDR reversal activity. Table 1 reports the RF values of compounds **1–27** in comparison with those of verapamil used as a standard reference. All our compounds enhanced the cytotoxicity of doxorubicin to a different extent and most of them showed higher RF values than those of verapamil. These data showed that the aryl moieties mainly influenced the P-gp inhibitory effects of these compounds since derivatives carrying the (*E*)*-*3-(3,4,5-trimethoxyphenyl)vinyl (**a**) and the 3,4,5-trimethoxyphenyl (**b**) groups gave the best results. Among these, the most potent compounds were **1**, **4**, and **5** (*n* = 5), **10**, **11**, and **14** (*n* = 6), and **20**, **22**, and **23** (*n* = 7), with RF values higher than 5.0 and 12.0 when used at 1 and 3 μM, respectively. Otherwise, the anthracene derivatives showed in general the lowest effects.

Notably, the potent P-gp inhibitors **5**, **14**, and **22** were also the most potent on hCA XII.

### Doxorubicin Cytotoxicity Enhancement Assay in HT29/DOX and A549/DOX Cells

To analyze the effect of these dual P-gp/hCA XII inhibitors in a specific environment where the two target proteins coexist, the most potent P-gp inhibitors bearing the aryl residues **a** and **b** (**1**, **2**, **4**, **5**, **10**, **11**, **13**, **14**, **19**, **20**, **22**, and **23**) were also tested in the doxorubicin cytotoxicity enhancement assay in doxorubicin-resistant human adenocarcinoma colon cells (HT29/DOX) and doxorubicin-resistant non-small cell lung cancer cells (A549/DOX), which overexpress both P-gp and hCA XII.^18^ Compounds carrying the anthracene moiety were not selected since they were the least active in the K562/DOX cell line test.

The expression levels of P-gp and hCA XII in sensitive HT29 and A549 cells and their resistant counterparts (HT29/DOX and A549/DOX cells) were checked by immunoblotting analysis, as described in the Experimental Section and reported in the Supporting information (Figure S44). The resistant sublines also showed an increased expression of MRP1 (Supporting information, Figure S44), another transporter involved in doxorubicin resistance, that, however, was not associated with hCA XII nor was affected in its efflux activity by hCA XII.^18^

The MTT assay was employed to evaluate the intrinsic cytotoxicity of the selected compounds at 1, 3, and 10 μM concentrations in the parental HT29 and A549 and the resistant HT29/DOX and A549/DOX cells, using the MTT assay.^42^ All compounds had no intrinsic cytotoxicity in the parental lines and showed a toxicity not exceeding 20% in the resistant lines at the three concentrations tested (Supporting information, Figure S46). Similarly, they did not reduce the viability by more than 20–25% in nontransformed epithelial colon EpiCoc and lung BEAS-2B cells at 10 μM (Supporting information, Figure S47). These data suggest that they could be used in the low micromolar range against cancer cells, in combination with classical chemotherapeutic drugs, without inducing toxic effects on nontransformed cells.

To verify this potential use, the ability of our compounds to increase the doxorubicin cytotoxicity was next evaluated by studying them at 1 and 3 μM in combination with the anticancer drug, and the RF values were measured (Table 2). All our compounds were able to restore the antineoplastic effect of doxorubicin, a typical substrate of P-gp, with highly reduced cell viability. The best results, in both the resistant cell lines (HT29/DOX and A549/DOX), were obtained for **5** and **14** (Ar, Ar_1_ = **b**, *n* = 5 and 6, respectively), **19** (Ar, Ar_1_ = **a**, *n* = 7), and **22** (Ar = **b**, Ar_1_ = **a**, *n* = 7), with RF values higher than 60 when used at 3 μM. Interestingly, all of these compounds showed RF values higher than those obtained in K562/DOX cells, which overexpress only P-gp, displaying a synergistic effect in the two resistant cell lines (HT29/DOX and A549/DOX) that overexpress both P-gp and hCA XII (see Tables 1 and 2). As an example, when **5** and **14** were tested at 3 μM in the A549/DOX cell line, they displayed RF values about 12 and 6 times higher than in K562/DOX cells, respectively (**5**: RF = 155.0 in A549/DOX and RF = 12.3 in K562/DOX; **14**: RF = 103.0 in A549/DOX and RF = 16.0 in K562/DOX). Compound **13** was the only one not showing a dose-dependent activity; in fact, it displayed a lower RF at 3 μM than at 1 μM in HT29/DOX and A549/DOX cells. This effect, however, was not detected in K562/DOX cells, devoid of hCA XII, differently from HT29/DOX and A549/DOX cells. We may hypothesize that **13** inhibits P-gp at both 1 and 3 μM, reversing doxorubicin resistance as in the case of K562/DOX. When both P-gp and hCA XII coexist, as in the case of HT29/DOX and A549/DOX cells, **13** regularly interfered with the P-gp/hCA XII complex reversing doxorubicin resistance at a low concentration (1 μM). At the higher concentration (3 μM), it may have a paradoxical loss of activity on hCA XII or even an activation, reducing its power as an agent counteracting doxorubicin resistance. Although this aspect requires further investigation, it is noteworthy that **13** is the only exception between the three cellular models analyzed, while all of the other compounds had a dose-dependent RF in all of the cell lines tested.

**Table 2:** RF Values of the 12 Selected Compounds in the Resistant HT29/DOX and A549/DOX Cell Lines

	HT29/DOX		A549/DOX	
	RF^a^		RF^a^	
compounds	1 μM	3 μM	1 μM	3 μM
**1**	14.9	18.7	21.5	30.2
**2**	38.4	44.5	54.0	64.3
**4**	21.7	41.1	39.5	65.0
**5**	44.4	85.7	70.4	155.0
**10**	40.2	47.5	65.1	85.4
**11**	10.8	29.7	17.6	49.8
**13**	14.1	4.15	16.5	11.6
**14**	46.2	63.2	67.4	103.0
**19**	60.5	87.0	92.2	131.6
**20**	13.1	16.2	19.2	31.5
**22**	37.6	61.9	61.9	82.4
**23**	27.2	26.6	37.0	41.3

a Inhibition of the P-gp transport activity on two resistant cell lines, expressed as RF that is the ratio between the IC_50_ of doxorubicin alone and in the presence of modulators (RF = IC_50_ of doxorubicin – modulator/IC_50_ of doxorubicin + modulator).

Thus, compounds **5** and **14** were selected for further studies as the best derivatives based on the results on the hCA XII isoform and in the K562/DOX, HT29/DOX, and A569/DOX cell lines. Interestingly, both carry the residue **b** as Ar and Ar_1_ moieties.

### Transport Inhibition of Fluorescent Probes in MDCK Transfected Cells

To further confirm the hypothesis that these derivatives were P-gp inhibitors, we tested the activity of compounds **5** and **14** on P-gp, MRP1, and BCRP in three Madin-Darby Canine Kidney (MDCK) transfected cell lines that overexpress the three proteins (P-gp, MRP1, or BCRP). The inhibiting activity of the two compounds on P-gp and MRP1 was evaluated by measuring the transport inhibition of the profluorescent probe calcein-AM (P-gp and MRP1 substrate) in MDCK-MDR1 and MDCK-MRP1 cells (P-gp- and MRP1-overexpressing cells, respectively). The activity on BCRP was instead evaluated using the fluorescent probe Hoechst 33342 (BCRP substrate) in MDCK-BCRP cells (BCRP-overexpressing cells).^43^

The results reported in Table 3 showed that compounds **5** and **14** inhibited the P-gp-mediated transport of calcein-AM, with EC_50_ values in the sub-micromolar range. Otherwise, they were completely inactive on the MRP1 and BCRP transporters.

**Table 3:** P-gp Interaction Activity of Compounds **5** and **14** in MDCK-MDR1, MDCK-MRP1, and MDCK-BCRP Cells Overexpressing P-gp, MRP1, and BCRP, Respectively

	EC_50_ μM^a^		
compounds	MDR1	MRP1	BCRP
**5**	0.15 ± 0.02	NA	NA
**14**	0.18 ± 0.03	NA	NA

a Values are the mean ± standard error of the mean (SEM) of two independent experiments, with samples in triplicate. NA, not active.

### Doxorubicin Cytotoxicity Enhancement Assay in P-gp Knockout (P-gp KO) and hCA XII Knockout (hCA XII KO) HT29/DOX and A549/DOX Cell Lines

To confirm the influence of the hCA XII catalytic effect on the P-gp efflux activity in MDR-resistant cells, P-gp and hCA XII were knocked out (KO) in resistant HT29/DOX and A549/DOX cell lines.

The expression levels of P-gp and hCA XII in P-gp KO and hCA XII KO HT29/DOX and A549/DOX cell lines were checked by immunoblotting, as described in the Experimental Section and reported in the Supporting information (Figure S44).

The results reported in Table 4 highlighted, as expected, that the IC_50_ doxorubicin values were much lower in P-gp KO cells than in wild-type-resistant HT29/DOX and A549/DOX cells (Table 4). The almost complete absence of the protein determines an increase in the cytotoxicity of doxorubicin also compared to the sensitive HT29 and A549 cells (Table 4), where the basal levels of P-gp confer a weak constitutive resistance to the drug. In P-gp KO HT29/DOX and A549/DOX cells, the coadministration of doxorubicin with 1 μM of the selected compounds **5** and **14** resulted in IC_50_ values lower than those of doxorubicin alone, except for **14** in P-gp KO A549/DOX cells (Table 4). The reduction of the IC_50_ values is likely due to the sum of the increase in the accumulation of doxorubicin, caused by the knockout of P-gp, and by an additional effect due to the inhibition of hCA XII exerted by **5** and **14**. The explanation of this effect requires further investigations.

**Table 4:** Doxorubicin Cytotoxicity in All Studied HT29 and A549 Cell Lines and RF Values in the Presence of Compounds **5** and **14** in P-gp KO and hCA XII KO HT29/DOX and A549/DOX Cell Lines

	IC_50_ μM							
treatment^a^	HT29	HT29/DOX	A549	A549/DOX				
DOX	1.38 ± 0.03	12.00 ± 0.85	2.38 ± 0.009	15.50 ± 0.93				

	HT29/DOX		HT29/DOX		A549/DOX		A549/DOX	
	P-gp KO		hCA XII KO		P-gp KO		hCA XII KO	
treatment^a^	IC_50_ μM	RF^b^	IC_50_ μM	RF^b^	IC_50_ μM	RF^b^	IC_50_ μM	RF^b^
DOX	0.46 ± 0.013		1.30 ± 0.06		0.9 ± 0.013		5.44 ± 1.5	
DOX + **5**	0.15 ± 0.044	3.1	1.30 ± 0.11	1	0.16 ± 0.012	6.0	4.79 ± 0.09	1.1
DOX + **14**	0.14 ± 0.009	3.3	1.25 ± 0.2	1	0.93 ± 0.015	1.03	4.10 ± 0.012	1.3

a Compounds **5** and **14** were tested at a 1 μM concentration.

b Inhibition of the P-gp transport activity on knockout cell lines expressed as RF that is the ratio between the IC_50_ of doxorubicin alone and with modulators (RF = IC_50_ of doxorubicin – modulator/IC_50_ of doxorubicin + modulator).

In hCA XII KO cells, doxorubicin IC_50_ values are similar or even slightly higher than those in the sensitive HT29 and A549 cells, and the coadministration with **5** and **14** did not significantly enhance the cytotoxicity of doxorubicin (Table 4). These results suggest that the complete absence of hCA XII impairs the efflux activity of P-gp that is expressed in these resistant cells; therefore, our dual inhibitors did not show any effect. Based on these results, we propose that our inhibitors show the maximal efficacy in cancer cells expressing both hCA XII and P-gp.

### Intracellular pH and Doxorubicin Accumulation

The intracellular pH (pHi) was measured in sensitive, wild-type-resistant, and P-gp KO- and hCA XII KO-resistant HT29 and A549 cells by a pH-sensitive fluorescent probe, and the results are reported in Table 5. The pHi value of the resistant HT29/DOX and A549/DOX cell lines was confirmed to be more alkaline than that of the parental counterparts (HT29 and A549). P-gp KO-resistant cells show pHi values like those of the wild-type-resistant HT29/DOX and A549/DOX cells: this result was expected since P-gp has never been reported to alter pHi. Otherwise, hCA XII KO-resistant cells had a pHi similar to that of sensitive cells, demonstrating the crucial role of hCA XII in regulating the pH of resistant cells, as previously reported.^18^

**Table 5:** Intracellular pH (pHi) Values of Sensitive, Wild-Type Resistant, and P-gp KO- and hCA XII KO-Resistant HT29 and A549 Cells

cell lines	pHi^a^	cell lines	pHi^a^
HT29	7.41 ± 0.05	A549	7.39 ± 0.03
HT29/DOX	7.64 ± 0.06	A549/DOX	7.66 ± 0.06
HT29/DOX KO P-gp	7.61 ± 0.08	A549/DOX KO P-gp	7.69 ± 0.05
HT29/DOX KO CAXII	7.42 ± 0.07	A549/DOX KO CAXII	7.41 ± 0.03

a The pHi was measured by a pH-sensitive fluorescent probe. Data are means ± standard deviation (SD) (*n* = 3).

**Figure 2:**
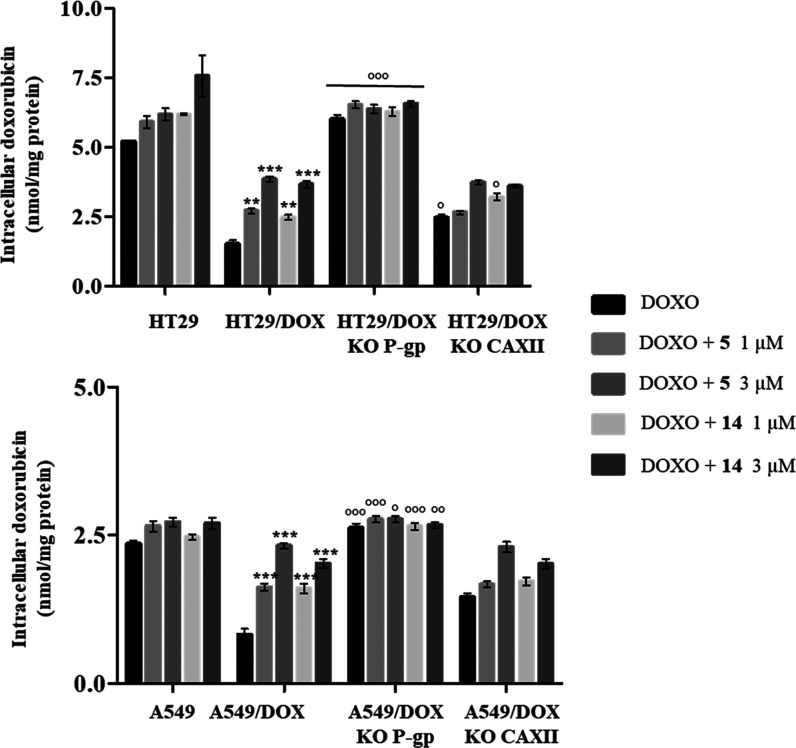
Intracellular accumulation of doxorubicin in sensitive, wild-type-resistant, and P-gp KO- and hCA XII KO-resistant cells. The cells were incubated for 24 h with 5 μM doxorubicin, with and without compounds **5** and **14** at 1 and 3 μM. The intracellular drug retention was measured spectrofluorimetrically. Data are means ± SD (*n* = 3). **p < 0.01, ***p < 0.001: versus wild-type HT29/DOX or A549/DOX cells treated with doxorubicin alone. °*p* < 0.05, °°*p* < 0.01, °°°*p* < 0.001: P-gp KO HT29/DOX or A549/DOX cells versus wild-type HT29/DOX or A549/DOX cells.

Considering that the slightly alkaline pH maintained by hCA XII promotes the P-gp efflux activity,^18^ we next measured the intracellular retention of doxorubicin alone and in the presence of 1 and 3 μM of compounds **5** and **14** in HT29 and A549 in their resistant counterparts (HT29/DOX and A549/DOX) and the corresponding resistant P-gp KO and hCA XII KO cell lines (Figure 2). As expected, HT29/DOX and A549/DOX cells, compared to HT29 and A549 cells, showed reduced intracellular retention of doxorubicin that was increased by both compounds **5** and **14** in a dose-dependent way. The intracellular accumulation of doxorubicin was lower in A549/DOX cells than in HT29/DOX, likely because of the slightly basal expression of MRP1^44^ (Figure S1), another transporter that can contribute to doxorubicin efflux.^4^ As expected, the accumulation of doxorubicin increased in P-gp KO cells, resembling sensitive counterparts. In these KO cells, compounds **5** and **14** did not significantly increase the retention of doxorubicin because of the absence of their first target P-gp.

In hCA XII KO cell lines, the intracellular concentration of doxorubicin was slightly higher than that in wild-type HT29/DOX and A549/DOX cell lines because the absence of hCA XII impaired the P-gp efflux activity. The presence of compounds **5** and **14** at 1 μM did not significantly increase the retention of doxorubicin, as evidenced also by the low RF values evaluated in these cells (Table 4). At the highest concentration tested (3 μM), our compounds caused an increase in doxorubicin intracellular concentration compared to doxorubicin administered alone.

### Kinetic Parameters of Doxorubicin Efflux in HT29 and A549, Their Wild-Type-Resistant Counterparts (HT29/DOX and A549/DOX), and the Corresponding hCA XII KO Cell Lines

Based on the previous results, we hypothesize that compounds **5** and **14** impaired the efflux kinetics of doxorubicin, thus increasing drug retention and toxicity. The results reported in Table 6 showed that the *K*_m_ of doxorubicin was increased by both compounds in the tested cells, suggesting that doxorubicin displayed a reduced affinity for P-gp. In hCA XII KO-resistant cells, they did not modify the *K*_m_ of doxorubicin compared to the wild-type-resistant cells, highlighting that the absence of hCA XII did not affect the affinity of doxorubicin for P-gp.

**Table 6:** Effects of Compounds **5** and **14** on Kinetic Parameters of Doxorubicin Efflux in HT29 and A549, Their Wild-Type-Resistant Counterparts (HT29/DOX and A549/DOX), and the Corresponding hCA XII KO Cell Lines^a^^b^

	HT29		HT29/DOX		hCA XII KO HT29/DOX	
	*K*_m_	*V*_max_	*K*_m_	*V*_max_	*K*_m_	*V*_max_
Doxo	0.55 ± 0.028	3.21 ± 0.04	0.54 ± 0.01	8.10 ± 0.15	0.58 ± 0.03	5.47 ± 0.12
Doxo + **5**(1 μM)	0.66 ± 0.009	3.29 ± 0.06	0.66 ± 0.009	5.30 ± 0.09	0.66 ± 0.01	4.30 ± 0.06
Doxo + **5**(3 μM)	0.72 ± 0.022^c^	3.12 ± 0.10	0.72 ± 0.009^d^	3.21 ± 0.05	0.74 ± 0.01^d^	3.22 ± 0.075
Doxo + **14**(1 μM)	0.64 ± 0.014	3.21 ± 0.08	0.63 ± 0.009	4.73 ± 0.25	0.67 ± 0.01	4.27 ± 0.11
Doxo + **14**(3 μM)	0.72 ± 0.009^d^	3.28 ± 0.07	0.73 ± 0.009^d^	3.80 ± 0.19	0.74 ± 0.008^d^	3.45 ± 0.075

	A549		A549/DOX		hCA XII KO A549/DOX	
	*K*_m_	*V*_max_	*K*_m_	*V*_max_	*K*_m_	*V*_max_
Doxo	0.47 ± 0.017	2.35 ± 0.08	0.45 ± 0.019	7.51 ± 0.31	0.55 ± 0.028	4.08 ± 0.32
Doxo + **5**(1 μM)	0.63 ± 0.013	2.36 ± 0.06	0.61 ± 0.015	5.10 ± 0.068	0.69 ± 0.021	3.75 ± 0.06
Doxo + **5**(3 μM)	0.75 ± 0.012^c^	2.35 ± 0.09	0.73 ± 0.012^c^	3.98 ± 0.077	0.75 ± 0.012^d^	2.74 ± 0.077
Doxo + **14**(1 μM)	0.64 ± 0.009	2.43 ± 0.09	0.58 ± 0.014	5.27 ± 0.09	0.62 ± 0.012	3.60 ± 0.09
Doxo + **14**(3 μM)	0.77 ± 0.02^c^	2.41 ± 0.07	0.68 ± 0.013^c^	4.14 ± 0.12	0.74 ± 0.016^d^	2.92 ± 0.09

a Cells were grown in the absence and presence of compounds **5** and **14** at 1 and 3 μM, respectively, with increasing concentrations of doxorubicin for 24 h. *K*_m_ (μM) and *V*_max_ (μmoles/min) were calculated with the Enzfitter software.

b Data are presented as means ± SD.

c *p* < 0.05.

d *p* < 0.01: versus doxo-treated cells.

Compounds **5** and **14** reduced the maximal velocity (*V*_max_) of the efflux in wild-type doxorubicin-resistant cell lines (HT29/DOX and A549/DOX cells), which was higher than in sensitive counterparts (HT29 and A549). The *V*_max_ values of doxorubicin in KO hCA XII-resistant cells were intermediate between those of sensitive and resistant cell lines, indicating that the absence of hCA XII reduced the maximal catalytic efficiency of doxorubicin efflux. No additional effects were observed in the presence of compounds **5** and **14** compared to wild-type HT29/DOX and A549/DOX since the compounds lacked one of their targets, hCA XII (Table 6). These data, again, indicated that the maximal efficacy of the compounds is achieved in the cells expressing both P-gp and hCA XII.

Overall, our results suggest that our compounds are maximally active when cancer cells coexpress both P-gp and hCA XII. While P-gp is widely diffused in normal tissues,^4^ hCA XII is an isoform mainly expressed in tumors.^45^ Exploiting this preferential expression, the dual P-gp and hCA XII inhibition proposed in this study is a reasonably safe and selective approach to target mostly cancer cells, sparing nontransformed tissues, with low or no expression of hCA XII. It is worth noting that several small molecules^46−48^ or monoclonal antibodies directed against hCA XII^49,50^ have shown a direct antitumor effect. Indeed, the study of hCA IX and hCA XII interactomes revealed that these enzymes are central regulators in cancer cell proliferation and migration, thanks to their activity on pH control in the tumor microenvironment. As an example, the dual hCA IX/XII inhibitor SLC-0111 is actually in phase Ib/II clinical trials for antitumor and antimetastasizing activity,^51^ and prompted by these promising results, the first hCA XII small inhibitors conjugated with monoclonal antibodies against hCA XII have been designed and proposed as strong antitumor agents.^52^ However, differently from these latest compounds, we did not observe a direct anticancer effect of our compounds. By contrast, we focused on the compounds used in the low micromolar range and evaluated their behavior in coadministration with classical chemotherapeutic drugs. Indeed, some hCA XII inhibitors also restored chemotherapeutic drug efficacy by controlling the pHi values, as for instance acetazolamide.^53,54^ Also, the activity of P-gp is deeply influenced by pHi, being higher at a slightly alkaline pH.^55^ These findings are in line with our observation, showing a higher *V*_max_, corresponding to a higher catalytic activity, in HT29/DOX and A549/DOX cells that have higher pHi than their sensitive counterpart, caused by the increased expression of hCA XII.^25^ By inhibiting the catalytic activity of hCA XII, compounds **5** and **14** create an unfavorable membrane environment for P-gp, contributing to a reduced efflux activity. The results of the present work are in line with several other pieces of evidence reporting that hCA XII inhibitors have an indirect inhibition on P-gp activity, as reported in references.^56,57^

Notably, P-gp and hCA XII are often coexpressed, as demonstrated in doxorubicin-resistant colon, lung, breast cancer, and osteosarcoma cell lines.^18,25^ Moreover, in glioblastoma, the two proteins are expressed in the drug-resistant stem cell component more than in the drug-sensitive, well-differentiated cells.^57^ The novelty of our compounds relies on the fact that they simultaneously inhibit P-gp and hCA XII. As proved by the assay in resistant cells selectively knocked out for P-gp or hCA XII, the inhibitors lost their maximal efficacy if one of these two actors is missing. This feature makes the compounds particularly promising as chemosensitizer agents in the most aggressive and drug-resistant tumors that coexpress both P-gp and hCA XII. This is the case of tumors rich in stem cells, which are often responsible for tumor relapse, metastization, and generation of drug-resistance clones,^58−60^ or hypoxic tumors that are the most invasive and resistant to the conventional therapies in use,^61−63^ where the transcription factor HIF-1α transcriptionally upregulates P-gp and hCA XII.^18^

## Conclusions

In this study, we reported new dual P-gp/hCA XII inhibitors based on the evidence that in several MDR cancer cells P-gp is colocalized to hCA XII and that the hCA XII catalytic activity modulates the P-gp efflux activity. The structure of these hybrid inhibitors contains both P-gp and hCA XII binding moieties to synergistically overcome the P-gp-mediated MDR in cancer cells that overexpress both proteins; thus, they presented the *N*,*N*-bis(alkanol)amine aryl diester group carrying a coumarin group on the nitrogen atom. All compounds showed inhibitory activities on P-gp and hCA XII proteins taken individually; in fact, they were able to enhance the cytotoxicity of the anticancer drug doxorubicin in resistant K562/DOX cells that overexpress only P-gp and inhibited hCA XII at nanomolar concentrations.

The doxorubicin cytotoxicity enhancement assays in HT29/DOX and A549/DOX cell lines, which overexpress both P-gp and hCA XII, highlighted a synergistic effect of these compounds since the selected derivatives bearing the aryl residues **a** and **b** were able to restore the antineoplastic effect of doxorubicin with RF values higher than those obtained in K562/DOX cells that overexpress only P-gp. The P-gp inhibition activity of compounds **5** and **14** was also confirmed by the assay in MDCK transfected cells where the selectivity toward P-gp with respect to the other MDR sister proteins was proved.

The influence of hCA XII catalytic activity on the P-gp efflux activity in MDR-resistant cells was confirmed by the evaluation of the IC_50_ values of doxorubicin alone or in the presence of the selected two compounds, **5** and **14**, in P-gp knockout (P-gp KO) and hCA XII knockout (hCA XII KO) HT29/DOX and A549/DOX cell lines. In P-gp KO cells, the almost complete absence of P-gp determines an expected reduction in doxorubicin IC_50_ values when used alone; however, in the presence of compounds **5** and **14**, an increase in the cytotoxicity of doxorubicin is observed compared to that of resistant wild-type cells, probably due to the increase in the intracellular accumulation of doxorubicin caused by the absence of the transporter and by an additional effect due to the inhibition of hCA XII exerted by **5** and **14**. In hCA XII KO cells, doxorubicin IC_50_ values were similar to those in the sensitive cells, either when doxorubicin was used alone or in the presence of compounds **5** and **14** because the complete absence of hCA XII impairs the P-gp efflux activity and the dual inhibitors did not show any effect. These results confirmed that our inhibitors show the maximal efficacy in cancer cells expressing both hCA XII and P-gp.

Based on these results, we identified a new series of hybrid compounds that act as dual P-gp/hCA XII inhibitors with a synergistic mechanism. These compounds displayed a higher reversing activity in resistant tumor cells overexpressing both P-gp and hCA XII than in cells overexpressing only P-gp, in agreement with our evidence that the efflux activity of P-gp is modulated by the hCA XII catalytic activity.

In particular, compounds **5** and **14** resulted as promising P-gp-mediated MDR reversers characterized by the maximal efficacy in cancer cells expressing both hCA XII and P-gp proteins.

## Experimental Section

### Chemistry

#### General Information

All melting points were taken on a Büchi apparatus and are uncorrected. NMR spectra were recorded on a Bruker Avance 400 spectrometer (400 MHz for ^1^H NMR, 100 MHz for ^13^C NMR). ^1^H and ^13^C NMR spectra were measured at room temperature (25 °C) in an appropriate solvent. ^1^H and ^13^C chemical shifts are expressed in ppm (δ) referenced to tetramethylsilane (TMS). Spectral data are reported using the following abbreviations: s = singlet, d = doublet, dd = doublet of doublets, t = triplet, bs = broad singlet, m = multiplet, and coupling constants are reported in Hz, followed by integration. Assignments of the ^13^C signals were performed using the attached proton test (APT) technique. Chromatographic separations were performed on a silica gel column by flash chromatography (Kieselgel 40, 0.040–0.063 mm; Merck). Yields are given after purification unless otherwise stated. The high-resolution mass spectrometry (HRMS) analysis was performed with a Thermo Finnigan LTQ Orbitrap mass spectrometer equipped with an electrospray ionization source (ESI). The accurate mass/charge ratio measure was carried out by introducing, via a syringe pump at 10 μL min^–1^, the sample solution (1.0 μg mL^–1^ in mQ water: acetonitrile 50:50), and the signal of the positive ions was acquired. The proposed experimental conditions allowed monitoring the protonated molecules of studied compounds ([M + H]^+^ species), which were measured with a proper dwell time to achieve 60 000 units of resolution at full width at half-maximum (FWHM). The elemental composition of each compound was calculated based on its measured accurate mass/charge ratio, accepting only results with an attribution error of less than 2.5 ppm and a not integer double bond/ring equivalent (RDB) value to consider only the protonated species.^64^ All compounds are >95% pure as determined by high-performance liquid chromatography (HPLC)/diode-array detection (DAD) analysis: the specific analytical method used to determine purity and representative HPLC/DAD traces is included in the Supporting Information.

Compounds were named following IUPAC rules, as applied by ChemBioDraw Ultra 14.0 software. When reactions were performed in anhydrous conditions, the mixtures were maintained under nitrogen. Free bases **1–27** were transformed into the corresponding hydrochlorides by treatment with a solution of acetyl chloride (1.1 equiv) in anhydrous CH_3_OH. The salts were crystallized from abs. ethanol/petroleum ether.

#### General Procedure for the Synthesis of (Hydroxyalkyl)aminoesters (**38–40**)

To a solution of the proper bromoesters **28–30**(28,35) (1 equiv) in the adequate amount of dry CH_3_CN, K_2_CO_3_ (1 equiv) and 7-aminoheptan-1-ol^36^ (2 equiv) were added. The mixture was stirred at 60 °C overnight; then, the solvent was removed under reduced pressure and the residue was treated with CH_2_Cl_2_. The organic layer was washed twice with 10% NaOH solution, dried over Na_2_SO_4_, and concentrated under reduced pressure. Finally, the residue was purified by flash chromatography using CH_2_Cl_2_/CH_3_OH/NH_4_OH 90:10:1 as an eluent, yielding the desired (hydroxyalkyl)aminoester as a pale yellow oil.

##### 3-((7-Hydroxyheptyl)amino)propyl 3,4,5-Trimethoxybenzoate (**38**)

Following the general procedure, compound **38** (0.10 g, yield: 70.6%) was synthesized from **28**(35) (0.12 g, 0.37 mmol) and 7-aminoheptan-1-ol^36^ (0.10 g, 0.74 mmol) in 5.0 mL of dry CH_3_CN. ^1^H NMR (400 MHz, CDCl_3_) δ: 7.25 (s, 2H), 4.35 (t, *J* = 6.4 Hz, 2H), 3.87 (s, 9H), 3.57 (t, *J* = 6.8 Hz, 2H), 2.74 (t, *J* = 6.8 Hz, 2H), 2.59 (t, *J* = 7.2 Hz, 2H), 2.22 (bs, 2H), 1.99–1.93 (m, 2H), 1.50–1.47 (m, 4H), 1.30–1.15 (m, 6H) ppm.

##### (*E*)-3-((7-Hydroxyheptyl)amino)propyl 3-(3,4,5-Trimethoxyphenyl)acrylate (**39**)

Following the general procedure, compound **39** (0.17 g, yield: 71.0%) was synthesized from **29**(28) (0.21 g, 0.58 mmol) and 7-aminoheptan-1-ol^36^ (0.15 g, 1.17 mmol) in 6.0 mL of dry CH_3_CN. ^1^H NMR (400 MHz, CDCl_3_) δ: 7.51 (d, *J* = 16.0 Hz, 1H), 6.68 (s, 2H), 6.27 (d, *J* = 16.0 Hz, 1H), 4.20 (t, *J* = 6.0 Hz, 2H), 3.81 (s, 6H), 3.80 (s, 3H), 3.52 (t, *J* = 6.4 Hz, 2H), 2.66 (t, *J* = 6.8 Hz, 2H), 2.53 (t, *J* = 6.8 Hz, 2H), 2.06 (bs, 2H), 1.85–1.82 (m, 2H), 1.52–1.39 (m, 4H), 1.30–1.19 (m, 6H) ppm.

##### 3-((7-Hydroxyheptyl)amino)propyl Anthracene-9-carboxylate (**40**)

Following the general procedure, compound **40** (0.33 g, yield: 72.4%) was synthesized from **30**(35) (0.40 g, 1.17 mmol) and 7-aminoheptan-1-ol^36^ (0.31 g, 2.33 mmol) in 15.0 mL of dry CH_3_CN. ^1^H NMR (400 MHz, CDCl_3_) δ: 8.49 (s, 1H), 7.99 (t, *J* = 9.2 Hz, 4H), 7.53–7.43 (m, 4H), 4.66 (t, *J* = 6.4 Hz, 2H), 3.62 (bs, 2H), 3.53 (t, *J* = 6.4 Hz, 2H), 2.82 (t, *J* = 7.2 Hz, 2H), 2.59 (t, *J* = 7.2 Hz, 2H), 2.14–2.07 (m, 2H), 1.51–1.37 (m, 4H), 1.29–1.16 (m, 6H) ppm.

##### 7-(3-Bromopropoxy)-2*H*-chromen-2-one (**51**)

To a solution of 7-hydroxy-2*H*-chromen-2-one (0.40 g, 2.46 mmol) in 30.0 mL of acetone, K_2_CO_3_ (1.02 g, 7.39 mmol) and 1,3-dibromopropane (1.25 mL, 12.31 mmol) were added. The reaction was refluxed overnight; then, it was cooled to rt and the solvent was removed under reduced pressure. The residue was dissolved in CH_2_Cl_2_ and washed twice with water; then, the organic phase was dried over Na_2_SO_4_ and concentrated under vacuum. **51** (0.64 g, yield 91.5%) was obtained as a pure white solid. TLC: CH_2_Cl_2_/CH_3_OH 95:5. ^1^H NMR (400 MHz, CDCl_3_) δ: 7.61 (d, *J* = 9.6 Hz, 1H), 7.35 (d, *J* = 8.0 Hz, 1H), 6.83–6.80 (m, 2H), 6.23 (d, *J* = 9.6 Hz, 1H), 4.14 (t, *J* = 6.4 Hz, 2H), 3.58 (t, *J* = 6.4 Hz, 2H), 2.36–2.29 (m, 2H) ppm.

#### General Procedure for the Synthesis of ((Hydroxyalkyl)alkylcoumarin)aminoester (**41–50**)

The suitable (hydroxyalkyl)aminoester **31–40** (1 or 1.2 equiv) was dissolved in the adequate amount of dry CH_3_CN; then, K_2_CO_3_ (3 equiv) and **51** (1 or 1.2 equiv) were added. The mixture was stirred at 60 °C for 20 h; then, it was cooled to rt and the solvent was removed under reduced pressure. The residue was dissolved in CH_2_Cl_2_, and then the organic layer was washed twice with 10% NaOH solution, dried over Na_2_SO_4_, and concentrated under reduced pressure. The residue was purified by flash chromatography using the proper eluting system, yielding the desired compound as a pale yellow oil.

##### (*E*)-3-((5-Hydroxypentyl)(3-((2-oxo-2*H*-chromen-7-yl)oxy)propyl)amino)propyl 3-(3,4,5-Trimethoxyphenyl)acrylate (**41**)

Following the general procedure, compound **41** (0.30 g, yield: 57.7%) was synthesized from **31**(28) (0.34 g, 0.89 mmol) and **51** (0.37 g, 1.07 mmol) in 13.0 mL of dry CH_3_CN. Chromatographic eluent: CH_2_Cl_2_/CH_3_OH/NH_4_OH 97:3:0.3. ^1^H NMR (400 MHz, CDCl_3_) δ: 7.54 (d, *J* = 9.2 Hz, 1H), 7.51 (d, *J* = 16.0 Hz, 1H), 7.27 (d, *J* = 8.4 Hz, 1H), 6.77–6.75 (m, 2H), 6.68 (s, 2H), 6.25 (d, *J* = 16.0 Hz, 1H), 6.14 (d, *J* = 9.2 Hz, 1H), 4.17 (t, *J* = 6.4 Hz, 2H), 4.02 (t, *J* = 6.0 Hz, 2H), 3.82 (s, 6H), 3.81 (s, 3H), 3.57 (t, *J* = 6.4 Hz, 2H), 2.56 (t, *J* = 6.4 Hz, 2H), 2.50 (t, *J* = 6.4 Hz, 2H), 2.39 (t, *J* = 6.4 Hz, 2H), 2.00–1.83 (m, 3H), 1.82–1.74 (m, 2H), 1.52–1.37 (m, 4H), 1.35–1.26 (m, 2H) ppm.

##### 3-((5-Hydroxypentyl)(3-((2-oxo-2*H*-chromen-7-yl)oxy)propyl)amino)propyl 3,4,5-Trimethoxybenzoate (**42**)

Following the general procedure, compound **42** (0.16 g, yield: 53.0%) was synthesized from **32**(28) (0.23 g, 0.64 mmol) and **51** (0.15 g, 0.54 mmol) in 20.0 mL of dry CH_3_CN. Chromatographic eluent: CH_2_Cl_2_/CH_3_OH/NH_4_OH 95:5:0.5. ^1^H NMR (400 MHz, CDCl_3_) δ: 7.53 (d, *J* = 9.2 Hz, 1H), 7.26 (d, *J* =8.4 Hz, 1H), 7.21 (s, 2H), 6.75–6.73 (m, 2H), 6.14 (d, *J* = 9.2 Hz, 1H), 4.26 (t, *J* = 6.4 Hz, 2H), 4.00 (t, *J* = 6.4 Hz, 2H), 3.83 (s, 3H), 3.82 (s, 6H), 3.53 (t, *J* = 6.4 Hz, 2H), 2.56–2.49 (m, 4H), 2.37 (t, *J* = 7.2 Hz, 2H), 2.14 (bs, 1H), 1.90–1.80 (m, 4H), 1.49–1.36 (m, 4H), 1.35–1.26 (m, 2H) ppm.

##### 3-((5-Hydroxypentyl)(3-((2-oxo-2*H*-chromen-7-yl)oxy)propyl)amino)propyl Anthracene-9-carboxylate (**43**)

Following the general procedure, compound **43** (0.15 g, yield: 46.8%) was synthesized from **33**(28) (0.25 g, 0.69 mmol) and **51** (0.16 g, 0.58 mmol) in 20.0 mL of dry CH_3_CN. Chromatographic eluent: CH_2_Cl_2_/CH_3_OH/NH_4_OH 97:3:0.3. ^1^H NMR (400 MHz, CDCl_3_) δ: 8.46 (s, 1H), 7.97 (t, *J* = 8.0 Hz, 4H), 7.50–7.42 (m, 5H), 7.19 (d, *J* = 9.2 Hz, 1H), 6.73–6.70 (m, 2H), 6.15 (d, *J* = 9.2 Hz, 1H), 4.61 (t, *J* = 6.4 Hz, 2H), 3.97 (t, *J* = 6.4 Hz, 2H), 3.52 (t, *J* = 6.4 Hz, 2H), 2.60–2.54 (m, 4H), 2.40 (t, *J* = 7.2 Hz, 2H), 2.00–1.92 (m, 2H), 1.88–1.82 (m, 2H), 1.47–1.39 (m, 4H), 1.33–1.26 (m, 2H) ppm.

##### (*E*)-6-((3-Hydroxypropyl)(3-((2-oxo-2*H*-chromen-7-yl)oxy)propyl)amino)hexyl 3-(3,4,5-Trimethoxyphenyl)acrylate (**44**)

Following the general procedure, compound **44** (0.27 g, yield: 59.6%) was synthesized from **34**(29) (0.36 g, 0.91 mmol) and **51** (0.21 g, 0.76 mmol) in 27.0 mL of dry CH_3_CN. Chromatographic eluent: CH_2_Cl_2_/CH_3_OH/NH_4_OH 97:3:0.3. ^1^H NMR (400 MHz, CDCl_3_) δ: 7.59 (d, *J* = 9.2 Hz, 1H), 7.56 (d, *J* = 16.0 Hz, 1H), 7.33 (d, *J* = 8.8 Hz, 1H), 6.82–6.77 (m, 2H), 6.73 (s, 2H), 6.32 (d, *J* = 16.0 Hz, 1H), 6.21 (d, *J* = 9.2 Hz, 1H), 4.14 (t, *J* = 6.4 Hz, 2H), 4.03 (t, *J* = 6.4 Hz, 2H), 3.86 (s, 6H), 3.85 (s, 3H), 3.77 (t, *J* = 6.4 Hz, 2H), 2.66–2.59 (m, 4H), 2.44 (t, *J* = 7.2 Hz, 2H), 2.00–1.94 (m, 2H), 1.72–1.62 (m, 4H), 1.51–1.44 (m, 2H), 1.41–1.28 (m, 4H) ppm.

##### 6-((3-Hydroxypropyl)(3-((2-oxo-2*H*-chromen-7-yl)oxy)propyl)amino)hexyl 3,4,5-Trimethoxybenzoate (**45**)

Following the general procedure, compound **45** (0.15 g, yield: 68.7%) was synthesized from **35**(26) (0.17 g, 0.47 mmol) and **51** (0.11 g, 0.39 mmol) in 10.0 mL of dry CH_3_CN. Chromatographic eluent: CH_2_Cl_2_/CH_3_OH/NH_4_OH 93:7:0.7. ^1^H NMR (400 MHz, CDCl_3_) δ: 7.50 (d, *J* = 9.2 Hz, 1H), 7.23 (d, *J* =8.4 Hz, 1H), 7.18 (s, 2H), 6.70 (dd, *J* = 8.4, 2.2 Hz, 1H), 6.66 (d, *J* = 2.2 Hz, 1H), 6.09 (d, *J* = 9.2 Hz, 1H), 4.16 (t, *J* = 6.4 Hz, 2H), 3.94 (t, *J* = 6.4 Hz, 2H), 3.78 (s, 9H), 3.65 (t, *J* = 6.4 Hz, 2H), 2.60–2.44 (m, 4H), 2.35 (t, *J* = 7.2 Hz, 2H), 1.93–1.80 (m, 2H), 1.69–1.52 (m, 4H), 1.45–1.34 (m, 2H), 1.33–1.18 (m, 4H) ppm.

##### 6-((3-Hydroxypropyl)(3-((2-oxo-2*H*-chromen-7-yl)oxy)propyl)amino)hexyl Anthracene-9-carboxylate (**46**)

Following the general procedure, compound **46** (0.13 g, yield: 60.4%) was synthesized from **36**(26) (0.16 g, 0.43 mmol) and **51** (0.10 g, 0.36 mmol) in 10.0 mL of dry CH_3_CN. Chromatographic eluent: CH_2_Cl_2_/CH_3_OH/NH_4_OH 97:3:0.3. ^1^H NMR (400 MHz, CDCl_3_) δ: 8.39 (s, 1H), 7.97 (d, *J* = 8.4 Hz, 2H), 7.91 (d, *J* = 8.4 Hz, 2H), 7.48–7.37 (m, 5H), 7.13 (d, *J* = 9.2 Hz, 1H), 6.66–6.64 (m, 2H), 6.08 (d, *J* = 9.2 Hz, 1H), 4.54 (t, *J* = 6.4 Hz, 2H), 3.87 (t, *J* = 6.4 Hz, 2H), 3.69 (t, *J* = 6.4 Hz, 2H), 2.54–2.47 (m, 4H), 2.34 (t, *J* = 7.2 Hz, 2H), 1.85–1.72 (m, 4H), 1.65–1.53 (m, 2H), 1.49–1.35 (m, 4H), 1.34–1.23 (m, 2H) ppm.

##### (*E*)-7-((3-Hydroxypropyl)(3-((2-oxo-2*H*-chromen-7-yl)oxy)propyl)amino)heptyl 3-(3,4,5-Trimethoxyphenyl)acrylate (**47**)

Following the general procedure, compound **47** (0.16 g, yield: 63.1%) was synthesized from **37**(29) (0.17 g, 0.42 mmol) and **51** (0.14 g, 0.50 mmol) in 7.0 mL of dry CH_3_CN. Chromatographic eluent: CH_2_Cl_2_/CH_3_OH/NH_4_OH 90:10:1. ^1^H NMR (400 MHz, CDCl_3_) δ: 7.57 (d, *J* = 9.6 Hz, 1H), 7.53 (d, *J* = 16.0 Hz, 1H), 7.30 (d, *J* = 8.4 Hz, 1H), 6.79–6.74 (m, 2H), 6.70 (s, 2H), 6.29 (d, *J* = 16.0 Hz, 1H), 6.17 (d, *J* = 9.6 Hz, 1H), 4.12 (t, *J* = 6.4 Hz, 2H), 4.01 (t, *J* = 6.0 Hz, 2H), 3.82 (s, 6H), 3.81 (s, 3H), 3.73 (t, *J* = 5.2 Hz, 2H), 2.66–2.61 (m, 4H), 2.44 (t, *J* = 7.2 Hz, 2H), 2.00–1.93 (m, 2H), 1.70–1.59 (m, 4H), 1.51–1.41 (m, 2H), 1.37–1.19 (m, 7H) ppm.

##### 3-((7-Hydroxyheptyl)(3-((2-oxo-2*H*-chromen-7-yl)oxy)propyl)amino)propyl 3,4,5-Trimethoxybenzoate (**48**)

Following the general procedure, compound **48** (0.16 g, yield: 55.1%) was synthesized from **38** (0.18 g, 0.50 mmol) and **51** (0.16 g, 0.56 mmol) in 7.0 mL of dry CH_3_CN. Chromatographic eluent: CH_2_Cl_2_/CH_3_OH/NH_4_OH 95:5:0.5. ^1^H NMR (400 MHz, CDCl_3_) δ: 7.58 (d, *J* = 9.6 Hz, 1H), 7.31 (d, *J* = 8.4 Hz, 1H), 6.24 (s, 2H), 6.79–6.77 (m, 2H), 6.20 (d, *J* = 9.6 Hz, 1H), 4.31 (t, *J* = 6.4 Hz, 2H), 4.05 (t, *J* = 6.4 Hz, 2H), 3.88 (s, 3H), 3.81 (s, 6H), 3.58 (t, *J* = 6.4 Hz, 2H), 2.61–2.54 (m, 4H), 2.40 (t, *J* = 7.2 Hz, 2H), 1.93–1.87 (m, 4H), 1.69 (bs, 1H), 1.53–1.45 (m, 2H), 1.44–1.35 (m, 2H), 1.33–1.20 (m, 6H) ppm.

##### (*E*)-3-((7-Hydroxyheptyl)(3-((2-oxo-2*H*-chromen-7-yl)oxy)propyl)amino)propyl 3-(3,4,5-Trimethoxyphenyl)acrylate (**49**)

Following the general procedure, compound **49** (0.15 g, yield: 59.1%) was synthesized from **39** (0.17 g, 0.42 mmol) and **51** (0.14 g, 0.50 mmol) in 7.0 mL of dry CH_3_CN. Chromatographic eluent: CH_2_Cl_2_/CH_3_OH/NH_4_OH 95:5:0.5. ^1^H NMR (400 MHz, CDCl_3_) δ: 7.53 (d, *J* = 9.6 Hz, 1H), 7.50 (d, *J* = 16.0 Hz, 1H), 7.27 (d, *J* = 8.4 Hz, 1H), 6.77–6.73 (m, 2H), 6.67 (s, 2H), 6.24 (d, *J* = 16.0 Hz, 1H), 6.13 (d, *J* = 9.6 Hz, 1H), 4.16 (t, *J* = 6.4 Hz, 2H), 4.01 (t, *J* = 6.0 Hz, 2H), 3.81 (s, 6H), 3.80 (s, 3H), 3.53 (t, *J* = 6.4 Hz, 2H), 2.53 (t, *J* = 6.4 Hz, 2H), 2.47 (t, *J* = 6.8 Hz, 2H), 2.34 (t, *J* = 6.8 Hz, 2H), 1.94 (bs, 1H), 1.90–1.82 (m, 2H), 1.80–1.72 (m, 2H), 1.49–1.40 (m, 2H), 1.39–1.30 (m, 2H), 1.29–1.16 (m, 6H) ppm.

##### 3-((7-Hydroxyheptyl)(3-((2-oxo-2*H*-chromen-7-yl)oxy)propyl)amino)propyl Anthracene-9-carboxylate (**50**)

Following the general procedure, compound **50** (0.32 g, yield: 64.0%) was synthesized from **40** (0.33 g, 0.84 mmol) and **51** (0.28 g, 1.01 mmol) in 15.0 mL of dry CH_3_CN. Chromatographic eluent: CH_2_Cl_2_/CH_3_OH/NH_4_OH 97:3:0.3. ^1^H NMR (400 MHz, CDCl_3_) δ: 8.48 (s, 1H), 8.00 (t, *J* = 9.6 Hz, 4H), 7.51–7.41 (m, 5H), 7.21 (d, *J* = 9.2 Hz, 1H), 6.74–6.72 (m, 2H), 6.17 (d, *J* = 9.2 Hz, 1H), 4.64 (t, *J* = 6.4 Hz, 2H), 3.99 (t, *J* = 6.4 Hz, 2H), 3.56 (t, *J* = 6.4 Hz, 2H), 2.62–2.56 (m, 4H), 2.41 (t, *J* = 7.2 Hz, 2H), 2.04–1.96 (m, 2H), 1.94–1.83 (m, 2H), 1.54–1.33 (m, 4H), 1.32–1.16 (m, 6H) ppm.

#### General Procedures for the Synthesis of Diester Compounds **1–27**

Diester compounds were synthesized using two different general procedures:

*Method A:* in an ice bath, to a solution of the suitable ((hydroxyalkyl)alkylcoumarin)aminoester **41–50** (1 equiv) in the adequate amount of dry CH_2_Cl_2_, the proper carboxylic acid (1.5 equiv), DMAP (0.8 equiv), and EDC hydrochloride (1.8 equiv) were added in this order. The reaction mixture was stirred at 0 °C for 1 h and then at rt for 48 h. Then, the residue was treated with CH_2_Cl_2_, and the organic layer was washed twice with water and with a saturated solution of NaHCO_3_, dried over Na_2_SO_4_, and concentrated under reduced pressure. Finally, the residue was purified by flash chromatography using CH_2_Cl_2_/CH_3_OH/NH_4_OH 97:3:0.3 as the proper eluting system, obtaining the desired compound as an oil. The final compounds were transformed into the corresponding hydrochloride as a solid. The salts were crystallized from abs. ethanol/petroleum ether.

*Method B:* the proper carboxylic acid (1.5 equiv) was transformed into the corresponding acyl chloride by treatment with SOCl_2_ (15 equiv) in the adequate amount of CHCl_3_ (free of ethanol) at 60 °C for 4–6 h. Upon completion of the reaction, the mixture was cooled to rt, and the solvent was removed under reduced pressure. The residue was treated twice with cyclohexane, and the solvent was removed under vacuum. The obtained acyl chloride was dissolved in the proper amount of CHCl_3_ (free of ethanol), and the suitable ((hydroxyalkyl)alkylcoumarin)aminoester **41–50** (1 equiv) was added. The mixture was stirred at rt for 18 h; then, the organic layer was washed twice with a saturated solution of NaHCO_3_, dried over Na_2_SO_4_, and concentrated under reduced pressure. Finally, the residue was purified by flash chromatography, using CH_2_Cl_2_/CH_3_OH/NH_4_OH 97:3:0.3 as the proper eluting system, yielding the desired compound as an oil. The final compounds were transformed into the corresponding hydrochloride as a solid. The salts were crystallized from abs. ethanol/petroleum ether.

##### (*E*)-5-((3-((2-Oxo-2*H*-chromen-7-yl)oxy)propyl)(3-(((*E*)-3-(3,4,5-trimethoxyphenyl)acryloyl)oxy)propyl)amino)pentyl 3-(3,4,5-Trimethoxyphenyl)acrylate (**1**)

Following *method A*, compound **1** (0.11 g, yield: 72.8%) was synthesized as a pale yellow oil, starting from **41** (0.11 g, 0.19 mmol) and (*E*)-3-(3,4,5-trimethoxyphenyl)acrylic acid (0.067 g, 0.28 mmol) in 4.0 mL of dry CH_2_Cl_2_. *Free base:* TLC: CH_2_Cl_2_/CH_3_OH/NH_4_OH 95:5:0.5. ^1^H NMR (400 MHz, CDCl_3_) δ: 7.54–7.50 (m, 3H), 7.26 (d, *J* = 9.6 Hz, 1H), 6.76–6.74 (m, 2H), 6.69 (s, 2H), 6.68 (s, 2H), 6.28 (d, *J* = 16.0 Hz, 1H), 6.25 (d, *J* = 16.0 Hz, 1H), 6.13 (d, *J* = 9.6 Hz, 1H), 4.18 (t, *J* = 6.4 Hz, 2H), 4.10 (t, *J* = 6.4 Hz, 2H), 4.02 (t, *J* = 6.4 Hz, 2H), 3.81 (s, 18H), 2.70–2.49 (m, 4H), 2.48–2.35 (m, 2H), 1.97–1.86 (m, 2H), 1.85–1.76 (m, 2H), 1.66–1.59 (m, 2H), 1.52–1.41 (m, 2H), 1.39–1.29 (m, 2H) ppm. ^13^C NMR (100 MHz, CDCl_3_) δ: 166.9 (C), 166.8 (C), 162.1 (C), 161.1 (C), 155.9 (C), 153.4 (C), 144.8 (CH), 144.6 (CH), 143.3 (CH), 140.3 (C), 140.2 (C), 129.9 (C), 129.8 (C), 128.8 (CH), 117.4 (CH), 117.1 (CH), 113.0 (CH), 112.6 (CH), 112.5 (C), 105.3 (CH), 104.5 (C), 101.5 (CH), 66.4 (CH_2_), 64.4 (CH_2_), 62.6 (CH_2_), 60.9 (CH_3_), 56.2 (CH_3_), 53.9 (CH_2_), 50.5 (CH_2_), 50.2 (CH_2_), 28.7 (CH_2_), 23.8 (CH_2_) ppm. ESI-HRMS (*m*/*z*) calculated for [M + H]^+^ ion species C_44_H_54_NO_13_ = 804.3590, found 804.3590. *Hydrochloride:* white solid; mp 81–84 °C.

##### (*E*)-3-((3-((2-Oxo-2*H*-chromen-7-yl)oxy)propyl)(5-((3-(3,4,5-trimethoxyphenyl)acryloyl)oxy)pentyl)amino)propyl 3,4,5-Trimethoxybenzoate (**2**)

Following *method B*, compound **2** (0.058 g, yield: 77.9%) was synthesized as a pale yellow oil, starting from (*E*)-3-(3,4,5-trimethoxyphenyl)acrylic acid (0.034 g, 0.14 mmol) and **42** (0.053 g, 0.096 mmol). *Free base*: TLC: CH_2_Cl_2_/CH_3_OH/NH_4_OH 95:5:0.5. ^1^H NMR (400 MHz, CDCl_3_) δ: 7.52 (d, *J* = 9.6 Hz, 1H), 7.52 (d, *J* = 15.6 Hz, 1H), 7.25 (d, *J* = 8.8 Hz, 1H), 7.20 (s, 2H), 6.75–6.72 (m, 2H), 6.69 (s, 2H), 6.28 (d, *J* = 15.6 Hz, 1H), 6.14 (d, *J* = 9.6 Hz, 1H), 4.28 (t, *J* = 6.4 Hz, 2H), 4.08 (t, *J* = 6.4 Hz, 2H), 4.01 (t, *J* = 6.4 Hz, 2H), 3.83 (s, 3H), 3.82 (s, 6H), 3.81 (s, 6H), 3.81 (s, 3H), 2.62–2.49 (m, 4H), 2.41 (t, *J* = 6.4 Hz, 2H), 1.95–1.82 (m, 4H), 1.63–1.56 (m, 2H), 1.50–1.38 (m, 2H), 1.37–1.30 (m, 2H) ppm. ^13^C NMR (100 MHz, CDCl_3_) δ: 167.0 (C), 166.1 (C), 162.2 (C), 161.1 (C), 155.8 (C), 153.4 (C), 152.9 (C), 144.7 (CH), 143.4 (CH), 142.2 (C), 140.1 (C), 129.9 (C), 128.8 (CH), 125.3 (C), 117.4 (CH), 113.0 (CH), 112.6 (CH), 112.5 (C), 106.8 (CH), 105.2 (CH), 101.4 (CH), 66.4 (CH_2_), 64.4 (CH_2_), 63.3 (CH_2_), 60.9 (CH_3_), 60.9 (CH_3_), 56.2 (CH_3_), 56.1 (CH_3_), 53.9 (CH_2_), 50.4 (CH_2_), 50.1 (CH_2_), 28.7 (CH_2_), 26.8 (CH_2_), 26.4 (CH_2_), 23.8 (CH_2_) ppm. ESI-HRMS (*m*/*z*) calculated for [M + H]^+^ ion species C_42_H_52_NO_13_ = 778.3433, found 778.3435. *Hydrochloride*: white solid; mp 128–131 °C.

##### (*E*)-3-((3-((2-Oxo-2*H*-chromen-7-yl)oxy)propyl)(5-((3-(3,4,5-trimethoxyphenyl)acryloyl)oxy)pentyl)amino)propyl Anthracene-9-carboxylate (**3**)

Following *method B*, compound **3** (0.046 g, yield: 74.0%) was synthesized as a pale yellow oil, starting from (*E*)-3-(3,4,5-trimethoxyphenyl)acrylic acid (0.028 g, 0.12 mmol) and **43** (0.045 g, 0.079 mmol). *Free base:* TLC: CH_2_Cl_2_/CH_3_OH/NH_4_OH 95:5:0.5. ^1^H NMR (400 MHz, CDCl_3_) δ: 8.46 (s, 1H), 7.97 (t, *J* = 7.2 Hz, 4H), 7.54 (d, *J* = 16.0 Hz, 1H), 7.50–7.41 (m, 5H), 7.20 (d, *J* = 8.8 Hz, 1H), 6.73–6.69 (m, 4H), 6.30 (d, *J* = 16.0 Hz, 1H), 6.15 (d, *J* = 9.2 Hz, 1H), 4.62 (t, *J* = 6.4 Hz, 2H), 4.10 (t, *J* = 6.4 Hz, 2H), 3.98 (t, *J* = 6.4 Hz, 2H), 3.83 (s, 3H), 3.80 (s, 6H), 2.62–2.53 (m, 4H), 2.44 (t, *J* = 6.4 Hz, 2H), 2.06–1.95 (m, 2H), 1.94–1.82 (m, 2H), 1.65–1.55 (m, 2H), 1.48–1.40 (m, 2H), 1.39–1.30 (m, 2H) ppm. ^13^C NMR (100 MHz, CDCl_3_) δ: 169.6 (C), 167.0 (C), 162.2 (C), 161.2 (C), 155.8 (C), 153.4 (C), 144.7 (CH), 143.3 (CH), 131.0 (C), 129.9 (C), 129.3 (CH), 128.7 (CH), 128.6 (CH), 128.4 (C), 128.0 (C), 126.9 (CH), 125.5 (CH), 124.9 (CH), 117.4 (CH), 112.9 (CH), 112.6 (CH), 112.4 (C), 105.3 (CH), 101.4 (CH), 66.4 (CH_2_), 64.4 (CH_2_), 64.0 (CH_2_), 60.9 (CH_3_), 56.1 (CH_3_), 54.0 (CH_2_), 50.7 (CH_2_), 50.2 (CH_2_), 28.7 (CH_2_), 26.8 (CH_2_), 23.9 (CH_2_) ppm. ESI-HRMS (*m*/*z*) calculated for [M + H]^+^ ion species C_47_H_50_NO_10_ = 788.3429, found 788.3429. *Hydrochloride:* pale yellow solid; mp 94–97 °C.

##### (*E*)-5-((3-((2-Oxo-2*H*-chromen-7-yl)oxy)propyl)(3-((3-(3,4,5-trimethoxyphenyl)acryloyl)oxy)propyl)amino)pentyl 3,4,5-Trimethoxybenzoate (**4**)

Following *method A*, compound **4** (0.10 g, yield: 83.5%) was synthesized as a pale yellow oil, starting from **41** (0.090 g, 0.15 mmol) and 3,4,5-trimethoxybenzoic acid (0.049 g, 0.23 mmol) in 4.0 mL of dry CH_2_Cl_2_. *Free base:* TLC: CH_2_Cl_2_/CH_3_OH/NH_4_OH 95:5:0.5. ^1^H NMR (400 MHz, CDCl_3_) δ: 7.53 (d, *J* = 16.0 Hz, 1H), 7.53 (d, *J* = 9.6 Hz, 1H), 7.28 (d, *J* = 9.2 Hz, 1H), 7.23 (s, 2H), 6.78–6.75 (m, 2H), 6.69 (s, 2H), 6.27 (d, *J* = 16.0 Hz, 1H), 6.15 (d, *J* = 9.6 Hz, 1H), 4.23 (t, *J* = 6.4 Hz, 2H), 4.19 (t, *J* = 6.4 Hz, 2H), 4.03 (t, *J* = 6.4 Hz, 2H), 3.85 (s, 9H), 3.83 (s, 9H), 2.72–2.49 (m, 4H), 2.48–2.35 (m, 2H), 2.01–1.87 (m, 2H), 1.86–1.77 (m, 2H), 1.76–1.67 (m, 2H), 1.58–1.45 (m, 2H), 1.44–1.31 (m, 2H) ppm. ^13^C NMR (100 MHz, CDCl_3_) δ: 166.9 (C), 166.2 (C), 162.1 (C), 161.1 (C), 155.9 (C), 153.4 (C), 152.9 (C), 144.8 (CH), 143.3 (CH), 142.2 (C), 140.2 (C), 129.8 (C), 128.8 (CH), 125.4 (C), 117.1 (CH), 113.0 (CH), 112.6 (CH), 112.5 (C), 106.9 (CH), 105.3 (CH), 101.5 (CH), 66.4 (CH_2_), 65.0 (CH_2_), 62.7 (CH_2_), 60.9 (CH_3_), 60.9 (CH_3_), 56.3 (CH_3_), 56.2 (CH_3_), 53.9 (CH_2_), 50.5 (CH_2_), 50.2 (CH_2_), 28.7 (CH_2_), 26.6 (CH_2_), 23.8 (CH_2_) ppm. ESI-HRMS (*m*/*z*) calculated for [M + H]^+^ ion species C_42_H_52_NO_13_ = 778.3433, found 778.3434. *Hydrochloride*: white solid; mp 74–77 °C.

##### 5-((3-((2-Oxo-2*H*-chromen-7-yl)oxy)propyl)(3-((3,4,5-trimethoxybenzoyl)oxy)propyl)amino)pentyl 3,4,5-Trimethoxybenzoate (**5**)

Following *method A*, compound **5** (0.055 g, yield: 74.2%) was synthesized as a pale yellow oil, starting from **42** (0.054 g, 0.098 mmol) and 3,4,5-trimethoxybenzoic acid (0.031 g, 0.15 mmol) in 5.0 mL of dry CH_2_Cl_2_. *Free base:* TLC: CH_2_Cl_2_/CH_3_OH/NH_4_OH 95:5:0.5. ^1^H NMR (400 MHz, CDCl_3_) δ: 7.52 (d, *J* = 9.6 Hz, 1H), 7.26 (d, *J* = 8.4 Hz, 1H), 7.22 (s, 2H), 7.20 (s, 2H), 6.75–6.72 (m, 2H), 6.14 (d, *J* = 9.6 Hz, 1H), 4.28 (t, *J* = 6.4 Hz, 2H), 4.21 (t, *J* = 6.4 Hz, 2H), 4.01 (t, *J* = 6.4 Hz, 2H), 3.84 (s, 6H), 3.84 (s, 6H), 3.83 (s, 6H), 2.70–2.51 (m, 4H), 2.50–2.35 (m, 2H), 1.97–1.80 (m, 4H), 1.72–1.64 (m, 2H), 1.56–1.42 (m, 2H), 1.41–1.32 (m, 2H) ppm. ^13^C NMR (100 MHz, CDCl_3_) δ: 166.2 (C), 166.1 (C), 162.1 (C), 161.1 (C), 155.8 (C), 152.9 (C), 143.3 (CH), 142.2 (C), 128.8 (CH), 125.4 (C), 125.2 (C), 113.0 (CH), 112.6 (CH), 112.5 (C), 106.8 (CH), 106.8 (CH), 101.4 (CH), 66.4 (CH_2_), 65.0 (CH_2_), 63.3 (CH_2_), 60.9 (CH_3_), 56.2 (CH_3_), 54.0 (CH_2_), 50.4 (CH_2_), 50.2 (CH_2_), 28.7 (CH_2_), 26.8 (CH_2_), 23.8 (CH_2_) ppm. ESI-HRMS (*m*/*z*) calculated for [M + H]^+^ ion species C_40_H_50_NO_13_ = 752.3277, found 752.3276. *Hydrochloride*: white solid; mp 84–87 °C.

##### 3-((3-((2-Oxo-2*H*-chromen-7-yl)oxy)propyl)(5-((3,4,5-trimethoxybenzoyl)oxy)pentyl)amino)propyl Anthracene-9-carboxylate (**6**)

Following *method A*, compound **6** (0.044 g, yield: 59.3%) was synthesized as a pale yellow oil, starting from **43** (0.054 g, 0.095 mmol) and 3,4,5-trimethoxybenzoic acid (0.030 g, 0.14 mmol) in 5.0 mL of dry CH_2_Cl_2_. *Free base:* TLC: CH_2_Cl_2_/CH_3_OH/NH_4_OH 95:5:0.5. ^1^H NMR (400 MHz, CDCl_3_) δ: 8.45 (s, 1H), 7.97 (t, *J* = 8.0 Hz, 4H), 7.50–7.40 (m, 5H), 7.24 (s, 2H), 7.19 (d, *J* = 8.8 Hz, 1H), 6.73–6.70 (m, 2H), 6.14 (d, *J* = 9.2 Hz, 1H), 4.62 (t, *J* = 6.4 Hz, 2H), 4.20 (t, *J* = 6.4 Hz, 2H), 3.97 (t, *J* = 6.4 Hz, 2H), 3.86 (s, 3H), 3.84 (s, 6H), 2.62–2.55 (m, 4H), 2.43 (t, *J* = 6.4 Hz, 2H), 2.02–1.94 (m, 2H), 1.90–1.83 (m, 2H), 1.70–1.63 (m, 2H), 1.49–1.43 (m, 2H), 1.42–1.33 (m, 2H) ppm. ^13^C NMR (100 MHz, CDCl_3_) δ: 169.6 (C), 166.2 (C), 162.2 (C), 161.1 (C), 155.8 (C), 152.9 (C), 143.3 (CH), 142.3 (C), 131.0 (C), 129.3 (CH), 128.7 (CH), 128.6 (CH), 128.4 (C), 128.0 (C), 126.9 (CH), 125.5 (CH), 124.9 (CH), 112.9 (CH), 112.7 (CH), 112.4 (C), 106.9 (CH), 101.4 (CH), 66.5 (CH_2_), 65.0 (CH_2_), 64.1 (CH_2_), 60.9 (CH_3_), 56.3 (CH_3_), 54.1 (CH_2_), 50.7 (CH_2_), 50.3 (CH_2_), 28.7 (CH_2_), 26.9 (CH_2_), 26.8 (CH_2_), 23.9 (CH_2_) ppm. ESI-HRMS (*m*/*z*) calculated for [M + H]^+^ ion species C_45_H_48_NO_10_ = 762.3273, found 762.3270. *Hydrochloride*: yellow solid; mp 114–117 °C.

##### (*E*)-5-((3-((2-Oxo-2*H*-chromen-7-yl)oxy)propyl)(3-((3-(3,4,5-trimethoxyphenyl)acryloyl)oxy)propyl)amino)pentyl Anthracene-9-carboxylate (**7**)

Following *method B*, compound **7** (0.020 g, yield: 18.5%) was synthesized as a pale yellow oil, starting from anthracene-9-carboxylic acid (0.046 g, 0.21 mmol) and **41** (0.080 g, 0.14 mmol). *Free base:* TLC: CH_2_Cl_2_/CH_3_OH/NH_4_OH 95:5:0.5. ^1^H NMR (400 MHz, CDCl_3_) δ: 8.47 (s, 1H), 7.97 (d, *J* = 9.2 Hz, 4H), 7.54 (d, *J* = 16.0 Hz, 1H), 7.51–7.42 (m, 5H), 7.24 (d, *J* = 9.2 Hz, 1H), 6.74–6.71 (m, 2H), 6.69 (s, 2H), 6.26 (d, *J* = 16.0 Hz, 1H), 6.15 (d, *J* = 9.2 Hz, 1H), 4.56 (t, *J* = 6.4 Hz, 2H), 4.18 (t, *J* = 6.4 Hz, 2H), 3.98 (t, *J* = 6.4 Hz, 2H), 3.83 (s, 3H), 3.82 (s, 6H), 2.80–2.34 (m, 6H), 1.91–1.78 (m, 4H), 1.66–1.42 (m, 6H) ppm. ^13^C NMR (100 MHz, CDCl_3_) δ: 160.9 (C), 155.7 (C), 153.5 (C), 146.0 (CH), 143.2 (CH), 130.9 (C), 129.5 (CH), 129.0 (CH), 128.7(CH), 128.3 (C), 127.2 (CH), 125.6 (CH), 124.8 (CH), 116.1 (CH), 113.6 (CH), 113.1 (C), 112.2 (CH), 105.4 (CH), 101.7 (CH), 65.2 (CH_2_), 61.0 (CH_2_), 61.0 (CH_3_), 56.2 (CH_3_), 52.7 (CH_2_), 50.3 (CH_2_), 28.1 (CH_2_), 23.7 (CH_2_), 23.5 (CH_2_), 23.0 (CH_2_), 22.9 (CH_2_) ppm. ESI-HRMS (*m*/*z*) calculated for [M + H]^+^ ion species C_47_H_50_NO_10_ = 788.3429, found 788.3430. *Hydrochloride*: yellow solid; mp 92–95 °C.

##### 5-((3-((2-Oxo-2*H*-chromen-7-yl)oxy)propyl)(3-((3,4,5-trimethoxybenzoyl)oxy)propyl)amino)pentyl Anthracene-9-carboxylate (**8**)

Following *method B*, compound **8** (0.063 g, yield: 79.7%) was synthesized as a yellow oil, starting from anthracene-9-carboxylic acid (0.034 g, 0.15 mmol) and **42** (0.058 g, 0.10 mmol). *Free base:* TLC: CH_2_Cl_2_/CH_3_OH/NH_4_OH 95:5:0.5. ^1^H NMR (400 MHz, CDCl_3_) δ: 8.46 (s, 1H), 7.98–7.95 (m, 4H), 7.51–7.41 (m, 5H), 7.22 (s, 2H), 7.20 (d, *J* = 8.8 Hz, 1H), 6.71–6.68 (m, 2H), 6.13 (d, *J* = 9.2 Hz, 1H), 4.54 (t, *J* = 6.4 Hz, 2H), 4.27 (t, *J* = 6.4 Hz, 2H), 3.94 (t, *J* = 6.4 Hz, 2H), 3.85 (s, 3H), 3.83 (s, 6H), 2.61–2.49 (m, 4H), 2.48–2.38 (m, 2H), 1.90–1.76 (m, 6H), 1.57–1.40 (m, 4H) ppm. ^13^C NMR (100 MHz, CDCl_3_) δ: 169.7 (C), 166.1 (C), 162.1 (C), 161.2 (C), 155.8 (C), 152.9 (C), 143.3 (CH), 131.0 (C), 129.3 (CH), 128.7 (CH), 128.6 (CH), 128.3 (C), 128.1 (C), 127.0 (CH), 125.5 (CH), 125.2 (C), 124.9 (CH), 113.0 (CH), 112.6 (CH), 112.5 (C), 106.8 (CH), 101.3 (CH), 66.3 (CH_2_), 65.7 (CH_2_), 63.2 (CH_2_), 60.9 (CH_3_), 56.2 (CH_3_), 53.9 (CH_2_), 50.4 (CH_2_), 50.1 (CH_2_), 28.6 (CH_2_), 24.0 (CH_2_) ppm. ESI-HRMS (*m*/*z*) calculated for [M + H]^+^ ion species C_45_H_48_NO_10_ = 762.3273, found 762.3270. *Hydrochloride*: pale yellow solid; mp 120–123 °C.

##### 3-((5-((Anthracene-9-carbonyl)oxy)pentyl)(3-((2-oxo-2*H*-chromen-7-yl)oxy)propyl)amino)propyl Anthracene-9-carboxylate (**9**)

Following *method B*, compound **9** (0.045 g, yield: 66.8%) was synthesized as a yellow oil, starting from anthracene-9-carboxylic acid (0.029 g, 0.13 mmol) and **43** (0.049 g, 0.086 mmol). *Free base:* TLC: CH_2_Cl_2_/CH_3_OH/NH_4_OH 95:5:0.5. ^1^H NMR (400 MHz, CDCl_3_) δ: 8.46 (s, 2H), 8.00–7.95 (m, 8H), 7.51–7.40 (m, 9H), 7.12 (d, *J* = 8.8 Hz, 1H), 6.68–6.65 (m, 2H), 6.12 (d, *J* = 9.2 Hz, 1H), 4.60 (t, *J* = 6.4 Hz, 2H), 4.52 (t, *J* = 6.4 Hz, 2H), 3.91 (t, *J* = 6.4 Hz, 2H), 2.59–2.53 (m, 4H), 2.42 (t, *J* = 6.4 Hz, 2H), 2.01–1.92 (m, 2H), 1.86–1.73 (m, 4H), 1.55–1.40 (m, 4H) ppm. ^13^C NMR (100 MHz, CDCl_3_) δ: 169.7 (C), 162.1 (C), 161.2 (C), 155.8 (C), 143.3 (CH), 142.6 (C), 141.9 (C), 131.0 (C), 129.3 (CH), 129.2 (CH), 128.6 (CH), 128.4 (C), 128.0 (C), 126.9 (CH), 125.5 (CH), 125.0 (CH), 124.9 (CH), 112.9 (CH), 112.6 (CH), 112.4 (C), 101.3 (CH), 66.4 (CH_2_), 65.7 (CH_2_), 64.0 (CH_2_), 53.9 (CH_2_), 50.6 (CH_2_), 50.1 (CH_2_), 28.7 (CH_2_), 26.7 (CH_2_), 26.6 (CH_2_), 24.0 (CH_2_) ppm. ESI-HRMS (*m*/*z*) calculated for [M + H]^+^ ion species C_50_H_46_NO_7_= 772.3269, found 772.3267. *Hydrochloride*: yellow solid; mp 118–121 °C.

##### (*E*)-6-((3-((2-Oxo-2*H*-chromen-7-yl)oxy)propyl)(3-(((*E*)-3-(3,4,5-trimethoxyphenyl)acryloyl)oxy)propyl)amino)hexyl 3-(3,4,5-Trimethoxyphenyl)acrylate (**10**)

Following *method A*, compound **10** (0.082 g, yield: 100.0%) was synthesized as a pale yellow oil, starting from **44** (0.060 g, 0.10 mmol) and (*E*)-3-(3,4,5-trimethoxyphenyl)acrylic acid (0.036 g, 0.15 mmol) in 4.0 mL of dry CH_2_Cl_2_. *Free base:* TLC: CH_2_Cl_2_/CH_3_OH/NH_4_OH 95:5:0.5. ^1^H NMR (400 MHz, CDCl_3_) δ: 7.55–7.51 (m, 3H), 7.28 (d, *J* = 9.2 Hz, 1H), 6.80–6.76 (m, 2H), 6.71 (s, 2H), 6.70 (s, 2H), 6.30 (d, *J* = 16.0 Hz, 1H), 6.27 (d, *J* = 16.0 Hz, 1H), 6.16 (d, *J* = 9.2 Hz, 1H), 4.19 (t, *J* = 6.4 Hz, 2H), 4.11 (t, *J* = 6.4 Hz, 2H), 4.04 (t, *J* = 6.4 Hz, 2H), 3.84 (s, 9H), 3.84 (s, 9H), 2.56 (t, *J* = 6.4 Hz, 2H), 2.50 (t, *J* = 6.4 Hz, 2H), 2.39 (t, *J* = 6.4 Hz, 2H), 1.93–1.86 (m, 2H), 1.82–1.77 (m, 2H), 1.64–1.57 (m, 2H), 1.43–1.29 (m, 6H) ppm. ^13^C NMR (100 MHz, CDCl_3_) δ: 167.0 (C), 162.3 (C), 155.9 (C), 153.4 (C), 144.7 (CH), 144.6 (CH), 143.4 (CH), 140.1 (C), 129.9 (C), 129.9 (C), 128.7 (CH), 117.4 (CH), 117.2 (CH), 113.0 (CH), 112.8 (CH), 112.4 (C), 105.2 (CH), 101.4 (CH), 66.5 (CH_2_), 64.6 (CH_2_), 62.9 (CH_2_), 61.0 (CH_3_), 56.2 (CH_3_), 54.1 (CH_2_), 50.5 (CH_2_), 50.2 (CH_2_), 28.7 (CH_2_), 27.3 (CH_2_), 27.2 (CH_2_), 27.0 (CH_2_), 26.7 (CH_2_), 25.9 (CH_2_) ppm. ESI-HRMS (*m*/*z*) calculated for [M + H]^+^ ion species C_45_H_56_NO_13_ = 818.3746, found 818.3748. *Hydrochloride*: white solid; mp 87–90 °C.

##### (*E*)-3-((3-((2-Oxo-2*H*-chromen-7-yl)oxy)propyl)(6-((3-(3,4,5-trimethoxyphenyl)acryloyl)oxy)hexyl)amino)propyl 3,4,5-Trimethoxybenzoate (**11**)

Following *method B*, compound **11** (0.043 g, yield: 40.6%) was synthesized as a pale yellow oil, starting from 3,4,5-trimethoxybenzoic acid (0.043 g, 0.20 mmol) and **44** (0.080 g, 0.13 mmol). *Free base:* TLC: CH_2_Cl_2_/CH_3_OH/NH_4_OH 95:5:0.5. ^1^H NMR (400 MHz, CDCl_3_) δ: 7.55 (d, *J* = 9.6 Hz, 1H), 7.54 (d, *J* = 16.0 Hz, 1H), 7.28 (d, *J* = 9.2 Hz, 1H), 7.22 (s, 2H), 6.78–6.75 (m, 2H), 6.71 (s, 2H), 6.30 (d, *J* = 16.0 Hz, 1H), 6.17 (d, *J* = 9.6 Hz, 1H), 4.30 (t, *J* = 6.4 Hz, 2H), 4.10 (t, *J* = 6.4 Hz, 2H), 4.03 (t, *J* = 6.4 Hz, 2H), 3.86 (s, 3H), 3.85 (s, 6H), 3.84 (s, 6H), 3.83 (s, 3H), 2.59–2.52 (m, 4H), 2.40 (t, *J* = 6.4 Hz, 2H), 1.91–1.84 (m, 4H), 1.64–1.56 (m, 2H), 1.44–1.36 (m, 2H), 1.35–1.25 (m, 4H) ppm. ^13^C NMR (100 MHz, CDCl_3_) δ: 167.0 (C), 166.1 (C), 162.3 (C), 161.2 (C), 155.9 (C), 153.4 (C), 152.9 (C), 144.6 (CH), 143.4 (CH), 142.2 (C), 140.1 (C), 129.9 (C), 128.7 (CH), 125.3 (C), 117.4 (CH), 113.0 (CH), 112.8 (CH), 112.4 (C), 106.7 (CH), 105.2 (CH), 101.3 (CH), 66.5 (CH_2_), 64.5 (CH_2_), 63.4 (CH_2_), 61.0 (CH_3_), 60.9 (CH_3_), 56.2 (CH_3_), 56.2 (CH_3_), 54.1 (CH_2_), 50.4 (CH_2_), 50.1 (CH_2_), 28.7 (CH_2_), 27.2 (CH_2_), 27.2 (CH_2_), 27.0 (CH_2_), 26.7 (CH_2_), 25.9 (CH_2_) ppm. ESI-HRMS (*m*/*z*) calculated for [M + H]^+^ ion species C_43_H_54_NO_13_ = 792.3590, found 792.3589. *Hydrochloride*: white solid; mp 71–74 °C.

##### (*E*)-3-((3-((2-Oxo-2*H*-chromen-7-yl)oxy)propyl)(6-((3-(3,4,5-trimethoxyphenyl)acryloyl)oxy)hexyl)amino)propyl Anthracene-9-carboxylate (**12**)

Following *method B*, compound **12** (0.068 g, yield: 56.5%) was synthesized as a pale yellow oil, starting from anthracene-9-carboxylic acid (0.050 g, 0.23 mmol) and **44** (0.090 g, 0.15 mmol). *Free base:* TLC: CH_2_Cl_2_/CH_3_OH/NH_4_OH 95:5:0.5. ^1^H NMR (400 MHz, CDCl_3_) δ: 8.47 (s, 1H), 8.01–7.94 (m, 4H), 7.55 (d, *J* = 16.0 Hz, 1H), 7.52–7.43 (m, 5H), 7.21 (d, *J* = 9.2 Hz, 1H), 6.74–6.72 (m, 2H), 6.71 (s, 2H), 6.30 (d, *J* = 16.0 Hz, 1H), 6.16 (d, *J* = 9.6 Hz, 1H), 4.62 (t, *J* = 6.4 Hz, 2H), 4.10 (t, *J* = 6.4 Hz, 2H), 3.99 (t, *J* = 6.4 Hz, 2H), 3.84 (s, 3H), 3.83 (s, 6H), 2.60–2.55 (m, 4H), 2.40 (t, *J* = 7.2 Hz, 2H), 2.01–1.95 (m, 2H), 1.89.1.83 (m, 2H), 1.62–1.55 (m, 2H), 1.45–1.36 (m, 2H), 1.34.1.25 (m, 4H) ppm. ^13^C NMR (100 MHz, CDCl_3_) δ: 169.6 (C), 167.0 (C), 162.2 (C), 161.2 (C), 155.8 (C), 153.4 (C), 144.6 (CH), 143.4 (CH), 140.1 (C), 131.0 (C), 129.9 (C), 129.3 (CH), 128.7 (CH), 128.4 (C), 128.0 (C), 127.0 (CH), 125.5 (CH), 124.9 (CH), 117.4 (CH), 112.9 (CH), 112.8 (CH), 112.4 (C), 105.2 (CH), 101.3 (CH), 66.4 (CH_2_), 64.5 (CH_2_), 64.1 (CH_2_), 61.0 (CH_3_), 56.1 (CH_3_), 54.1 (CH_2_), 50.6 (CH_2_), 50.2 (CH_2_), 28.7 (CH_2_), 27.2 (CH_2_), 26.9 (CH_2_), 26.7 (CH_2_), 25.9 (CH_2_) ppm. ESI-HRMS (*m*/*z*) calculated for [M + H]^+^ ion species C_48_H_52_NO_10_ = 802.3586, found 802.3591. *Hydrochloride*: pale yellow solid; mp 99–102 °C.

##### (*E*)-6-((3-((2-Oxo-2*H*-chromen-7-yl)oxy)propyl)(3-((3-(3,4,5-trimethoxyphenyl)acryloyl)oxy)propyl)amino)hexyl 3,4,5-Trimethoxybenzoate (**13**)

Following *method B*, compound **13** (0.096 g, yield: 97.7%) was synthesized as a pale yellow oil, starting from (*E*)-3-(3,4,5-trimethoxyphenyl)acrylic acid (0.044 g, 0.19 mmol) and **45** (0.071 g, 0.12 mmol). *Free base:* TLC: CH_2_Cl_2_/CH_3_OH/NH_4_OH 95:5:0.5. ^1^H NMR (400 MHz, CDCl_3_) δ: 7.51 (d, *J* = 9.6 Hz, 1H), 7.50 (d, *J* =15.6 Hz, 1H), 7.25 (d, *J* = 8.8 Hz, 1H), 7.21 (s, 2H), 6.75–6.72 (m, 2H), 6.66 (s, 2H), 6.24 (d, *J* =15.6 Hz, 1H), 6.11 (d, *J* = 9.6 Hz, 1H), 4.20–4.14 (m, 4H), 4.00 (t, *J* = 6.4 Hz, 2H), 3.82 (s, 9H), 3.80 (s, 6H), 3.80 (s, 3H), 2.53 (t, *J* = 6.4 Hz, 2H), 2.48 (t, *J* = 6.4 Hz, 2H), 2.36 (t, *J* = 6.4 Hz, 2H), 1.90–1.82 (m, 2H), 1.81–1.72 (m, 2H), 1.71–1.62 (m, 2H), 1.45–1.23 (m, 6H) ppm. ^13^C NMR (100 MHz, CDCl_3_) δ: 166.9 (C), 166.2 (C), 162.3 (C), 161.1 (C), 155.9 (C), 153.4 (C), 152.9 (C), 144.7 (CH), 143.4 (CH), 142.1 (C), 140.1 (C), 129.8 (C), 128.7 (CH), 125.5 (C), 117.2 (CH), 112.9 (CH), 112.7 (CH), 112.4 (C), 106.8 (CH), 105.2 (CH), 101.3 (CH), 66.5 (CH_2_), 65.1 (CH_2_), 62.8 (CH_2_), 60.9 (CH_3_), 60.9 (CH_3_), 56.2 (CH_3_), 56.1 (CH_3_), 54.1 (CH_2_), 50.5 (CH_2_), 50.1 (CH_2_), 28.7 (CH_2_), 27.1 (CH_2_), 26.9 (CH_2_), 26.6 (CH_2_), 25.9 (CH_2_) ppm. ESI-HRMS (*m*/*z*) calculated for [M + H]^+^ ion species C_43_H_54_NO_13_ = 792.3590, found 792.3590. *Hydrochloride*: white solid; mp 78–81 °C.

##### 6-((3-((2-Oxo-2*H*-chromen-7-yl)oxy)propyl)(3-((3,4,5-trimethoxybenzoyl)oxy)propyl)amino)hexyl 3,4,5-Trimethoxybenzoate (**14**)

Following *method A*, compound **14** (0.089 g, yield: 91.7%) was synthesized as a pale yellow oil, starting from **45** (0.072 g, 0.13 mmol) and 3,4,5-trimethoxybenzoic acid (0.040 g, 0.19 mmol) in 5.0 mL of dry CH_2_Cl_2_. *Free base:* TLC: CH_2_Cl_2_/CH_3_OH/NH_4_OH 95:5:0.5. ^1^H NMR (400 MHz, CDCl_3_) δ: 7.52 (d, *J* = 9.6 Hz, 1H), 7.25 (d, *J* = 8.8 Hz, 1H), 7.22 (s, 2H), 7.20 (s, 2H), 6.75–6.72 (m, 2H), 6.14 (d, *J* = 9.6 Hz, 1H), 4.27 (t, *J* = 6.4 Hz, 2H), 4.20 (t, *J* = 6.4 Hz, 2H), 4.00 (t, *J* = 6.4 Hz, 2H), 3.84 (s, 9H), 3.83 (s, 9H), 2.58–2.49 (m, 4H), 2.38 (t, *J* = 6.4 Hz, 2H), 1.89–1.81 (m, 4H), 1.69–1.62 (m, 2H), 1.45–1.23 (m, 6H) ppm. ^13^C NMR (100 MHz, CDCl_3_) δ: 166.2 (C), 166.1 (C), 162.2 (C), 161.1 (C), 155.9 (C), 152.9 (C), 143.3 (CH), 142.2 (C), 128.7 (CH), 125.5 (C), 125.3 (C), 112.9 (CH), 112.7 (CH), 112.4 (C), 106.8 (CH), 106.8 (CH), 101.3 (CH), 66.4 (CH_2_), 65.1 (CH_2_), 63.4 (CH_2_), 60.9 (CH_3_), 56.2 (CH_3_), 56.2 (CH_3_), 54.1 (CH_2_), 50.4 (CH_2_), 50.1 (CH_2_), 28.7 (CH_2_), 27.1 (CH_2_), 26.9 (CH_2_), 26.6 (CH_2_), 25.9 (CH_2_) ppm. ESI-HRMS (*m*/*z*) calculated for [M + H]^+^ ion species C_41_H_52_NO_13_ = 766.3433, found 766.3430. *Hydrochloride*: pale yellow solid; mp 83–85 °C.

##### 3-((3-((2-Oxo-2*H*-chromen-7-yl)oxy)propyl)(6-((3,4,5-trimethoxybenzoyl)oxy)hexyl)amino)propyl Anthracene-9-carboxylate (**15**)

Following *method B*, compound **15** (0.016 g, yield: 14.2%) was synthesized as a yellow oil, starting from anthracene-9-carboxylic acid (0.049 g, 0.21 mmol) and **45** (0.084 g, 0.15 mmol). *Free base:* TLC: CH_2_Cl_2_/CH_3_OH/NH_4_OH 95:5:0.5. ^1^H NMR (400 MHz, CDCl_3_) δ: 8.47 (s, 1H), 7.99–7.96 (m, 4H), 7.52–7.42 (m, 5H), 7.24 (s, 2H), 7.21 (d, *J* = 8.8 Hz, 1H), 6.73–6.71 (m, 2H), 6.16 (d, *J* = 9.2 Hz, 1H), 4.62 (t, *J* = 6.4 Hz, 2H), 4.20 (t, *J* = 6.4 Hz, 2H), 3.98 (t, *J* = 6.4 Hz, 2H), 3.86 (s, 3H), 3.85 (s, 6H), 2.71–2.57 (m, 4H), 2.52–2.40 (m, 2H), 2.10–1.95 (m, 2H), 1.94–1.85 (m, 2H), 1.71–1.62 (m, 2H), 1.50–1.39 (m, 2H), 1.37–1.25 (m, 4H) ppm. ^13^C NMR (100 MHz, CDCl_3_) δ: 166.2 (C), 162.1 (C), 161.1 (C), 152.9 (C), 143.3 (CH), 142.2 (C), 131.0 (C), 129.3 (CH), 128.7 (CH), 128.4 (C), 127.0 (CH), 125.5 (CH), 124.9 (CH), 113.0 (CH), 112.7 (CH), 112.5 (C), 106. 9 (CH), 101.4 (CH), 66.4 (CH_2_), 65.1 (CH_2_), 64.0 (CH_2_), 60.9 (CH_3_), 56.3 (CH_3_), 54.1 (CH_2_), 50.6 (CH_2_), 50.3 (CH_2_), 28.7 (CH_2_), 27.1 (CH_2_), 25.9 (CH_2_) ppm. ESI-HRMS (*m*/*z*) calculated for [M + H]^+^ ion species C_46_H_50_NO_10_ = 776.3429, found 776.3435. *Hydrochloride*: yellow solid; mp 148–151 °C.

##### (*E*)-6-((3-((2-Oxo-2*H*-chromen-7-yl)oxy)propyl)(3-((3-(3,4,5-trimethoxyphenyl)acryloyl)oxy)propyl)amino)hexyl Anthracene-9-carboxylate (**16**)

Following *method B*, compound **16** (0.060 g, yield: 100.0%) was synthesized as a yellow oil, starting from (*E*)-3-(3,4,5-trimethoxyphenyl)acrylic acid (0.027 g, 0.11 mmol) and **46** (0.043 g, 0.074 mmol). *Free base:* TLC: CH_2_Cl_2_/CH_3_OH/NH_4_OH 95:5:0.5. ^1^H NMR (400 MHz, CDCl_3_) δ: 8.44 (s, 1H), 7.98 (d, *J* = 8.4 Hz, 2H), 7.95 (d, *J* = 8.4 Hz, 2H), 7.54 (d, *J* = 16.0 Hz, 1H), 7.50–7.40 (m, 5H), 7.17 (d, *J* = 8.8 Hz, 1H), 6.71–6.69 (m, 2H), 6.68 (s, 2H), 6.27 (d, *J* = 16.0 Hz, 1H), 6.09 (d, *J* = 9.2 Hz, 1H), 4.54 (t, *J* = 6.4 Hz, 2H), 4.18 (t, *J* = 6.4 Hz, 2H), 3.96 (t, *J* = 6.4 Hz, 2H), 3.83 (s, 3H), 3.80 (s, 6H), 2.54 (t, *J* = 6.4 Hz, 2H), 2.49 (t, *J* = 6.4 Hz, 2H), 2.38 (t, *J* = 6.4 Hz, 2H), 1.86–1.75 (m, 6H), 1.49–1.27 (m, 6H) ppm. ^13^C NMR (100 MHz, CDCl_3_) δ: 169.6 (C), 166.9 (C), 162.2 (C), 161.1 (C), 155.8 (C), 153.4 (C), 144.7 (CH), 143.3 (CH), 141.9 (C), 140.2 (C), 131.0 (C), 129.9 (C), 129.2 (CH), 128.7 (CH), 128.6 (CH), 128.4 (C), 128.2 (C), 126.9 (CH), 125.5 (CH), 125.0 (CH), 117.2 (CH), 112.9 (CH), 112.7 (CH), 112.4 (C), 105.3 (CH), 101.4 (CH), 66.5 (CH_2_), 65.8 (CH_2_), 62.8 (CH_2_), 60.9 (CH_3_), 56.2 (CH_3_), 54.0 (CH_2_), 50.5 (CH_2_), 50.2 (CH_2_), 28.7 (CH_2_), 27.1 (CH_2_), 26.9 (CH_2_), 26.6 (CH_2_), 26.1 (CH_2_) ppm. ESI-HRMS (*m*/*z*) calculated for [M + H]^+^ ion species C_48_H_52_NO_10_ = 802.3586, found 802.3590. *Hydrochloride*: pale yellow solid; mp 92–95 °C.

##### 6-((3-((2-Oxo-2*H*-chromen-7-yl)oxy)propyl)(3-((3,4,5-trimethoxybenzoyl)oxy)propyl)amino)hexyl Anthracene-9-carboxylate (**17**)

Following *method A*, compound **17** (0.076 g, yield: 77.7%) was synthesized as a yellow oil, starting from **46** (0.074 g, 0.13 mmol) and 3,4,5-trimethoxybenzoic acid (0.040 g, 0.19 mmol) in 5.0 mL of dry CH_2_Cl_2_. *Free base:* TLC: CH_2_Cl_2_/CH_3_OH/NH_4_OH 95:5:0.5. ^1^H NMR (400 MHz, CDCl_3_) δ: 8.44 (s, 1H), 7.98 (d, *J* = 8.4 Hz, 2H), 7.95 (d, *J* = 8.4 Hz, 2H), 7.51–7.40 (m, 5H), 7.22 (s, 2H), 7.16 (d, *J* = 8.8 Hz, 1H), 6.70–6.67 (m, 2H), 6.11 (d, *J* = 9.2 Hz, 1H), 4.54 (t, *J* = 6.4 Hz, 2H), 4.27 (t, *J* = 6.4 Hz, 2H), 3.95 (t, *J* = 6.4 Hz, 2H), 3.85 (s, 3H), 3.83 (s, 6H), 2.56–2.48 (m, 4H), 2.37 (t, *J* = 6.4 Hz, 2H), 1.89–1.74 (m, 6H), 1.48–1.28 (m, 6H) ppm. ^13^C NMR (100 MHz, CDCl_3_) δ: 169.7 (C), 166.1 (C), 162.2 (C), 161.1 (C), 155.8 (C), 152.9 (C), 143.3 (CH), 142.3 (C), 131.0 (C), 129.2 (CH), 128.7 (CH), 128.6 (CH), 128.4 (C), 128.2 (C), 126.9 (CH), 125.5 (CH), 125.3 (C), 125.0 (CH), 112.9 (CH), 112.7 (CH), 112.4 (C), 106.8 (CH), 101.3 (CH), 66.4 (CH_2_), 65.8 (CH_2_), 63.4 (CH_2_), 60.9 (CH_3_), 56.2 (CH_3_), 54.0 (CH_2_), 50.4 (CH_2_), 50.1 (CH_2_), 28.7 (CH_2_), 27.1 (CH_2_), 26.9 (CH_2_), 26.6 (CH_2_), 26.1 (CH_2_) ppm. ESI-HRMS (*m*/*z*) calculated for [M + H]^+^ ion species C_46_H_50_NO_10_ = 776.3429, found 776.3425. *Hydrochloride*: yellow solid; mp 87–90 °C.

##### 3-((6-((Anthracene-9-carbonyl)oxy)hexyl)(3-((2-oxo-2*H*-chromen-7-yl)oxy)propyl)amino)propyl Anthracene-9-carboxylate (**18**)

Following *method B*, compound **18** (0.030 g, yield: 44.3%) was synthesized as a pale yellow oil, starting from anthracene-9-carboxylic acid (0.029 g, 0.13 mmol) and **46** (0.050 g, 0.086 mmol). *Free base:* TLC: CH_2_Cl_2_/CH_3_OH/NH_4_OH 95:5:0.5. ^1^H NMR (400 MHz, CDCl_3_) δ: 8.47 (s, 1H), 8.45 (s, 1H), 8.00–7.94 (m, 8H), 7.51–7.40 (m, 9H), 7.12 (d, *J* = 9.2 Hz, 1H), 6.68–6.66 (m, 2H), 6.12 (d, *J* = 9.6 Hz, 1H), 4.60 (t, *J* = 6.4 Hz, 2H), 4.53 (t, *J* = 6.4 Hz, 2H), 3.93 (t, *J* = 6.0 Hz, 2H), 2.58–2.52 (m, 4H), 2.38 (t, *J* = 7.2 Hz, 2H), 1.99–1.92 (m, 2H), 1.83–1.73 (m, 4H), 1.44–1.30 (m, 6H) ppm. ^13^C NMR (100 MHz, CDCl_3_) δ: 169.7 (C), 169.7 (C), 162.2 (C), 161.2 (C), 155.8 (C), 143.4 (CH), 142.7 (C), 141.9 (C), 131.0 (C), 129.3 (CH), 129.2 (CH), 128.6 (CH), 128.4 (C), 128.2 (C), 128.0 (C), 126.9 (CH), 125.5 (CH), 125.0 (CH), 125.0 (CH), 112.8 (CH), 112.7 (CH), 112.3 (C), 101.3 (CH), 66.4 (CH_2_), 65.8 (CH_2_), 64.1 (CH_2_), 54.0 (CH_2_), 50.6 (CH_2_), 50.2 (CH_2_), 28.7 (CH_2_), 27.1 (CH_2_), 26.9 (CH_2_), 26.7 (CH_2_), 26.1 (CH_2_) ppm. ESI-HRMS (*m*/*z*) calculated for [M + H]^+^ ion species C_51_H_48_NO_7_ = 786.3425, found 786.3424. *Hydrochloride*: yellow solid, mp 136–139 °C.

##### (*E*)-7-((3-((2-Oxo-2*H*-chromen-7-yl)oxy)propyl)(3-(((*E*)-3-(3,4,5-trimethoxyphenyl)acryloyl)oxy)propyl)amino)heptyl 3-(3,4,5-Trimethoxyphenyl)acrylate (**19**)

Following *method B*, compound **19** (0.050 g, yield: 56.6%) was synthesized as a pale yellow oil, starting from (*E*)-3-(3,4,5-trimethoxyphenyl)acrylic acid (0.029 g, 0.12 mmol) and **47** (0.050 g, 0.080 mmol). *Free base:* TLC: CH_2_Cl_2_/CH_3_OH/NH_4_OH 95:5:0.5. ^1^H NMR (400 MHz, CDCl_3_) δ: 7.55–7.51 (m, 3H), 7.28 (d, *J* = 9.2 Hz, 1H), 6.78–6.75 (m, 2H), 6.70 (s, 2H), 6.68 (s, 2H), 6.29 (d, *J* = 16.0 Hz, 1H), 6.26 (d, *J* = 16.0 Hz, 1H), 6.15 (d, *J* = 9.6 Hz, 1H), 4.18 (t, *J* = 6.0 Hz, 2H), 4.11 (t, *J* = 6.0 Hz, 2H), 4.03 (t, *J* = 6.0 Hz, 2H), 3.83 (s, 18H), 2.67–2.47 (m, 4H), 2.44–2.32 (m, 2H), 1.95–1.85 (m, 2H), 1.84–1.74 (m, 2H), 1.66–1.56 (m, 2H), 1.44–1.35 (m, 2H), 1.34–1.17 (m, 6H) ppm.

^13^C NMR (100 MHz, CDCl_3_) δ: 167.0 (C), 166.9 (C), 162.2 (C), 161.2 (C), 155.9 (C), 153.4 (C), 144.8 (CH), 144.6 (CH), 143.4 (CH), 140.2 (C), 140.1 (C), 129.9 (C), 129.8 (C), 128.7 (CH), 117.4 (CH), 117.1 (CH), 113.0 (CH), 112.7 (CH), 112.5 (C), 105.2 (CH), 105.2 (CH), 101.4 (CH), 66.4 (CH_2_), 64.6 (CH_2_), 62.7 (CH_2_), 61.0 (CH_3_), 56.2 (CH_3_), 54.0 (CH_2_), 50.5 (CH_2_), 50.2 (CH_2_), 29.7 (CH_2_), 29.2 (CH_2_), 28.7 (CH_2_), 27.3 (CH_2_), 25.9 (CH_2_) ppm. ESI-HRMS (*m*/*z*) calculated for [M + H]^+^ ion species C_46_H_58_NO_13_ = 832.3903, found 832.3903. *Hydrochloride*: white solid, mp 90–92 °C.

##### (*E*)-3-((3-((2-Oxo-2*H*-chromen-7-yl)oxy)propyl)(7-((3-(3,4,5-trimethoxyphenyl)acryloyl)oxy)heptyl)amino)propyl 3,4,5-Trimethoxybenzoate (**20**)

Following *method B*, compound **20** (0.026 g, yield: 33.0%) was synthesized as a pale yellow oil, starting from (*E*)-3-(3,4,5-trimethoxyphenyl)acrylic acid (0.035 g, 0.15 mmol) and **48** (0.058 g, 0.10 mmol). *Free base:* TLC: CH_2_Cl_2_/CH_3_OH/NH_4_OH 95:5:0.5. ^1^H NMR (400 MHz, CDCl_3_) δ: 7.59 (d, *J* = 9.2 Hz, 1H), 7.58 (d, *J* = 16.0 Hz, 1H), 7.32 (d, *J* = 8.4 Hz, 1H), 7.26 (s, 2H), 6.81–6.78 (m, 2H), 6.75 (s, 2H), 6.34 (d, *J* = 16.0 Hz, 1H), 6.21 (d, *J* = 9.2 Hz, 1H), 4.33 (t, *J* = 6.4 Hz, 2H), 4.15 (t, *J* = 6.4 Hz, 2H), 4.06 (t, *J* = 6.4 Hz, 2H), 3.89 (s, 3H), 3.88 (s, 6H), 3.87 (s, 6H), 3.86 (s, 3H), 2.70–2.50 (m, 4H), 2.49–2.31 (m, 2H), 2.03–1.82 (m, 4H), 1.75–1.57 (m, 2H), 1.51–1.39 (m, 2H), 1.33–1.22 (m, 6H) ppm. ^13^C NMR (100 MHz, CDCl_3_) δ: 167.0 (C), 166.1 (C), 162.1 (C), 161.1 (C), 155.9 (C), 153.4 (C), 152.9 (C), 144.6 (CH), 143.3 (CH), 142.2 (C), 140.1 (C), 129.9 (C), 128.8 (CH), 125.2 (C), 117.4 (CH), 113.1 (CH), 112.7 (CH), 112.5 (C), 106.8 (CH), 105.2 (CH), 101.4 (CH), 66.3 (CH_2_), 64.5 (CH_2_), 63.2 (CH_2_), 61.0 (CH_3_), 60.9 (CH_3_), 56.2 (CH_3_), 56.2 (CH_3_), 54.0 (CH_2_), 50.4 (CH_2_), 50.2 (CH_2_), 29.2 (CH_2_), 28.7 (CH_2_), 28.5 (CH_2_), 27.3 (CH_2_), 25.9 (CH_2_) ppm. ESI-HRMS (*m*/*z*) calculated for [M + H]^+^ ion species C_44_H_56_NO_13_ = 806.3746, found 806.3749. *Hydrochloride*: white solid, mp 84–87 °C.

##### (*E*)-3-((3-((2-Oxo-2*H*-chromen-7-yl)oxy)propyl)(7-((3-(3,4,5-trimethoxyphenyl)acryloyl)oxy)heptyl)amino)propyl Anthracene-9-carboxylate (**21**)

Following *method B*, compound **21** (0.080 g, yield: 86.0%) was synthesized as a pale yellow oil, starting from anthracene-9-carboxylic acid (0.038 g, 0.17 mmol) and **47** (0.070 g, 0.11 mmol). *Free base:* TLC: CH_2_Cl_2_/CH_3_OH/NH_4_OH 95:5:0.5. ^1^H NMR (400 MHz, CDCl_3_) δ: 8.48 (s, 1H), 7.98 (d, *J* = 8.4 Hz, 4H), 7.57–7.42 (m, 6H), 7.21 (d, *J* = 9.2 Hz, 1H), 6.73–6.70 (m, 4H), 6.30 (d, *J* = 16.0 Hz, 1H), 6.16 (d, *J* = 9.6 Hz, 1H), 4.62 (t, *J* = 6.4 Hz, 2H), 4.12 (t, *J* = 6.4 Hz, 2H), 3.98 (t, *J* = 6.4 Hz, 2H), 3.84 (s, 9H), 2.70–2.53 (m, 4H), 2.50–2.32 (m, 2H), 2.10–1.96 (m, 2H), 1.95–1.79 (m, 2H), 1.66–1.56 (m, 2H), 1.44–1.35 (m, 2H), 1.29–1.12 (m, 6H) ppm. ^13^C NMR (100 MHz, CDCl_3_) δ: 169.6 (C), 167.0 (C), 162.2 (C), 161.2 (C), 155.8 (C), 153.4 (C), 144.6 (CH), 143.4 (CH), 131.0 (C), 129.9 (C), 129.3 (CH), 128.7 (CH), 128.4 (C), 127.0 (CH), 125.5 (CH), 124.9 (CH), 117.5 (CH), 112.9 (CH), 112.7 (CH), 112.4 (C), 105.2 (CH), 101.4 (CH), 66.4 (CH_2_), 64.6 (CH_2_), 64.0 (CH_2_), 61.0 (CH_3_), 56.1 (CH_3_), 54.1 (CH_2_), 50.6 (CH_2_), 50.2 (CH_2_), 29.7 (CH_2_), 29.2 (CH_2_), 28.7 (CH_2_), 27.4 (CH_2_), 26.1 (CH_2_), 25.9 (CH_2_) ppm. ESI-HRMS (*m*/*z*) calculated for [M + H]^+^ ion species C_49_H_54_NO_10_ = 816.3742, found 816.3743. *Hydrochloride*: pale yellow solid, mp 100–102 °C.

##### (*E*)-7-((3-((2-Oxo-2*H*-chromen-7-yl)oxy)propyl)(3-((3-(3,4,5-trimethoxyphenyl)acryloyl)oxy)propyl)amino)heptyl 3,4,5-Trimethoxybenzoate (**22**)

Following *method A*, compound **22** (0.054 g, yield: 74.5%) was synthesized as a pale yellow oil, starting from **49** (0.055 g, 0.090 mmol) and 3,4,5-trimethoxybenzoic acid (0.029 g, 0.14 mmol). *Free base:* TLC: CH_2_Cl_2_/CH_3_OH/NH_4_OH 95:5:0.5. ^1^H NMR (400 MHz, CDCl_3_) δ: 7.53 (d, *J* = 9.6 Hz, 1H), 7.52 (d, *J* = 16.0 Hz, 1H), 7.27 (d, *J* = 8.8 Hz, 1H), 7.23 (s, 2H), 6.77–6.74 (m, 2H), 6.68 (s, 2H), 6.26 (d, *J* = 16.0 Hz, 1H), 6.14 (d, *J* = 9.6 Hz, 1H), 4.21 (t, *J* = 6.4 Hz, 2H), 4.17 (t, *J* = 6.4 Hz, 2H), 4.02 (t, *J* = 6.4 Hz, 2H), 3.84 (s, 3H), 3.84 (s, 6H), 3.82 (s, 6H), 3.82 (s, 3H), 2.55 (t, *J* = 6.4 Hz, 2H), 2.49 (t, *J* = 6.4 Hz, 2H), 2.37 (t, *J* = 6.4 Hz, 2H), 1.90–1.85 (m, 2H), 1.80–1.76 (m, 2H), 1.70–1.63 (m, 2H), 1.43–1.22 (m, 8H) ppm. ^13^C NMR (100 MHz, CDCl_3_) δ: 166.9 (C), 166.2 (C), 162.3 (C), 161.2 (C), 155.9 (C), 153.4 (C), 152.9 (C), 144.8 (CH), 143.4 (CH), 142.1 (C), 140.1 (C), 129.8 (C), 128.7 (CH), 125.5 (C), 117.2 (CH), 112.9 (CH), 112.7 (CH), 112.4 (C), 106.8 (CH), 105.2 (CH), 101.4 (CH), 66.5 (CH_2_), 65.2 (CH_2_), 62.8 (CH_2_), 61.0 (CH_3_), 60.9 (CH_3_), 56.2 (CH_3_), 56.2 (CH_3_), 54.1 (CH_2_), 50.5 (CH_2_), 50.2 (CH_2_), 29.3 (CH_2_), 28.7 (CH_2_), 27.4 (CH_2_), 26.9 (CH_2_), 26.0 (CH_2_) ppm. ESI-HRMS (*m*/*z*) calculated for [M + H]^+^ ion species C_44_H_56_NO_13_ = 806.3746, found 806.3751. *Hydrochloride*: white solid, mp 73–76 °C.

##### 7-((3-((2-Oxo-2*H*-chromen-7-yl)oxy)propyl)(3-((3,4,5-trimethoxybenzoyl)oxy)propyl)amino)heptyl 3,4,5-Trimethoxybenzoate (**23**)

Following *method A*, compound **23** (0.052 g, yield: 69.2%) was synthesized as a pale yellow oil, starting from **48** (0.056 g, 0.10 mmol) and 3,4,5-trimethoxybenzoic acid (0.031 g, 0.14 mmol). *Free base:* TLC: CH_2_Cl_2_/CH_3_OH/NH_4_OH 95:5:0.5. ^1^H NMR (400 MHz, CDCl_3_) δ: 7.57 (d, *J* = 9.6 Hz, 1H), 7.30 (d, *J* = 8.8 Hz, 1H), 7.27 (s, 2H), 7.25 (s, 2H), 6.79–6.76 (m, 2H), 6.19 (d, *J* = 9.6 Hz, 1H), 4.32 (t, *J* = 6.4 Hz, 2H), 4.25 (t, *J* = 6.4 Hz, 2H), 4.05 (t, *J* = 6.4 Hz, 2H), 3.88 (s, 12H), 3.87 (s, 6H), 2.64–2.52 (m, 4H), 2.46–2.35 (m, 2H), 1.97–1.84 (m, 4H), 1.75–1.66 (m, 2H), 1.46–1.24 (m, 8H) ppm. ^13^C NMR (100 MHz, CDCl_3_) δ: 166.2 (C), 166.1 (C), 162.2 (C), 161.2 (C), 155.8 (C), 152.9 (C), 143.4 (CH), 142.1 (C), 142.1 (C), 128.7 (CH), 125.5 (C), 125.3 (C), 113.0 (CH), 112.7 (CH), 112.4 (C), 106.7 (CH), 106.7 (CH), 101.3 (CH), 66.4 (CH_2_), 65.2 (CH_2_), 63.4 (CH_2_), 60.9 (CH_3_), 56.2 (CH_3_), 54.1 (CH_2_), 50.4 (CH_2_), 50.1 (CH_2_), 29.3 (CH_2_), 28.7 (CH_2_), 27.4 (CH_2_), 26.9 (CH_2_), 26.5 (CH_2_), 26.0 (CH_2_) ppm. ESI-HRMS (*m*/*z*) calculated for [M + H]^+^ ion species C_42_H_54_NO_13_ = 780.3590, found 780.3589. *Hydrochloride*: pale yellow solid, mp 74–77 °C.

##### 3-((3-((2-Oxo-2*H*-chromen-7-yl)oxy)propyl)(7-((3,4,5-trimethoxybenzoyl)oxy)heptyl)amino)propyl Anthracene-9-carboxylate (**24**)

Following *method A*, compound **24** (0.11 g, yield: 92.3%) was synthesized as a yellow oil, starting from **50** (0.090 g, 0.15 mmol) and 3,4,5-trimethoxybenzoic acid (0.048 g, 0.23 mmol). *Free base:* TLC: CH_2_Cl_2_/CH_3_OH/NH_4_OH 95:5:0.5. ^1^H NMR (400 MHz, CDCl_3_) δ: 8.51 (s, 1H), 8.01 (d, *J* = 8.8 Hz, 4H), 7.55–7.45 (m, 5H), 7.28 (s, 2H), 7.25 (d, *J* = 8.4 Hz, 1H), 6.76–6.74 (m, 2H), 6.19 (d, *J* = 9.2 Hz, 1H), 4.65 (t, *J* = 6.4 Hz, 2H), 4.25 (t, *J* = 6.4 Hz, 2H), 4.01 (t, *J* = 6.4 Hz, 2H), 3.89 (s, 3H), 3.89 (s, 6H), 2.82–2.54 (m, 4H), 2.53–2.38 (m, 2H), 2.13–1.98 (m, 2H), 1.97–1.84 (m, 2H), 1.76–1.66 (m, 2H), 1.53–1.39 (m, 2H), 1.38–1.23 (m, 6H) ppm. ^13^C NMR (100 MHz, CDCl_3_) δ: 169.6 (C), 166.2 (C), 162.2 (C), 161.2 (C), 155.8 (C), 152.9 (C), 143.4 (CH), 142.2 (C), 131.0 (C), 129.3 (CH), 128.7 (CH), 128.6 (CH), 128.3 (C), 128.0 (C), 126.9 (CH), 125.5 (C), 125.5 (CH), 124.9 (CH), 112.8 (CH), 112.7 (CH), 112.4 (C), 106.8 (CH), 101.3 (CH), 66.5 (CH_2_), 65.2 (CH_2_), 64.1 (CH_2_), 60.9 (CH_3_), 56.2 (CH_3_), 54.2 (CH_2_), 50.7 (CH_2_), 50.2 (CH_2_), 29.2 (CH_2_), 28.7 (CH_2_), 27.4 (CH_2_), 27.2 (CH_2_), 26.9 (CH_2_), 26.8 (CH_2_), 26.0 (CH_2_) ppm. ESI-HRMS (*m*/*z*) calculated for [M + H]^+^ ion species C_47_H_52_NO_10_ = 790.3586, found 790.3586. *Hydrochloride*: pale yellow solid, mp 75–77 °C.

##### (*E*)-7-((3-((2-Oxo-2*H*-chromen-7-yl)oxy)propyl)(3-((3-(3,4,5-trimethoxyphenyl)acryloyl)oxy)propyl)amino)heptyl Anthracene-9-carboxylate (**25**)

Following *method B*, compound **25** (0.13 g, yield: 93.5%) was synthesized as a pale yellow oil, starting from anthracene-9-carboxylic acid (0.060 g, 0.27 mmol) and **49** (0.11 g, 0.18 mmol). *Free base:* TLC: CH_2_Cl_2_/CH_3_OH/NH_4_OH 95:5:0.5. ^1^H NMR (400 MHz, CDCl_3_) δ: 8.46 (s, 1H), 7.97 (t, *J* = 8.4 Hz, 4H), 7.54 (d, *J* = 16.0 Hz, 1H), 7.51–7.41 (m, 5H), 7.21 (d, *J* = 9.6 Hz, 1H), 6.74–6.71 (m, 2H), 6.69 (s, 2H), 6.28 (d, *J* = 16.0 Hz, 1H), 6.12 (d, *J* = 9.2 Hz, 1H), 4.55 (t, *J* = 6.4 Hz, 2H), 4.19 (t, *J* = 6.4 Hz, 2H), 3.99 (t, *J* = 6.4 Hz, 2H), 3.83 (s, 3H), 3.82 (s, 6H), 2.56–2.48 (m, 4H), 2.39–2.34 (m, 2H), 1.91–1.74 (m, 6H), 1.47–1.21 (m, 8H) ppm. ^13^C NMR (100 MHz, CDCl_3_) δ: 169.7 (C), 166.9 (C), 162.2 (C), 161.2 (C), 155.8 (C), 153.4 (C), 144.7 (CH), 143.4 (CH), 140.1 (C), 131.0 (C), 129.9 (C), 129.2 (CH), 128.7 (CH), 128.6 (CH), 128.3 (C), 128.2 (C), 126.9 (CH), 125.5 (CH), 125.0 (CH), 117.2 (CH), 112.8 (CH), 112.6 (CH), 112.4 (C), 105.2 (CH), 101.4 (CH), 66.5 (CH_2_), 65.9 (CH_2_), 62.8 (CH_2_), 61.0 (CH_3_), 56.1 (CH_3_), 54.1 (CH_2_), 50.5 (CH_2_), 50.1 (CH_2_), 29.2 (CH_2_), 28.7 (CH_2_), 27.4 (CH_2_), 27.1 (CH_2_), 26.9 (CH_2_), 26.6 (CH_2_), 26.1 (CH_2_) ppm. ESI-HRMS (*m*/*z*) calculated for [M + H]^+^ ion species C_49_H_54_NO_10_ = 816.3742, found 816.3744. *Hydrochloride*: pale yellow solid, mp 107–110 °C.

##### 7-((3-((2-Oxo-2*H*-chromen-7-yl)oxy)propyl)(3-((3,4,5-trimethoxybenzoyl)oxy)propyl)amino)heptyl Anthracene-9-carboxylate (**26**)

Following *method B*, compound **26** (0.073 g, yield: 77.7%) was synthesized as a pale yellow oil, starting from anthracene-9-carboxylic acid (0.040 g, 0.18 mmol) and **48** (0.070 g, 0.12 mmol). *Free base:* TLC: CH_2_Cl_2_/CH_3_OH/NH_4_OH 95:5:0.5. ^1^H NMR (400 MHz, CDCl_3_) δ: 8.47 (s, 1H), 8.01 (d, *J* = 8.4 Hz, 2H), 7.98 (d, *J* = 8.4 Hz, 2H), 7.53–7.43 (m, 5H), 7.25 (s, 2H), 7.21 (d, *J* = 9.2 Hz, 1H), 6.74–6.71 (m, 2H), 6.15 (d, *J* = 9.6 Hz, 1H), 4.58 (t, *J* = 6.4 Hz, 2H), 4.31 (t, *J* = 6.4 Hz, 2H), 3.99 (t, *J* = 6.4 Hz, 2H), 3.88 (s, 3H), 3.86 (s, 6H), 2.58–2.52 (m, 4H), 2.40 (t, *J* = 6.4 Hz, 2H), 1.88–1.79 (m, 6H), 1.48–1.37 (m, 4H), 1.36–1.23 (m, 4H) ppm. ^13^C NMR (100 MHz, CDCl_3_) δ: 169.7 (C), 166.1 (C), 162.2 (C), 161.2 (C), 155.8 (C), 152.9 (C), 143.4 (CH), 142.2 (C), 131.0 (C), 129.2 (CH), 128.7 (CH), 128.6 (CH), 128.4 (C), 128.2 (C), 126.9 (CH), 125.5 (CH), 125.3 (C), 125.0 (CH), 112.9 (CH), 112.6 (CH), 112.4 (C), 106.8 (CH), 101.3 (CH), 66.4 (CH_2_), 65.9 (CH_2_), 63.4 (CH_2_), 60.9 (CH_3_), 56.2 (CH_3_), 54.1 (CH_2_), 50.4 (CH_2_), 50.1 (CH_2_), 29.2 (CH_2_), 28.7 (CH_2_), 27.4 (CH_2_), 26.9 (CH_2_), 26.6 (CH_2_), 26.1 (CH_2_) ppm. ESI-HRMS (*m*/*z*) calculated for [M + H]^+^ ion species C_47_H_52_NO_10_ = 790.3586, found 790.3585. *Hydrochloride*: yellow solid; mp 97–99 °C.

##### 3-((7-((Anthracene-9-carbonyl)oxy)heptyl)(3-((2-oxo-2*H*-chromen-7-yl)oxy)propyl)amino)propyl Anthracene-9-carboxylate (**27**)

Following *method B*, compound **27** (0.11 g, yield: 91.1%) was synthesized as a yellow oil, starting from anthracene-9-carboxylic acid (0.050 g, 0.23 mmol) and **50** (0.090 g, 0.15 mmol). *Free base:* TLC: CH_2_Cl_2_/CH_3_OH/NH_4_OH 95:5:0.5. ^1^H NMR (400 MHz, CDCl_3_) δ: 8.48 (s, 2H), 8.05–7.98 (m, 8H), 7.54–7.43 (m, 9H), 7.15 (d, *J* = 8.8 Hz, 1H), 6.71–6.69 (m, 2H), 6.15 (d, *J* = 9.2 Hz, 1H), 4.65 (t, *J* = 6.4 Hz, 2H), 4.58 (t, *J* = 6.4 Hz, 2H), 3.96 (t, *J* = 6.4 Hz, 2H), 2.70–2.49 (m, 4H), 2.42 (m, 2H), 2.07–1.95 (m, 2H), 1.91–1.78 (m, 4H), 1.48–1.36 (m, 4H), 1.34–1.22 (m, 4H) ppm. ^13^C NMR (100 MHz, CDCl_3_) δ: 169.8 (C), 169.7 (C), 162.2 (C), 161.3 (C), 155.8 (C), 143.4 (CH), 131.0 (C), 129.3 (CH), 129.2 (CH), 128.7 (CH), 128.4 (C), 128.2 (C), 128.0 (C), 126.9 (CH), 125.5 (CH), 125.0 (CH), 125.0 (CH), 112.8 (CH), 112.7 (CH), 112.4 (C), 101.3 (CH), 66.4 (CH_2_), 65.9 (CH_2_), 64.1 (CH_2_), 54.1 (CH_2_), 50.6 (CH_2_), 50.2 (CH_2_), 29.2 (CH_2_), 28.7 (CH_2_), 27.4 (CH_2_), 27.0 (CH_2_), 26.8 (CH_2_), 26.7 (CH_2_), 26.1 (CH_2_) ppm. ESI-HRMS (*m*/*z*) calculated for [M + H]^+^ ion species C_52_H_50_NO_7_ = 800.3582, found 800.3578. *Hydrochloride*: pale yellow solid; mp 117–119 °C.

### Stability Tests

#### Chemicals

Acetonitrile (Chromasolv), formic acid and ammonium formate (MS grade), NaCl, KCl, Na_2_HPO_4_ 2H_2_O, and KH_2_PO_4_ (Reagent grade), verapamil hydrochloride (analytical standard, used as the internal standard or IS), and ketoprofen (analytical standard) were purchased from Sigma-Aldrich (Milan, Italy). Ketoprofen ethyl ester (KEE) was obtained by Fisher’s reaction from ketoprofen and ethanol.

Ultrapure water or mQ water (18 MΩ cm) was obtained from Millipore’s Simplicity system (Milan, Italy).

Phosphate-buffered solution (PBS) was prepared by adding 8.01 g L^–1^ of NaCl, 0.2 g L^–1^ of KCl, 1.78 g L^–1^ of Na_2_HPO_4_ 2H_2_O, and 0.27 g L^–1^ of KH_2_PO_4_. Human plasma was collected from healthy volunteers, pooled in a single batch, and kept at −80 °C until use.

#### Preparation of Samples

Each sample was prepared by adding 10 μL of working solution 1 (for details, see the Supporting Information) to 100 μL of the tested matrix (PBS or human plasma) in microcentrifuge tubes. The obtained solutions correspond to 1 μM of the analyte.

Each set of samples was incubated in triplicate at four different times, 0, 30, 60, and 120 min at 37 °C. Therefore, the degradation profile of each analyte was represented by a batch of 12 samples (4 incubation times × 3 replicates). After the incubation, the samples were added with 300 μL of IS solution (for details, see the Supporting Information) and centrifuged (room temperature for 5 min at 10 000 rpm). The supernatants were transferred in auto sampler vials and dried under a gentle stream of nitrogen. The dried samples were dissolved in 1.0 mL of 10 mM formic acid in mQ water: acetonitrile 80:20 solution. The obtained sample solutions were analyzed by LC-MS/MS methods described in the Supporting Information.

### CA Inhibition Assay

An SX.18MV-R Applied Photophysics (Oxford, U.K.) stopped-flow instrument has been used to assay the catalytic/inhibition of various CA isozymes.^38^ Phenol Red (at a concentration of 0.2 mM) has been used as an indicator, working at an absorbance maximum of 557 nm, with 10 mM Hepes (pH 7.4) as a buffer, 0.1 M Na_2_SO_4_ or NaClO_4_ (for maintaining constant the ionic strength; these anions are not inhibitory in the used concentration), following the CA-catalyzed CO_2_ hydration reaction for a period of 5–10 s. Saturated CO_2_ solutions in water at 25 °C were used as a substrate. Stock solutions of inhibitors were prepared at a concentration of 10 μM (in DMSO–water 1:1, v/v), and dilutions up to 0.01 nM were done with the assay buffer mentioned above. At least seven different inhibitor concentrations have been used for measuring the inhibition constant. Inhibitor and enzyme solutions were preincubated together for 10 min at room temperature prior to assay to allow for the formation of the E–I complex. Triplicate experiments were done for each inhibitor concentration, and the values reported throughout the paper are the mean of such results. The inhibition constants were obtained by nonlinear least-squares methods using the Cheng–Prusoff equation, as reported earlier,^32^ and represent the mean from at least three different determinations. All CA isozymes used here were recombinant proteins obtained as reported earlier by our group, and their concentrations in the assay system were 5.6–12 nM.^65,66^

### Cells

Human chemosensitive colon cancer HT29 cells, lung cancer A549 cells, not-transformed human colon epithelial EpiCoC cells, and lung epithelial BEAS-2B cells were purchased from ATCC (Manassas, VA). The K562 leukemia cells derived from a patient with chronic myelogenous leukemia^41^ and the P-gp overexpressing K562/DOX cells were obtained from Prof. J. P. Marie (Hopital Hotel-Dieu, Paris, France). These cells were cultured in RPMI 1640 medium supplemented with 10% fetal calf serum (FCS; GIBCO) at 37 ◦C in a humidified incubator with 5% CO_2_. To maintain the resistance, every month, resistant cells were cultured for 3 days with 400 nM doxorubicin. Human HT29/DOX and A549/DOX were generated by stepwise selection in medium with increasing concentration of doxorubicin, as reported by us,^67^ and maintained in the culture medium with a final concentration of 200 and 100 nM doxorubicin, respectively. All cell lines were authenticated by microsatellite analysis, using the PowerPlex kit (Promega Corporation, Madison, WI; last authentication: January 2022). Cells were maintained in media supplemented with 10% v/v fetal bovine serum, 1% v/v penicillin–streptomycin, and 1% v/v l-glutamine. To generate KO clones, 5 × 10^5^ cells were transduced with 1 μg of RNA vector (CRISPR pCas guide vector) for CAXII, P-gp or 1 μg not-targeting vector, mixed with 1 μg of donor DNA vector (Origene, Rockville, MD), following the manufacturer’s instructions. Stable knockout cells were selected in complete medium containing 1 μg/mL of puromycin for 3 weeks. Knockout efficacy was evaluated by immunoblotting, as reported below.

MDCK-MDR1, MDCK-MRP1, and MDCK-BCRP cells are a gift from Prof. P. Borst, NKI-AVL Institute, Amsterdam, The Netherlands. Cells were grown in Dulbecco’s modified Eagle’s medium (DMEM) high glucose supplemented with 10% fetal bovine serum, 2 mM glutamine, 100 U/mL of penicillin, and 100 μg/mL of streptomycin in a humidified incubator at 37 °C with a 5% CO_2_ atmosphere. Cell culture reagents were purchased from Celbio s.r.l. (Milano, Italy). CulturePlate 96/wells plates were purchased from PerkinElmer Life Science (Waltham, MA) and Falcon (BD Biosciences, Bedford, MA). Calcein-AM and Hoechst 33342 (bisbenzimide H 33342 trihydrochloride) were obtained from Sigma-Aldrich (Milan, Italy). The other reagents were purchased from Sigma Merck Millipore.

### Drugs and Chemicals

Doxorubicin hydrochloride, verapamil hydrochloride, dimethylsulfoxide (DMSO), and 3-(4,5-dimethylthiazolyl-2)-2,5-diphenyl tetrazolium bromide (MTT) were purchased from Sigma-Aldrich (Milan Italy). Stock solutions of the tested compounds as hydrochloride salts were prepared in DMSO at 10^–2^ M. Stock solutions of doxorubicin hydrochloride and verapamil hydrochloride were prepared in water at 10^–2^ M. All of the stock solutions were then diluted with complete medium to obtain the 10x desired final maximum test concentrations.

### Immunoblotting

Cells were rinsed with ice-cold lysis buffer (50 mM, tris, 10 mM ethylenediamine tetraacetic acid (EDTA), 1% v/v Triton-X100), supplemented with the protease inhibitor cocktail set III (80 μM aprotinin, 5 mM bestatin, 1.5 mM leupeptin, 1 mM pepstatin; Calbiochem, San Diego, CA), 2 mM phenylmethylsulfonyl fluoride, and 1 mM Na_3_VO_4_, then sonicated and centrifuged at 13 000*g* for 10 min at 4 °C. Protein extracts (20 μg) were subjected to sodium dodecyl sulfate polyacrylamide gel electrophoresis (SDS-PAGE) and probed with the following antibodies: anti-P-gp (C219, Calbiochem), anti-MRP1 (Abcam, Cambridge, U.K.), anti-CAXII (Abcam, Cambridge, U.K.), and anti-β-tubulin (Santa Cruz Biotechnology Inc., Santa Cruz, CA), followed by a peroxidase-conjugated secondary antibody (Bio-Rad Laboratories). The membranes were washed with tris-buffered saline-Tween 0.1% v/v solution, and the proteins were detected by enhanced chemiluminescence (Bio-Rad Laboratories).

### Coadministration Assays in K562/DOX, HT29/DOX, and A549/DOX Cells

K562/DOX cells were incubated for 72 h with compounds (1, 3, 10 μM) alone for intrinsic cytotoxicity and with doxorubicin in combination with 1 or 3 μM of the indicated compounds; HT29/DOX and A549/DOX cells were incubated for 72 h with doxorubicin, in the range from 10 nM to 30 μM, alone or in combination with 1 or 3 μM of the indicated compounds. Cell viability of the three cell lines was measured by the MTT assay, using a Synergy HT microplate spectrofluorimeter (Bio-Tek Instruments, Winooski, VT). The absorbance of untreated cells was considered 100%; results were expressed as the percentage of viable treated cells versus the control untreated cells. IC_50_ was the concentration killing 50% of cells and was determined graphically from relative survival curves obtained by GraphPad Prism 5 software (GraphPad, San Diego, CA).

### pHi Measurement

pH_i_ was measured by incubating whole cells with 5 μM 2′,7′-bis-(2-carboxyethyl)-5-(and-6)-carboxyfluorescein acetoxymethyl ester for 15 min at 37 °C and reading the intracellular fluorescence by a FACSCalibur flow cytometer (Becton Dickinson). The intracellular fluorescence was converted into pH units according to a titration curve, as described previously.^68^

### Intracellular Doxorubicin Accumulation and Kinetic Parameters

Cells were incubated for 3 h with 5 μM doxorubicin, alone or with the selected compounds, washed with PBS, trypsinized, centrifuged at 13 000*g* for 5 min, and resuspended in 0.5 mL of 1/1 solution ethanol/0.3 N HCl. A 50 μL aliquot was sonicated and used for the measurement of the protein content. The intracellular fluorescence of doxorubicin was measured spectrofluorimetrically using a Synergy HT microplate spectrofluorimeter (Bio-Tek Instruments). Excitation and emission wavelengths were 475 and 553 nm, respectively. Fluorescence was converted in nmol/mg cell proteins using a calibration curve previously set. To calculate the kinetic parameters, cells were incubated for 20 min with increasing (0–100 μM) concentrations of doxorubicin, alone or with the indicated compounds, then analyzed for the intracellular concentration of doxorubicin. A second series of dishes, after the incubation under the same experimental conditions for a further 10 min at 37 °C, were washed and tested for the intracellular drug content. The difference in the doxorubicin concentration between the two series, expressed as nmol doxorubicin extruded/min/mg cell proteins, was plotted versus the initial drug concentration.^69^ Values were fitted to the Michaelis–Menten equation to calculate *V*_max_ and *K*_m_, using the Enzfitter software (Biosoft Corporation, Cambridge, United Kingdom).

### Statistical Analysis

All data in the text and figures are provided as means ± SD. The results were analyzed by one-way analysis of variance (ANOVA) and Tukey’s test, using Statistical Package for Social Science (SPSS) software (IBM SPSS Statistics v.19). *p* < 0.05 was considered significant.

## Supporting Information Available

^1^H NMR (400 MHz), ^13^C-APT-NMR (100 MHz) spectra of compounds **1–27**; chemical stability data of compounds **1–27** and reference compound KEE; analytical method used to determine the purity and chromatographic profiles of HPLC–DAD analysis of representative compounds (**2**, **3**, **5**, **6**, **8**, **9**, **13**–**17**); UV spectra of compounds **2**, **3**, **5**, **6**, **8**, **9**, **13**–**17**; expression levels of P-gp and hCA XII in sensitive HT29 and A549 human cancer cell lines, in resistant wild-type HT29/DOX and A549/DOX cells, and in their P-gp or hCA XII knockout (KO) counterparts; cytotoxicity on HT29, HT29/DOX, A549, and A549/DOX cell lines of selected compounds (**1**, **2**, **4**, **5**, **10**, **11**, **13**, **14**, **19**, **20**, **22**, **23**); and reduction of viability in EpiCoc and BEARS-2B cell lines of selected compounds (**1**, **2**, **4**, **5**, **10**, **11**, **13**, **14**, **19**, **20**, **22**, **23**) (PDF)
SMILES format representations of compounds (CSV)

## Abbreviations Used

AAZ: acetazolamide
BCRP: breast cancer resistance protein
CA: carbonic anhydrase
DMAP: 4-dimethylaminopyridine
Doxo: doxorubicin
EDC: 1-ethyl-3-(3′-dimethylaminopropyl)carbodiimide
KEE: ketoprofen ethyl ester
KO: knockout
MDCK: Madin-Darby Canine Kidney
MDR: multidrug resistance
MRP1: multidrug-resistance-associated protein-1
MTT: 3-(4,5-dimethylthiazolyl-2)-2,5-diphenyl tetrazolium bromide
PBS: phosphate-buffered solution
P-gp: P-glycoprotein
RF: reversal fold
