# Supplementary material for: New Dual P-Glycoprotein (P-gp) and Human Carbonic Anhydrase XII (hCA XII) Inhibitors as Multidrug Resistance (MDR) Reversers in Cancer Cells
Source: J Med Chem. 2022 Oct 21;65(21):14655–72. doi: 10.1021/acs.jmedchem.2c01175 (PMC9661477; doi:10.1021/acs.jmedchem.2c01175)
Supplement: Supplementary file 1 — jm2c01175_si_001.pdf [file jm2c01175_si_001.pdf]

Supporting information

New dual P-glycoprotein (P-gp) and human carbonic anhydrase XII (hCA XII) inhibitors as multidrug resistance (MDR) reversers in cancer cells

Laura Braconi,[§] Elisabetta Teodori,*[§] Chiara Riganti,[‡] Marcella Coronnello,[†] Alessio Nocentini,[§] Gianluca Bartolucci,[§] Marco Pallecchi,[§] Marialessandra Contino,[$] Dina Manetti,[§] Maria Novella Romanelli,[§] Claudiu T. Supuran,[§] Silvia Dei[§]

[§]Department of Neuroscience, Psychology, Drug Research and Child Health - Section of Pharmaceutical and Nutraceutical Sciences, University of Florence, via Ugo Schiff 6, 50019 Sesto Fiorentino (FI), Italy.
[‡]Department of Oncology, University of Turin, Via Santena 5/bis, 10126 Torino, Italy.
[†]Department of Health Sciences - Clinical Pharmacology and Oncology Section, University of Florence, Viale Pieraccini 6, 50139 Firenze, Italy
[$]Department of Pharmacy - Drug Sciences, University of Bari "A. Moro", via Orabona 4, 70125, Bari, Italy.

Corresponding Author: Elisabetta Teodori: e-mail, elisabetta.teodori@unifi.it

Table of Contents

- ^{1}H-NMR (400 MHz), ^{13}C-APT- NMR (100 MHz) spectra of compounds **1-27** S2
- Chemical stability data of compounds **1-27** and reference compound KEE S29
- Analytical method used to determine purity and chromatographic profiles of HPLC-DAD analysis of representative compounds (**2**, **3**, **5**, **6**, **8**, **9**, **13-17**) S45
- UV spectra of compounds **2**, **3**, **5**, **6**, **8**, **9**, **13-17** S51
- Expression levels of P-gp and hCA XII in sensitive HT29 and A549 human cancer cell lines, in resistant wild-type HT29/DOX and A549/DOX cells, and in their P-gp or hCA XII knock-out (KO) counterparts S54
- Cytotoxicity on K562/DOX cells of compounds **1-27** S55
- Cytotoxicity on HT29, HT29/DOX, A549, A549/DOX cell lines of selected compounds **1**, **2**, **4**, **5**, **10**, **11**, **13**, **14**, **19**, **20**, **22**, **23** S56
- Reduction of viability EpiCoc and BEARS-2B cell lines on data of selected compounds **1**, **2**, **4**, **5**, **10**, **11**, **13**, **14**, **19**, **20**, **22**, **23** S57

^{1}H-NMR and ^{13}C-APT-NMR spectra of compound **1**

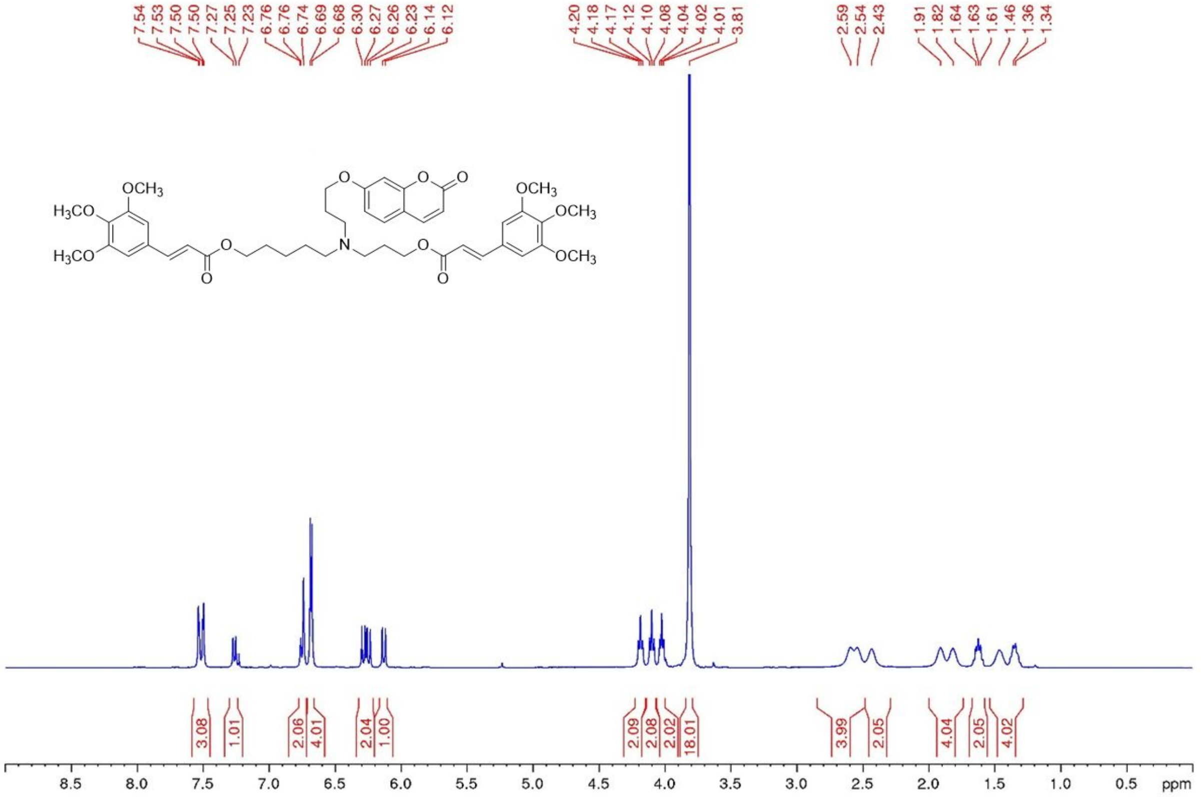

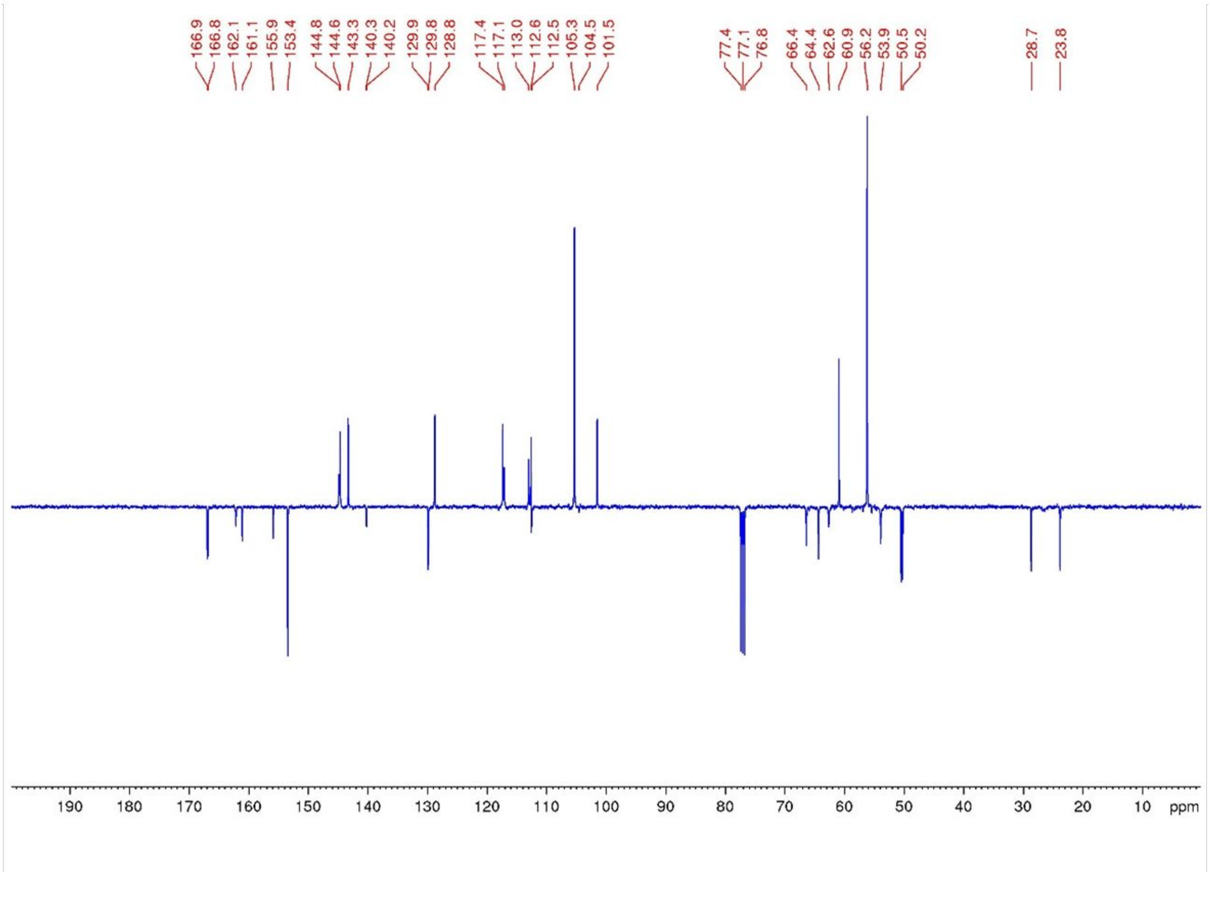

^{1}H-NMR and ^{13}C-APT-NMR spectra of compound **2**

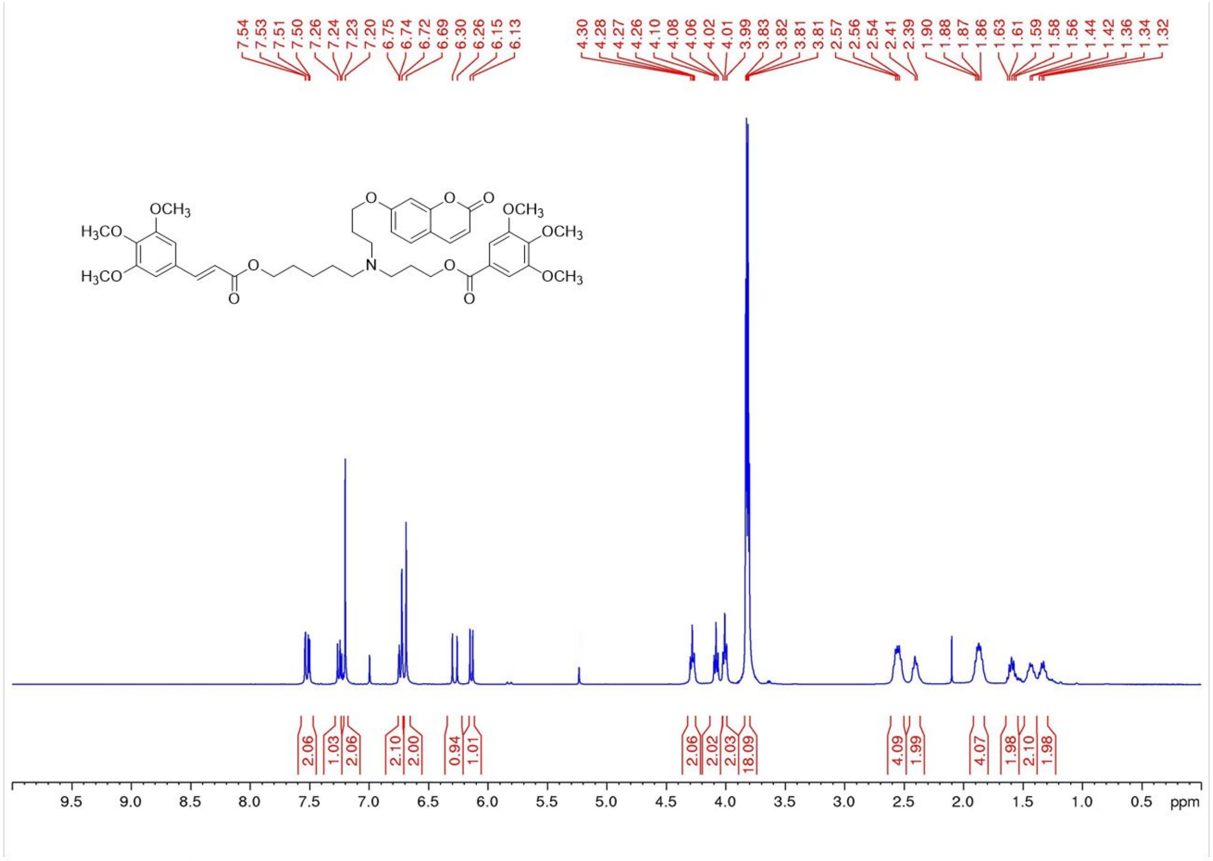

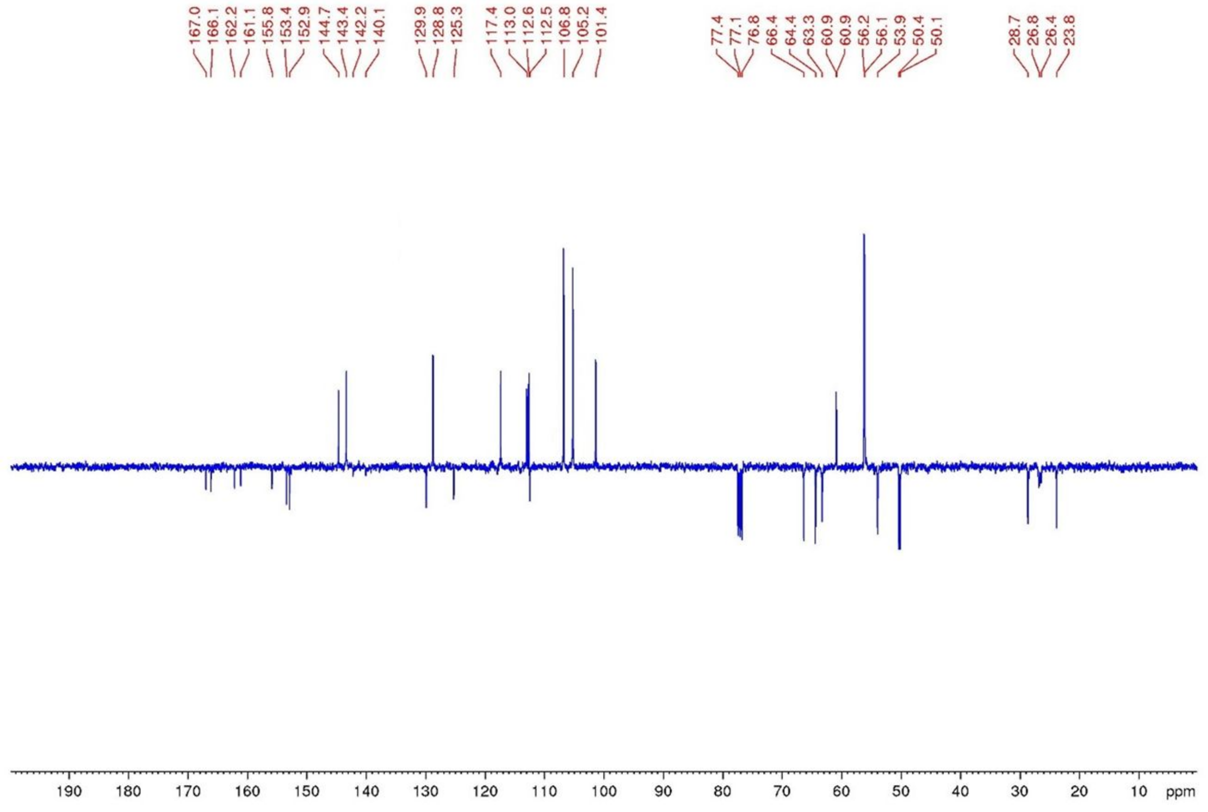

^{1}H-NMR and ^{13}C-APT-NMR spectra of compound **3**

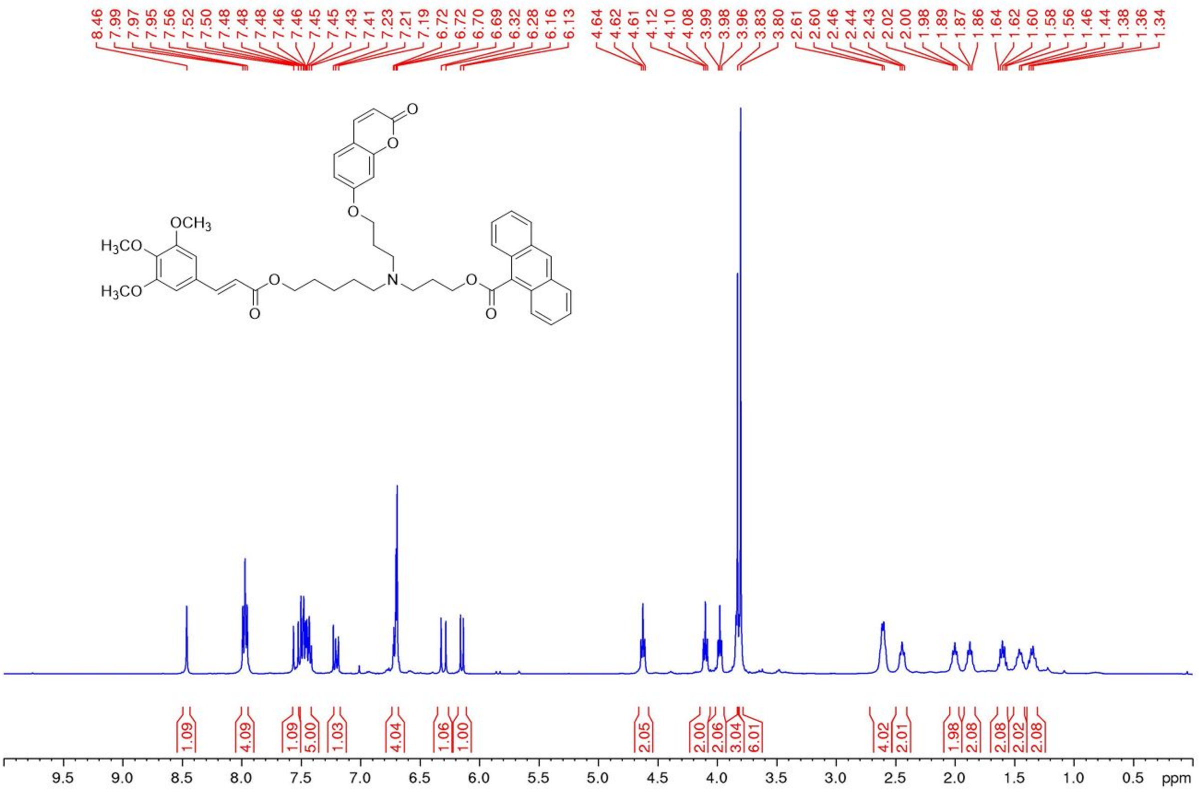

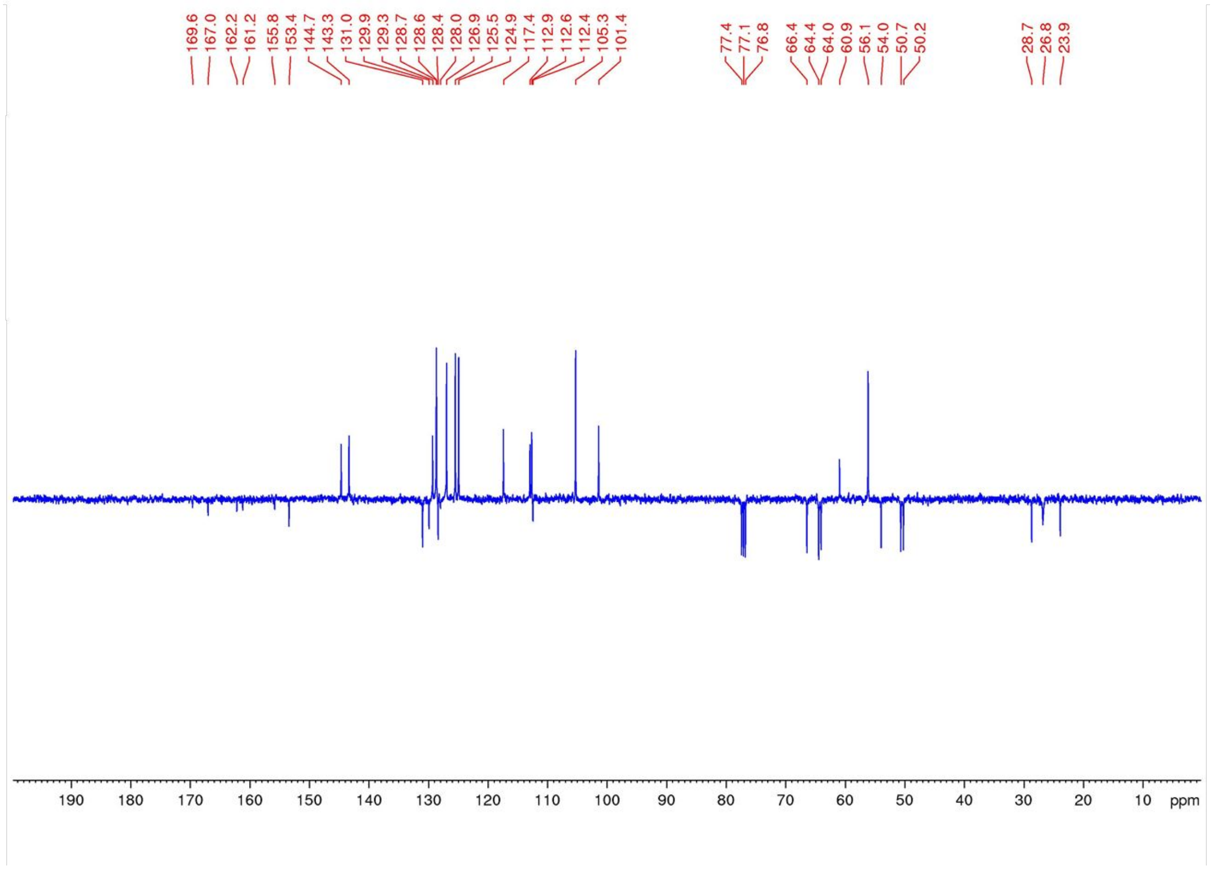

^{1}H-NMR and ^{13}C-APT-NMR spectra of compound **4**

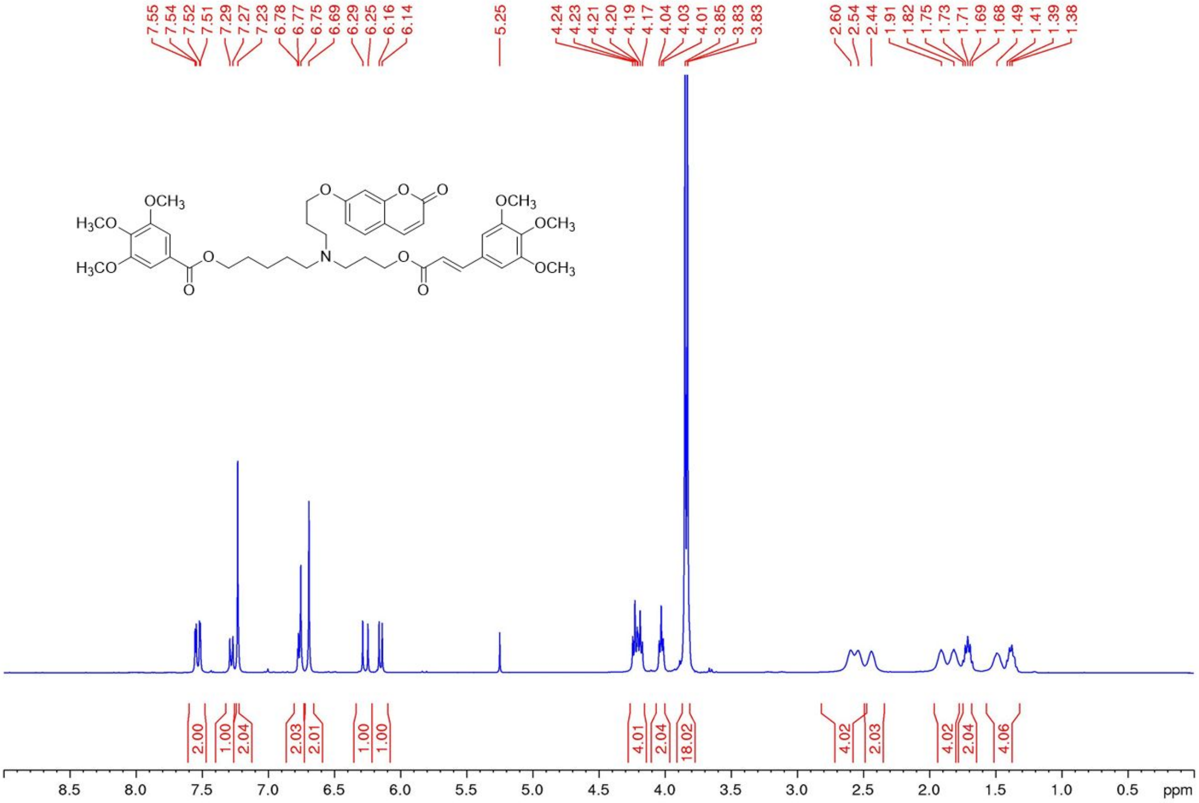

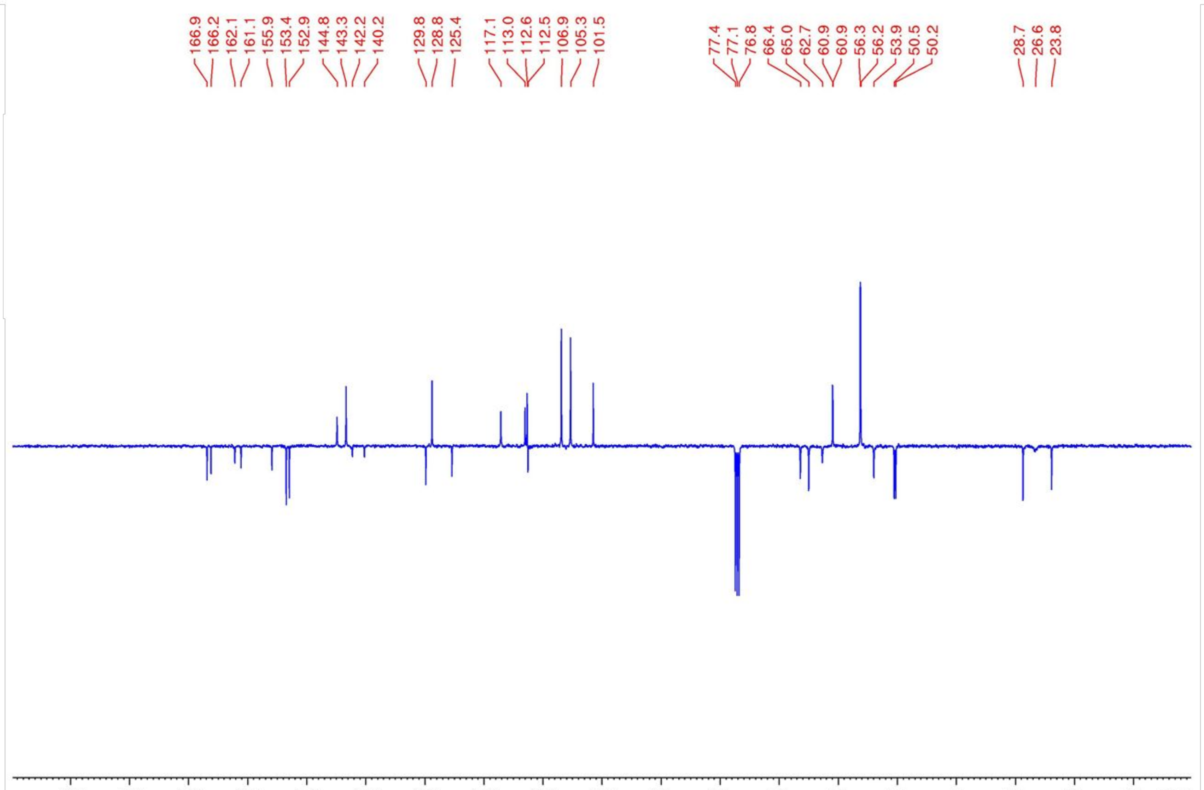

^{1}H-NMR and ^{13}C-APT-NMR spectra of compound **5**

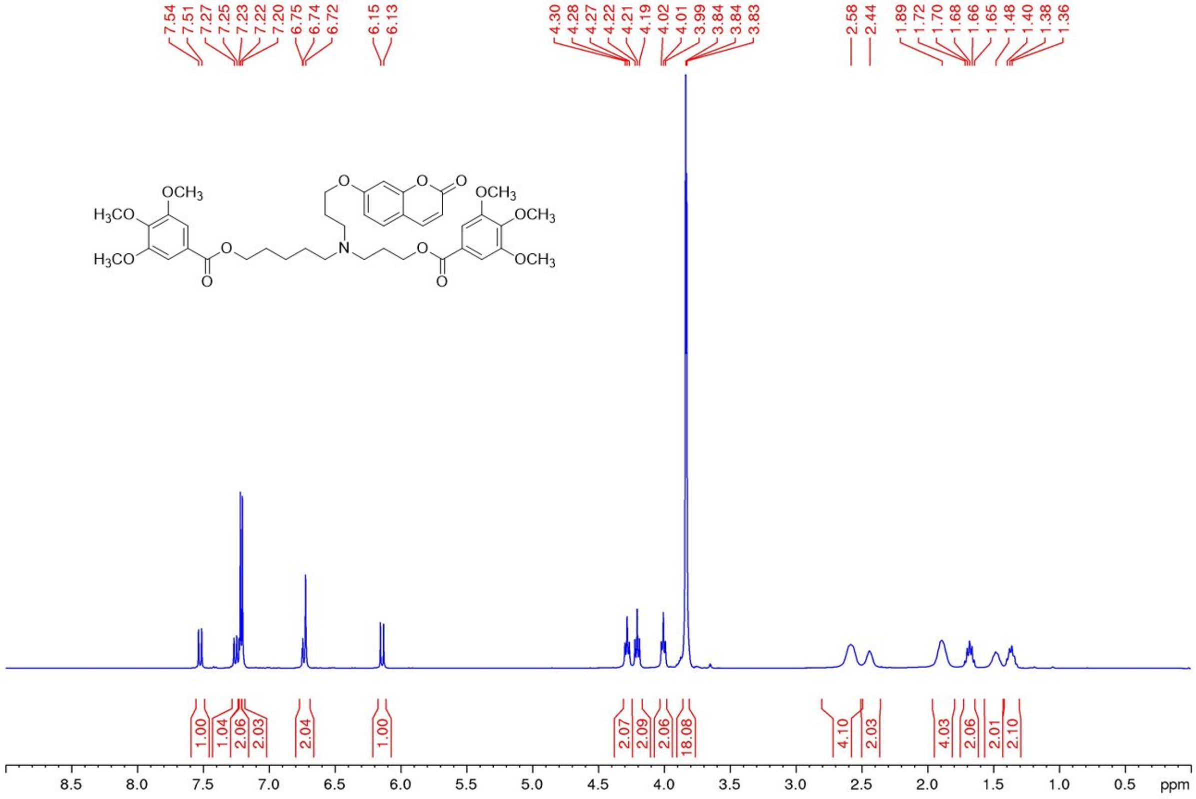

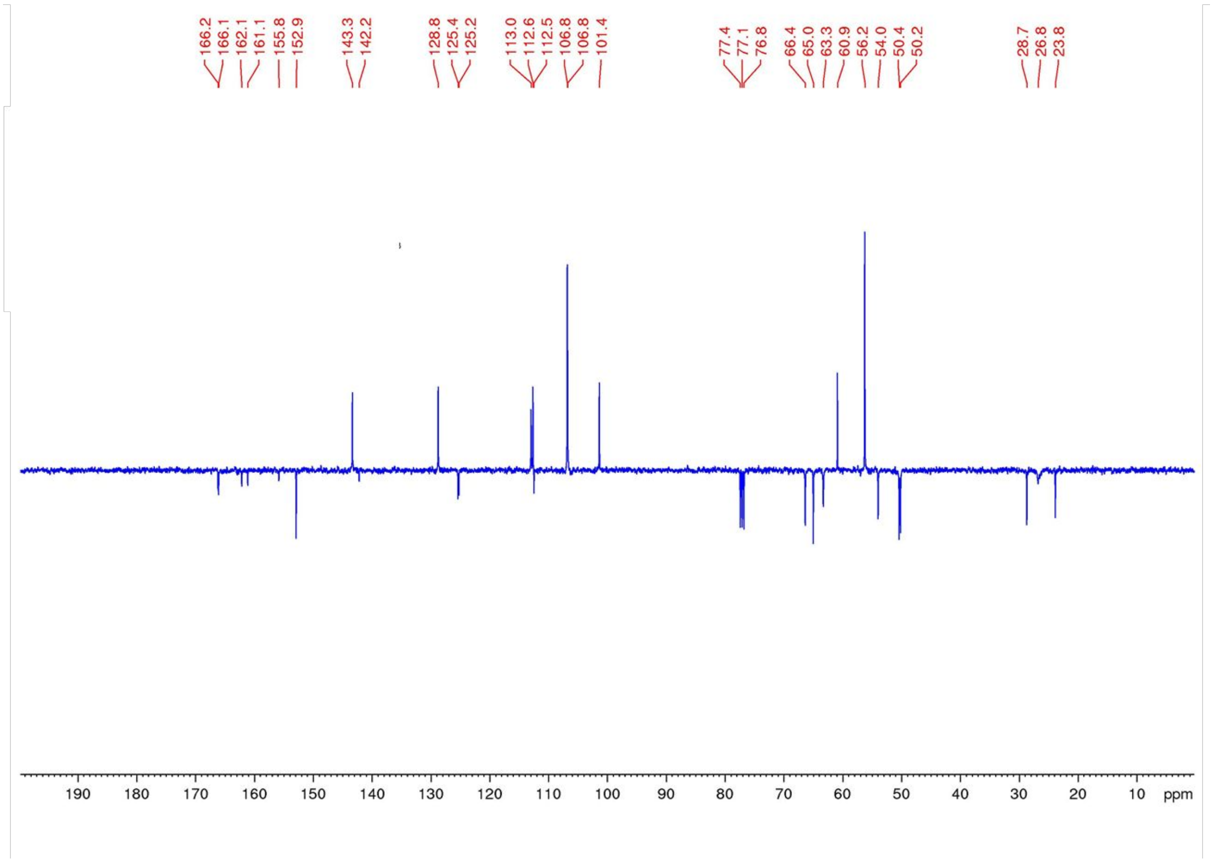

^{1}H-NMR and ^{13}C-APT-NMR spectra of compound **6**

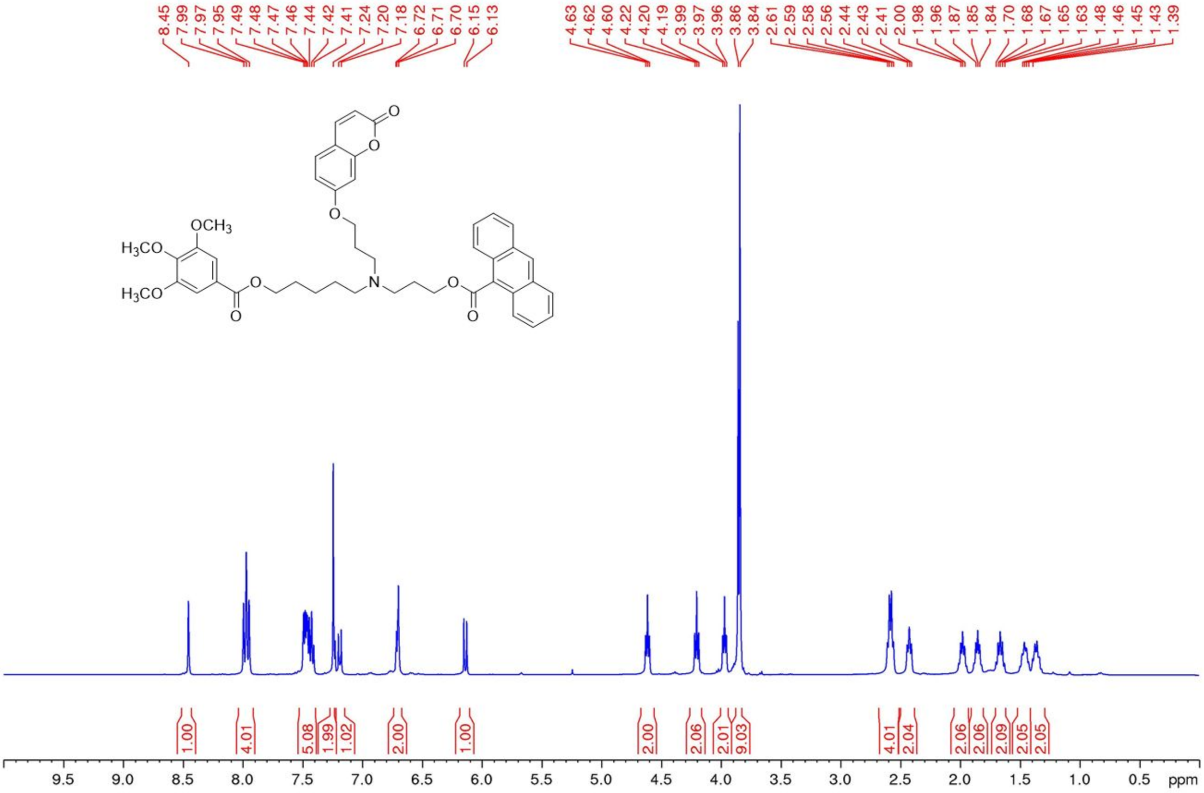

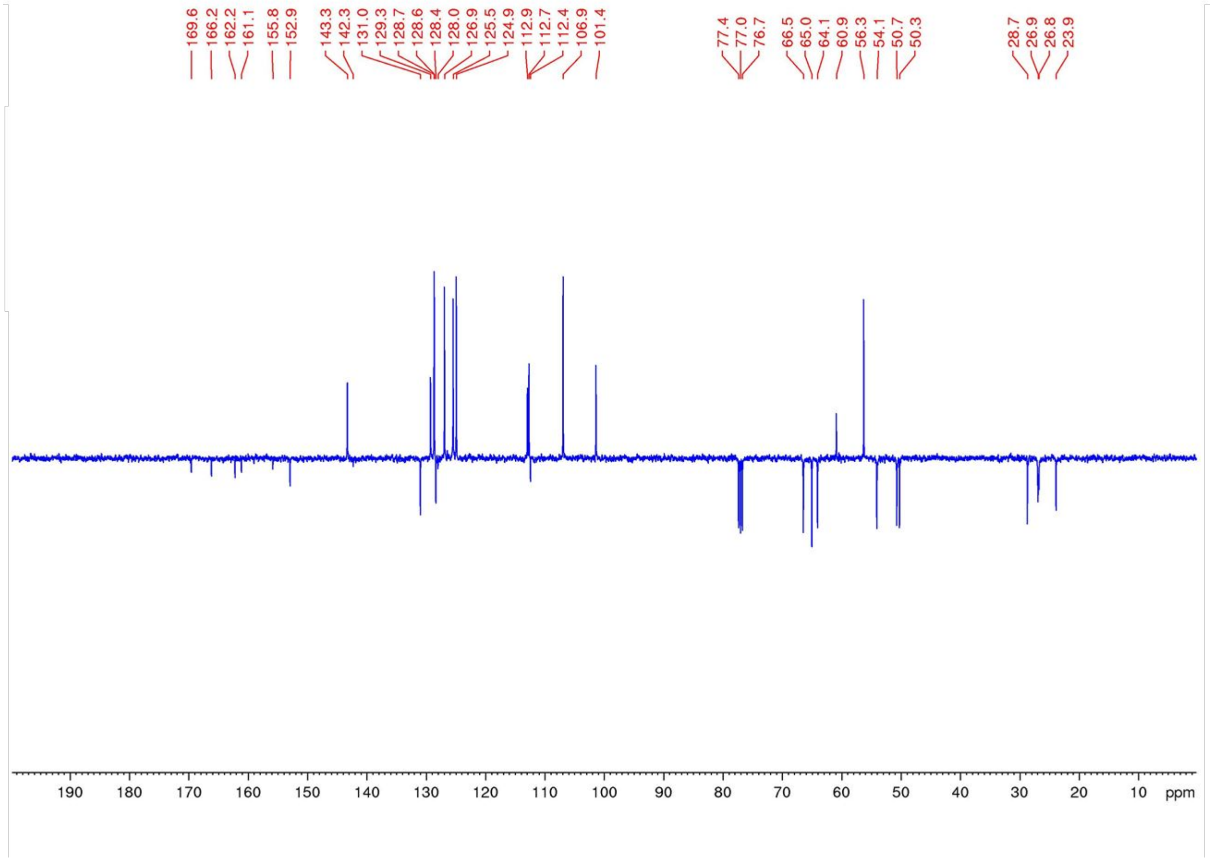

^{1}H-NMR spectrum of compound **7**

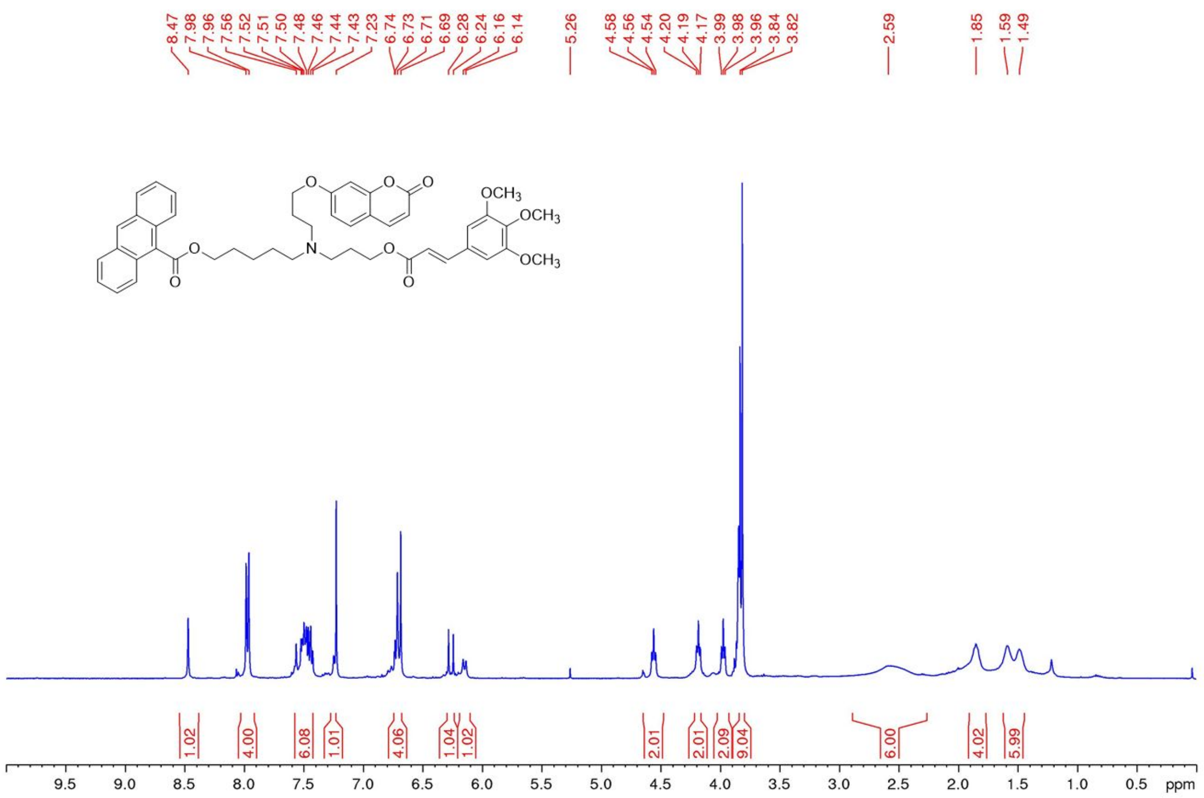

^{1}H-NMR and ^{13}C-APT-NMR spectra of compound **8**

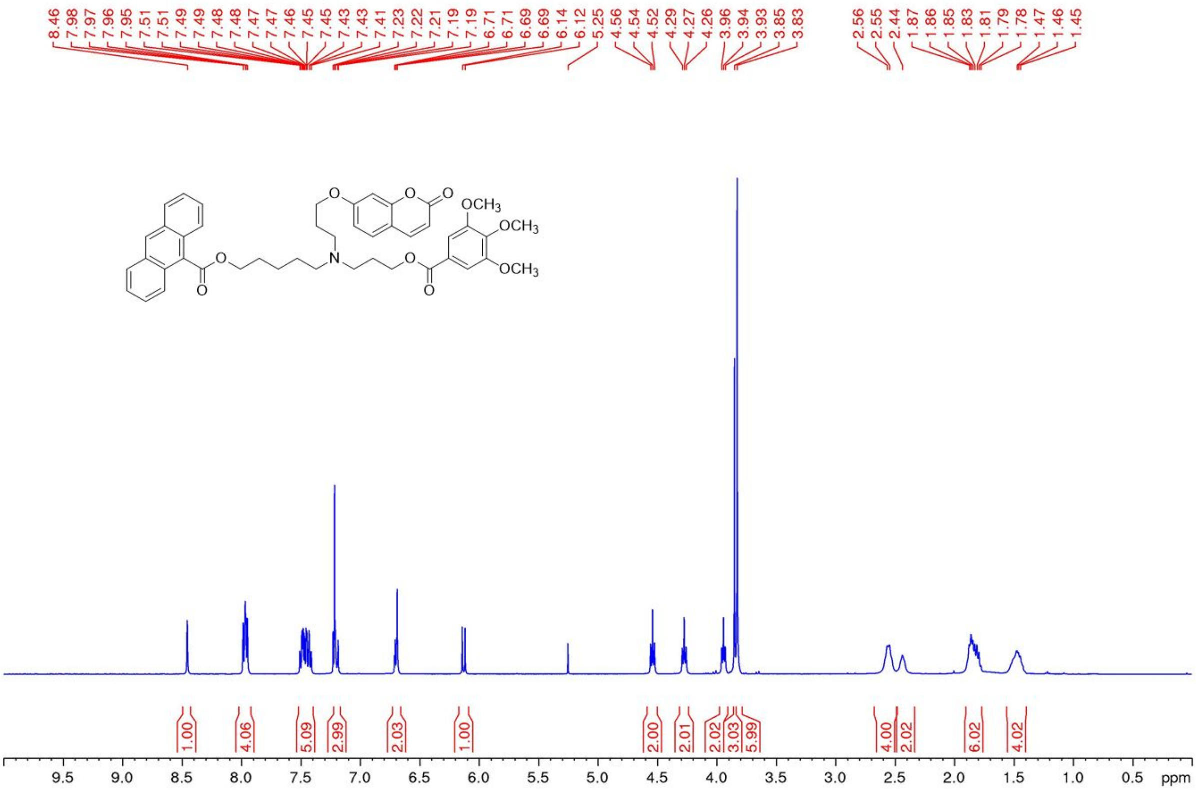

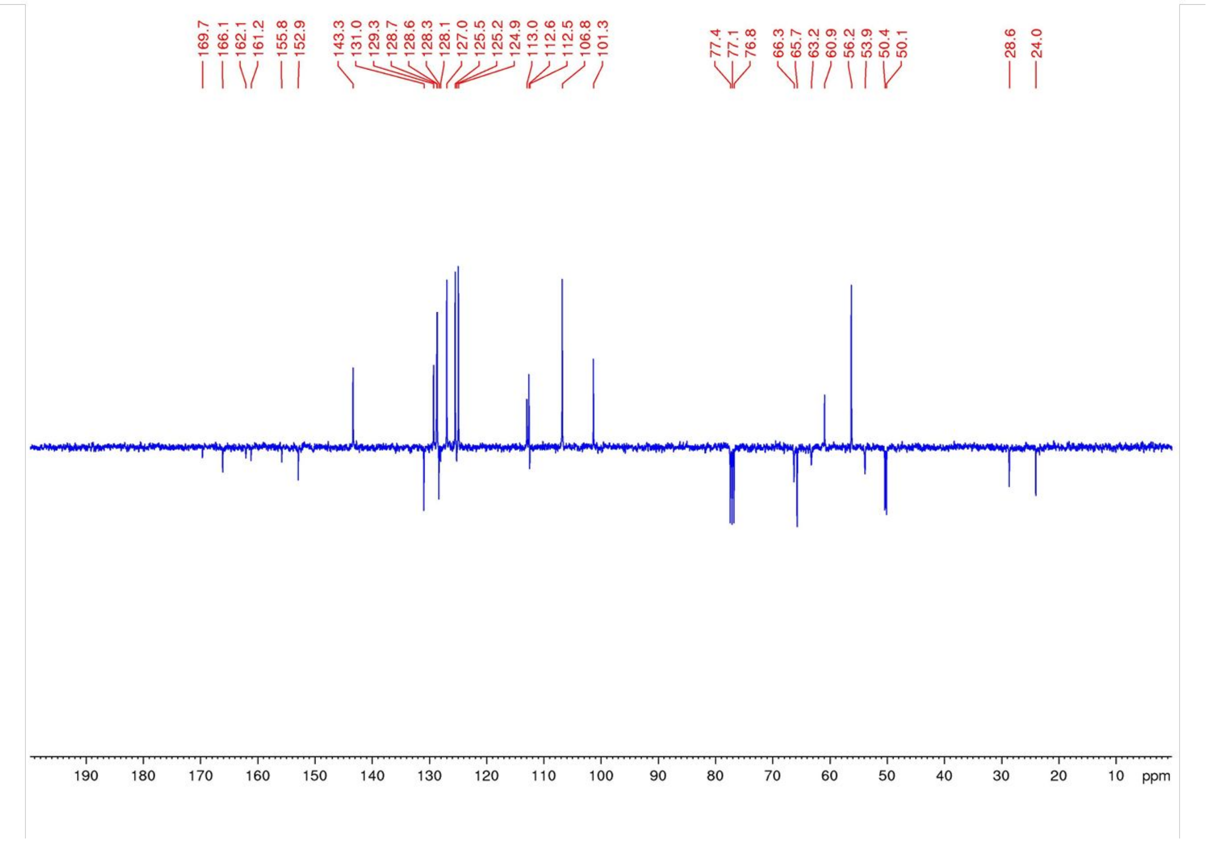

^{1}H-NMR and ^{13}C-APT-NMR spectra of compound **9**

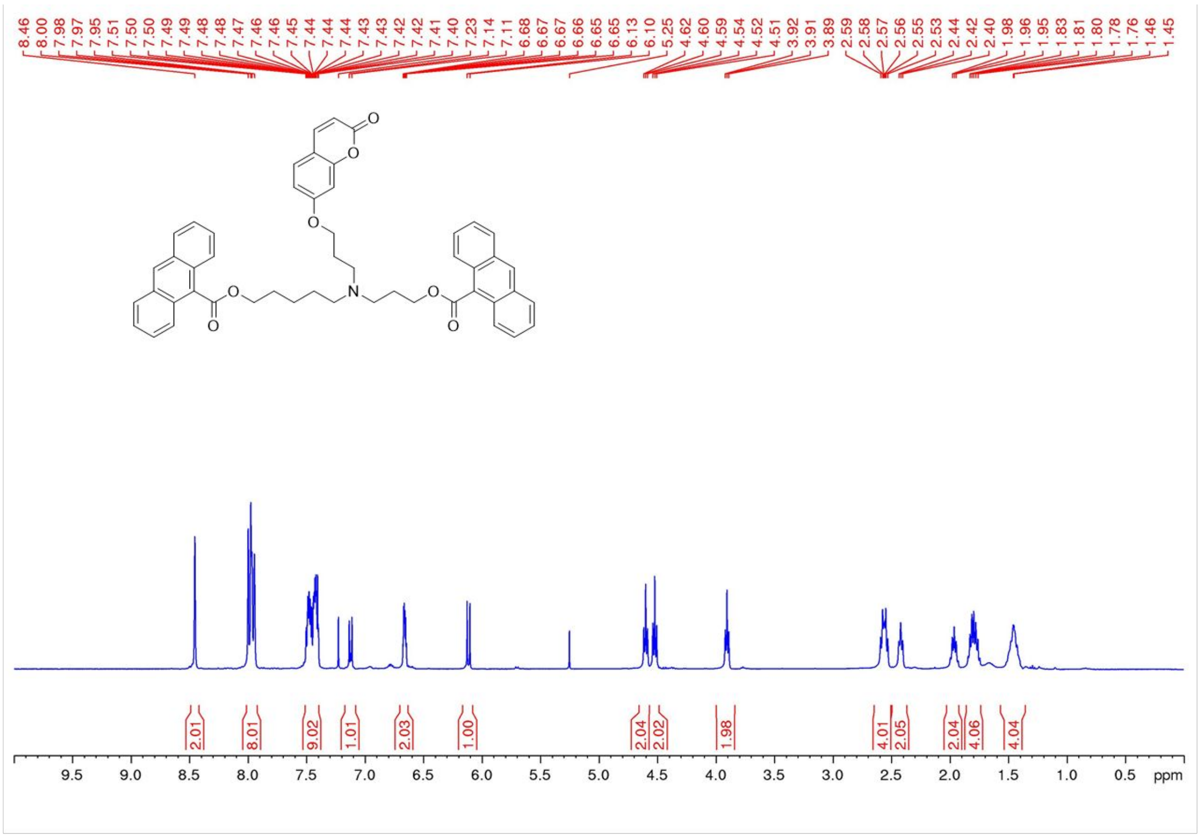

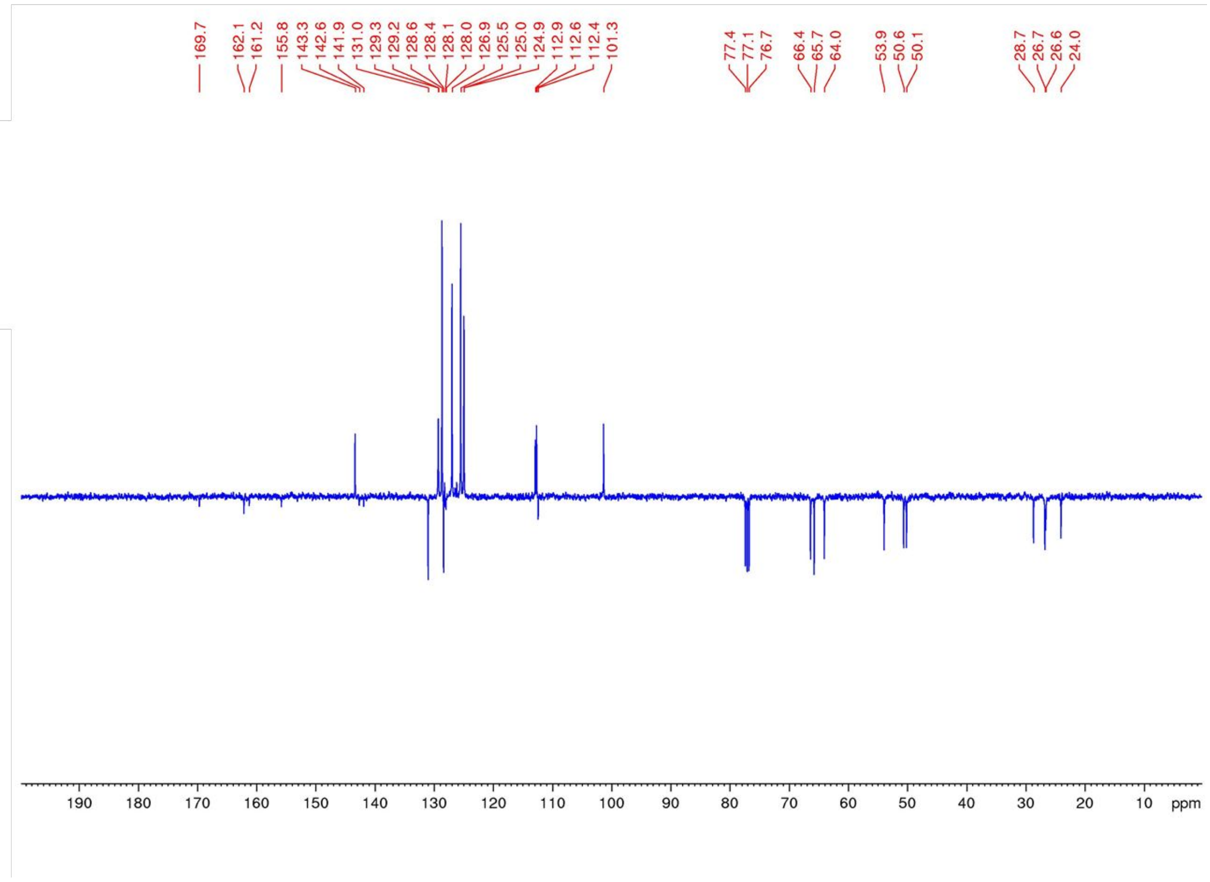

^{1}H-NMR and ^{13}C-APT-NMR spectra of compound **10**

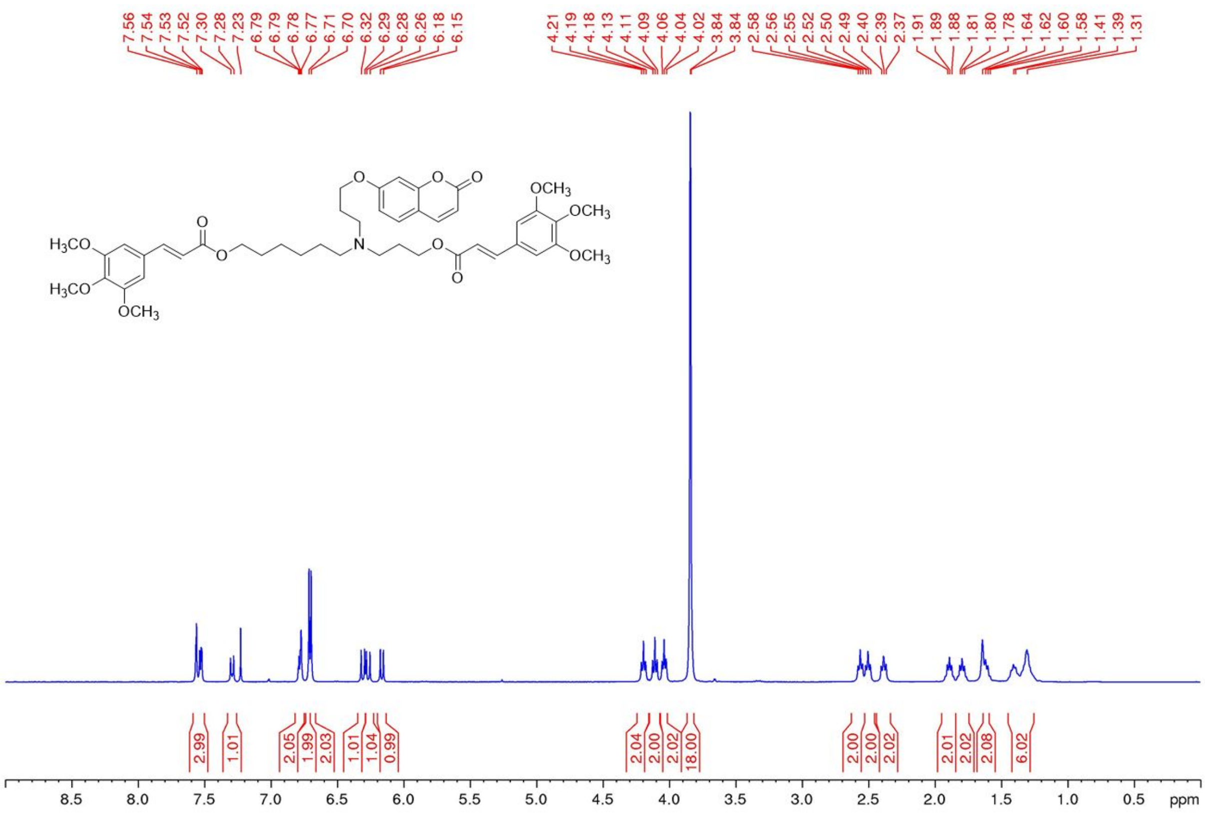

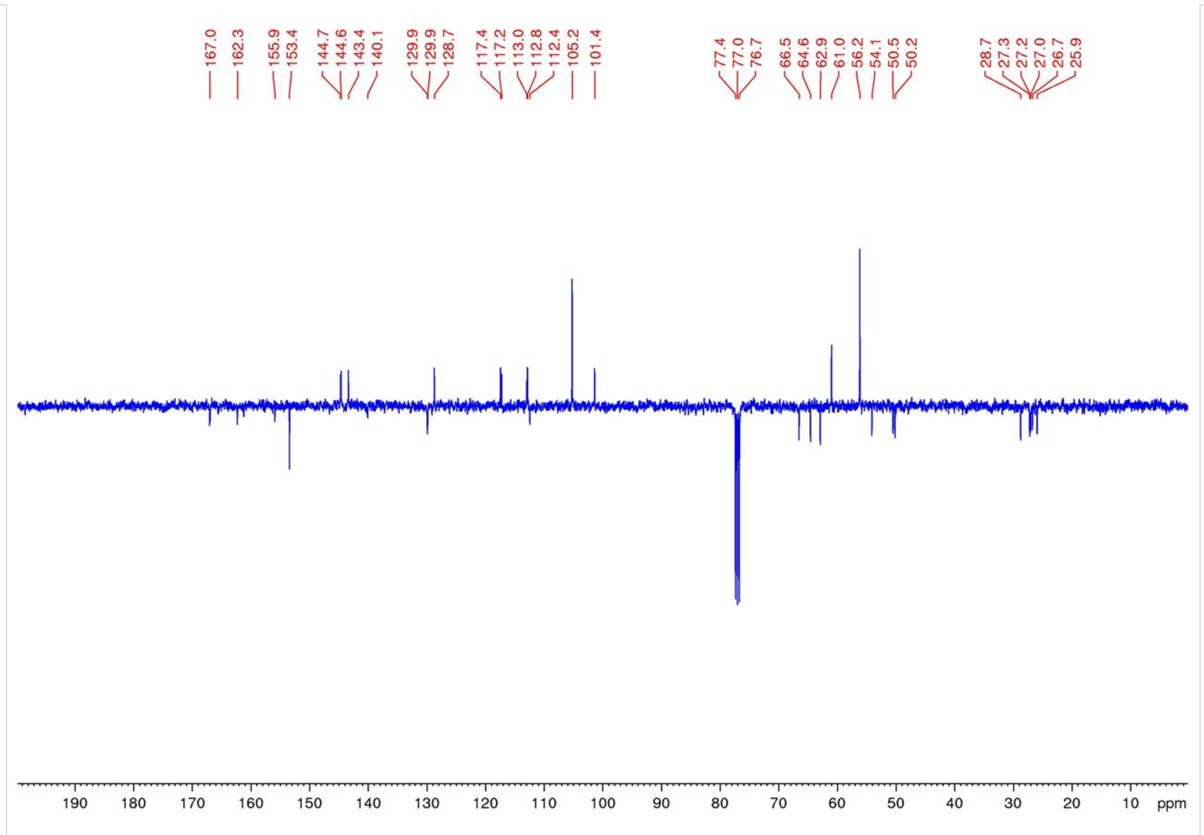

^{1}H-NMR and ^{13}C-APT-NMR spectra of compound **11**

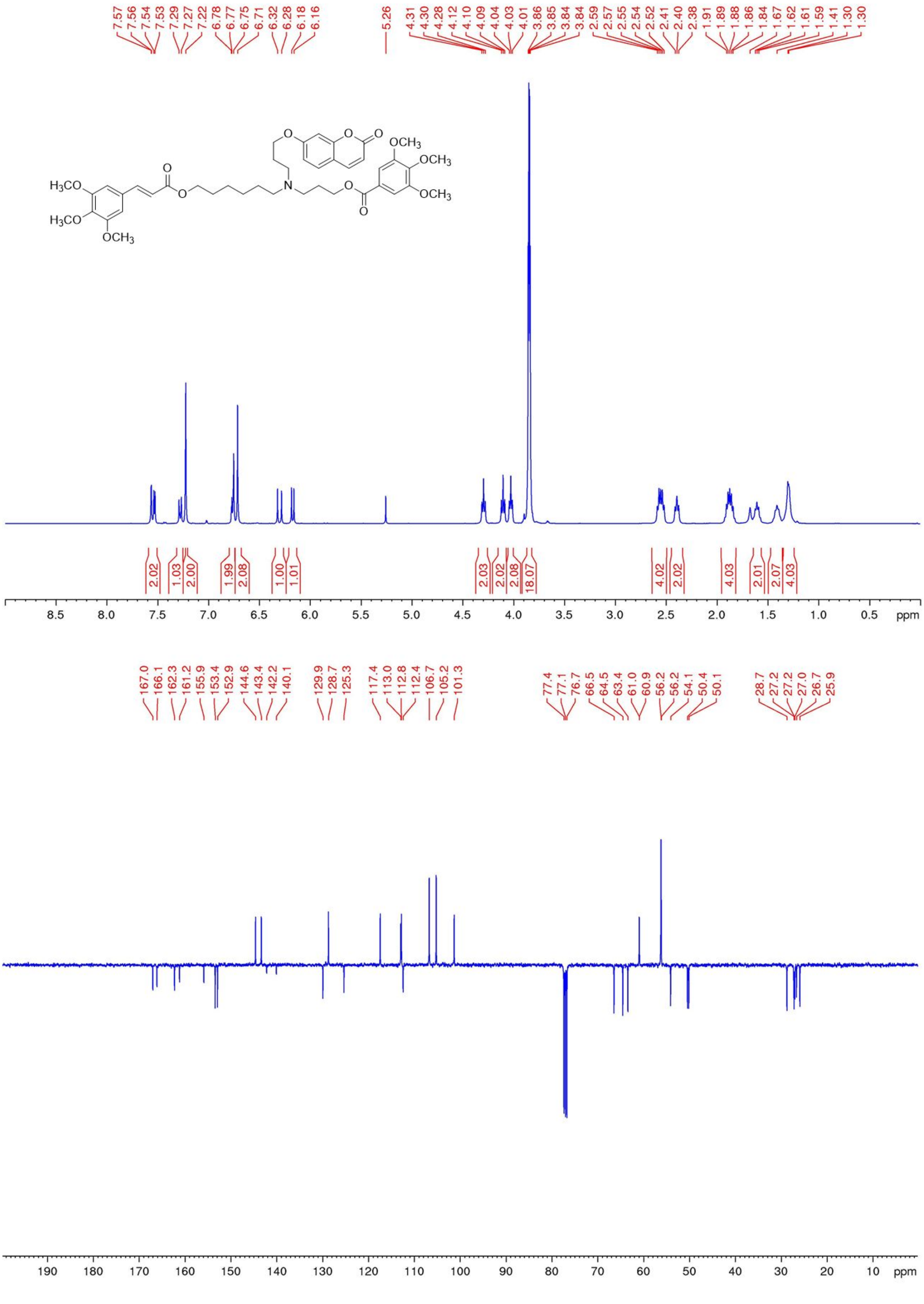

^{1}H-NMR and ^{13}C-APT-NMR spectra of compound **12**

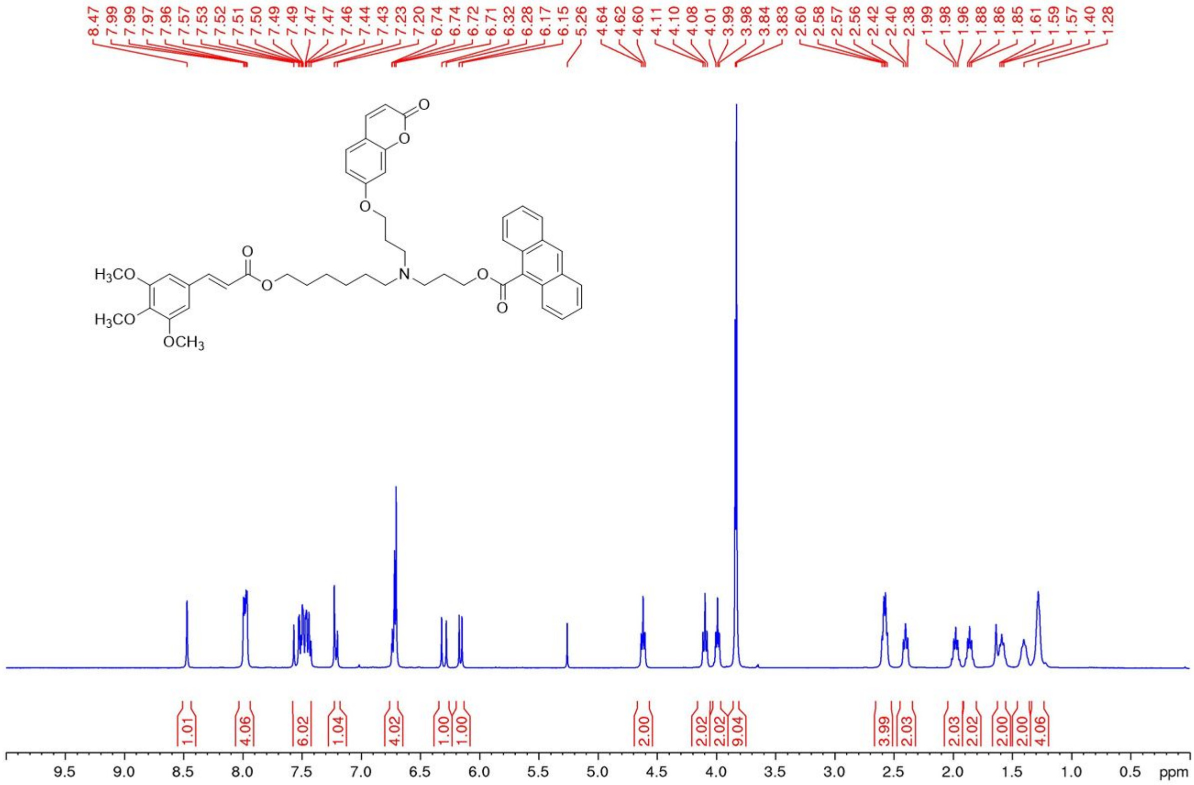

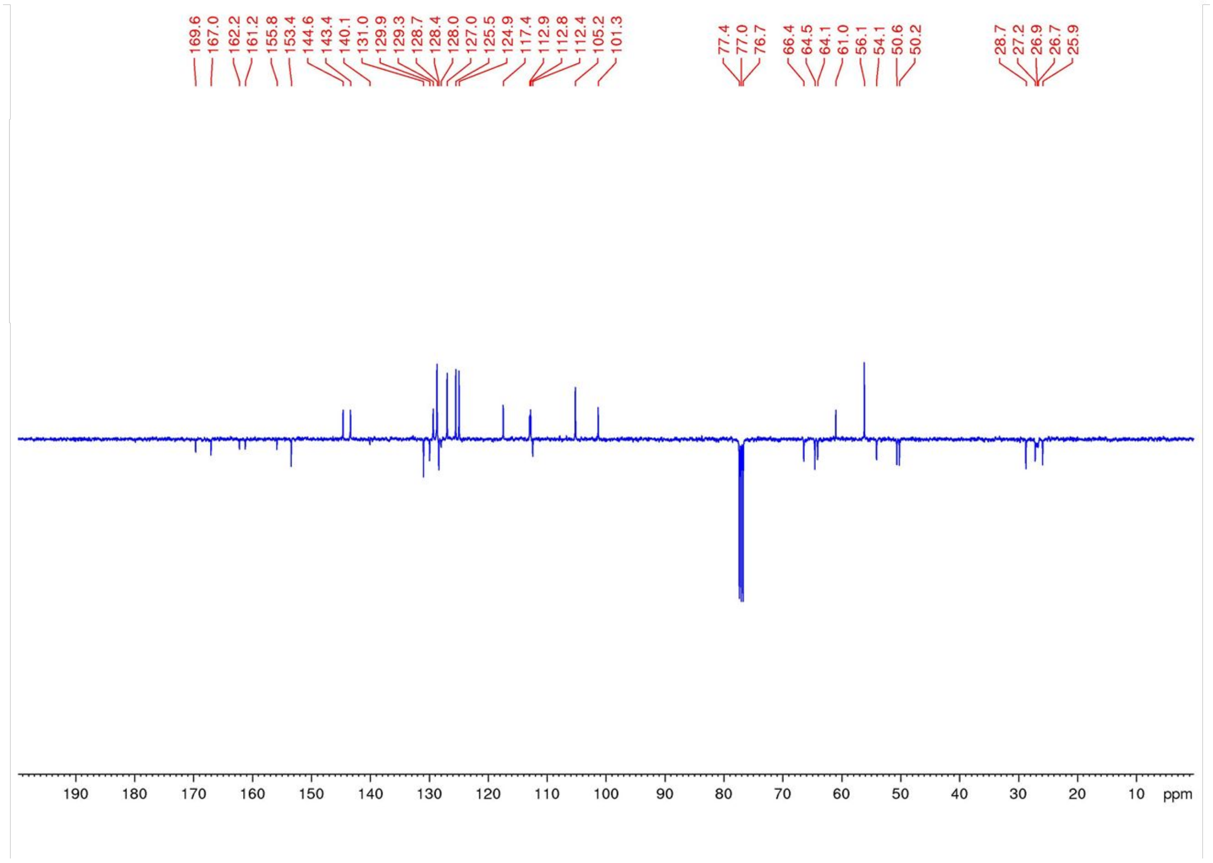

^{1}H-NMR and ^{13}C-APT-NMR spectra of compound **13**

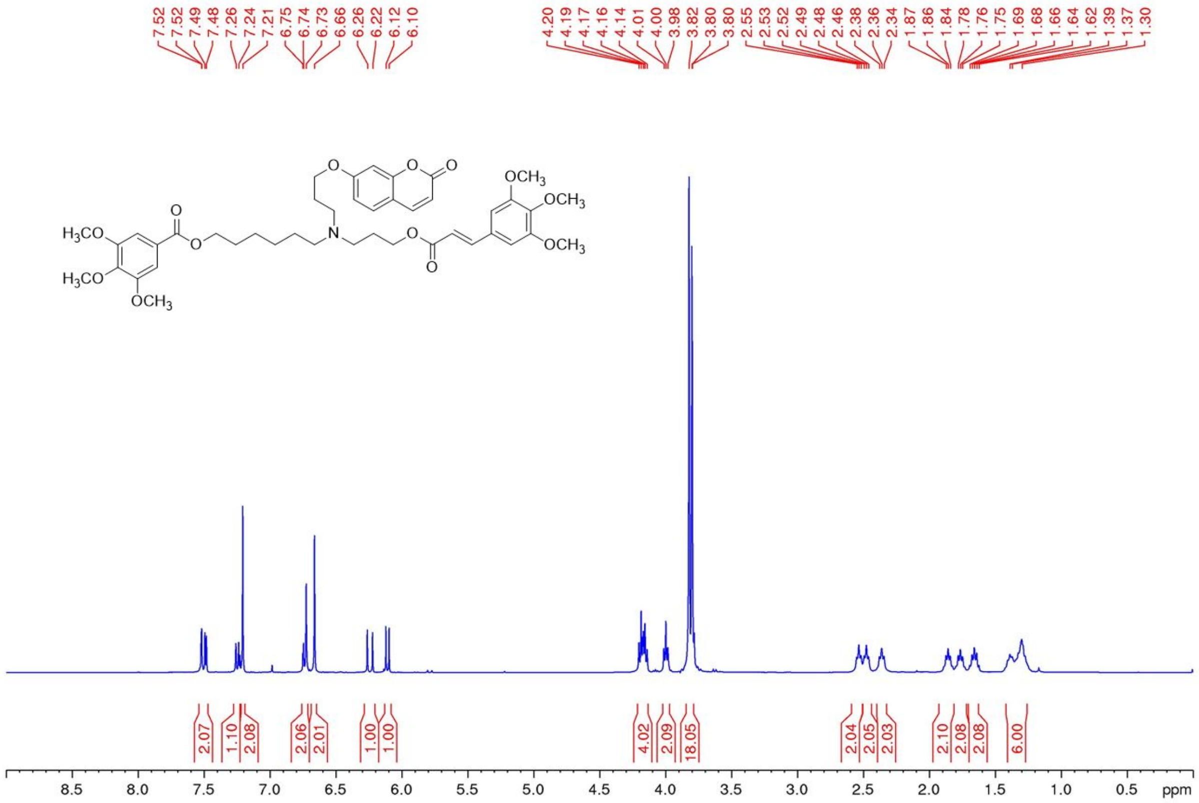

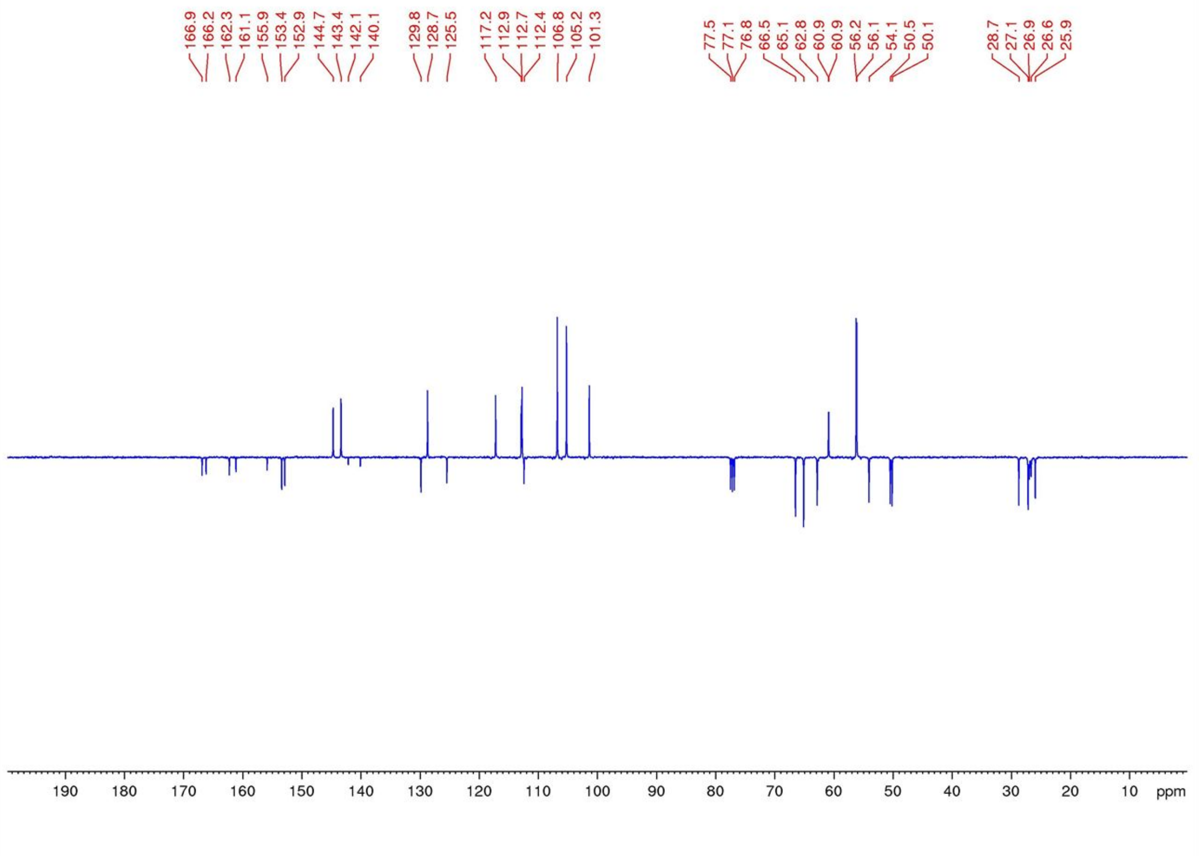

^{1}H-NMR and ^{13}C-APT-NMR spectra of compound **14**

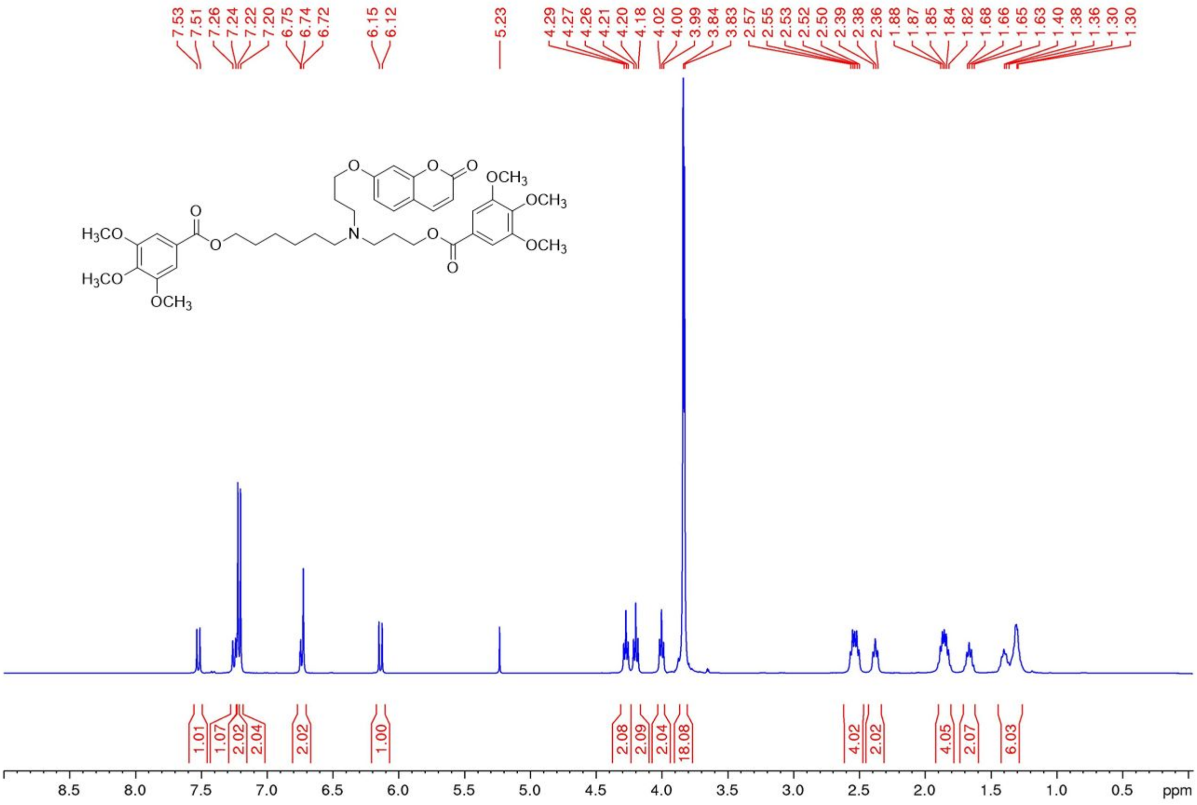

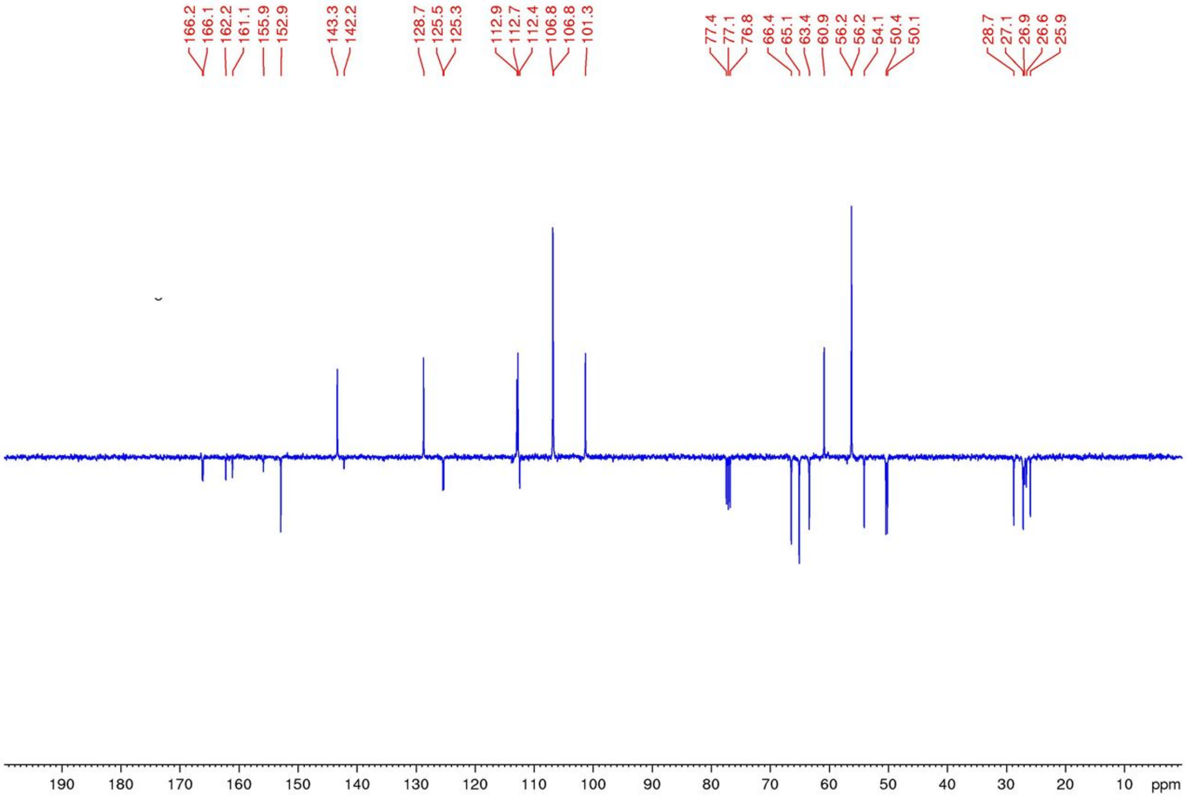

^{1}H-NMR and ^{13}C-APT-NMR spectra of compound **15**

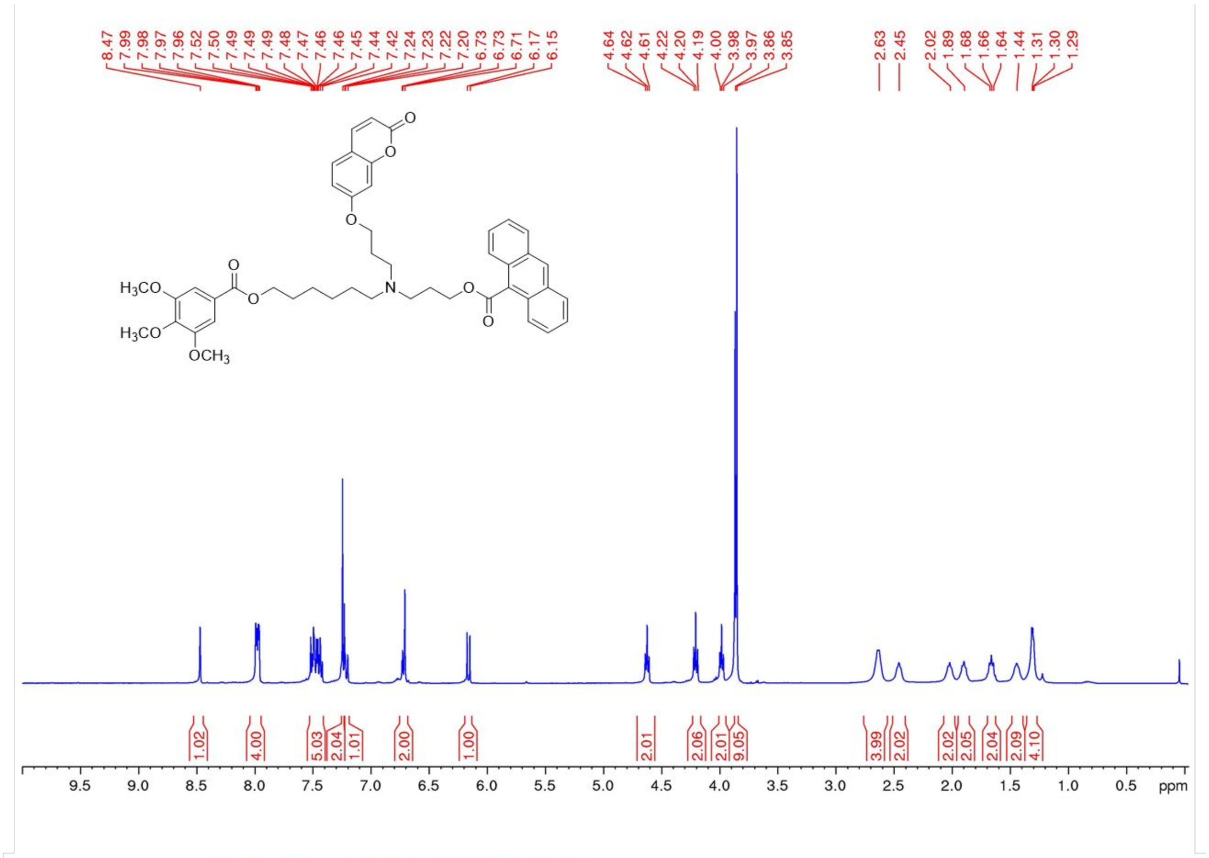

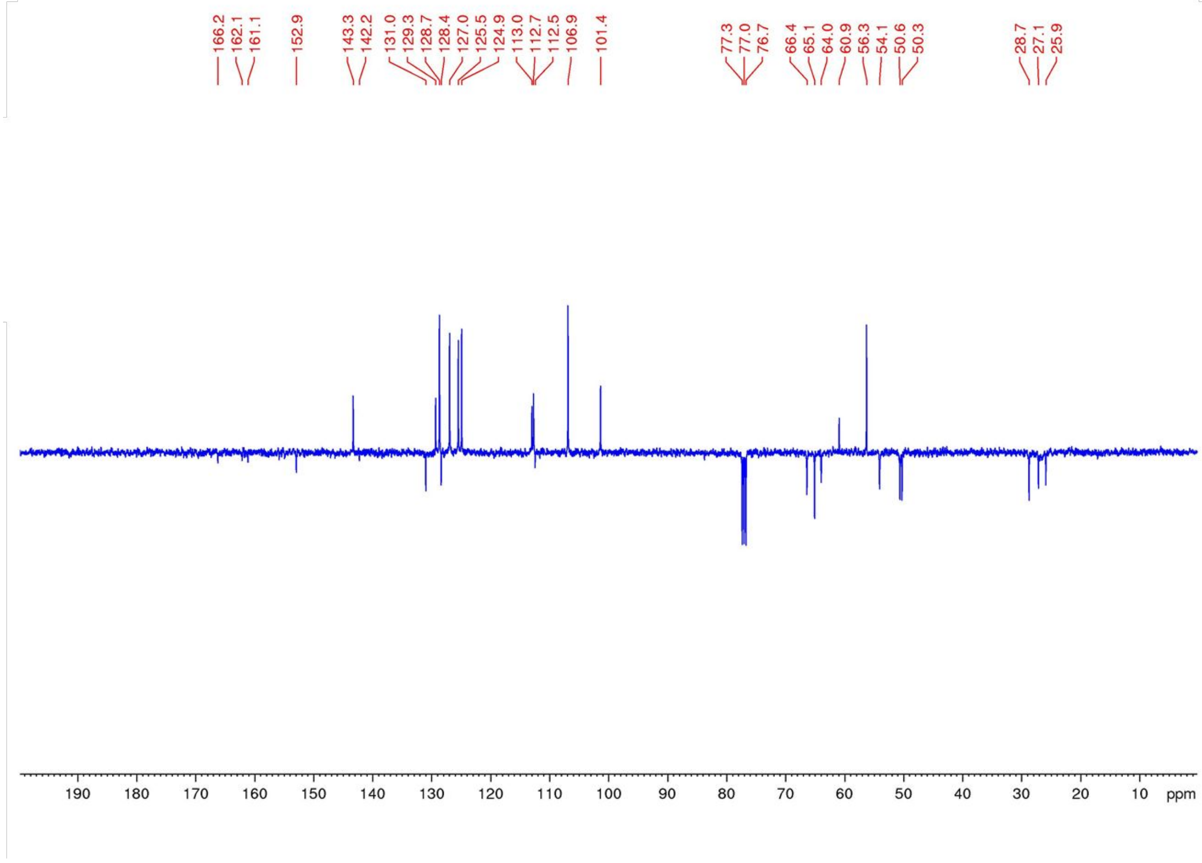

^{1}H-NMR and ^{13}C-APT-NMR spectra of compound **16**

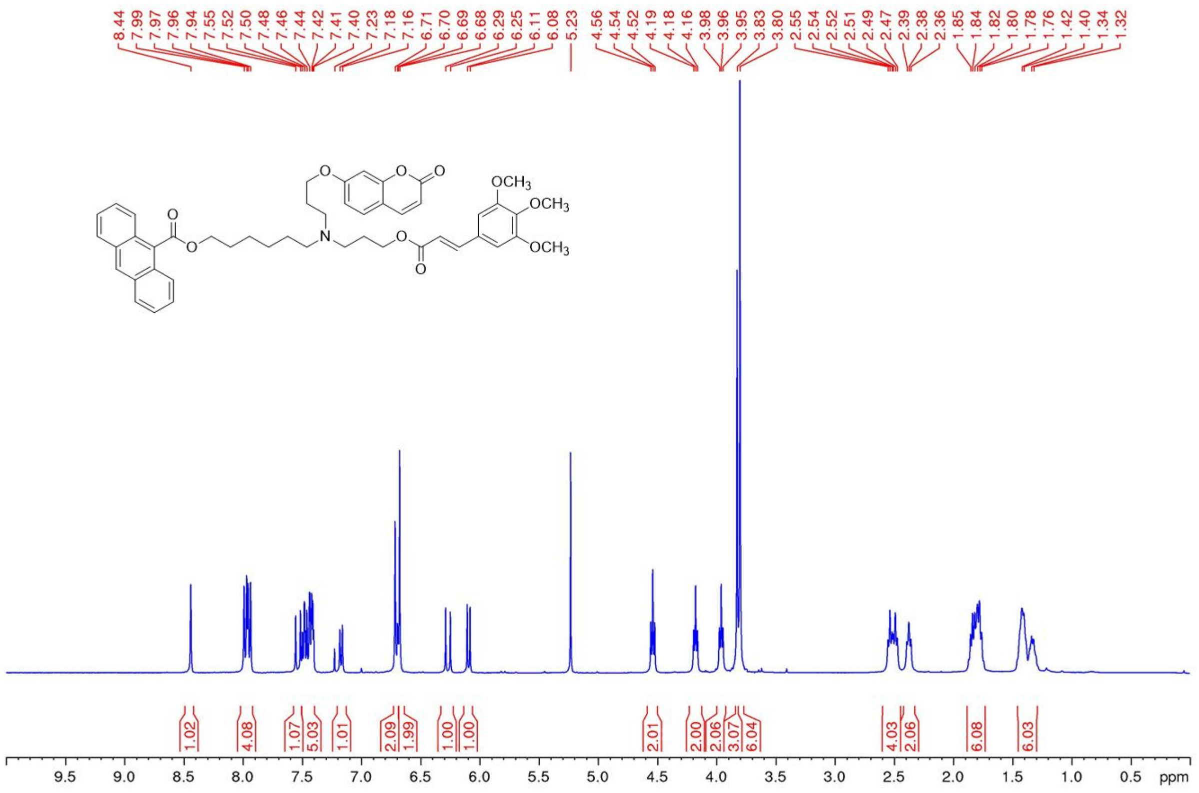

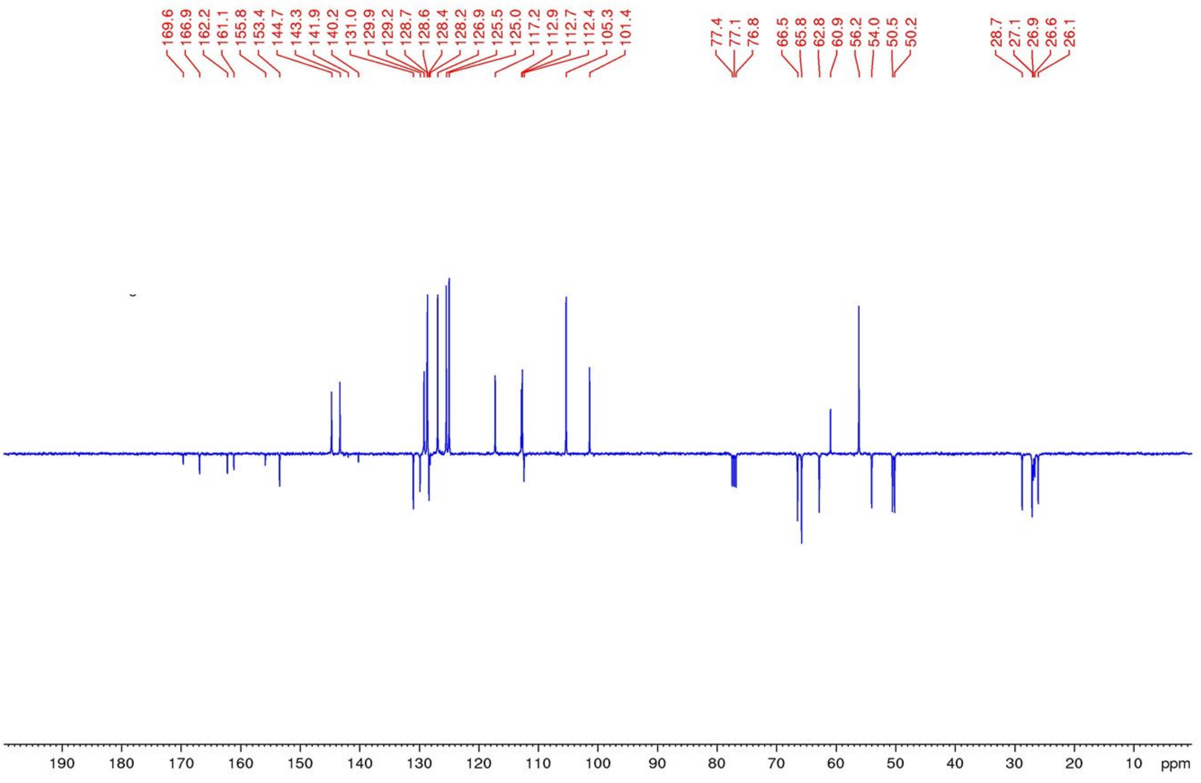

^{1}H-NMR and ^{13}C-APT-NMR spectra of compound **17**

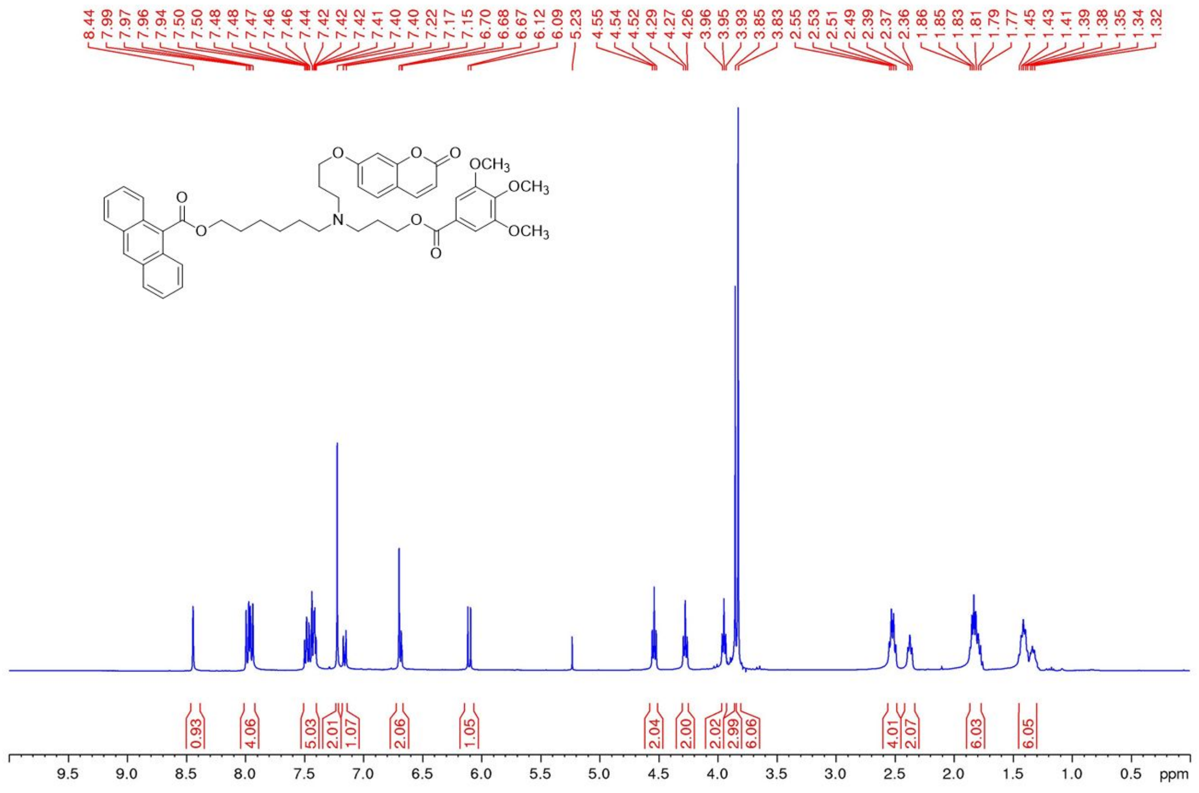

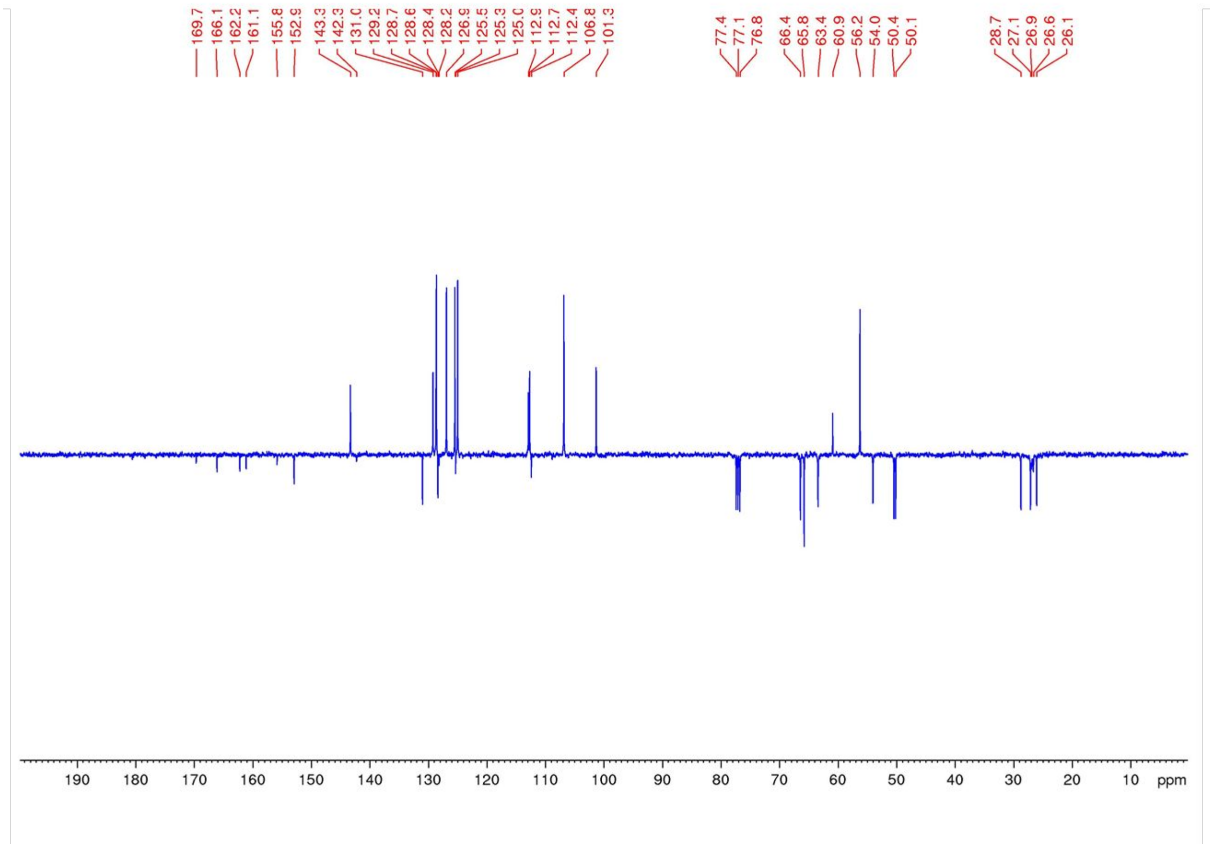

^{1}H-NMR and ^{13}C-APT-NMR spectra of compound **18**

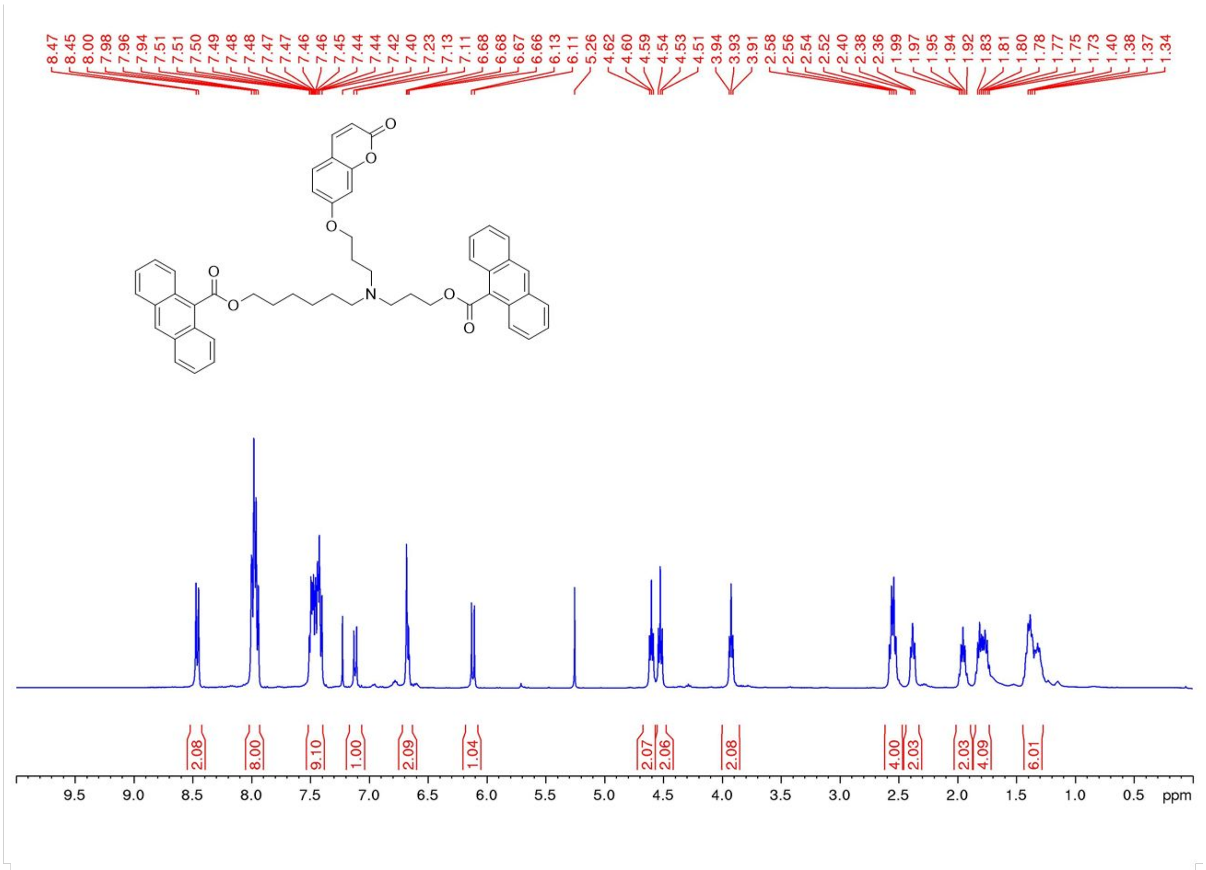

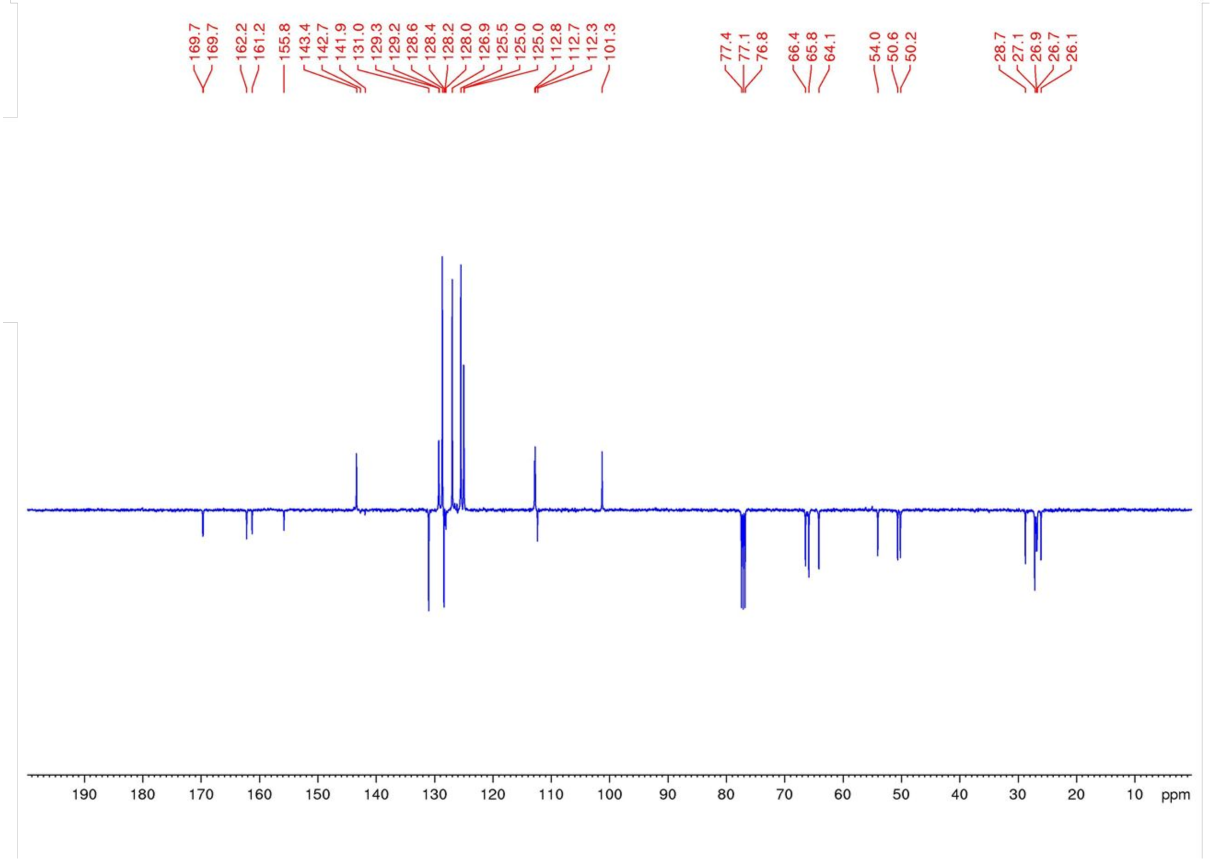

^{1}H-NMR and ^{13}C-APT-NMR spectra of compound **19**

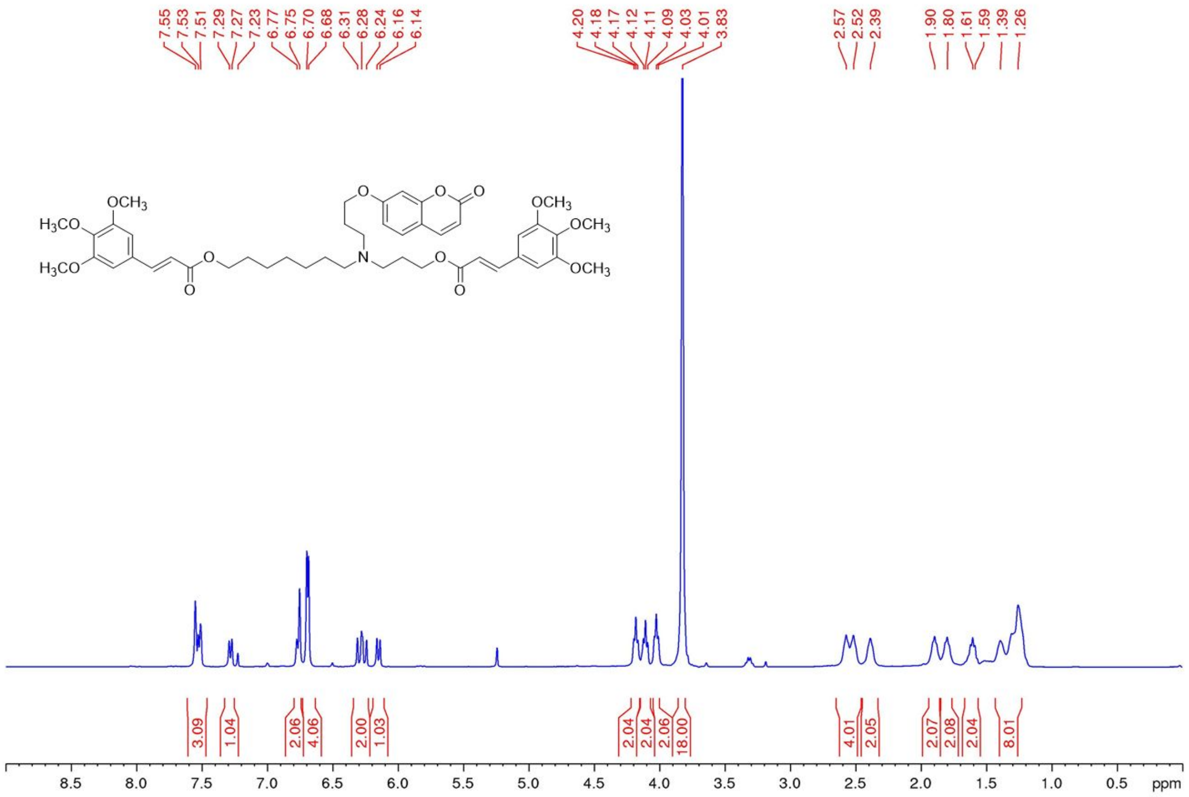

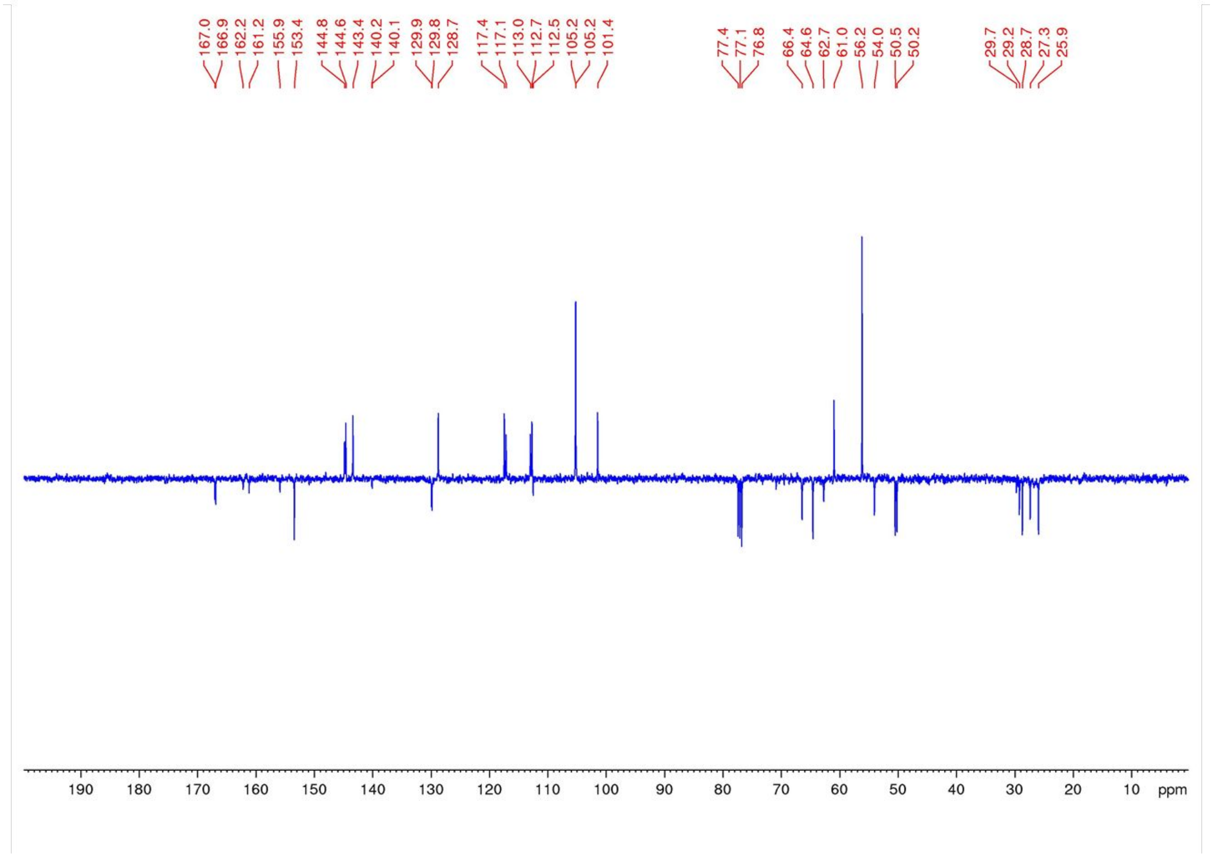

^{1}H-NMR and ^{13}C-APT-NMR spectra of compound **20**

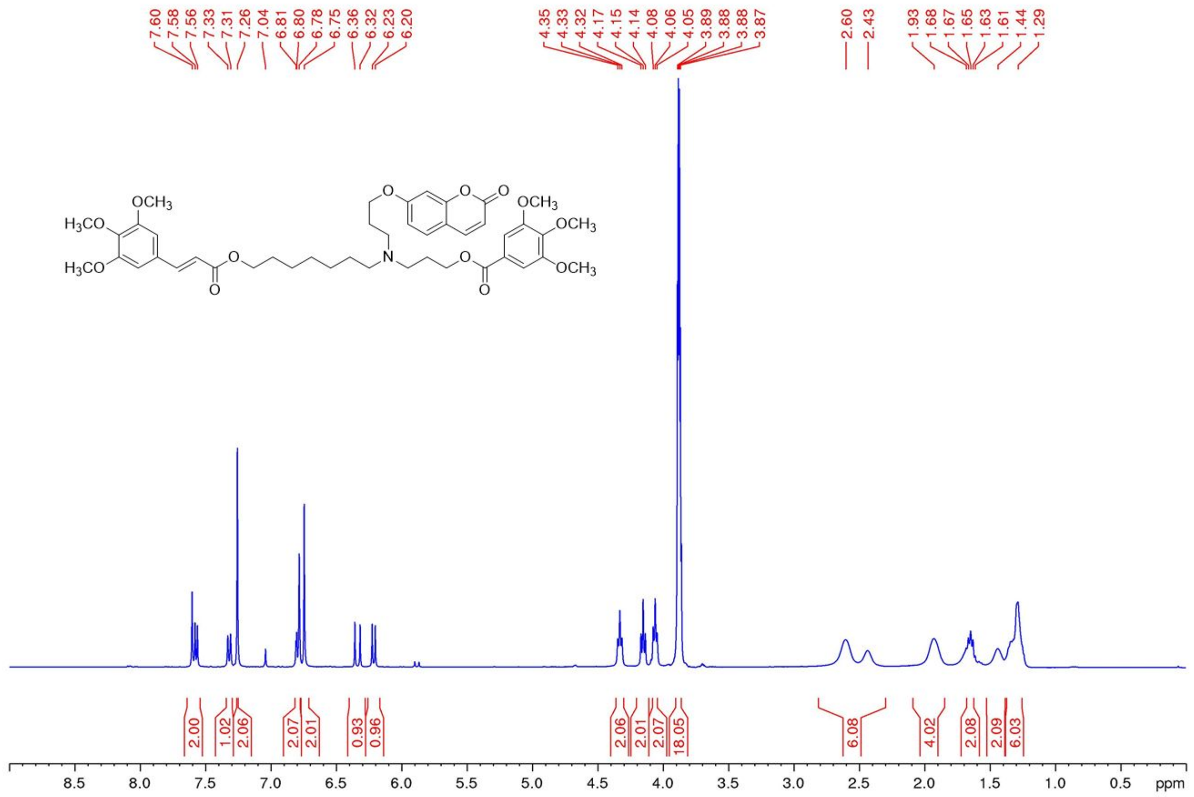

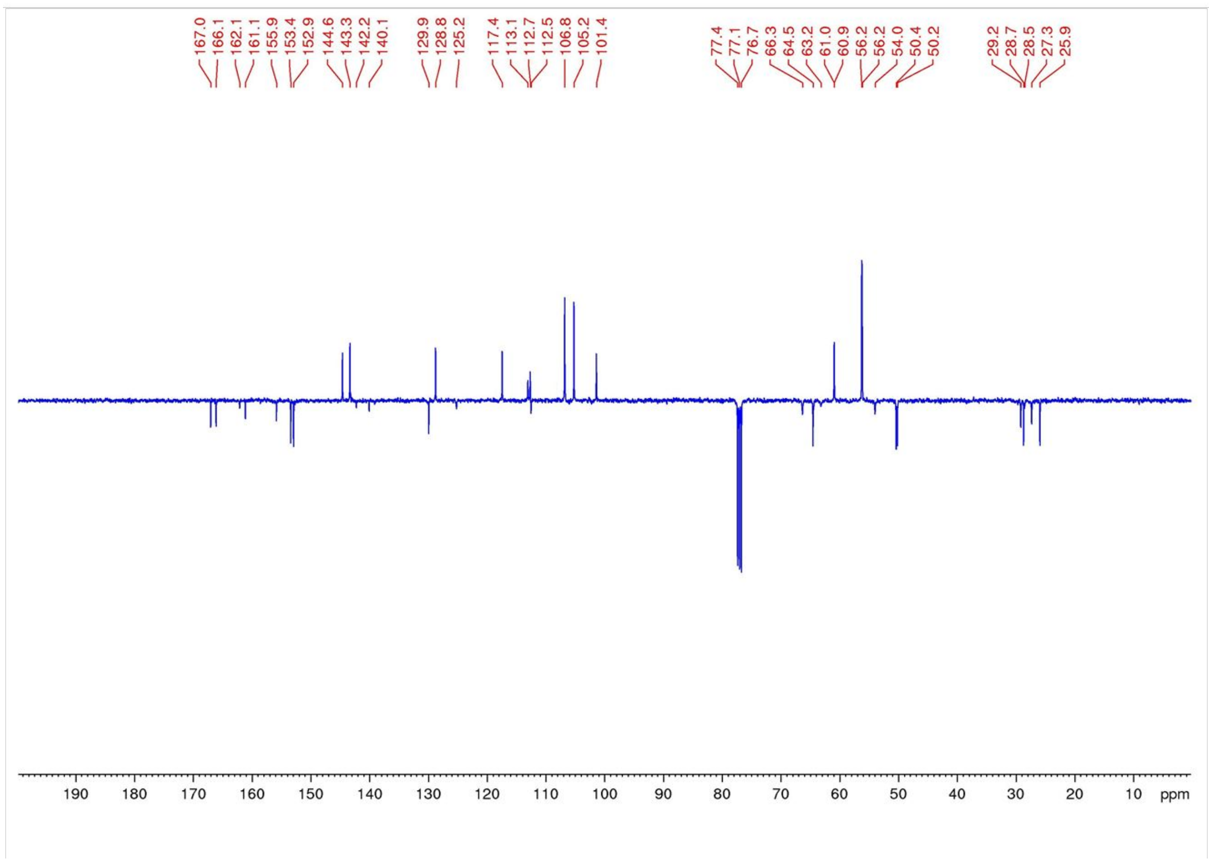

^{1}H-NMR and ^{13}C-APT-NMR spectra of compound **21**

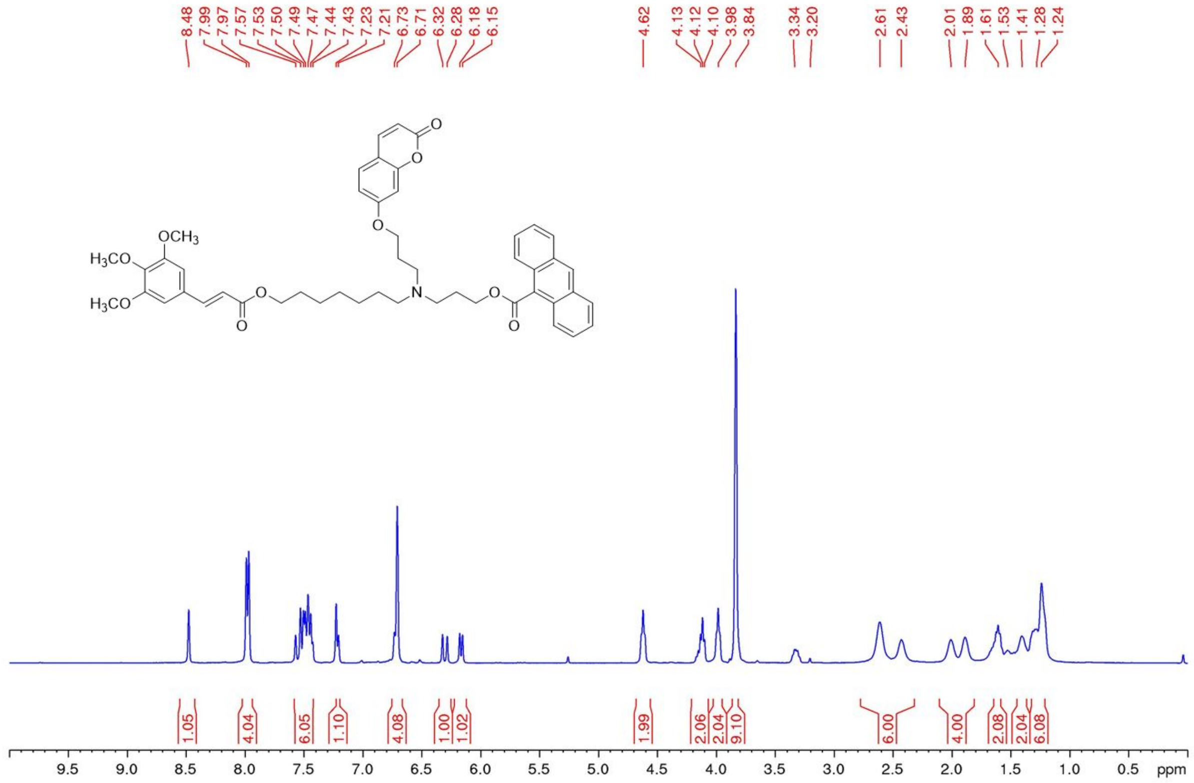

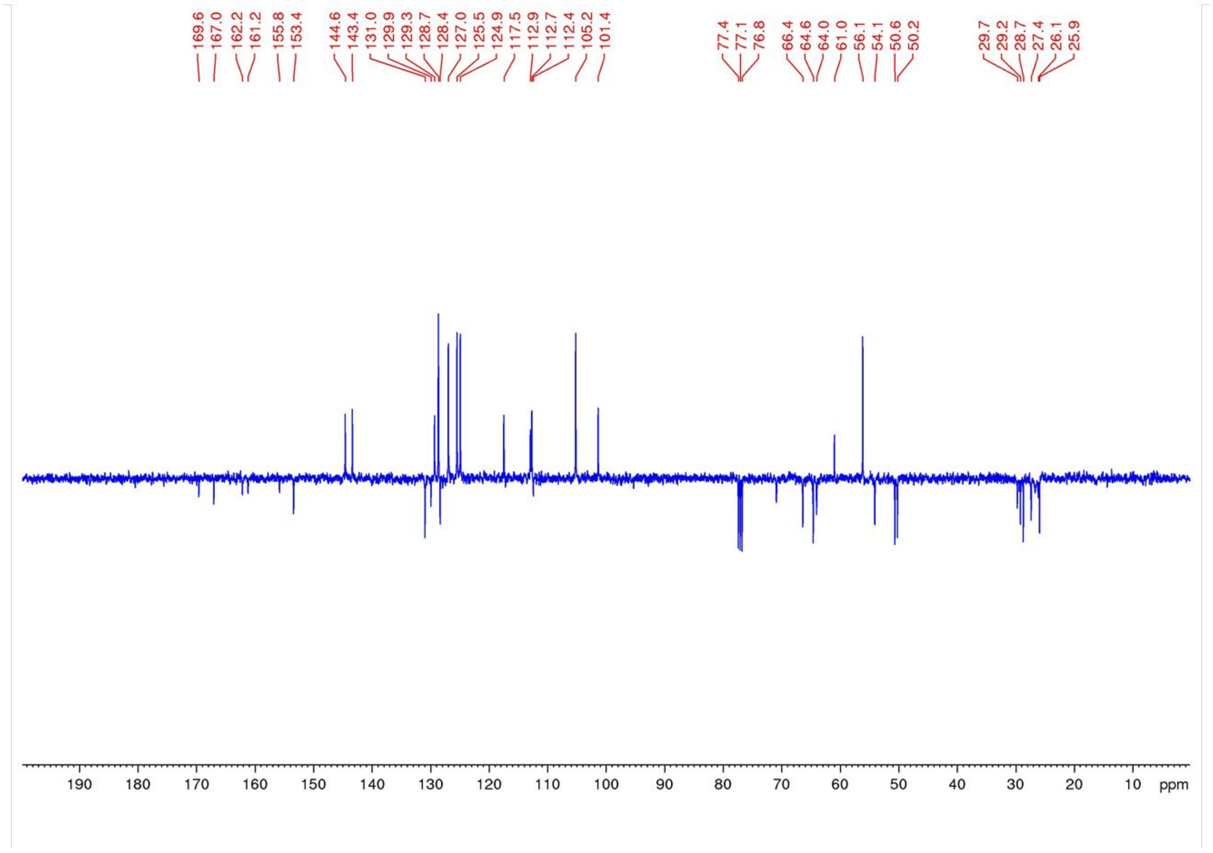

^{1}H-NMR and ^{13}C-APT-NMR spectra of compound **22**

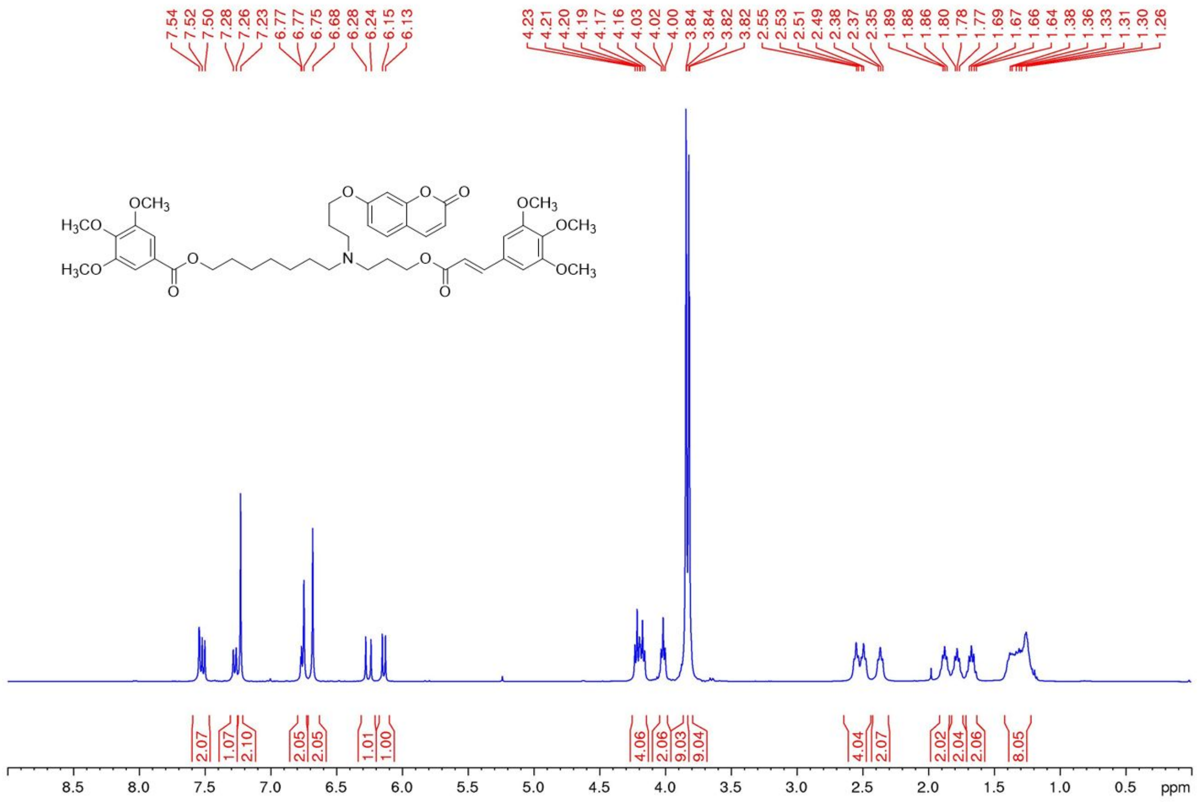

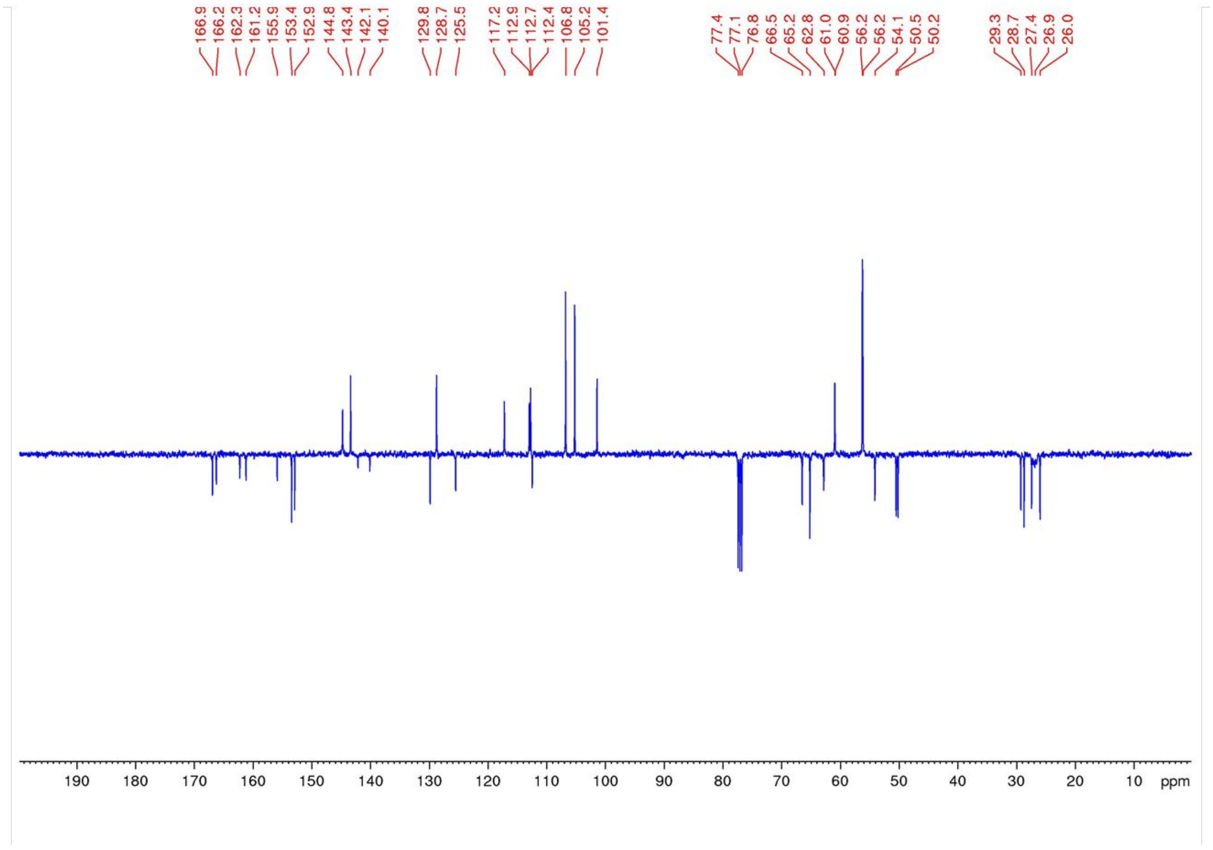

^{1}H-NMR and ^{13}C-APT-NMR spectra of compound **23**

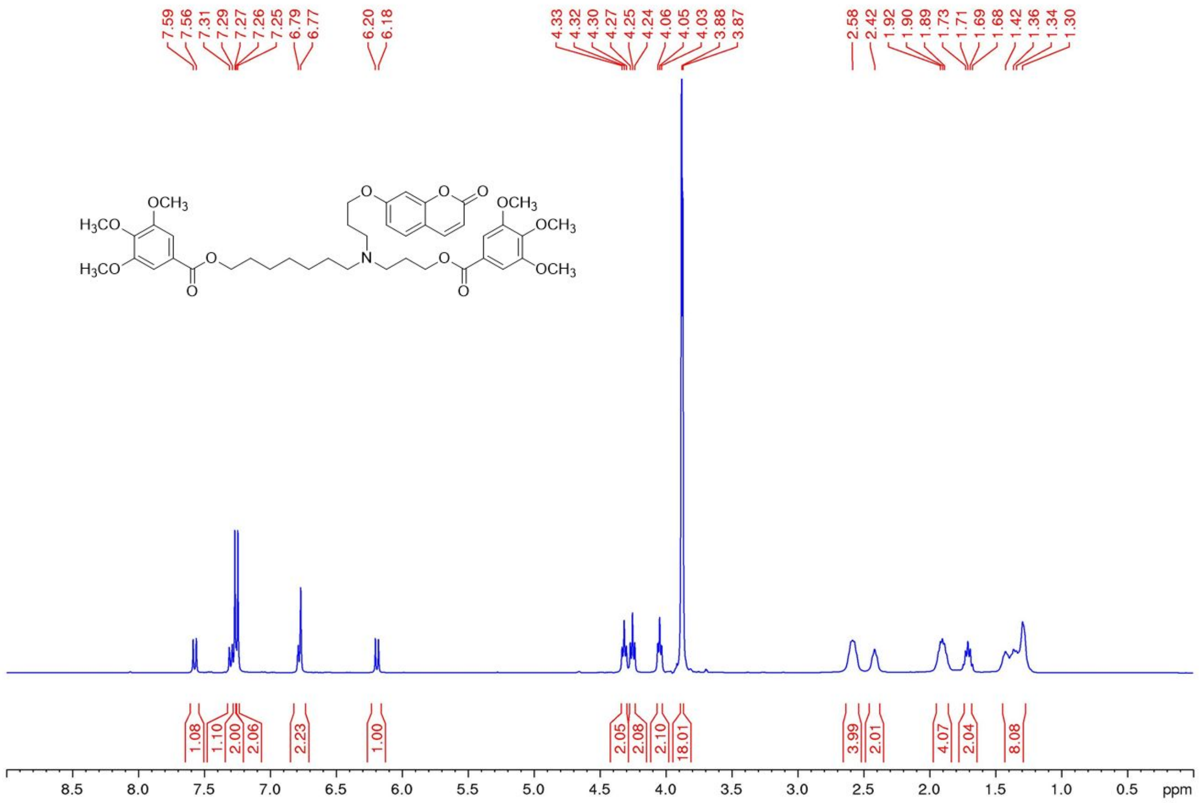

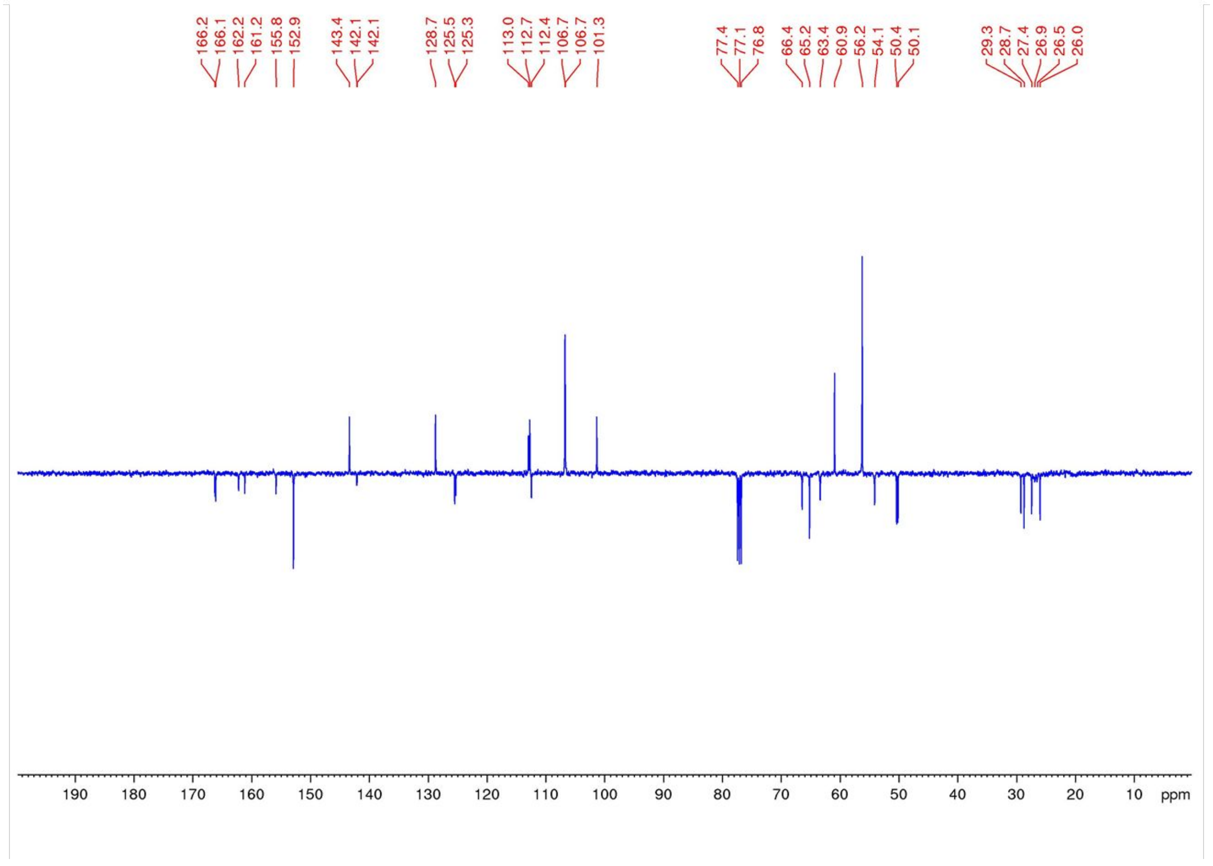

^{1}H-NMR and ^{13}C-APT-NMR spectra of compound **24**

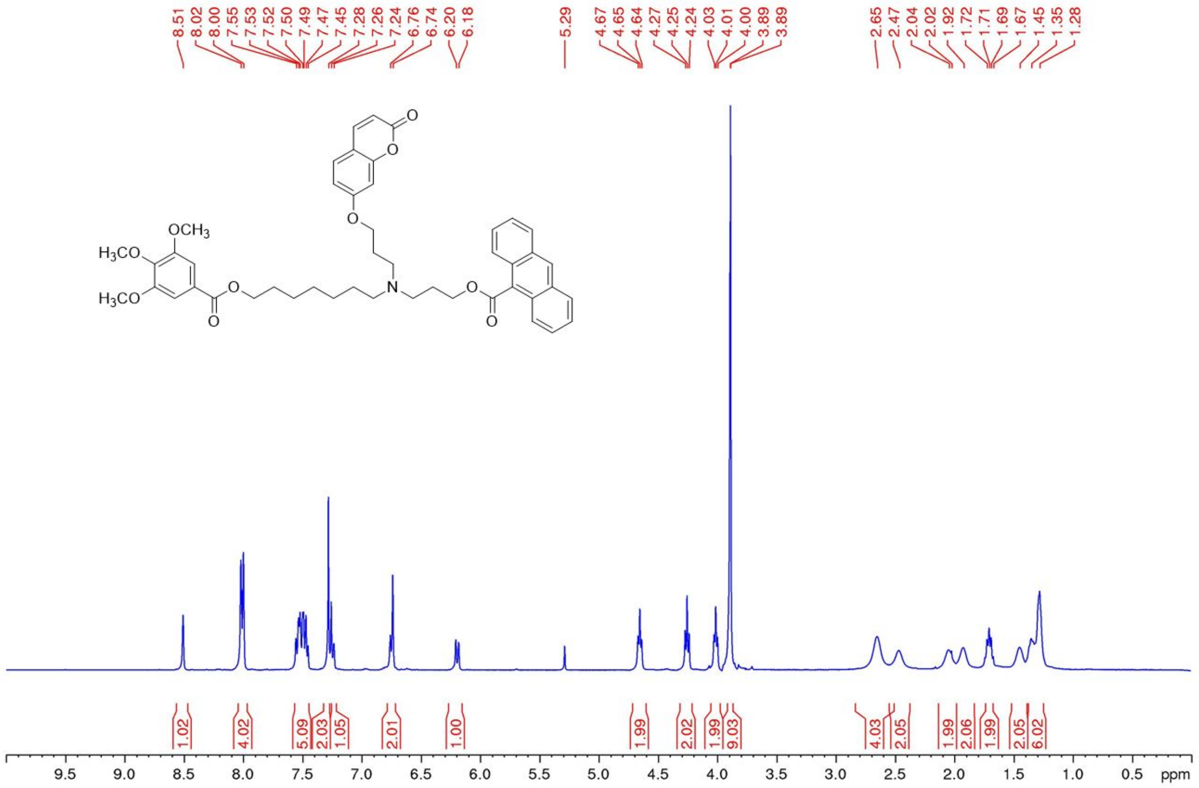

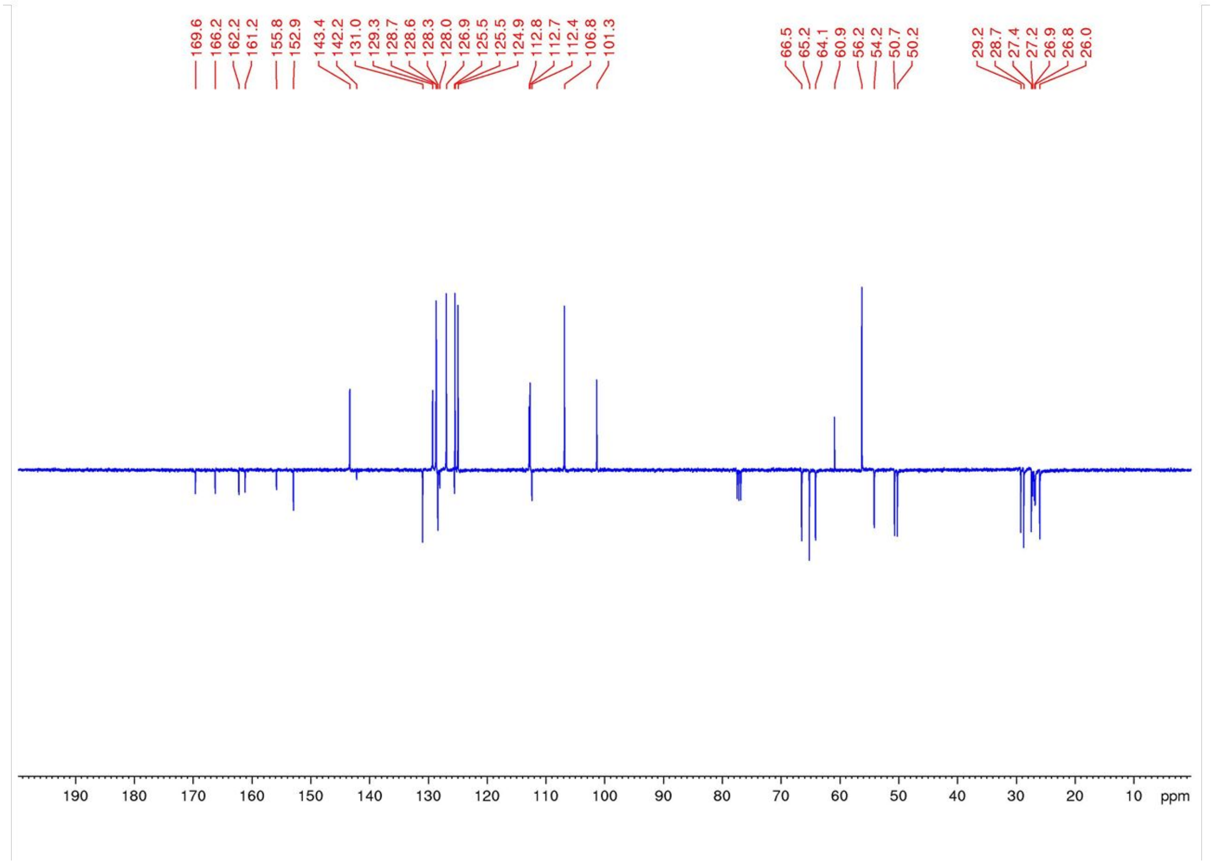

^{1}H-NMR and ^{13}C-APT-NMR spectra of compound **25**

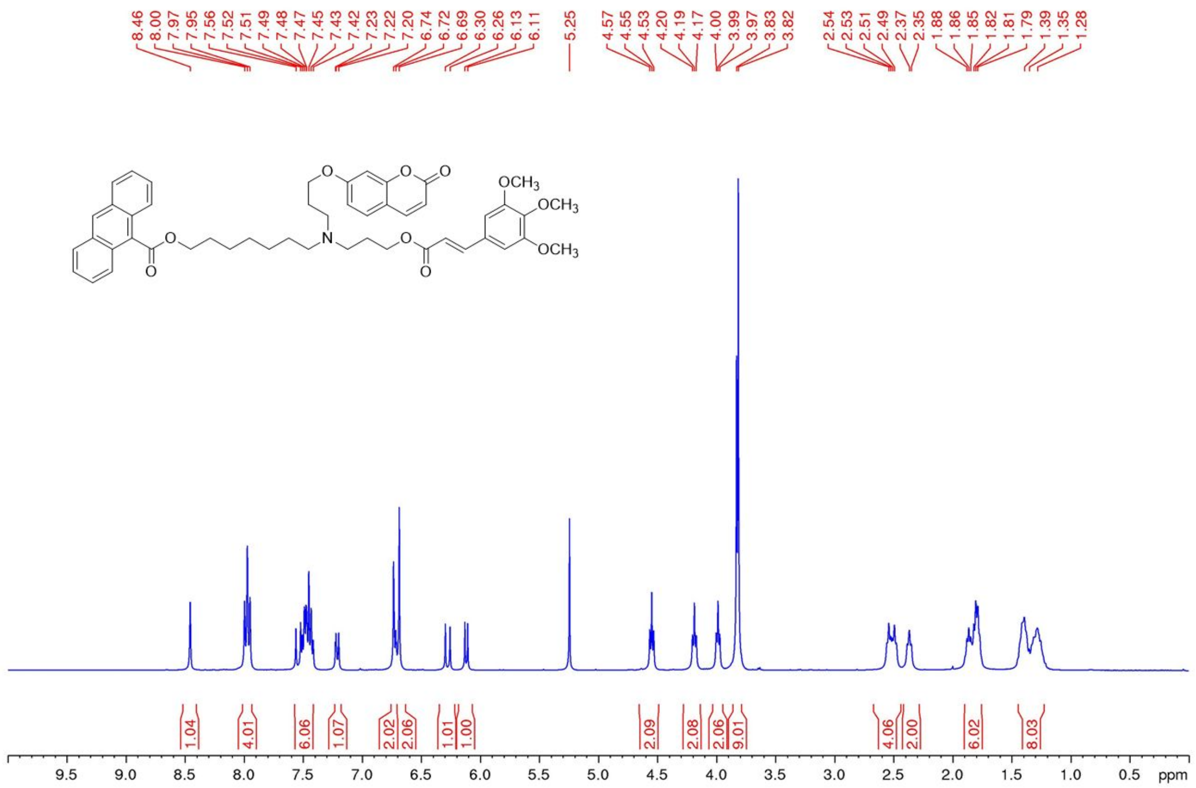

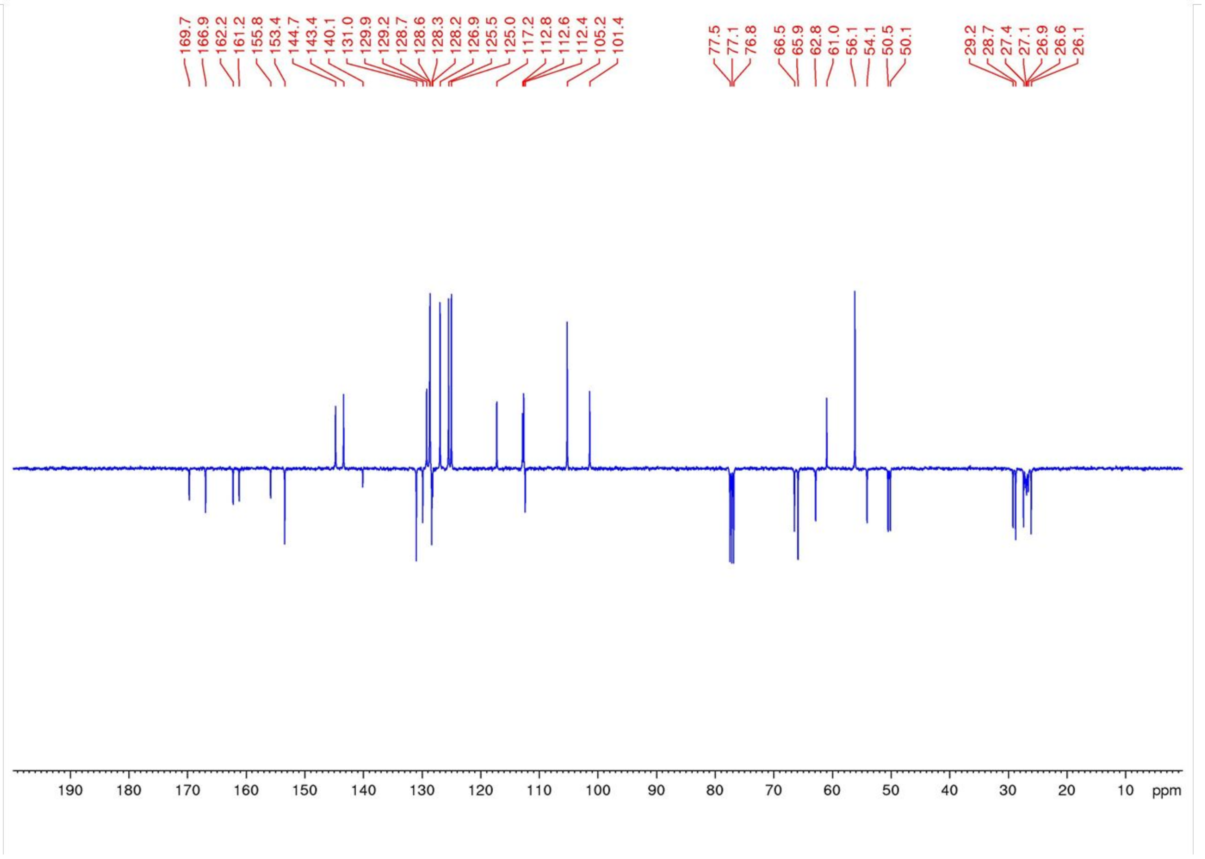

^{1}H-NMR and ^{13}C-APT-NMR spectra of compound **26**

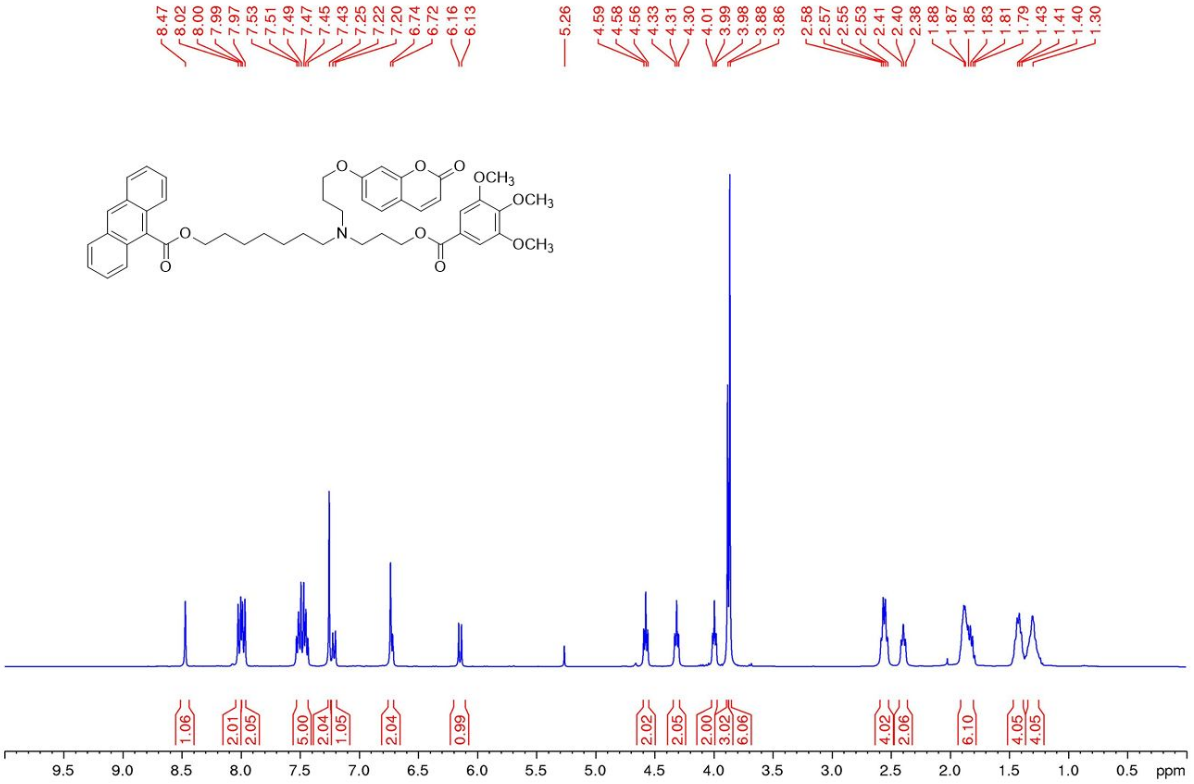

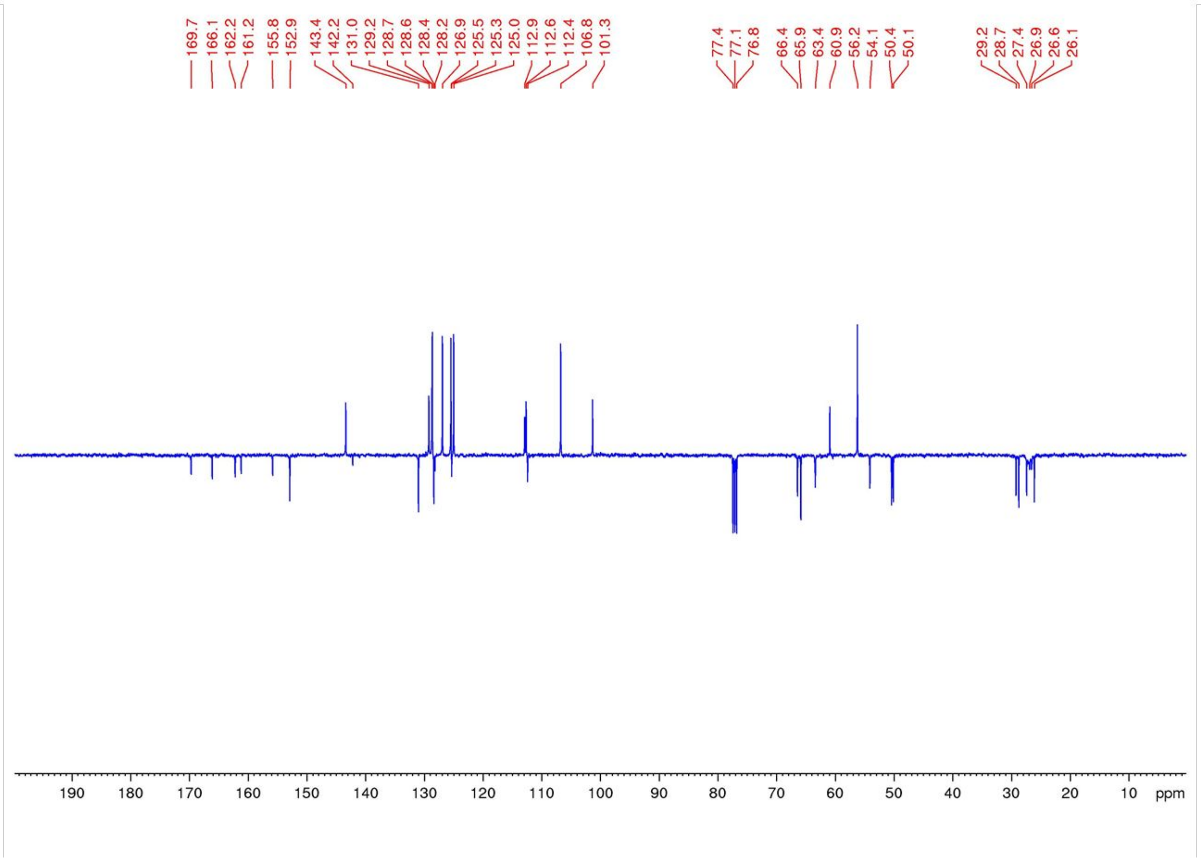

^{1}H-NMR and ^{13}C-APT-NMR spectra of compound **27**

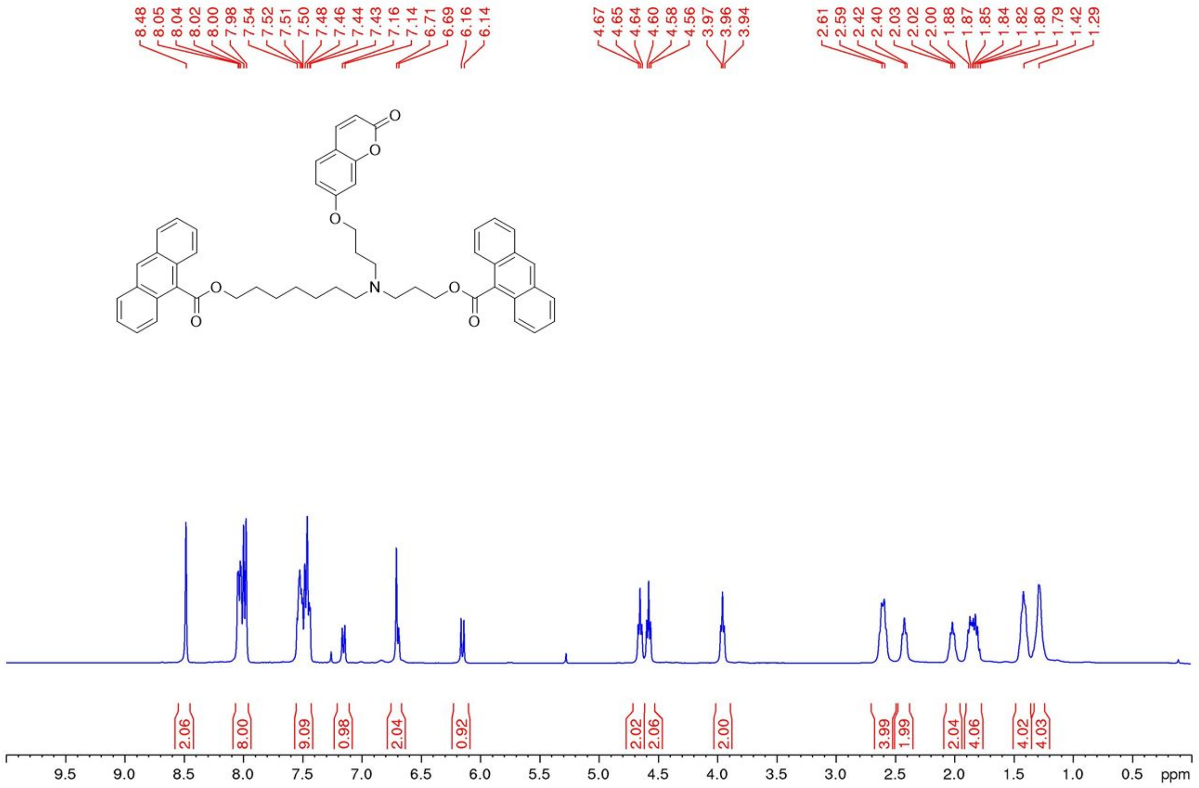

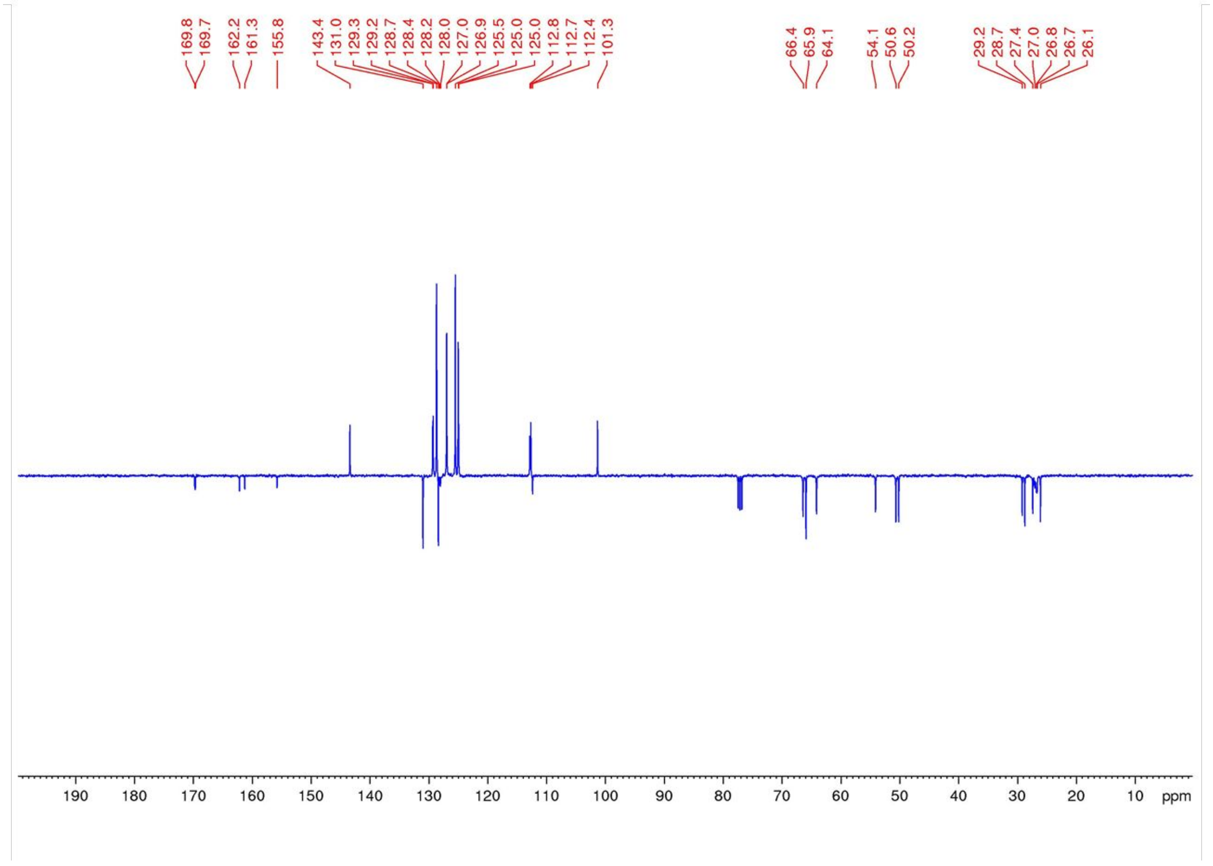

Chemical stability data

Instrumental. The LC-MS/MS analyses were carried out using a Varian 1200L triple quadrupole system (Palo Alto, CA, USA) equipped by two Prostar 210 pumps, a Prostar 410 autosampler and an Elettrospray Source (ESI) operating in positive ions mode. Raw-data were collected and processed by Varian Workstation Vers. 6.8 software. G-Therm 015 thermostatic oven was used to keep the samples at 37 °C during the degradation tests. Eppendorf microcentrifuge 5415D was employed to centrifuge plasma samples.

Standard solutions and calibration curves. Stock solutions of analytes and verapamil hydrochloride (IS) were prepared in acetonitrile at 1.0 mg mL^{-1} and stored at 4 °C. Working solutions of each analyte were freshly prepared by diluting stock solutions up to a concentration of 10 µM and 1 µM (working solution 1 and 2 respectively) in mQ water: acetonitrile 80:20 (v/v) solution. The IS working solution was prepared in acetonitrile at 20 ng mL^{-1} (IS solution).
A seven levels calibration curve was prepared by adding proper volumes of working solution of each analyte to 500 µL of IS solution. The obtained solutions were dried under a gentle nitrogen stream and dissolved in 1.0 mL of 10 mM of formic acid in mQ water: acetonitrile 80:20 (v/v) solution. Final concentrations of calibration levels were: 0, 2.5, 5, 10, 25, 50 and 100 ng mL^{-1} of analyte in the sample. All calibration levels were analysed by the appropriate LC-MS/MS method.

LC-MS/MS method. The chromatographic parameters were reported as follows:
- column, Phenomenex Luna C18 length = 20 mm; internal diameter = 2 mm; particle size = 3 µm, purchased from Merck.
- acidic mobile phase, composed by 5 mM of ammonium formate and 10mM of formic acid in mQ water: acetonitrile 90:10 (v/v) solution (solvent A), 5 mM of ammonium formate and 10mM of formic acid in mQ water: acetonitrile 10:90 (v/v) solution (solvent B).
- flow rate and the injection volume were 0.25 mL min^{-1} and 5 µL, respectively.

The elution gradient is shown in Table S1.

Table S1: Elution gradient of mobile phase used for LC-MS/MS analyses

Time (min)	A (%)	B (%)
0.00	90	10
4.00	10	90
6.00	10	90
6.10	90	10
8.00	90	10

The analyses were acquired in MRM (Multiple Reaction Monitoring), using Argon as collision gas, and parameters are reported in Table S2.

Table S2: MRM parameters

Compounds	Precursor ion (m/z)	Quantitation ion (m/z) [CE (V)]	Qualification ion (m/z) [CE (V)]
**KEE**	283	209 [15]	105 [30]
**1**	804	221 [30]	279 [30]
**2**	778	221 [50]	195 [45]
**3**	788	205 [45]	221 [40]
**4**	778	221 [35]	195 [35]
**5**	752	195 [50]	253 [45]
**6**	762	205 [45]	195 [50]
**7**	788	205 [35]	221 [30]
**8**	762	205 [40]	195 [50]
**9**	772	205 [45]	263 [40]
**10**	818	221 [35]	279 [35]
**11**	792	221 [30]	195 [45]
**12**	802	205 [30]	221 [30]
**13**	792	221 [50]	195 [50]
**14**	766	195 [50]	253 [45]
**15**	776	205 [45]	195 [40]
**16**	802	221 [50]	205 [40]
**17**	776	205 [45]	195 [50]
**18**	786	205 [35]	177 [50]
**19**	832	221 [35]	279 [35]
**20**	806	221 [40]	195 [40]
**21**	816	205 [35]	221 [30]
**22**	806	221 [35]	195 [40]
**23**	780	195 [35]	253 [35]
**24**	790	205 [35]	195 [30]
**25**	816	221 [35]	205 [30]
**26**	790	205 [35]	195 [45]
**27**	800	205 [30]	177 [45]

Linearity and LOD. Calibration curves of analytes were obtained by plotting the peak area ratios (PAR), between quantitation ions of analyte and IS, versus the nominal concentration of the calibration solution. A linear regression analysis was applied to obtain the best fitting function between the calibration points.

The precision was evaluated through the relative standard deviation (RSD%) of the quantitative data of the replicate analysis of highest level of calibration curves.

In order to obtain reliable LOD values, the standard deviation of response and slope approach was employed. The estimated standard deviations of responses were obtained by the standard deviation of y-intercepts (SDY-I) of regression lines. The obtained linear regressions, the linearity coefficients, precision and the estimated LOD values for each analyte are reported in Table S3.

Table S3: Linear regressions data, linearity coefficients, precision and LOD values for each analyte

Compounds	Slope (PAR/μM)	Intercept (PAR)	R^2	Precision (RSD)	LOD (μM)
**1**	2.53	0.04	0.999	5.5%	0.03
**2**	2.41	0.11	0.996	0.7%	0.04
**3**	5.35	0.07	0.996	3.3%	0.05
**4**	2.74	0.03	0.998	1.9%	0.04
**5**	5.86	0.14	0.998	8.1%	0.04
**6**	6.37	0.08	0.997	8.6%	0.06
**7**	4.22	0.08	0.996	2.7%	0.04
**8**	4.27	0.06	0.998	3.1%	0.06
**9**	3.29	0.05	0.998	6.7%	0.04
**10**	3.68	0.10	0.998	2.5%	0.04
**11**	3.72	0.08	0.999	6.7%	0.04
**12**	3.49	0.10	0.998	7.1%	0.04
**13**	3.92	0.13	0.999	4.7%	0.05
**14**	1.68	0.14	0.997	3.4%	0.04
**15**	6.83	0.15	0.998	4.7%	0.06
**16**	1.41	0.17	0.999	0.7%	0.05
**17**	1.43	0.11	0.997	3.3%	0.06
**18**	2.97	0.09	0.994	1.0%	0.07
**19**	3.97	0.12	0.991	0.8%	0.07
**20**	3.43	0.07	0.999	1.1%	0.03
**21**	2.93	0.14	0.996	5.6%	0.06
**22**	4.98	0.11	0.998	5.0%	0.04
**23**	5.8	0.12	0.998	1.4%	0.04
**24**	6.0	0.3	0.996	5.3%	0.06
**25**	3.6	0.29	0.991	4.9%	0.09
**26**	4.4	0.3	0.993	5.9%	0.08
**27**	5.7	0.3	0.991	2.1%	0.09

Solution stability profiles. The solution stability profiles in PBS and human plasma were obtained by monitoring the variation of analyte concentration at different incubation times. They are reported in Figures S1-S27.

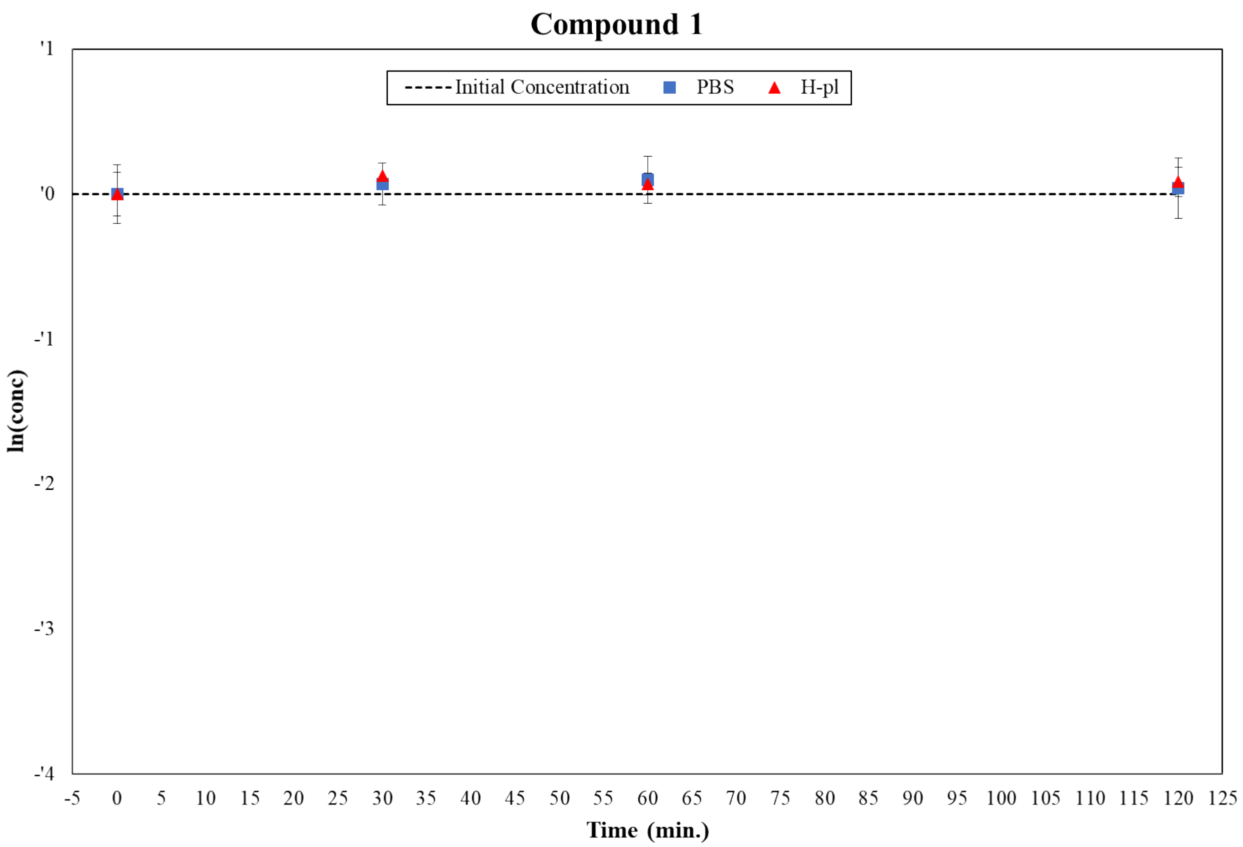

Figure S1: Degradation plots of **1** in PBS (blue square) and human plasma (red triangle), with the proper error bars.

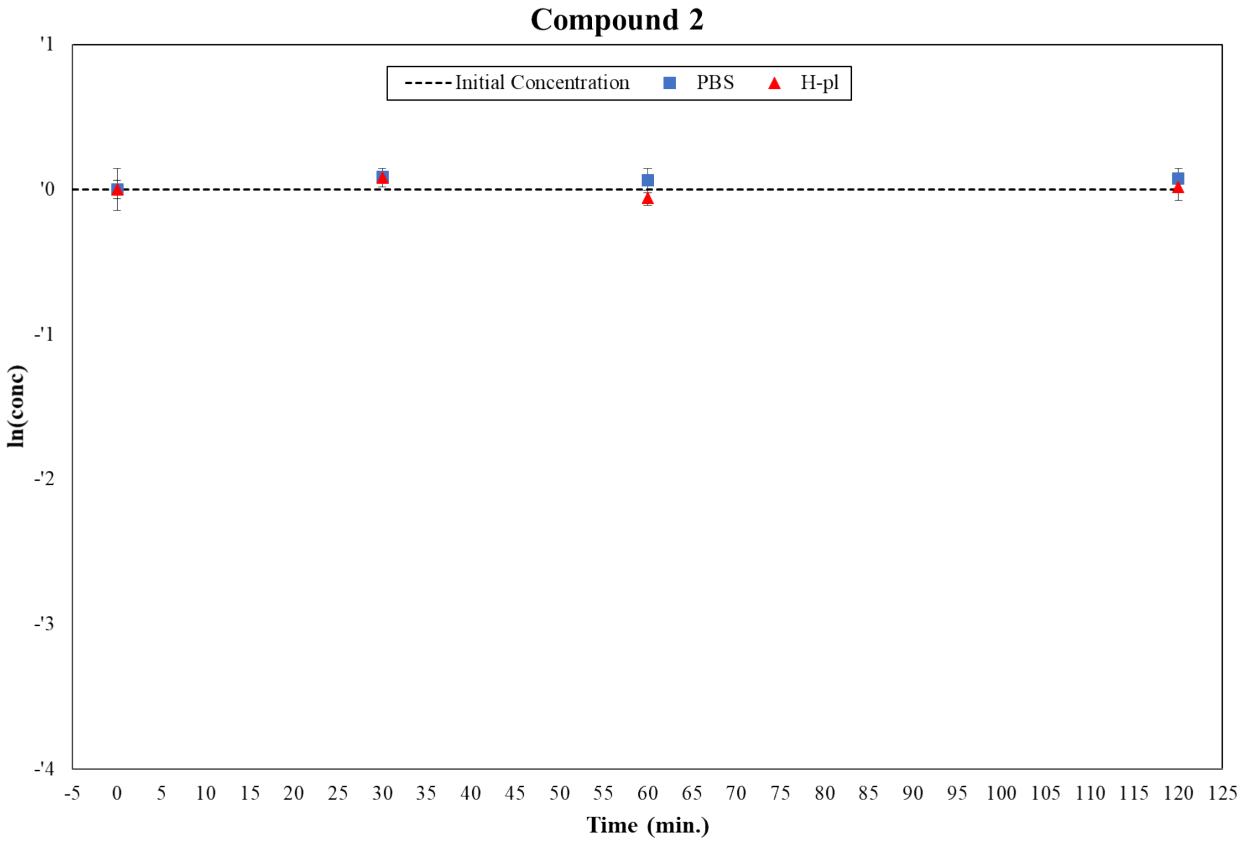

Figure S2: Degradation plots of **2** in PBS (blue square) and human plasma (red triangle), with the proper error bars.

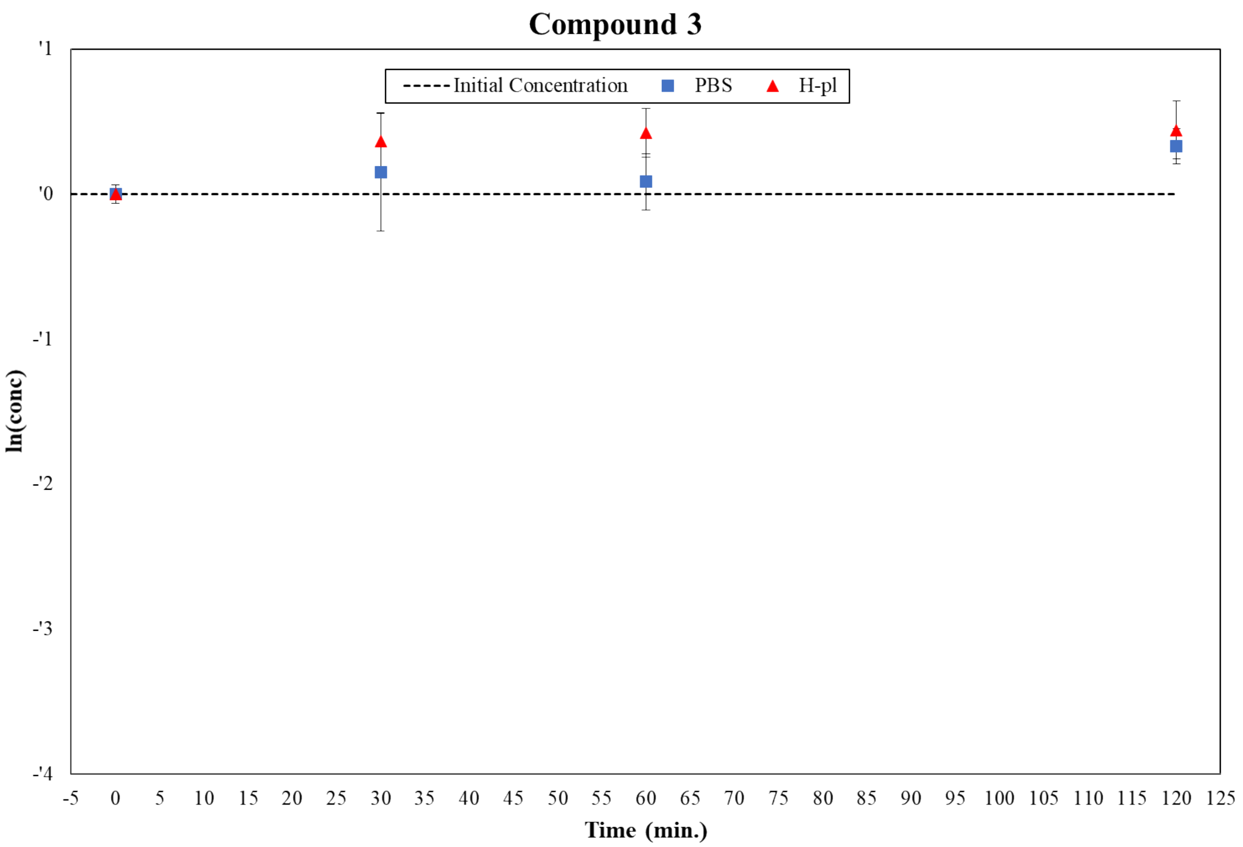

Figure S3: Degradation plots of **3** in PBS (blue square) and human plasma (red triangle), with the proper error bars.

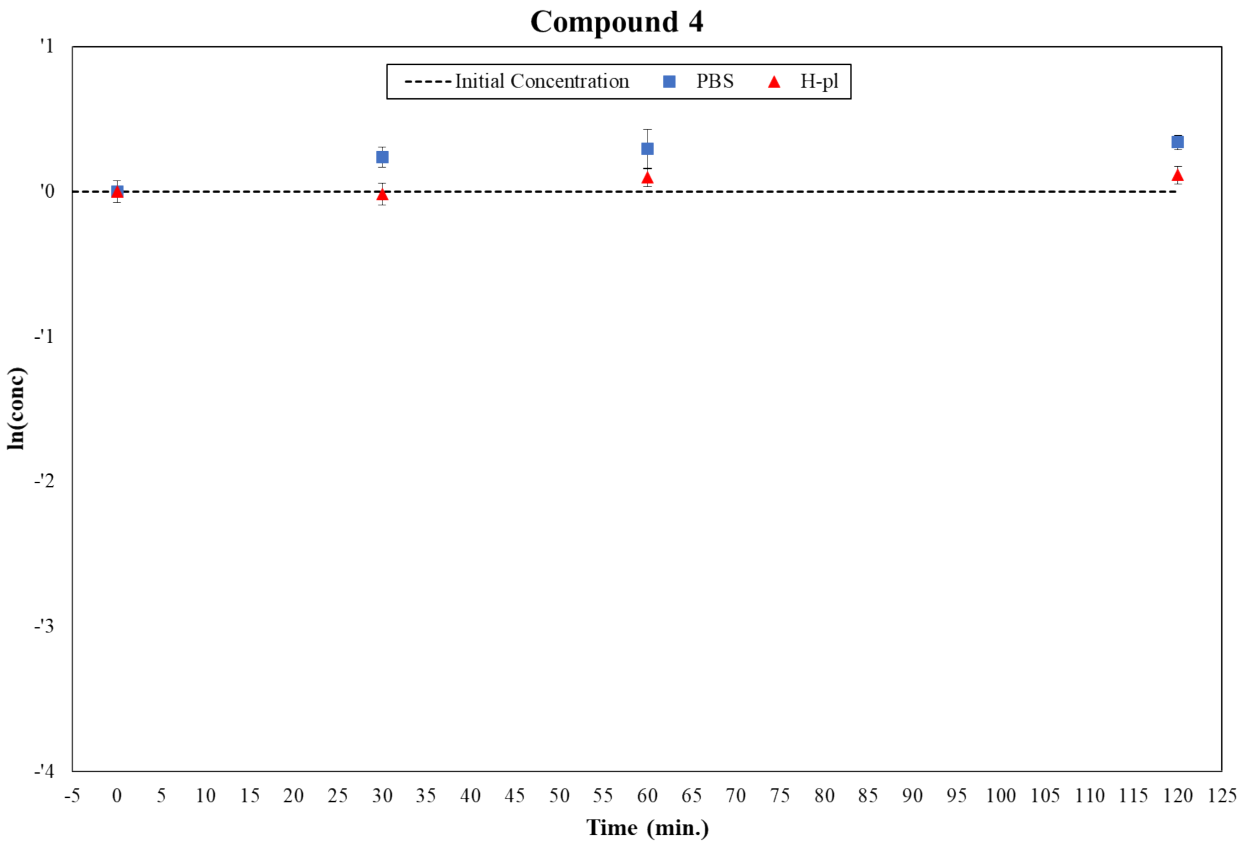

Figure S4: Degradation plots of **4** in PBS (blue square) and human plasma (red triangle), with the proper error bars.

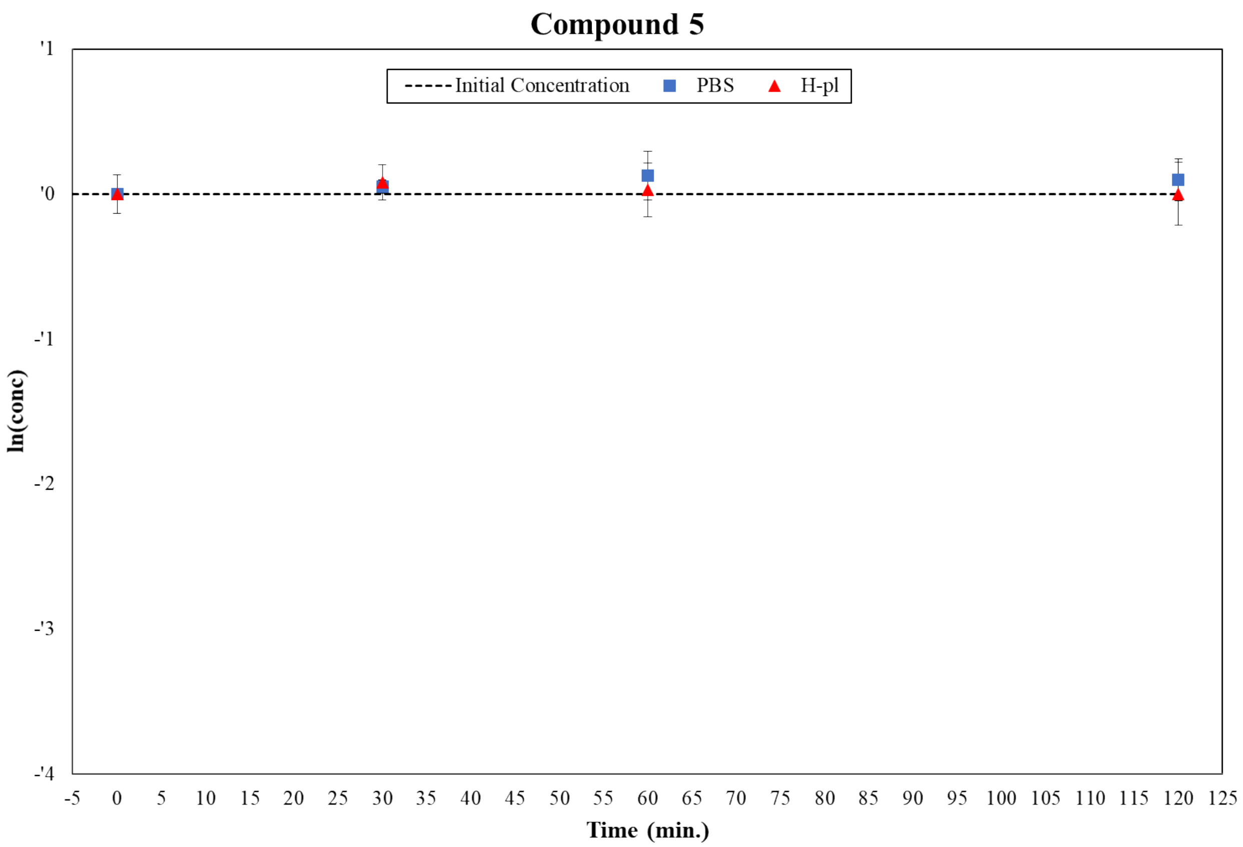

Figure S5: Degradation plots of **5** in PBS (blue square) and human plasma (red triangle), with the proper error bars.

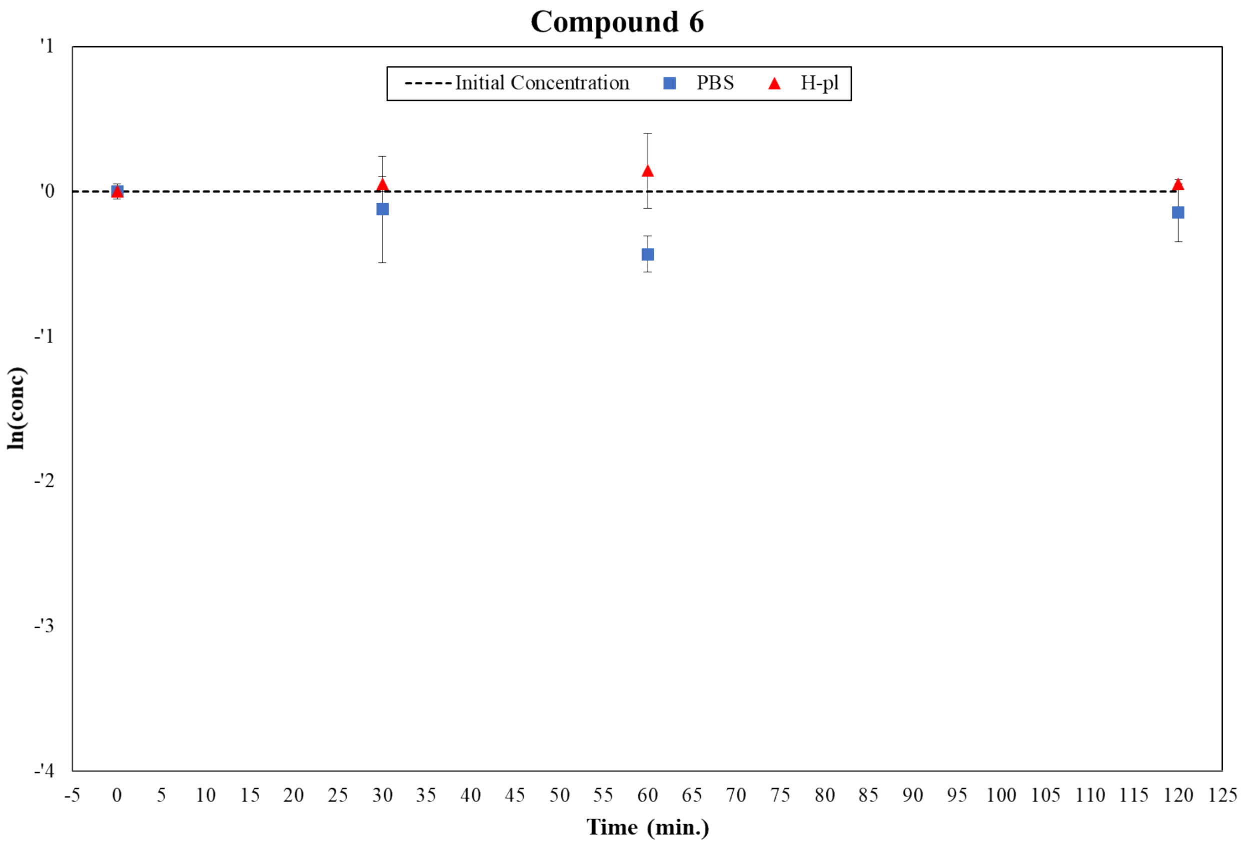

Figure S6: Degradation plots of **6** in PBS (blue square) and human plasma (red triangle), with the proper error bars.

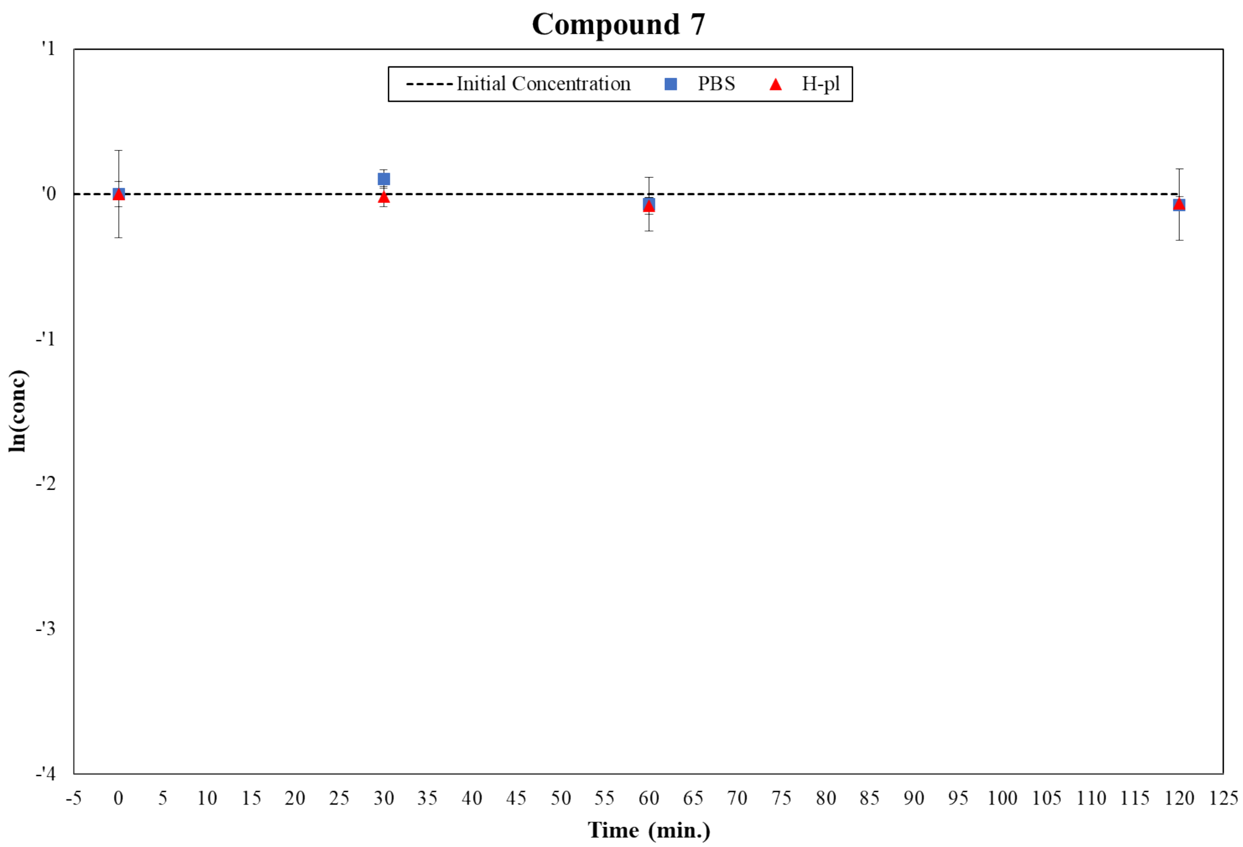

Figure S7: Degradation plots of **7** in PBS (blue square) and human plasma (red triangle), with the proper error bars.

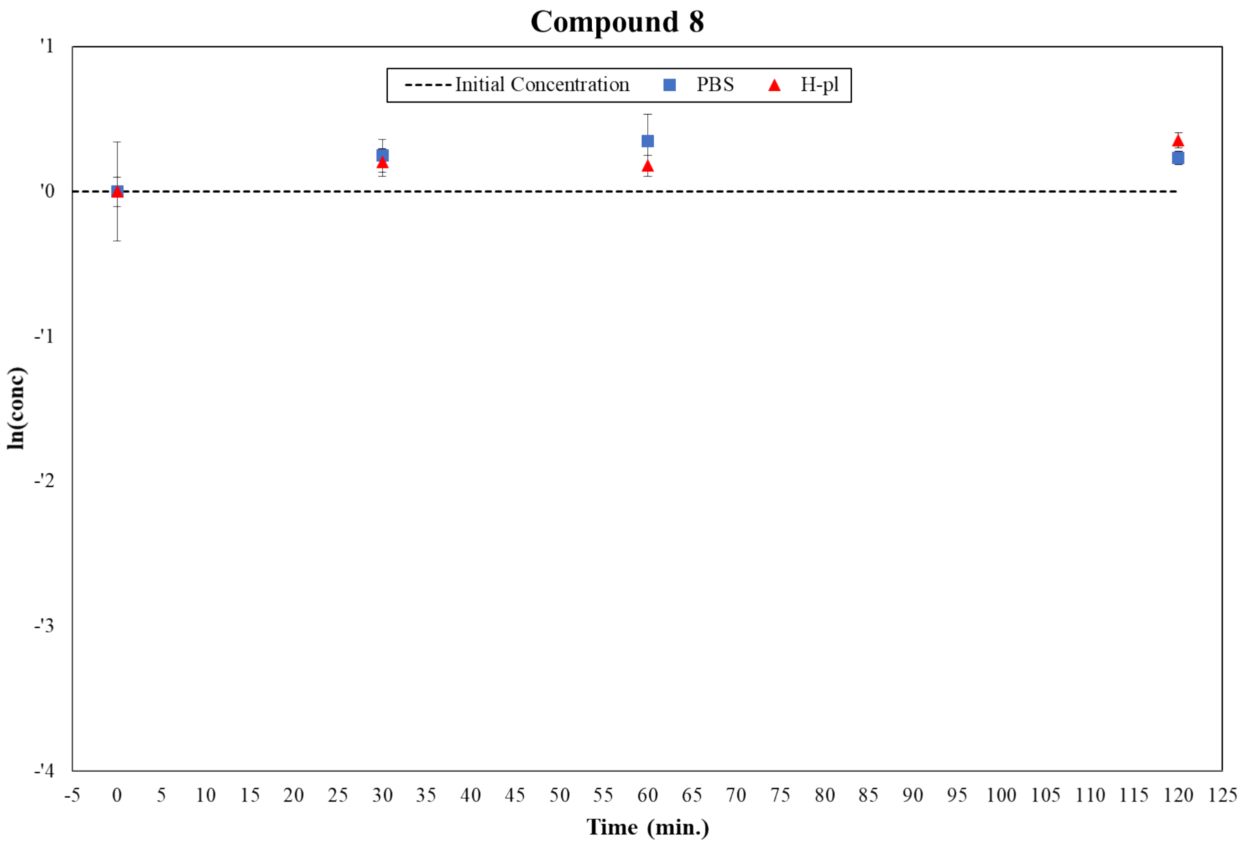

Figure S8: Degradation plots of **8** in PBS (blue square) and human plasma (red triangle), with the proper error bars.

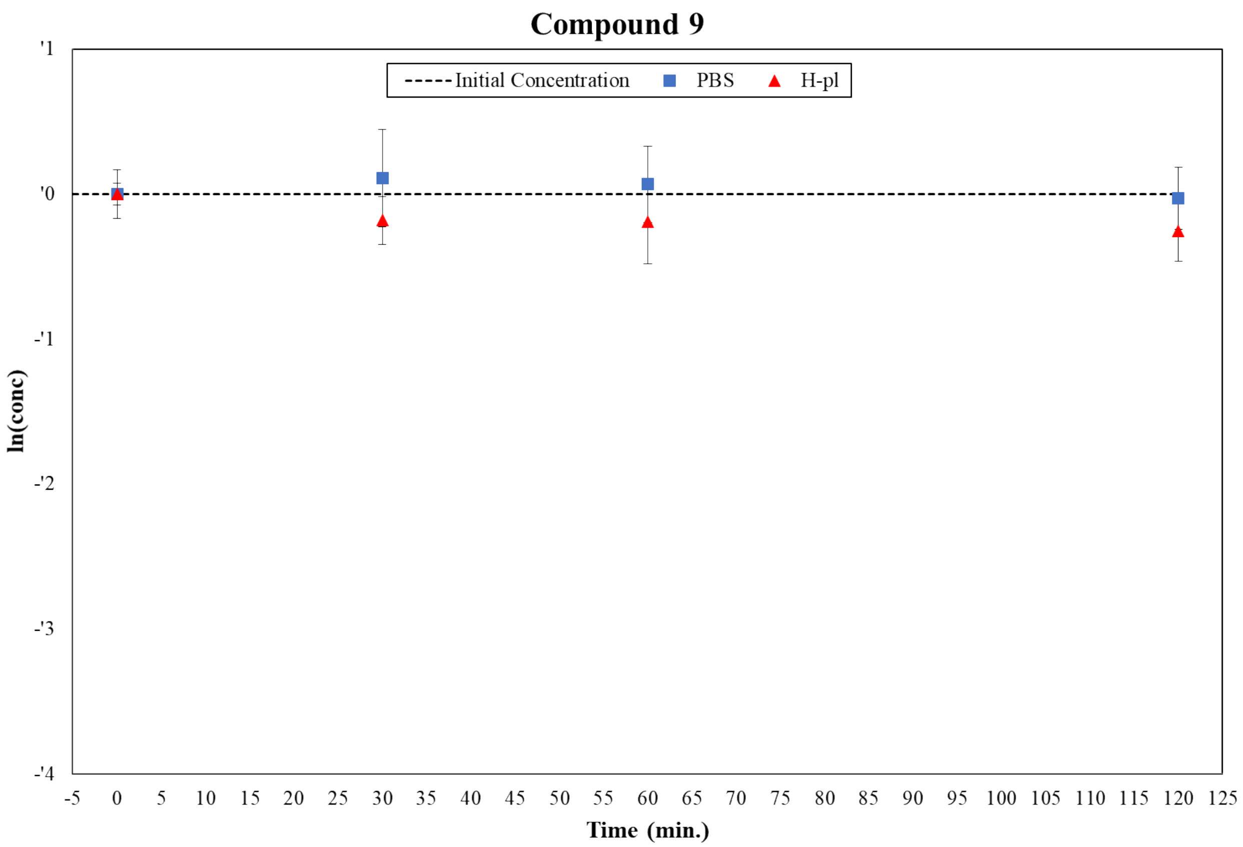

Figure S9: Degradation plots of **9** in PBS (blue square) and human plasma (red triangle), with the proper error bars.

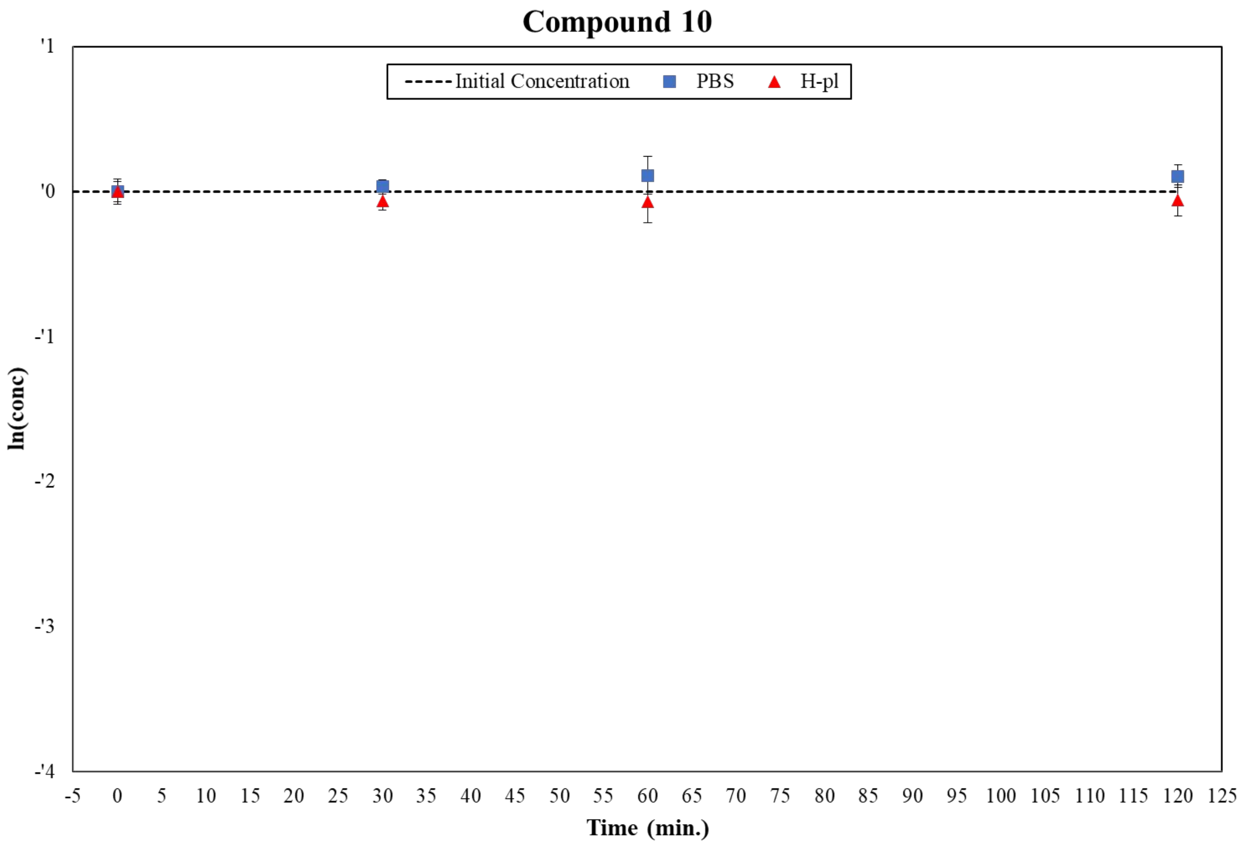

Figure S10: Degradation plots of **10** in PBS (blue square) and human plasma (red triangle), with the proper error bars.

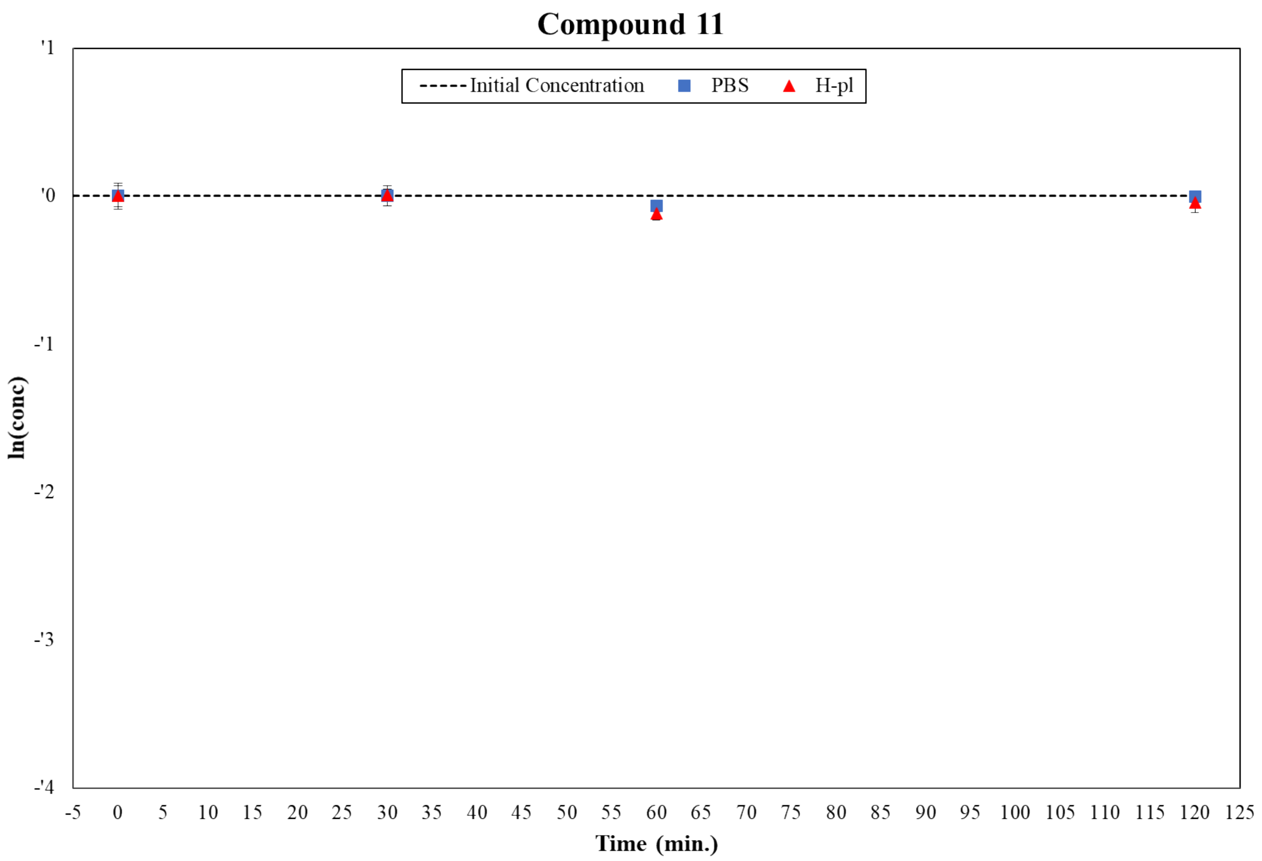

Figure S11: Degradation plots of **11** in PBS (blue square) and human plasma (red triangle), with the proper error bars.

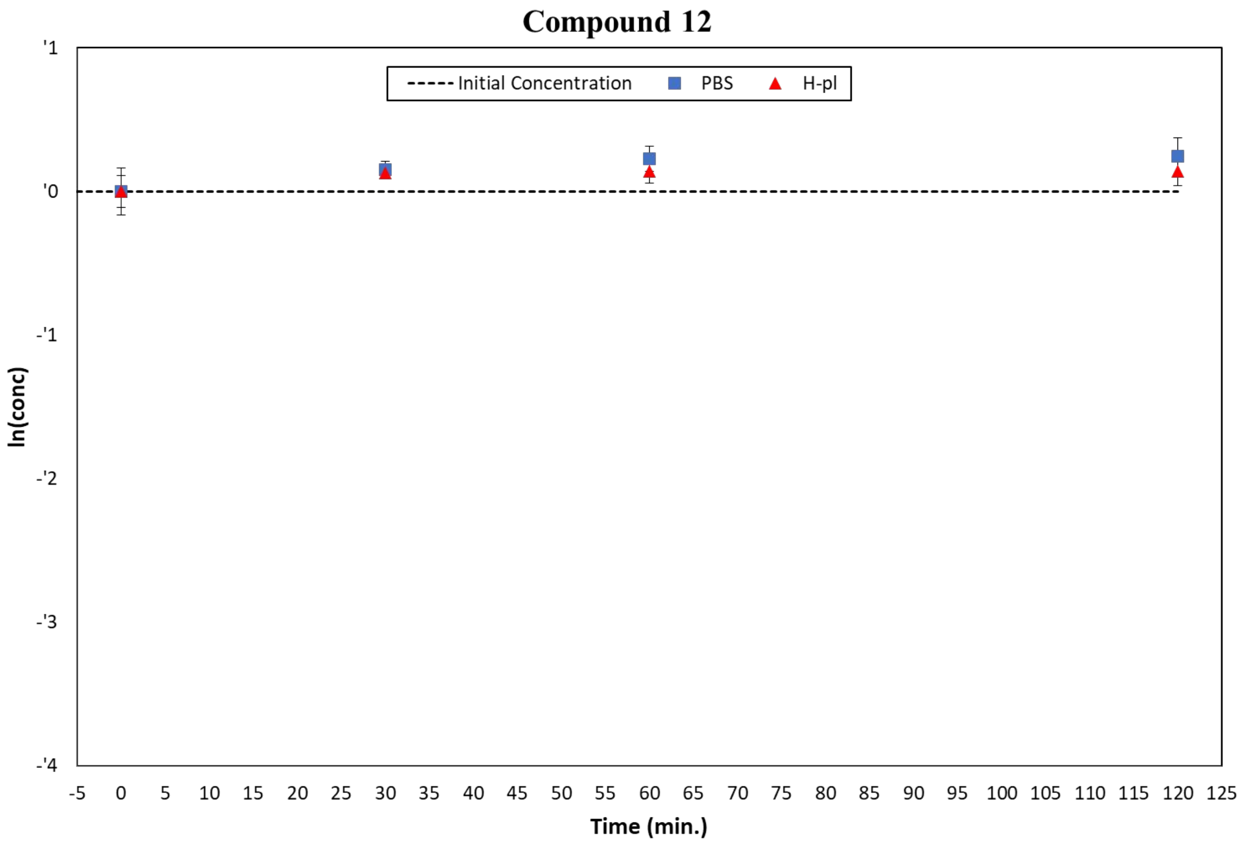

Figure S12: Degradation plots of **12** in PBS (blue square) and human plasma (red triangle), with the proper error bars.

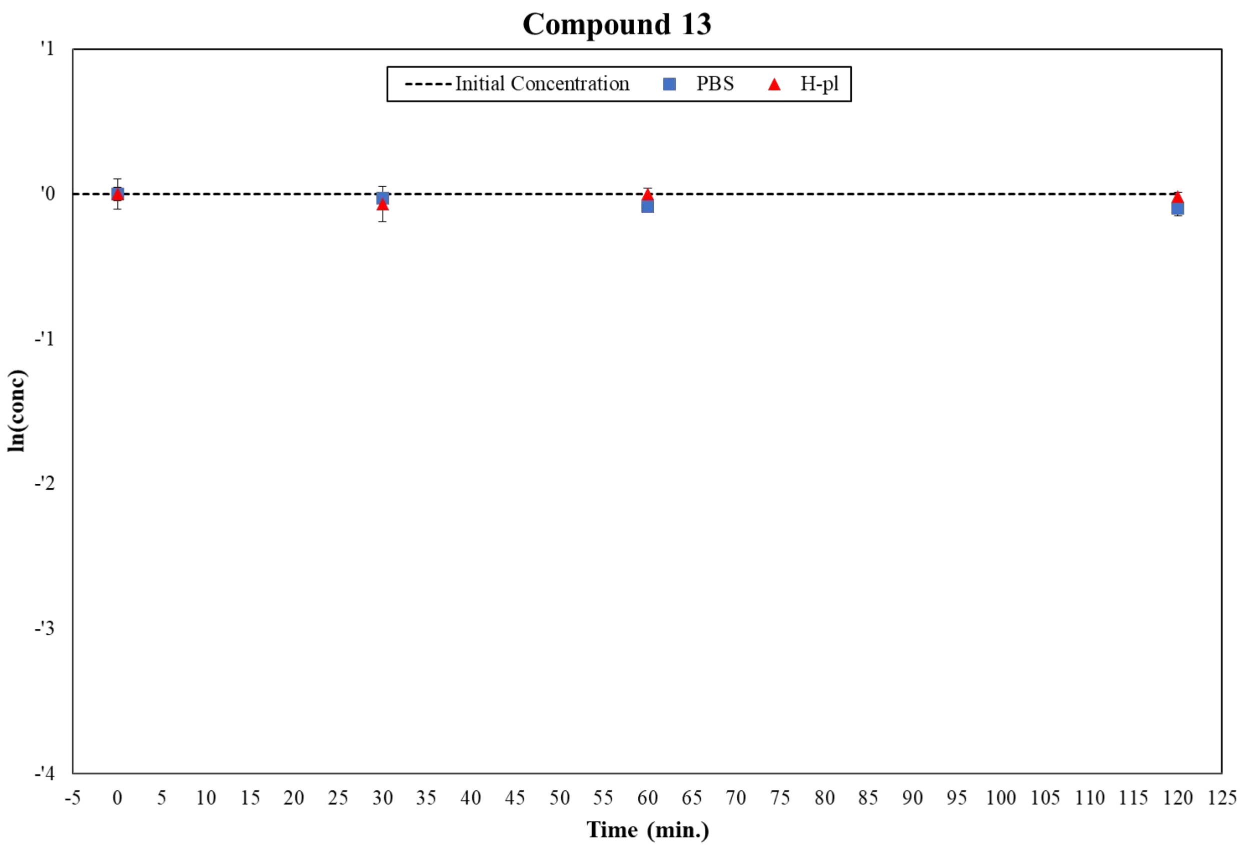

Figure S13: Degradation plots of **13** in PBS (blue square) and human plasma (red triangle), with the proper error bars.

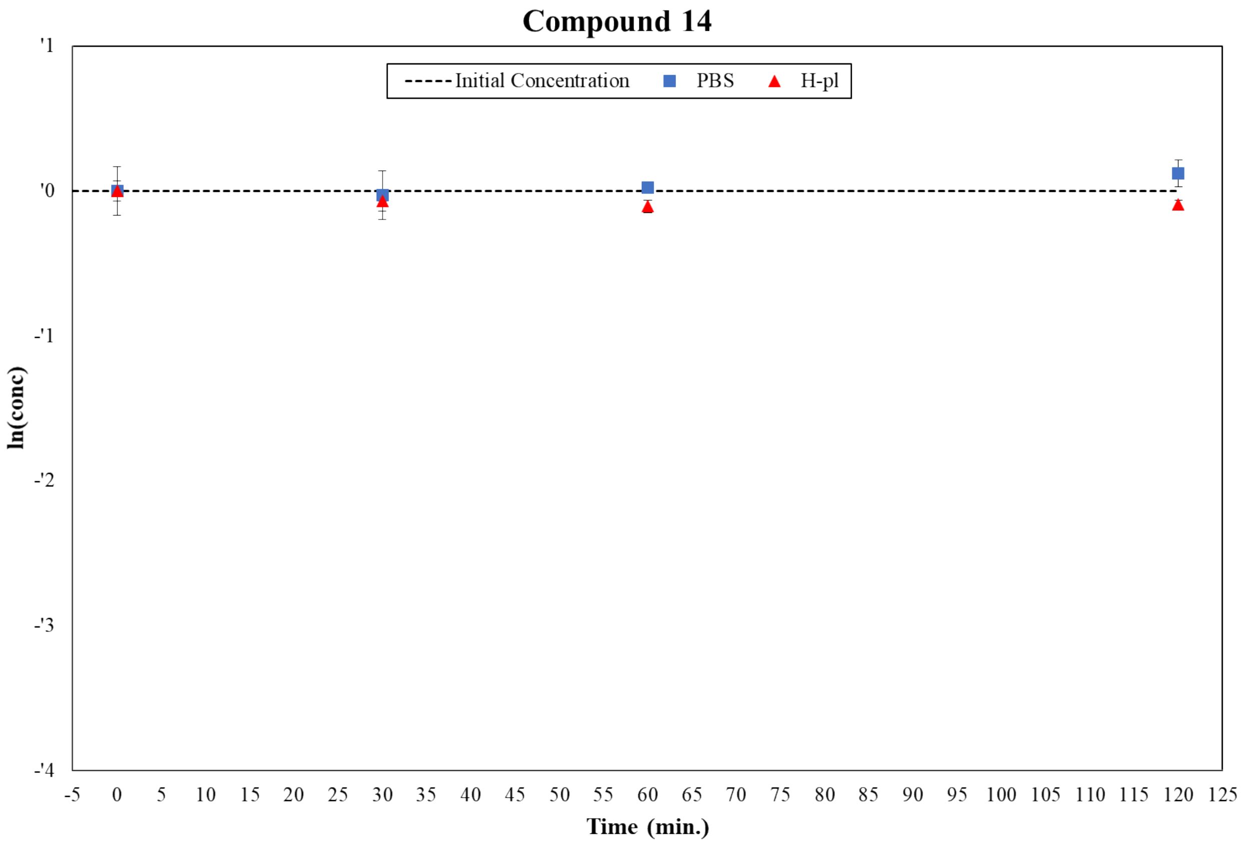

Figure S14: Degradation plots of **14** in PBS (blue square) and human plasma (red triangle), with the proper error bars.

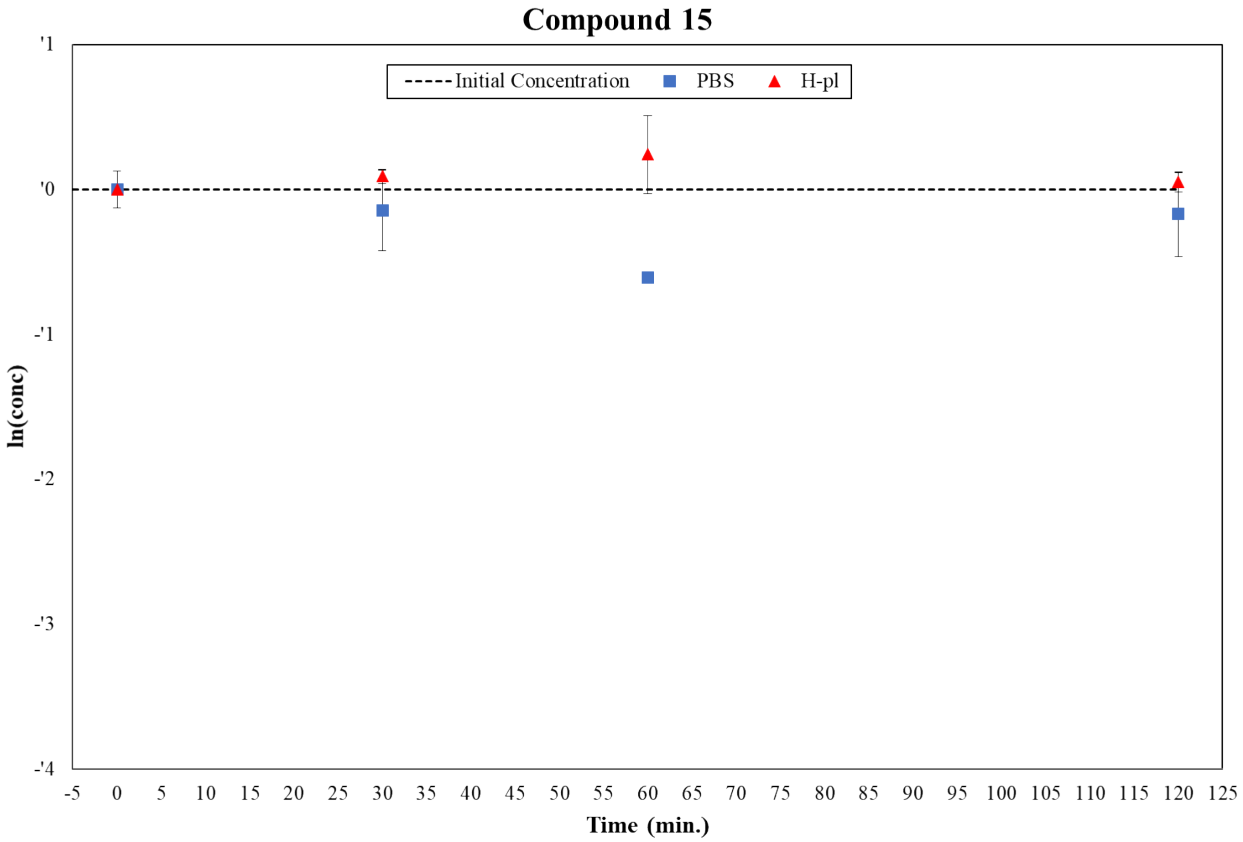

Figure S15: Degradation plots of **15** in PBS (blue square) and human plasma (red triangle), with the proper error bars.

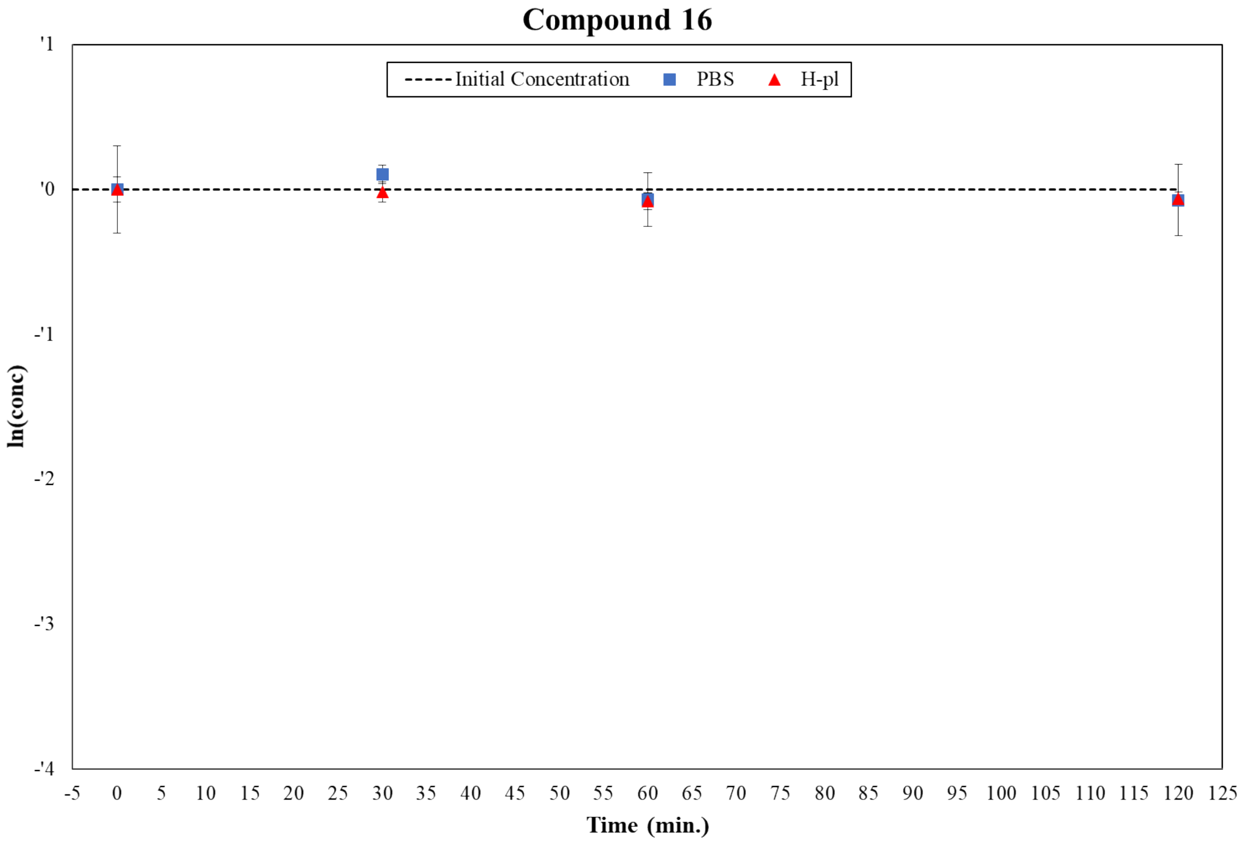

Figure S16: Degradation plots of **16** in PBS (blue square) and human plasma (red triangle), with the proper error bars.

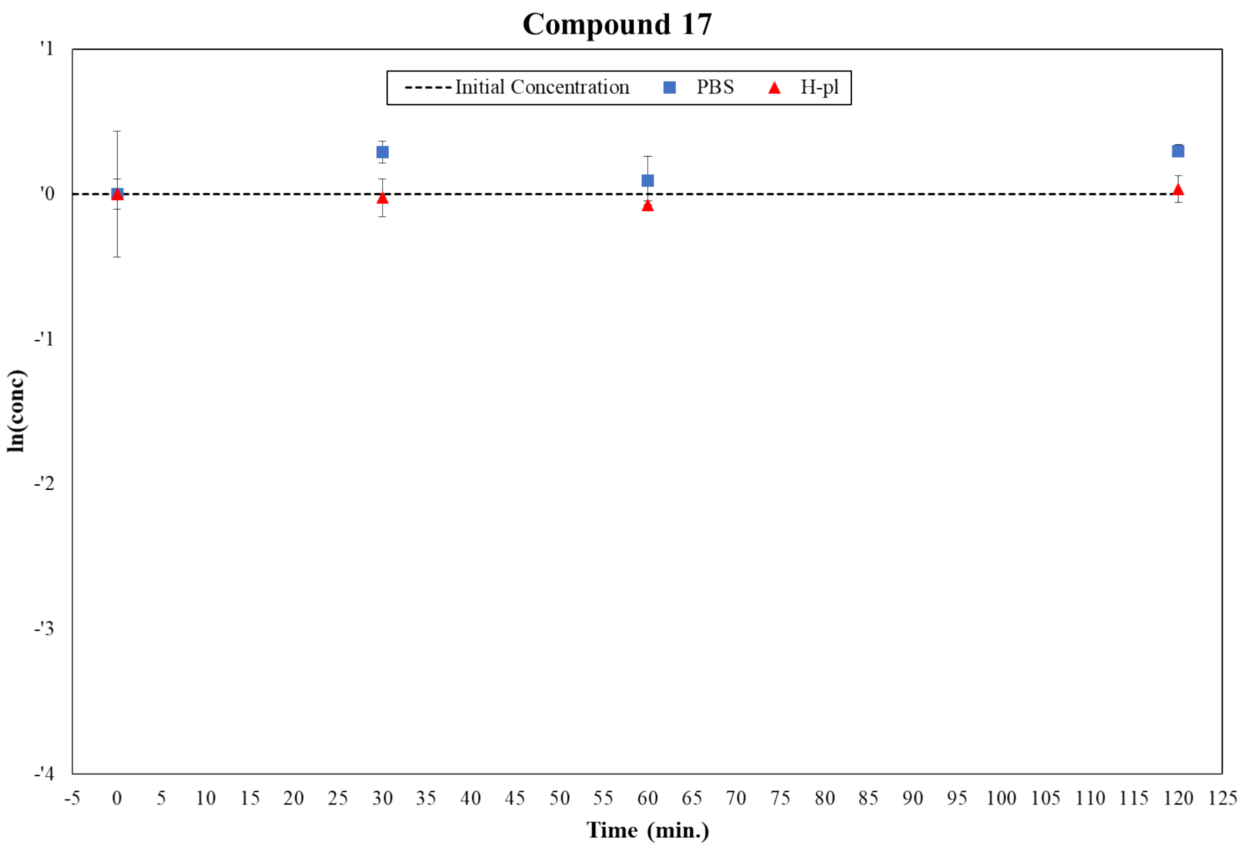

Figure S17: Degradation plots of **17** in PBS (blue square) and human plasma (red triangle), with the proper error bars.

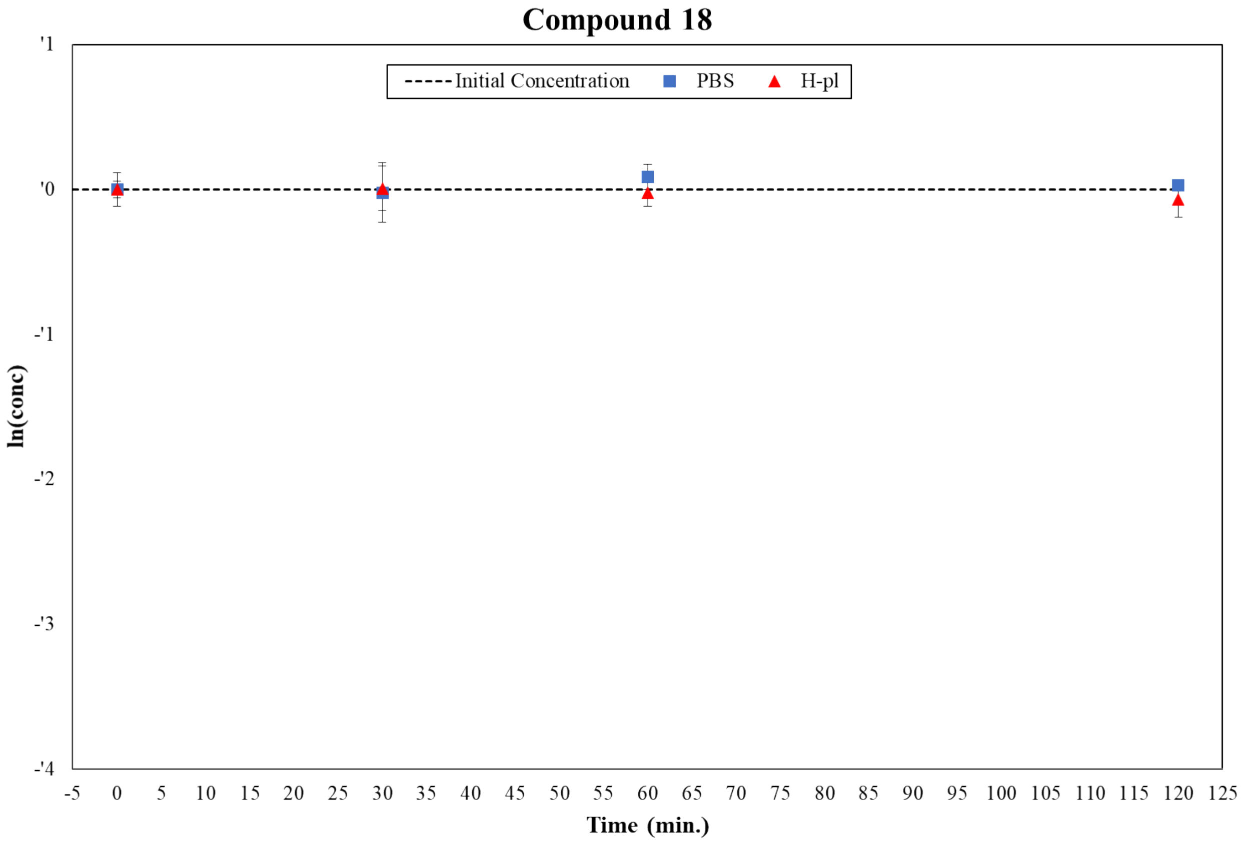

Figure S18: Degradation plots of **18** in PBS (blue square) and human plasma (red triangle), with the proper error bars.

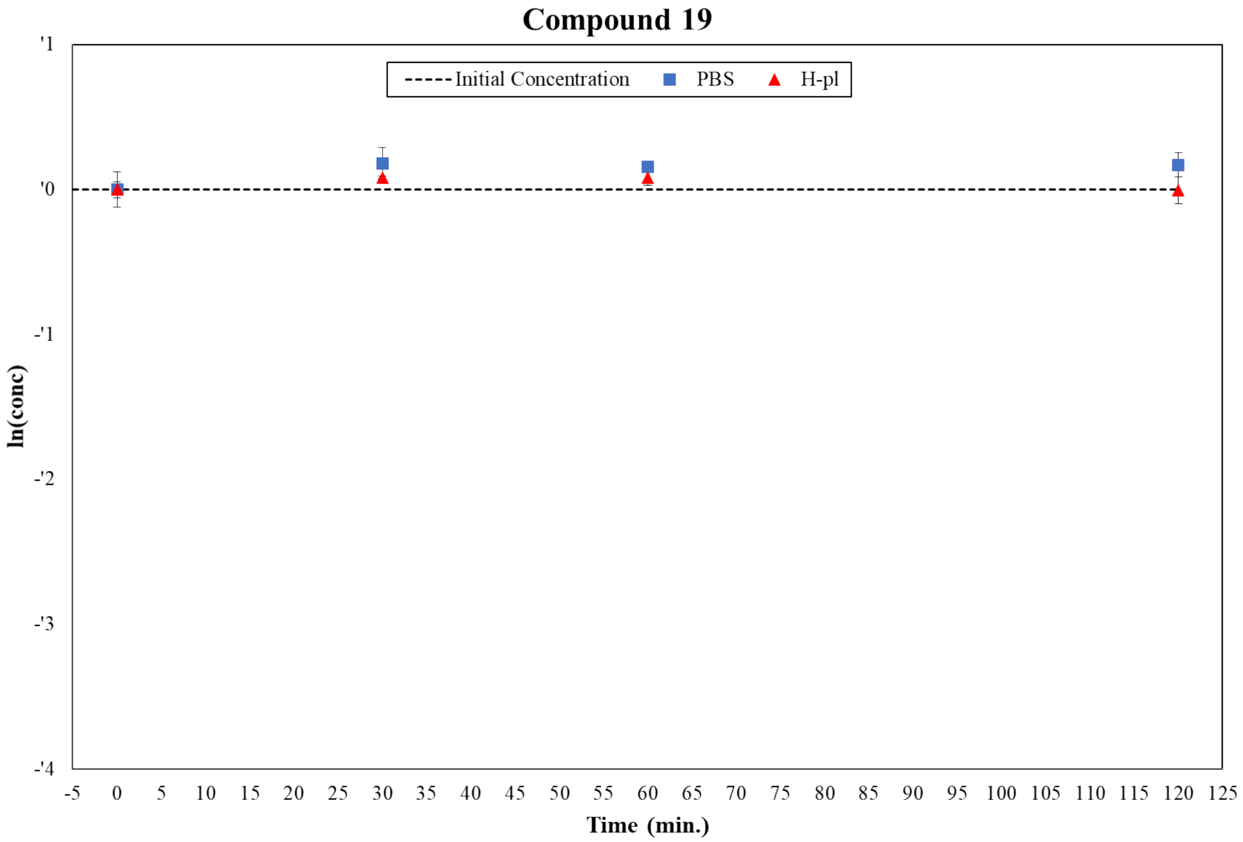

Figure S19: Degradation plots of **19** in PBS (blue square) and human plasma (red triangle), with the proper error bars.

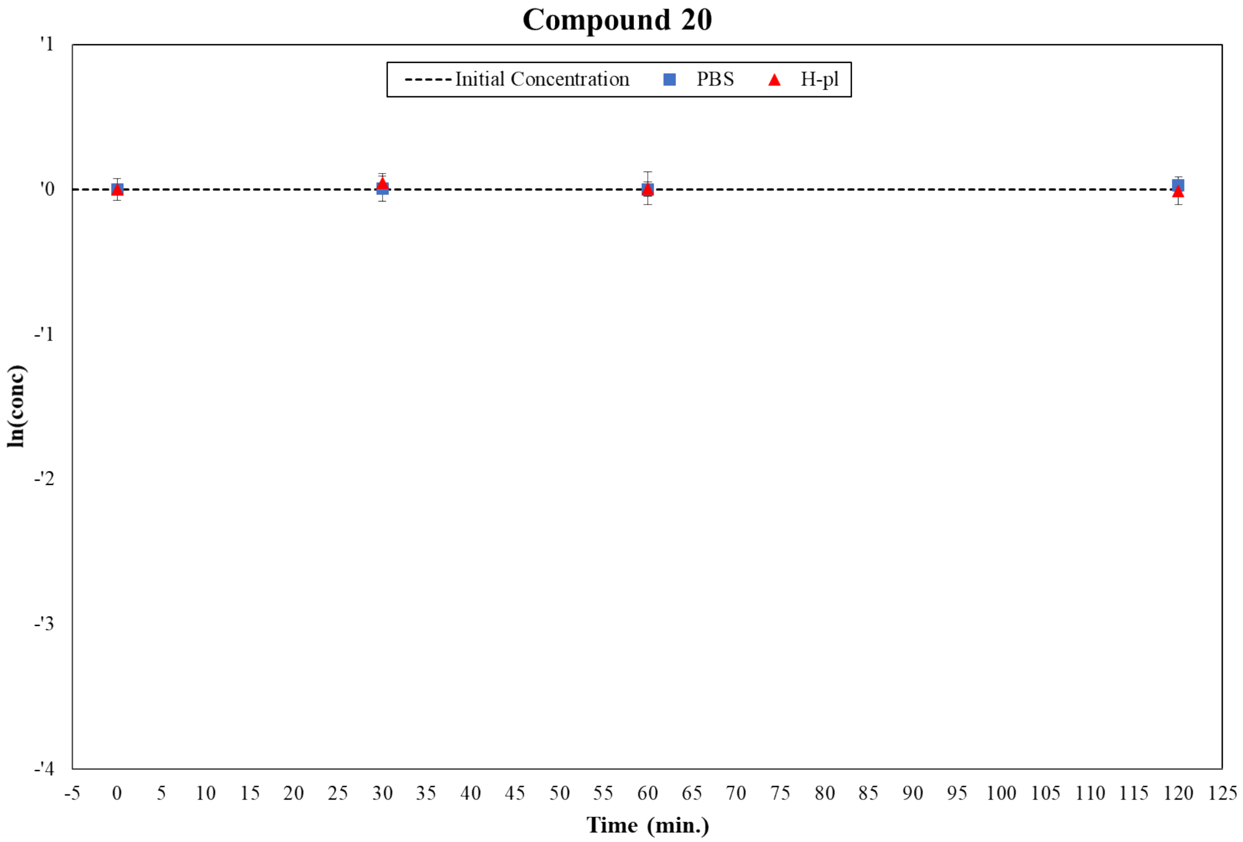

Figure S20: Degradation plots of **20** in PBS (blue square) and human plasma (red triangle), with the proper error bars.

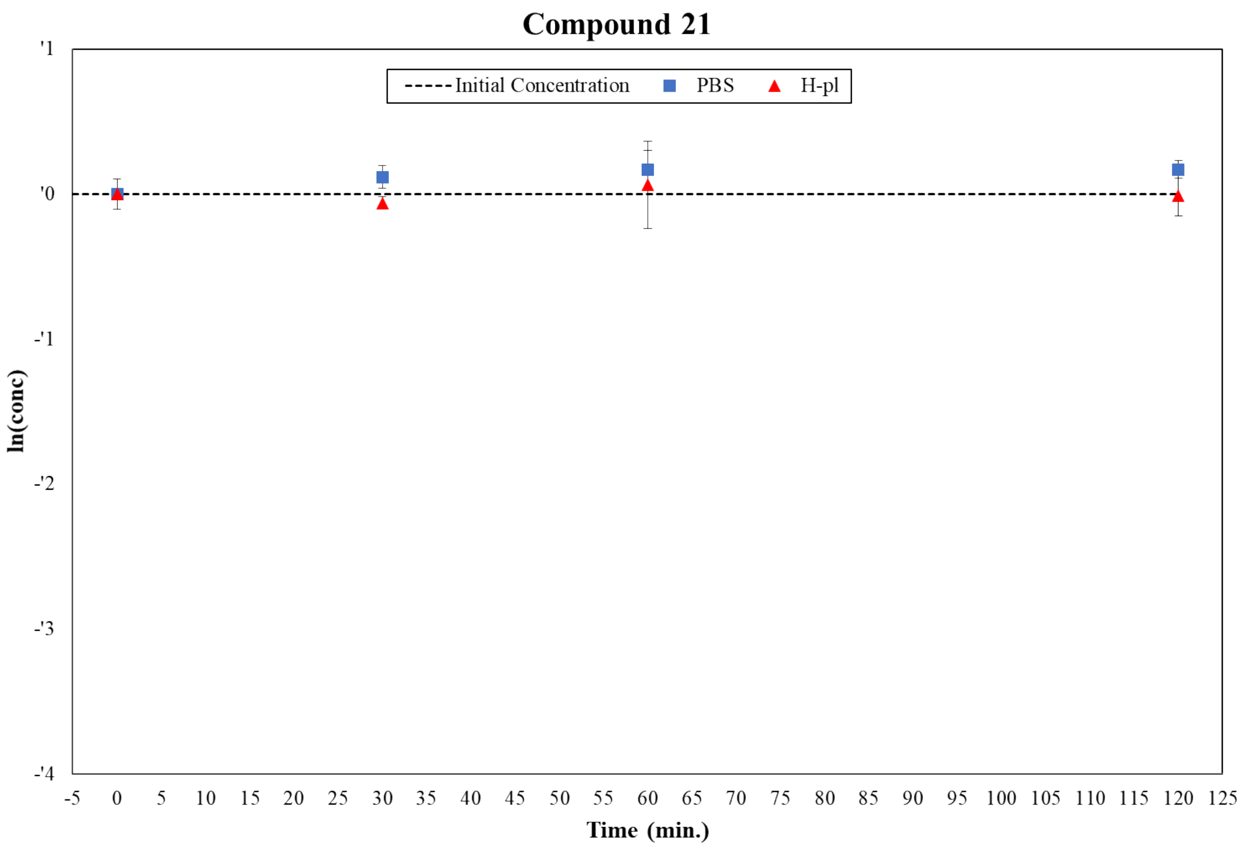

Figure S21: Degradation plots of **21** in PBS (blue square) and human plasma (red triangle), with the proper error bars.

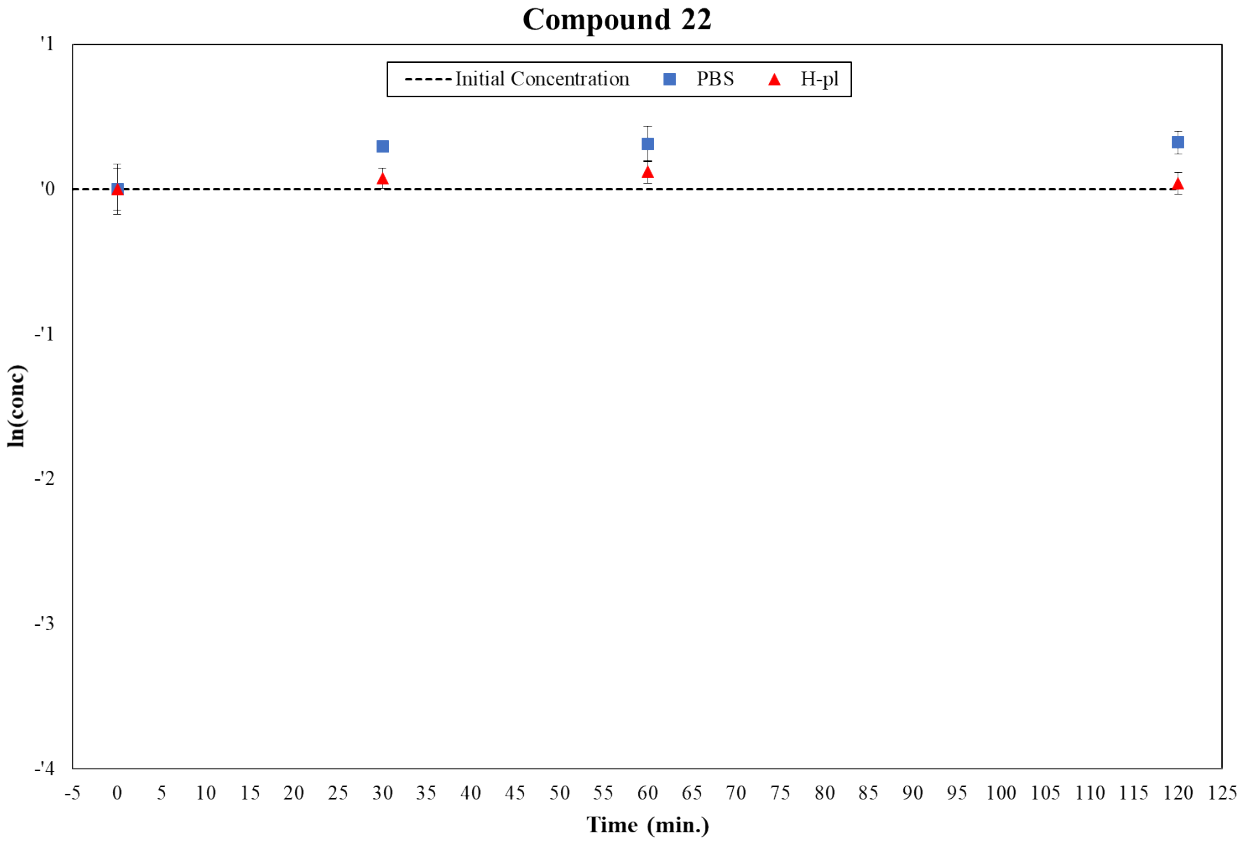

Figure S22: Degradation plots of **22** in PBS (blue square) and human plasma (red triangle), with the proper error bars.

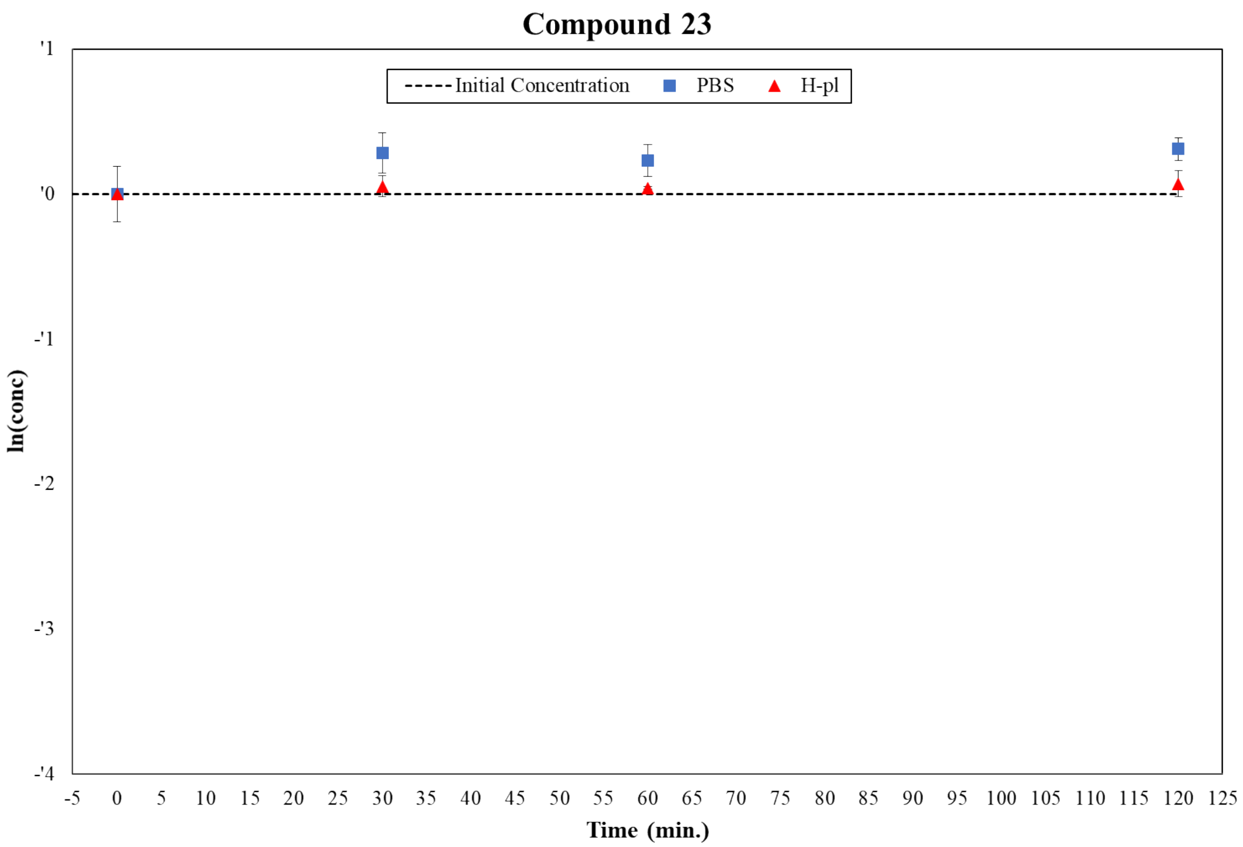

Figure S23: Degradation plots of **23** in PBS (blue square) and human plasma (red triangle), with the proper error bars.

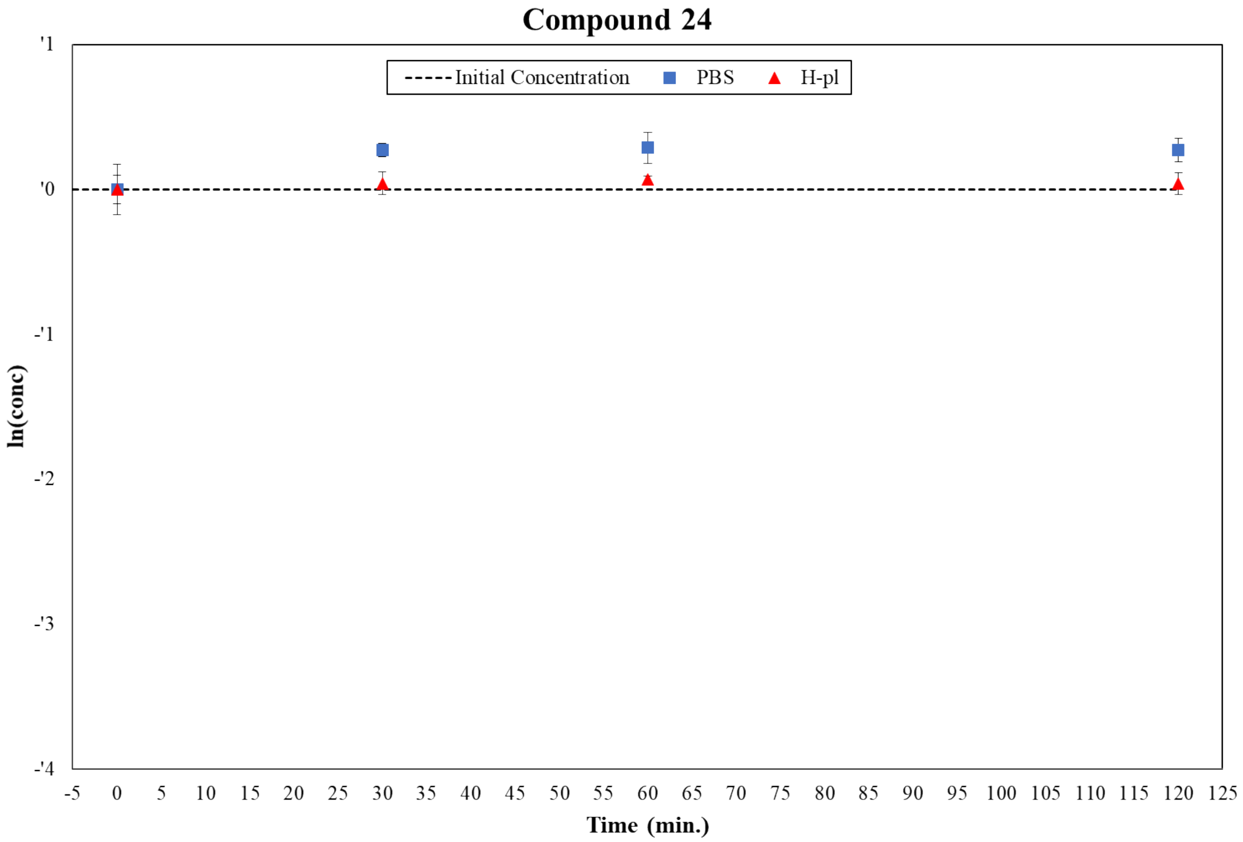

Figure S24: Degradation plots of **24** in PBS (blue square) and human plasma (red triangle), with the proper error bars.

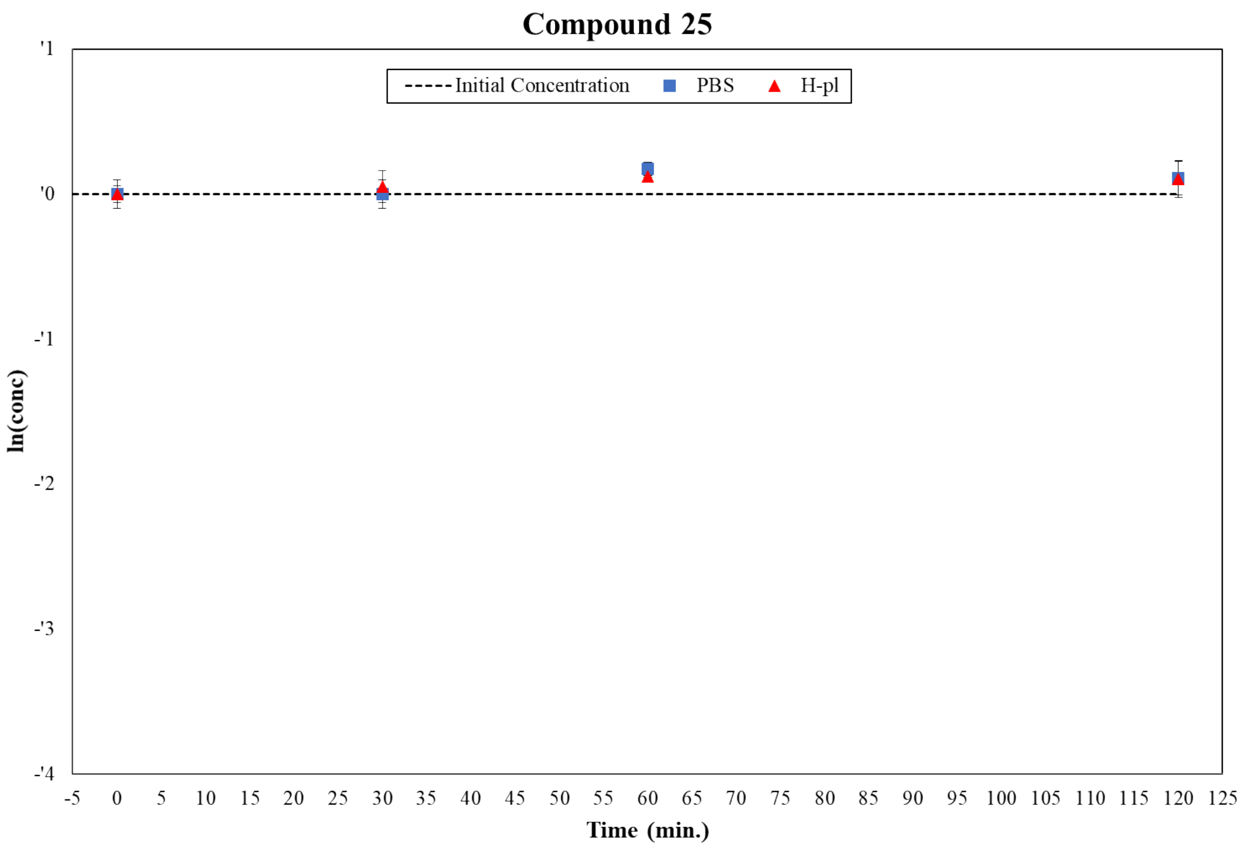

Figure S25: Degradation plots of **25** in PBS (blue square) and human plasma (red triangle), with the proper error bars.

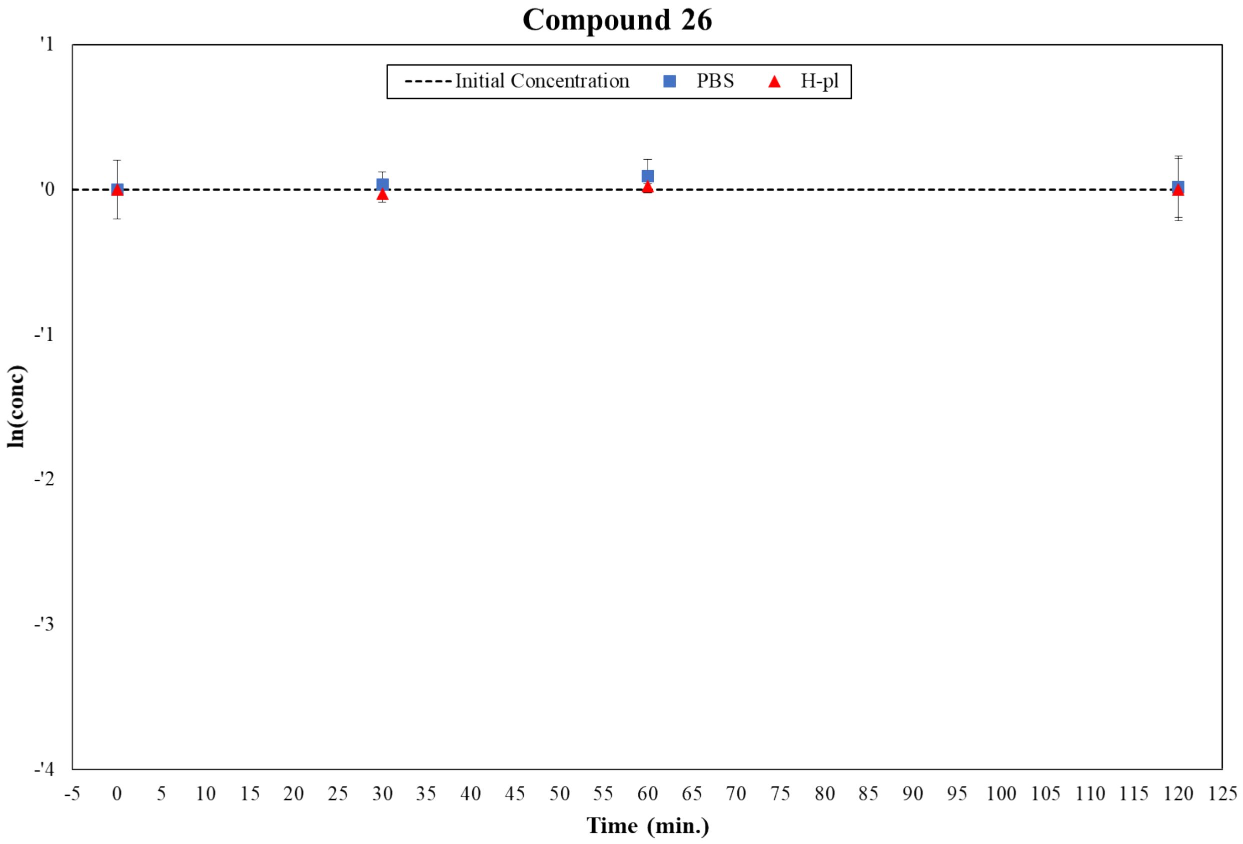

Figure S26: Degradation plots of **26** in PBS (blue square) and human plasma (red triangle), with the proper error bars.

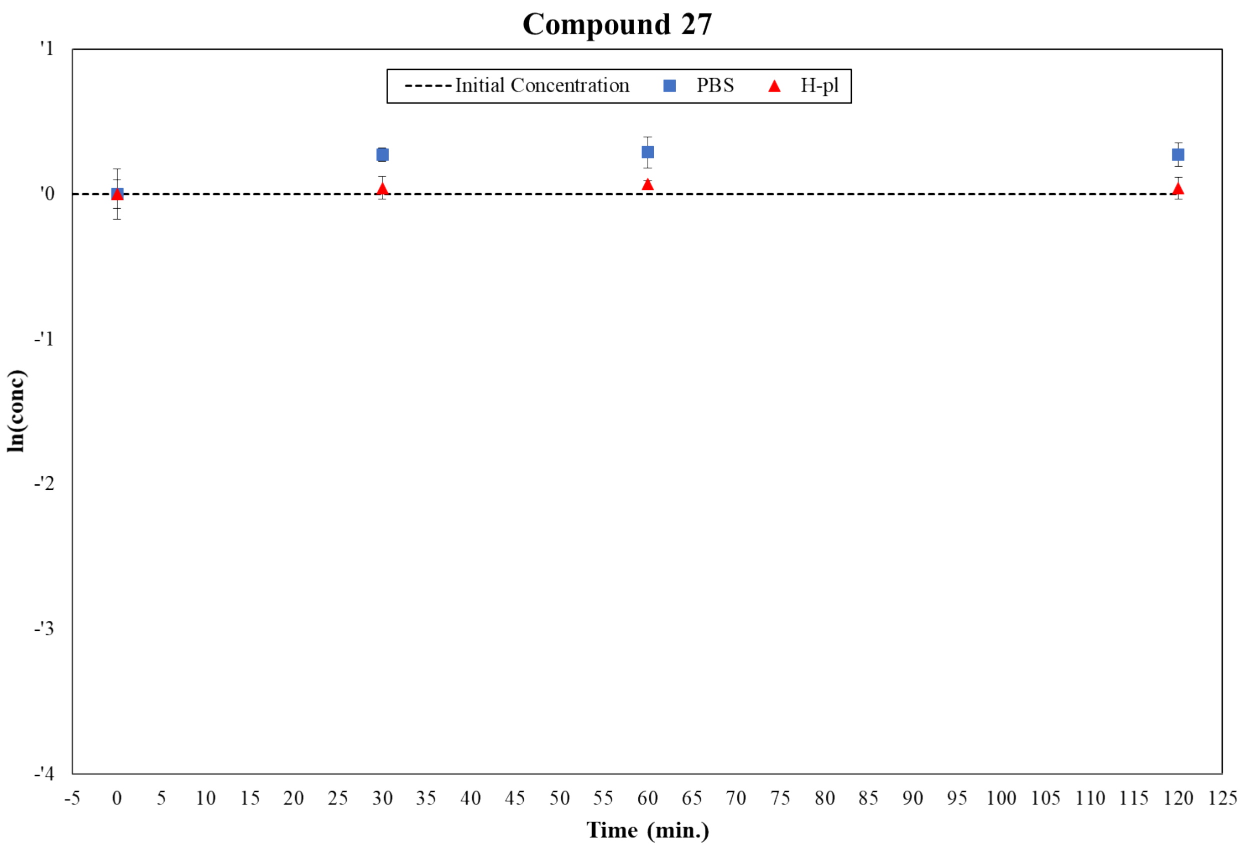

Figure S27: Degradation plots of **27** in PBS (blue square) and human plasma (red triangle), with the proper error bars.

HPLC-DAD method for purity analysis

The employed chromatographic parameters to check the purity of representative compounds were reported as follows:

- column, Pursuit C18 length = 50 mm, internal diameter= 2mm; particle size = 5 µm purchased from Agilent Technologies (Palo Alto, CA, USA)
- acidic mobile phase, composed by 5 mM of ammonium formate and 10mM of formic acid in mQ water: acetonitrile 90:10 (v/v) solution (solvent A), 10 mM of ammonium formate and 5 mM of formic acid in mQ water: acetonitrile 10:90 (v/v) solution (solvent B).
- flow rate and the injection volume were 0.25 mL min^{-1} and 5 µL respectively.
- DAD detection set up was in the UV range between 210 to 400 nm. The chromatographic profile of each analyte was monitored at the λ of its maximum absorbance in acquired range.

The elution gradient is shown in Table S4.

Table S4: Elution gradient of mobile phase used for HPLC-DAD analysis

Time (min)	A (%)
0.00	90
8.00	10
13.00	10
13.01	90
18.00	90

The sample solution of each analyte/compound was prepared at 100 μg mL^{-1} in mQ water: acetonitrile 50:50 and analyzed by the HPLC-DAD method described above.

Chromatographic profiles of HPLC-DAD analysis of representative compounds (**2**, **3**, **5**, **6**, **8**, **9**, **13-17**) were reported in Figures S28-S38.

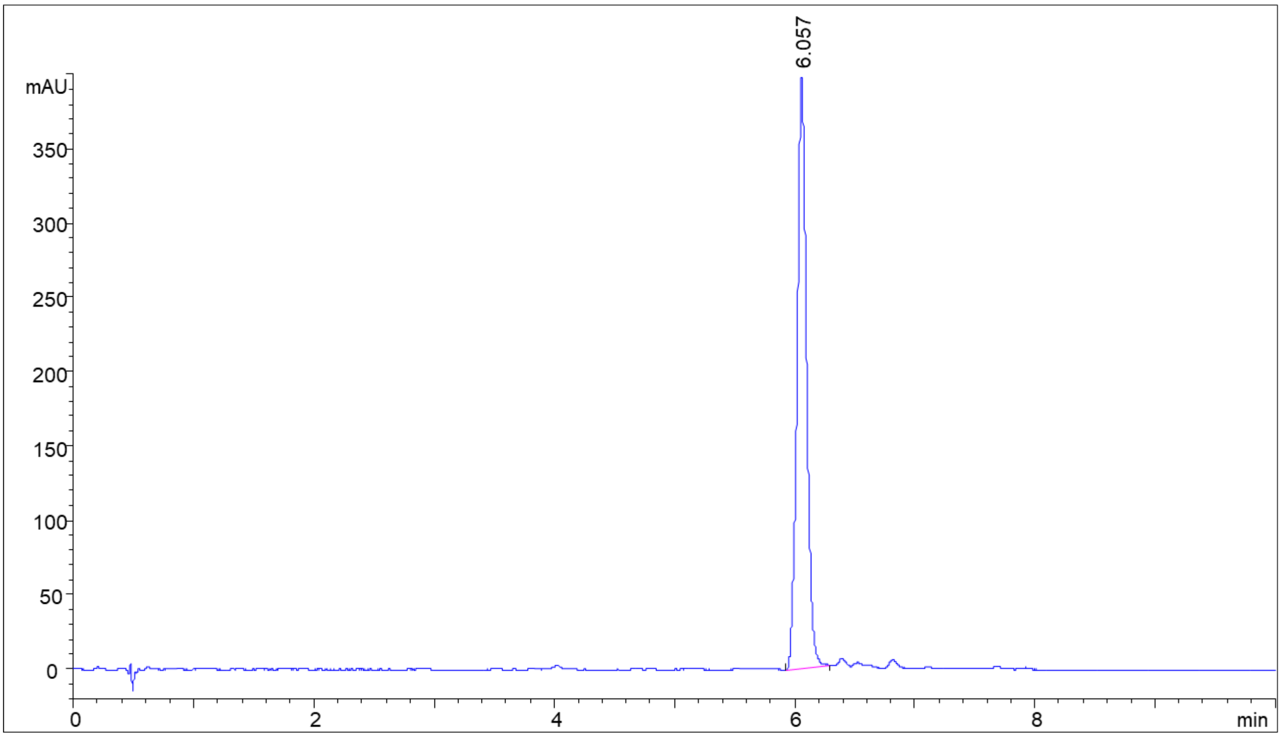

Figure S28: Chromatographic profile of compound **2** monitored at λ=220 nm**.**

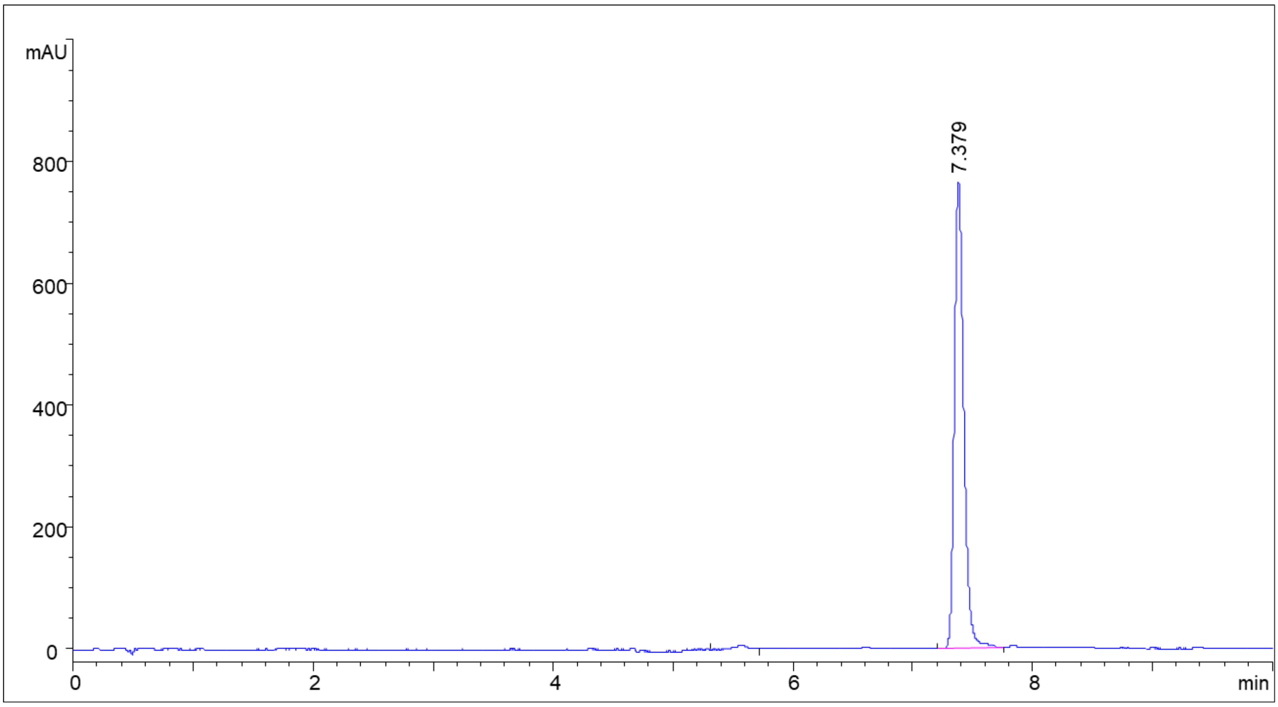

Figure S29: Chromatographic profile of compound **3** monitored at λ=250 nm**.**

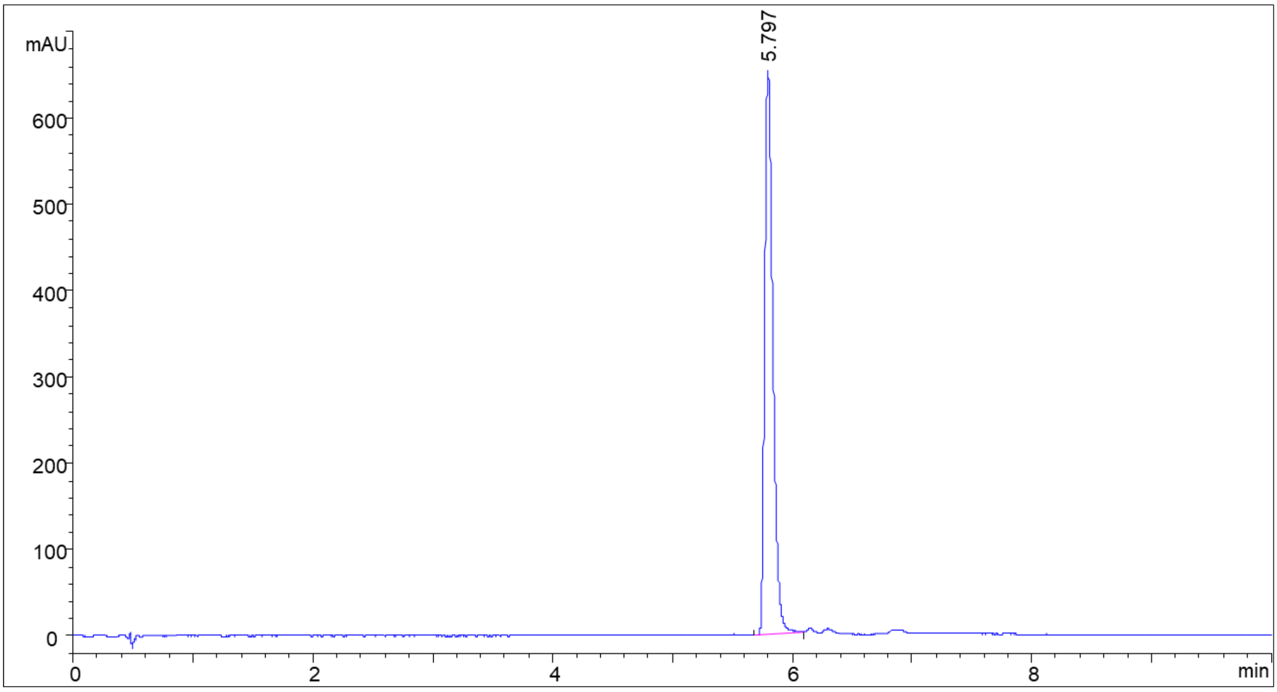

Figure S30: Chromatographic profile of compound **5** monitored at λ=220 nm.

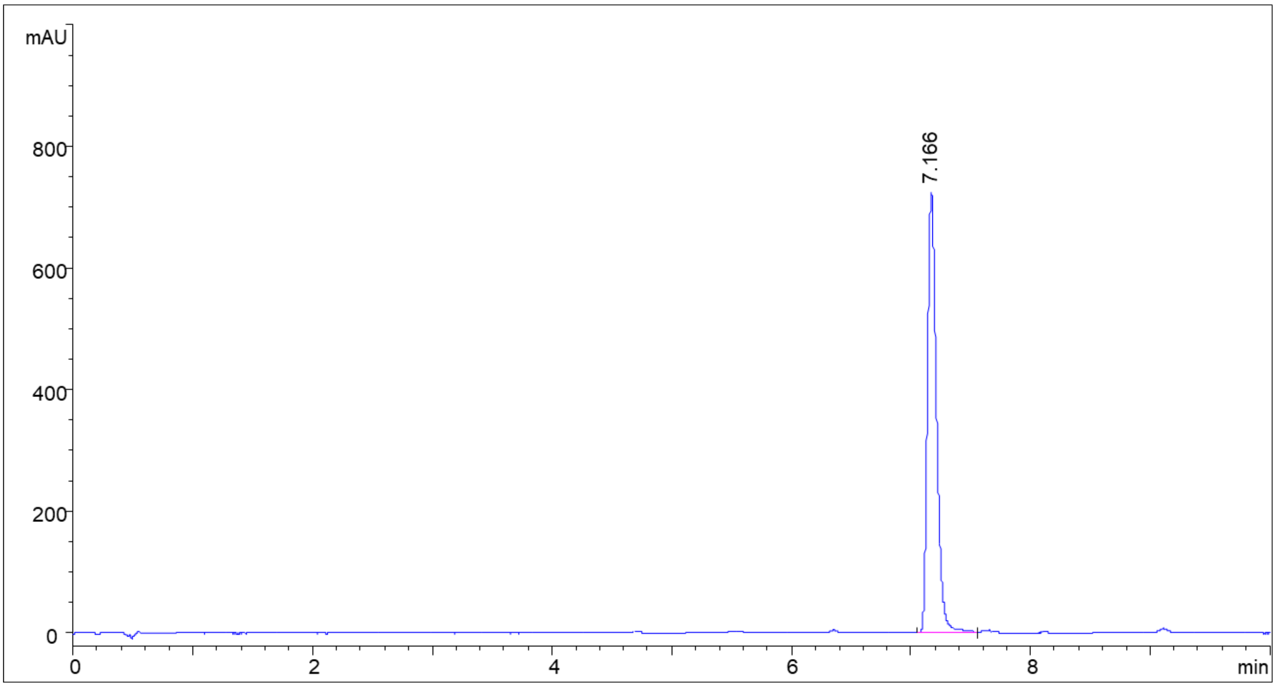

Figure S31: Chromatographic profile of compound **6** monitored at λ=250 nm.

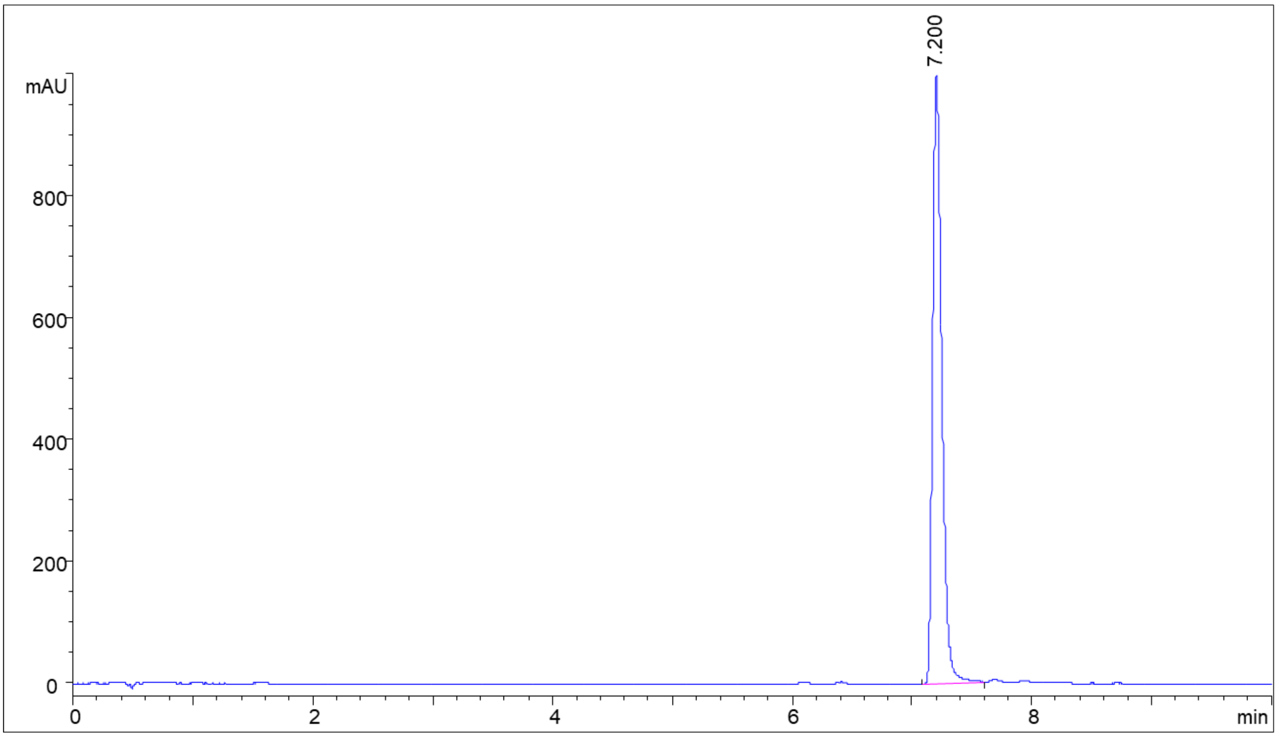

Figure S32: Chromatographic profile of compound **8** monitored at λ=250 nm.

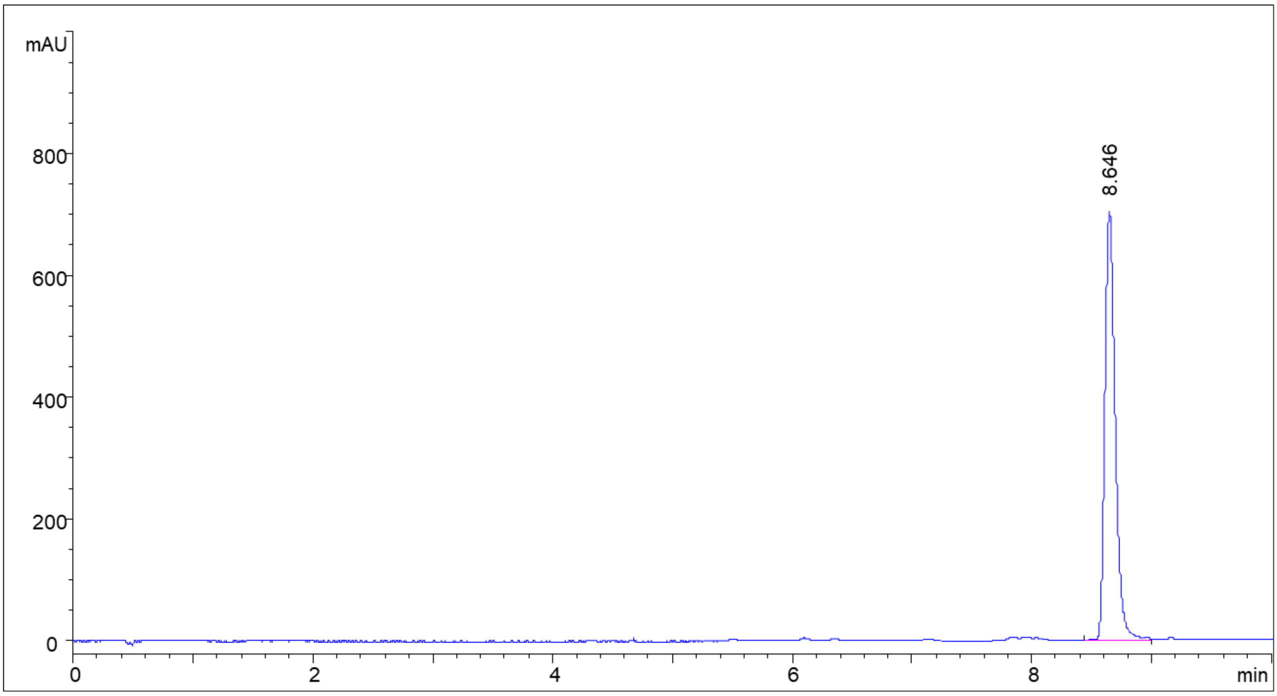

Figure S33: Chromatographic profile of compound **9** monitored at λ=250 nm.

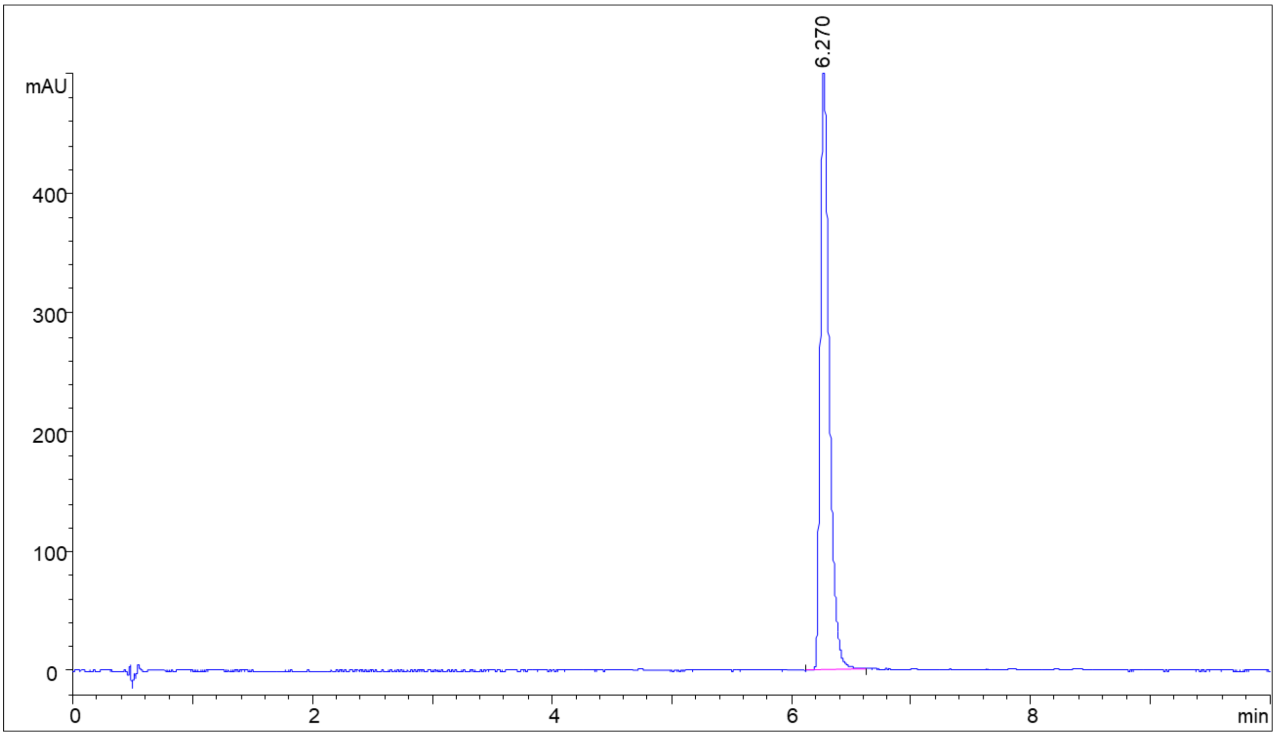

Figure S34: Chromatographic profile of compound **13** monitored at λ=220 nm**.**

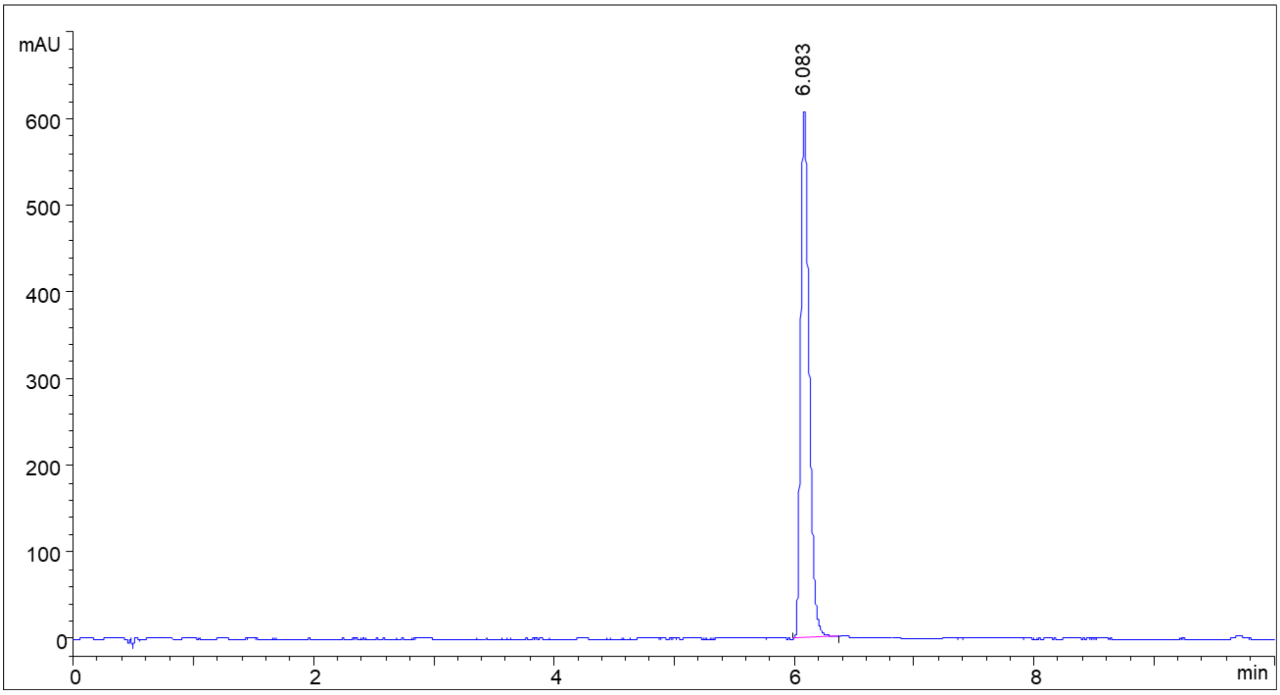

Figure S35: Chromatographic profile of compound **14** monitored at λ=220 nm**.**

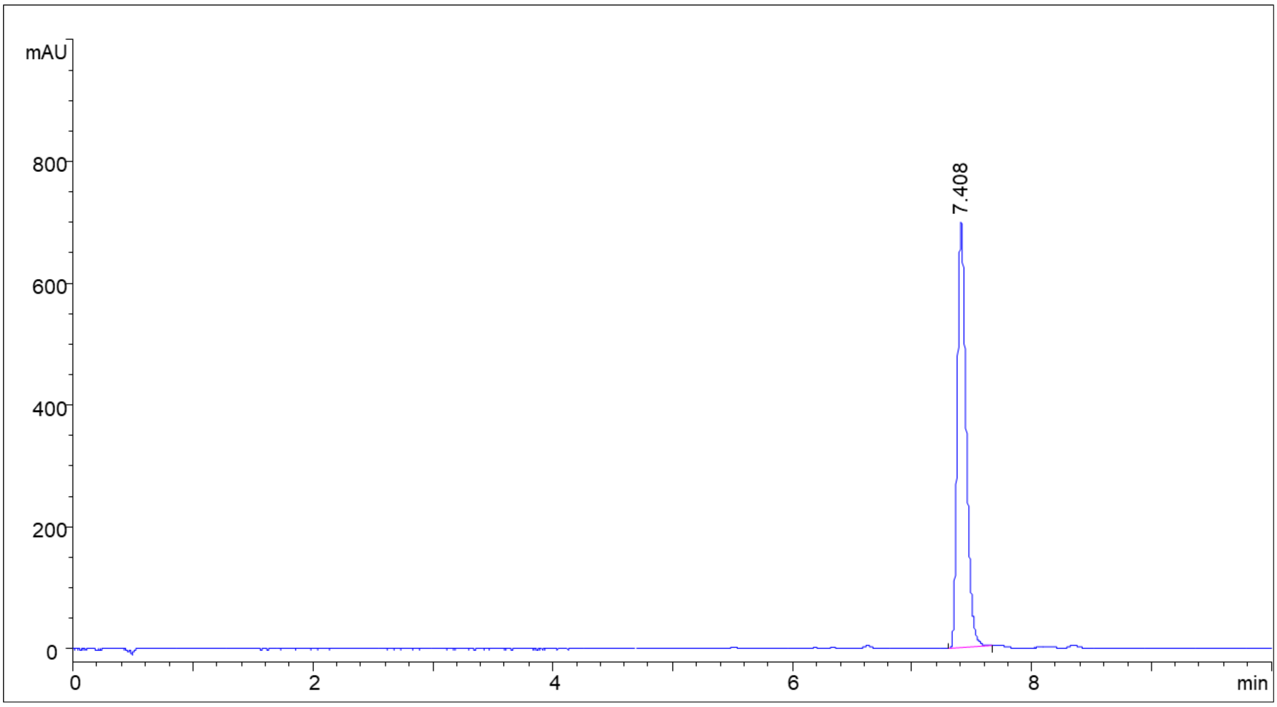

Figure S36: Chromatographic profile of compound **15** monitored at λ=250 nm**.**

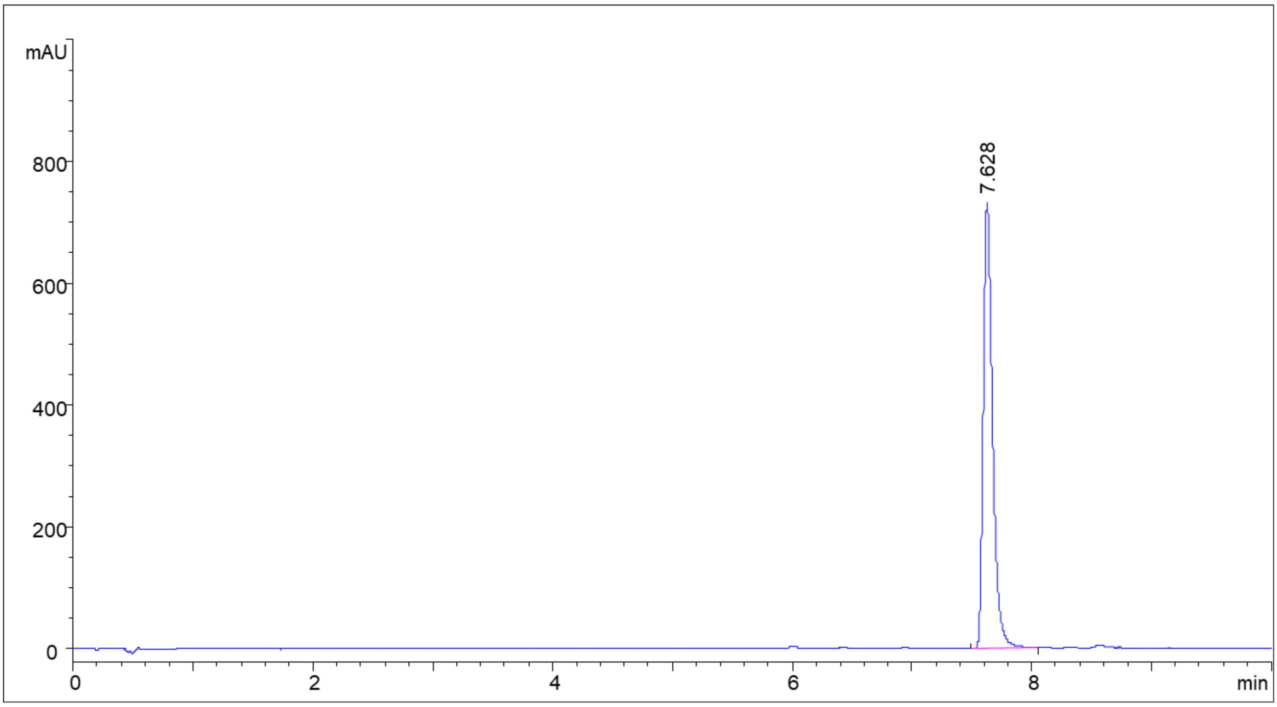

Figure S37: Chromatographic profile of compound **16** monitored at λ=250 nm**.**

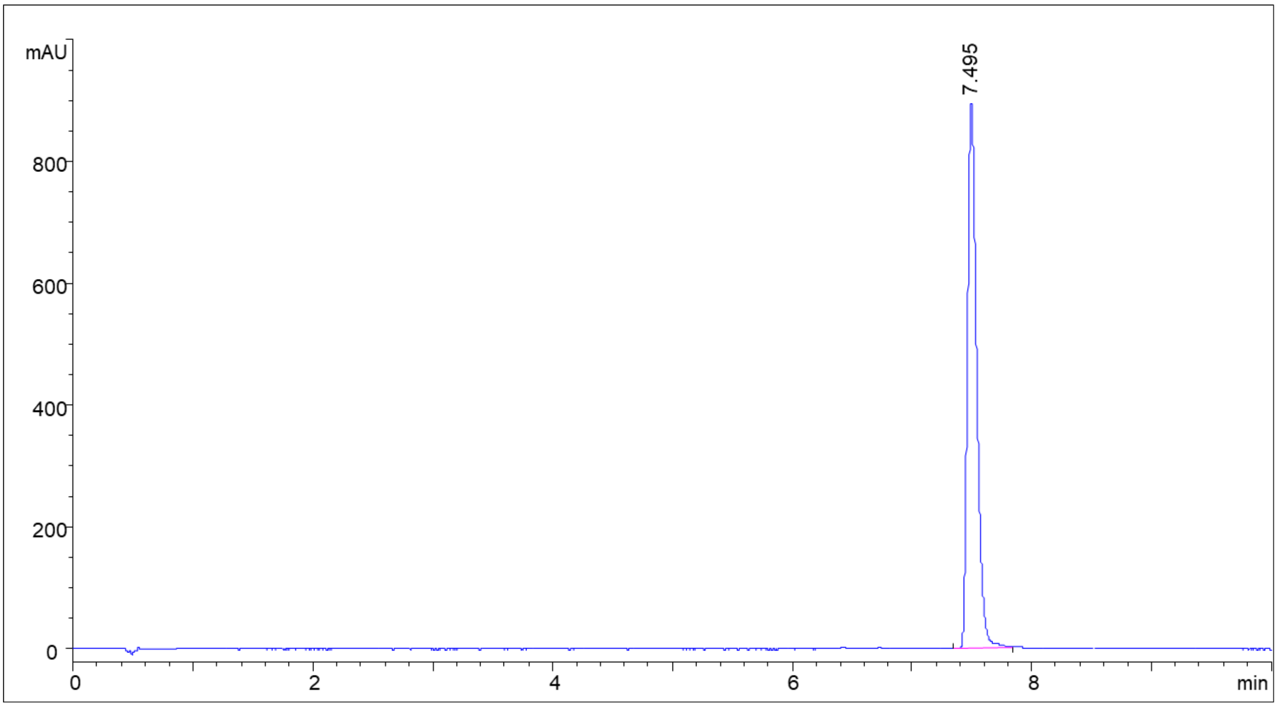

Figure S38: Chromatographic profile of compound **17** monitored at λ=250 nm**.**

UV spectra of compounds **2**, **3**, **5**, **6**, **8**, **9**, **13-17** were reported in Figures S39-S43.

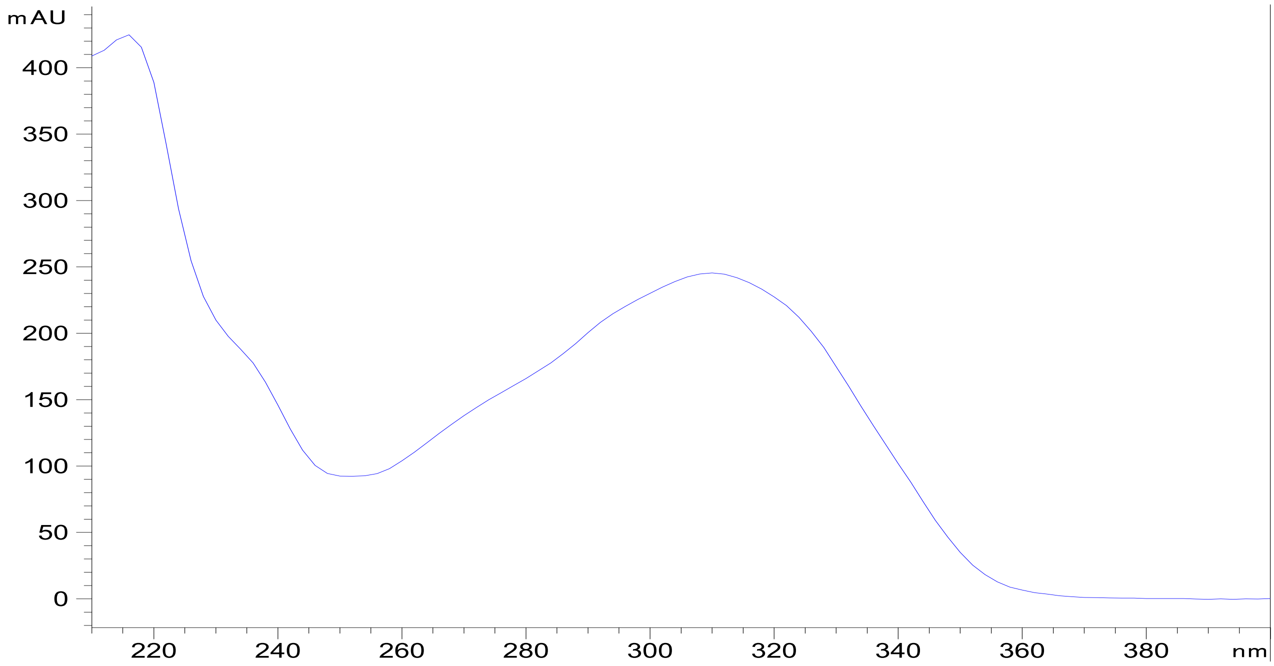

Figure S39: UV spectrum of compounds **2** and **13**.

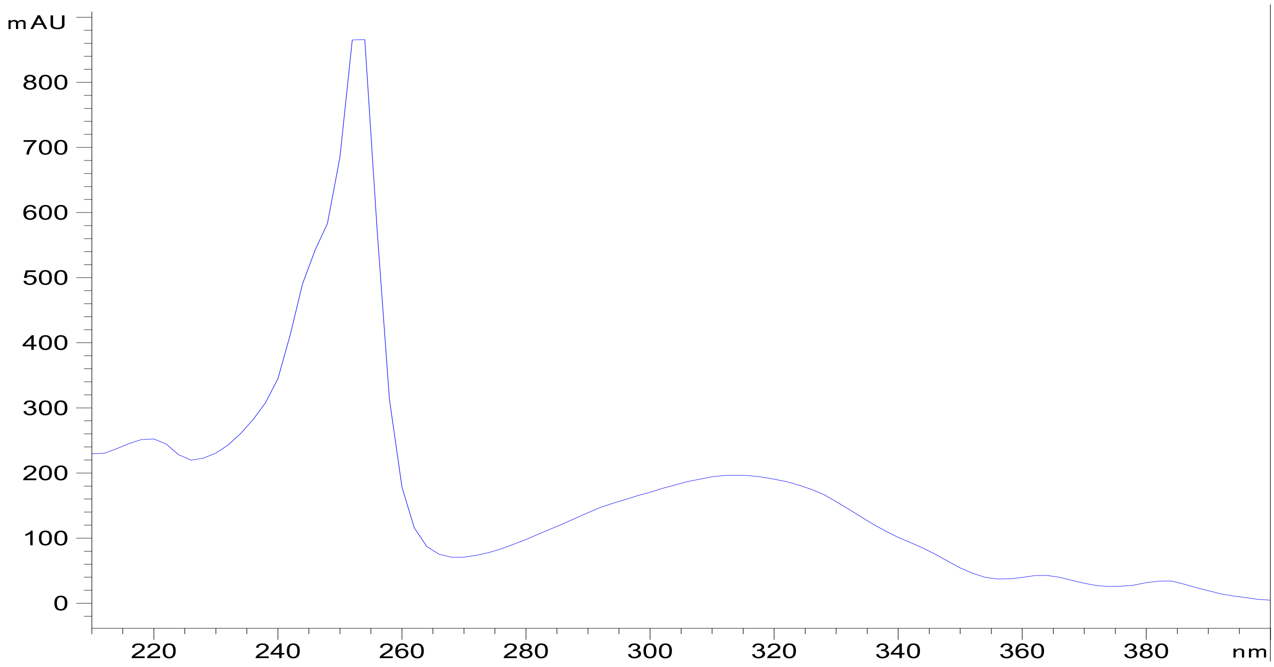

Figure S40: UV spectrum of compounds **3** and **16**.

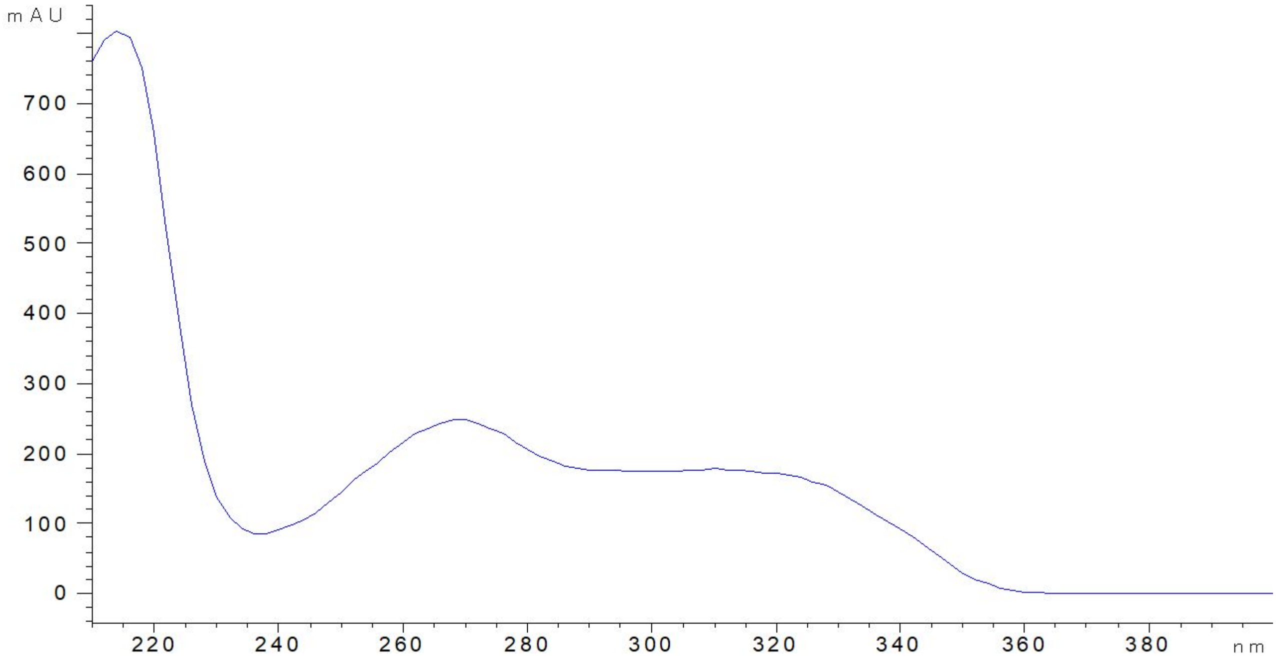

Figure S41: UV spectrum of compounds **5** and **14**.

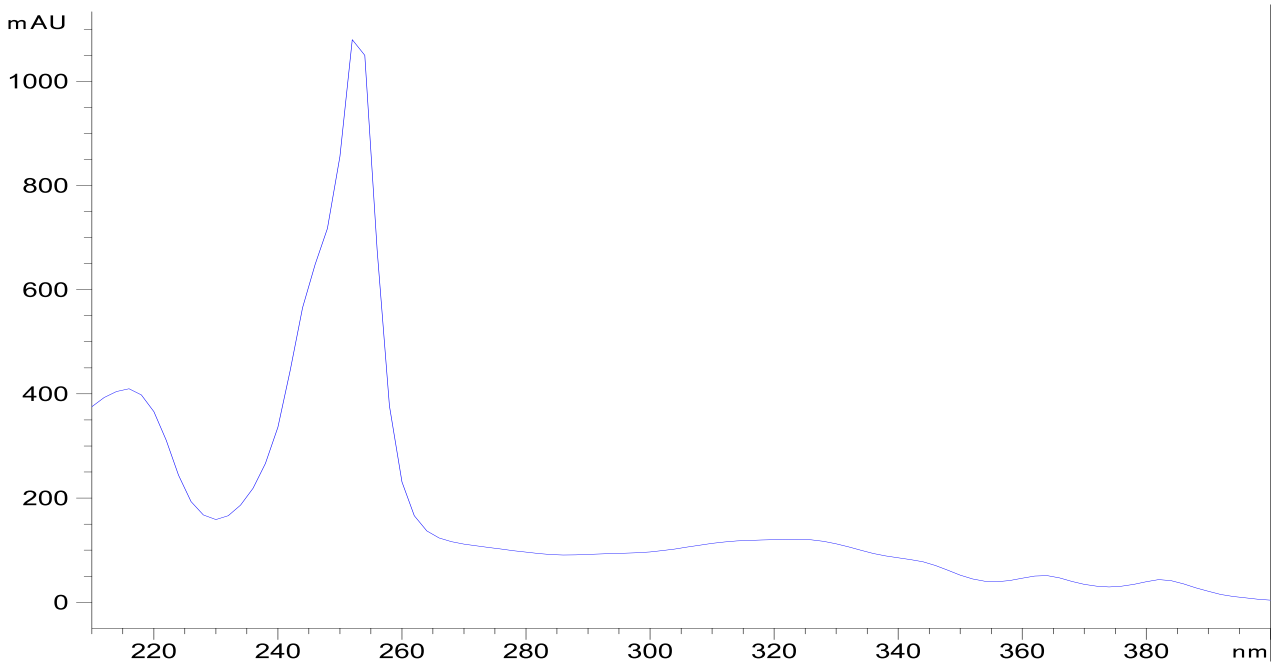

Figure S42: UV spectrum of compounds **6**, **8**, **15** and **17**.

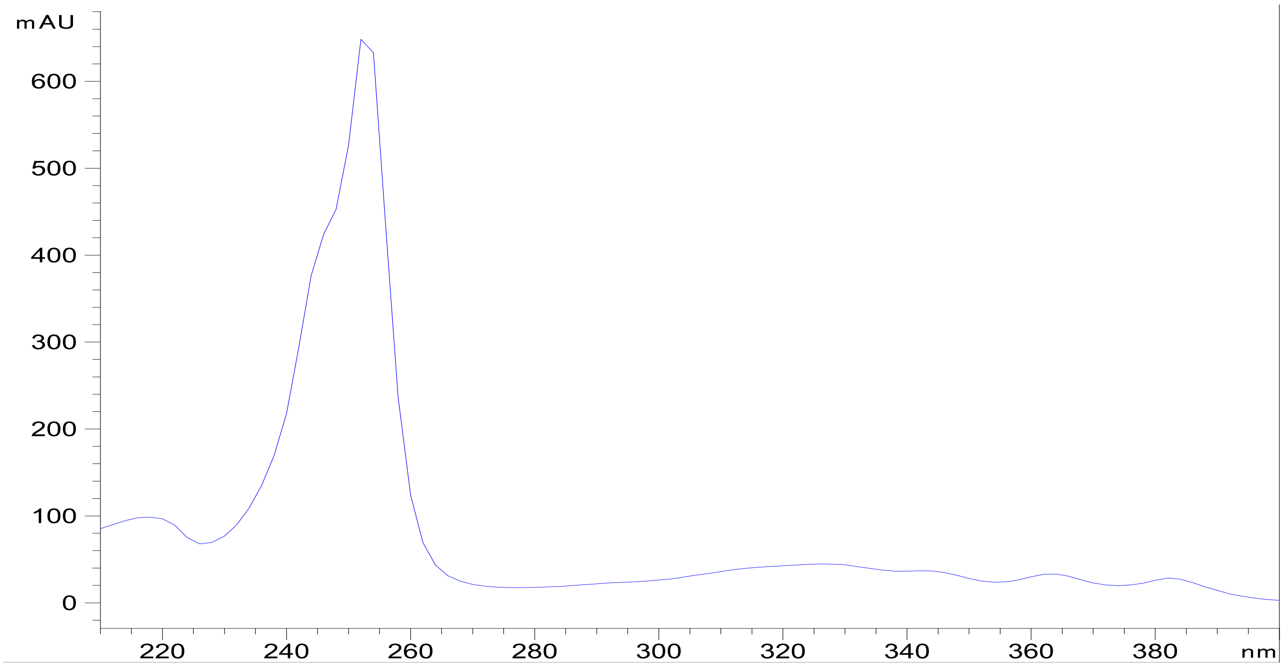

Figure S43: UV spectrum of compound **9**.

Expression levels of P-gp and hCA XII in sensitive HT29 and A549 human cancer cell lines, in resistant wild-type HT29/DOX and A549/DOX cells, and in their P-gp or hCA XII knock-out (KO) counterparts.

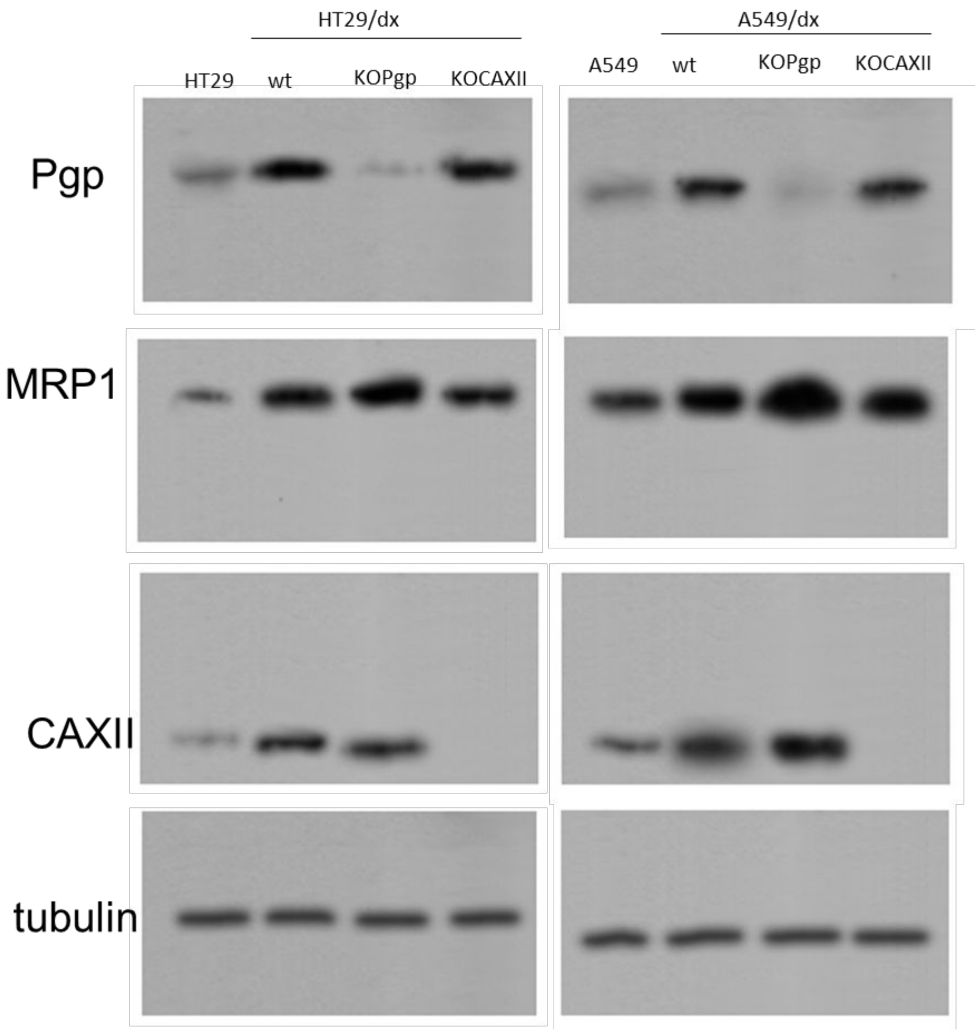

Figure S44. Immunoblotting analysis of P-gp and hCA XII in doxorubicin sensitive HT29 and A549 human cancer cell lines, in wild-type doxorubicin resistant HT29/DOX and A549/DOX cells and in their P-gp or hCA XII knocked-out (KO) counterparts. wt: parental wild-type HT29/DOX and A549/DOX cells. Tubulin was used as control of equal protein loading. The image is representative of three independent experiments.

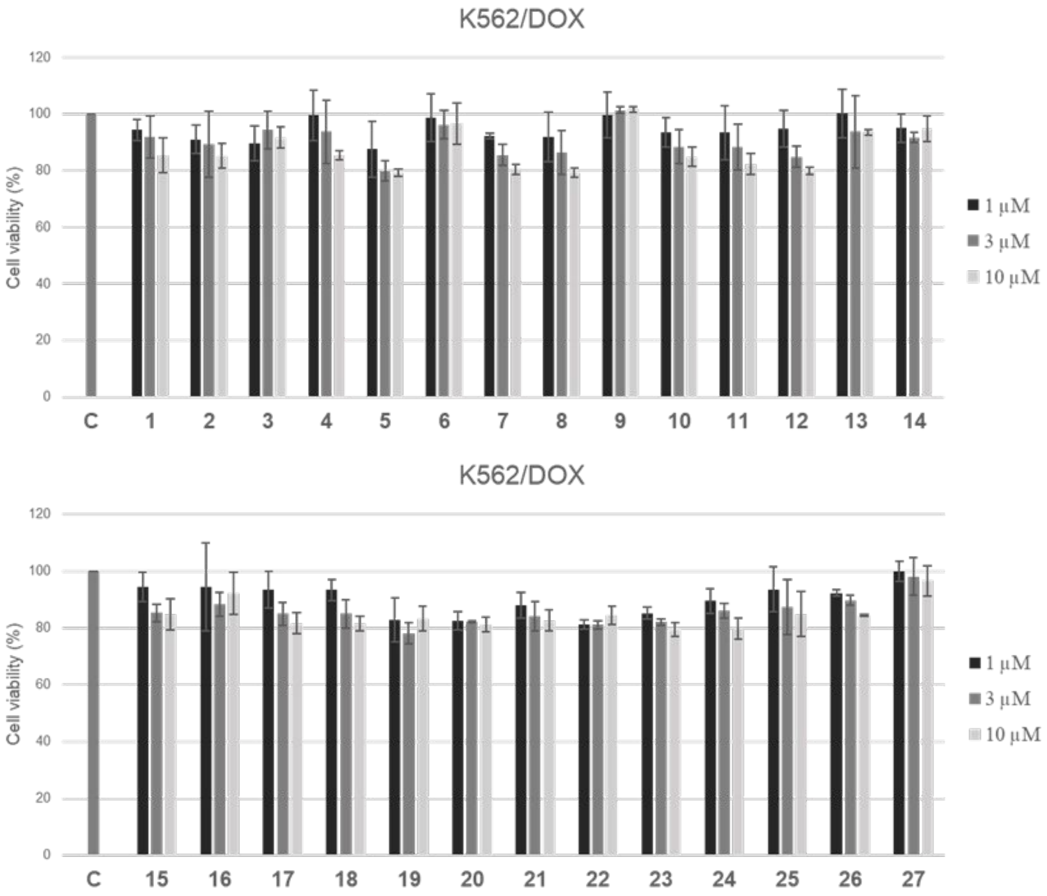

Figure S45. Viability of K562/DOX, cells incubated for 72 h with compounds **1-27** at 1, 3 and 10 μM, measured by the MTT assay, in triplicates. Data are the means + SD (n= 3).

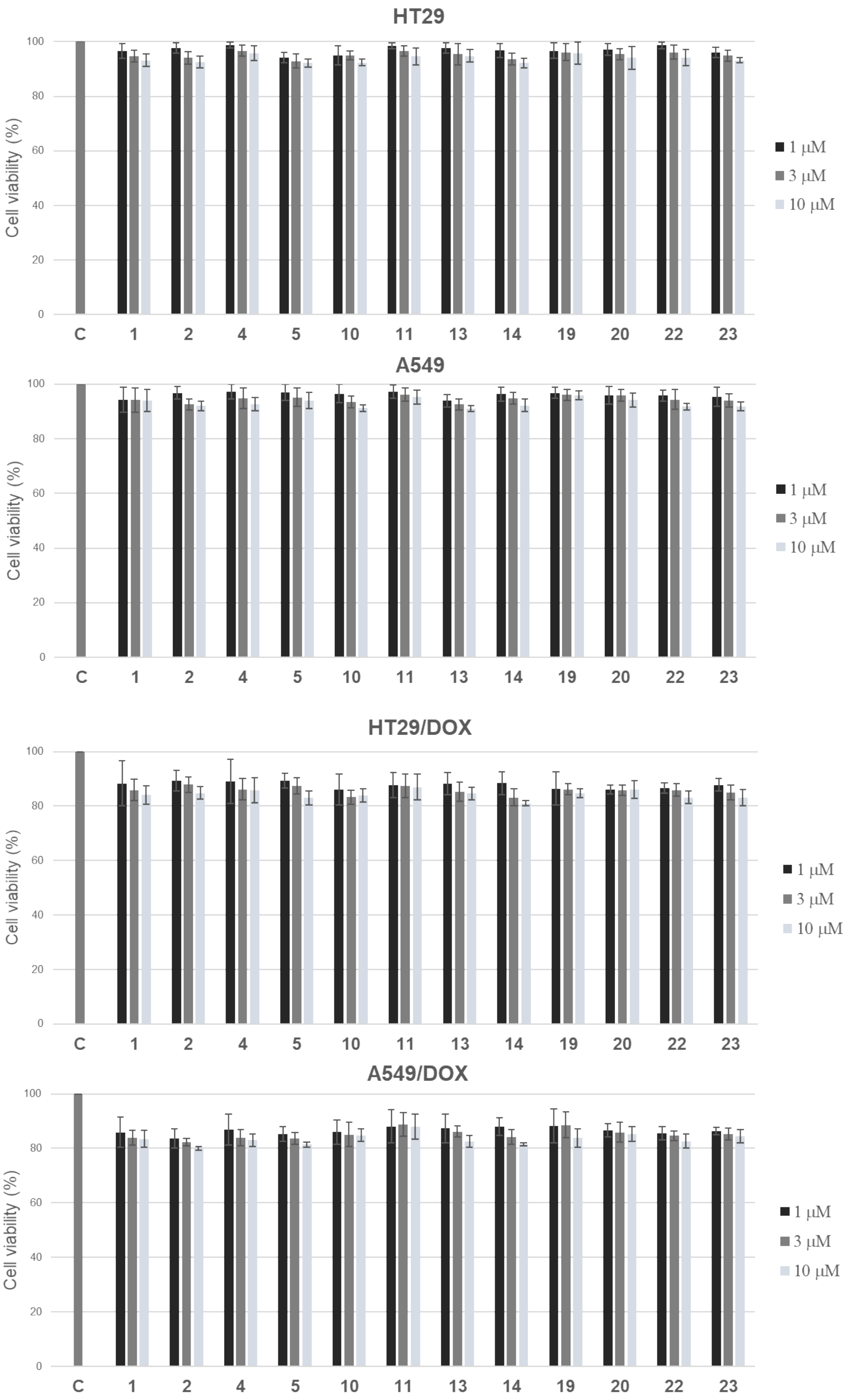

Figure S46. Viability of colon and lung cancer cells. Viability of HT29, A549, HT29/DOX and A549/DOX cells, incubated for 72 h with selected compounds at 1, 3 and 10 μM, measured by the MTT assay, in triplicates. Data are the means ± SD (n= 3).

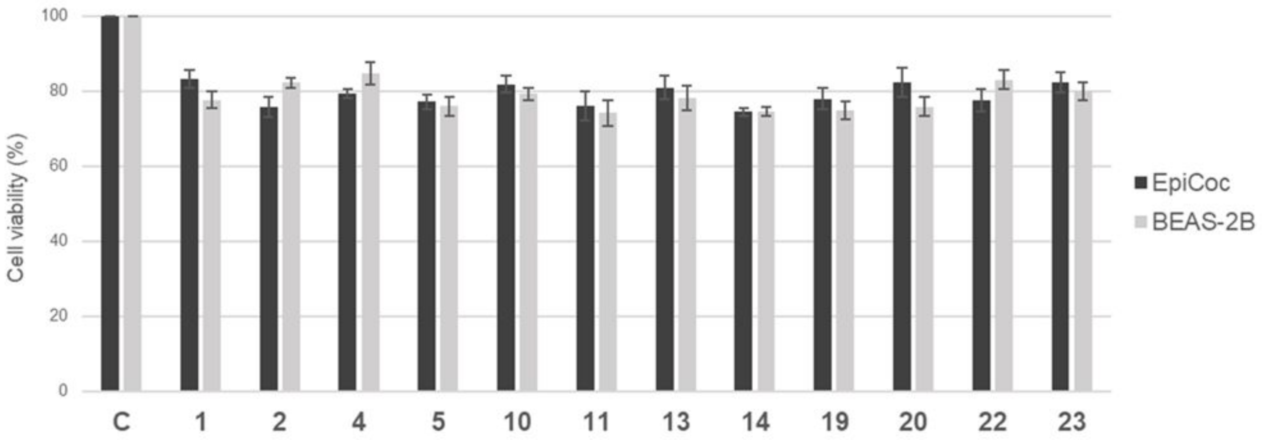

Figure S47. Viability of non-transformed epithelial colon and lung cells. Viability of EpiCoC and BEAS-2B cells, incubated for 72 h with selected compounds at 10 μM, measured by the MTT assay, in triplicates. Data are the means ± SD (n= 3).
